# Are the 50 m Race Segments Changed From Heats to Finals at the 2021 European Swimming Championships?

**DOI:** 10.3389/fphys.2022.797367

**Published:** 2022-07-13

**Authors:** Raúl Arellano, Jesús J. Ruiz-Navarro, Tiago M. Barbosa, Gracia López-Contreras, Esther Morales-Ortíz, Ana Gay, Óscar López-Belmonte, Ángela González-Ponce, Francisco Cuenca-Fernández

**Affiliations:** ^1^ Aquatics Lab, Department of Physical Education and Sports, Faculty of Sport Sciences, University of Granada, Granada, Spain; ^2^ Department of Sport Sciences, Instituto Politécnico de Bragança, Bragança, Portugal; ^3^ Research Centre in Sports, Health and Human Development, Vila Real, Portugal

**Keywords:** race analysis, sprint swimming, start, performance, technique, kinematic

## Abstract

This study explored in the 50 m races of the four swimming strokes the performance parameters and/or technical variables that determined the differences between swimmers who reach the finals and those who do not. A total of 322 performances retrieved from the 2021 Budapest European championships were the focus of this study. The results of the performances achieved during the finals compared to the heats showed that the best swimmers did not excel during the heats, as a significant progression of performance was observed in most of the strokes as the competition progressed. Specifically, combining men and women, the swimmers had in freestyle a mean coefficient of variation (CV) of ∼0.6%, with a mean range of performance improvement (∆%) of ∆ = ∼0.7%; in breaststroke a mean CV of ∼0.5% and ∆ = −0.2%; in backstroke a mean CV of ∼0.5% and ∆ = −0.6%, and; in butterfly a mean CV of ∼0.7% and ∆ = −0.9%. For all strokes, it was a reduction of the underwater phase with the aim of increasing its speed. However, this result was not always transferred to the final performance. In any case, most of the swimmers tried to make improvements from the start of the race up to 15 m. Furthermore, the swimmers generated an overall increase in stroke rate as the rounds progressed. However, a decrease in stroke length resulted and, this balance appeared to be of little benefit to performance.

## 1 Introduction

In the sport of swimming, race analysis, when combined with video sequences, provides crucial information in the development of swimmers’ performance (Gonjo and Olstad, 2021). Therefore, race performances are often analyzed during or after a championship and compared with those of other events to conduct changes in race strategy or technique for the enhancement of future events (Arellano et al., 1994; Marinho et al., 2009). In this sense, during major championships is required that swimmers qualify from the initial round (heats) to the following rounds (semi-finals and/or finals) (Tijani Jed et al., 2021; Cuenca-Fernández et al., 2021b), which means that individual performances may differ. In this regard, while the literature has provided sufficient information on the differences between strokes or distances (Morais et al., 2019; Gonjo and Olstad, 2021), or performance variability in middle- and long-distance swimming events (Hopkins et al., 1999; Skorski et al., 2013; Skorski et al., 2014), no attention has been paid to different strokes of the shorter sprint events (i.e., 50 m freestyle, breaststroke, backstroke and butterfly), probably due to only sprint freestyle is included in the Olympic swimming events list.

A widely held notion in international swimming is that progression between rounds is necessary to ensure that a swimmer qualifies from the heats to the semi-finals and then to the final, when medals are decided (Mujika et al., 2019). For instance, swimmers who participated at the 2004 Athens Olympics were 0.58% slower compared to their qualifying times (Issurin et al., 2008); however, medallists and finalists were able to progress between rounds by 0.35 and 0.12%, respectively. On this variability in performance, known as the intra-athlete coefficient of variation (CV), it has previously been reported that in closely matched competitions where swimmers strive to win a medal or reach a final, they must improve their performance by at least ∼0.5% for that change to have an impact on performance (Stewart and Hopkins, 2000; Trewin et al., 2004). In this regard, a CV of ∼0.5 and −0.6% was observed in United States and Australian Olympic swimmers in 50 and 100 m freestyle, respectively (Pyne et al., 2004). Thus, considering the evolution and all the rules’ modifications in the last 15 years, it is necessary to know whether these variations would occur nowadays in a sample of international swimmers. If so, this raises the question of where do swimmers manage such changes over the race?

In short-duration sports, such as the 50 m swimming, an all-out strategy is often employed (Abbiss and Laursen, 2008; McGibbon et al., 2018; Morais et al., 2021); despite the short duration, fatigue evoke a decrease in swim speed throughout the race (Morais et al., 2021). In this regard, planning and executing a proper race strategy is a key factor to excel in competitive swimming (Morais et al., 2019). It was recently shown that during the European Swimming Championships 2021, swimmers competing in the 100 and 200 m events progressed in their performance from round to round by increasing performance in the first key-moments of the race (Cuenca-Fernández et al., 2021b), indicating that the fastest swimmers did not perform at their best from the very beginning until they were trying to reach the final or win a medal. This strategy was suggested as a possible way to save energy that could allow swimmers to excel when needed (Stewart and Hopkins, 2000; (Cuenca-Fernández et al., 2021b). Indeed, achieving high performance in competitive swimming requires striking a fine balance between stability and variability of performance because, although swimmers need to achieve consistent results, they also need to be able to successfully adapting their stroke parameters to changes in the performance environment (such as the level of the other contenders) (Simbaña-Escobar et al., 2018). Therefore, although the strategy during the 50 m has previously been indicated as a rapid acceleration at the start followed by a progressive reduction in swim speed throughout the race (McGibbon et al., 2018; Morais et al., 2021), it is unknown whether this strategy happens in all rounds (e.g., even during the heats).

Swimming is a cyclic sport, yet its performance should not be conceived as a whole, but as a series of different segments that make up the race and that depend on different biomechanical and physiological adaptations (Hay et al., 1983; Marinho et al., 2009). The start, the clean swim, and the finish are the three main segments that make up the 50 m race (Gonjo and Olstad, 2021). However, such analysis can be even more detailed. E.g., the lap time can be divided into sub-sections including the split times, the time from 25 to 50 m (Morais et al., 2021), and the underwater phase. Furthermore, considering that the velocity of swimming is determined by the interplay between the stroke rate and the stroke length (Wakayoshi et al., 1995), the analysis of these stroke patterns may provide additional insights into the final results (Sánchez, Arellano, and Cuenca-Fernández, 2021). On the other hand, given that the best swimmers would be trying to perform at their best during the finals compared to the early rounds of competition, these variables could entail intentional modifications between rounds aimed to progress in performance. Therefore, analysis of each of these segments could provide further information on how swimmers are able to improve their performance throughout the rounds, i.e., progression within competition, in the four different swimming strokes. For that reason, this study aimed to: 1) study the coefficient of variation (CV) and performance progress (%∆) in total time (i.e., T50) in the four different swimming strokes, and; 2) specifically analyze which of the race segments and stroke variables are most modified to achieve improvement across the rounds. It was hypothesized that performance would improve over the rounds, and that these changes would be a consequence of the improvement in the performance variables corresponding to the different segments of the race.

## 2 Materials and Methods

### 2.1 Participants

European swimmers who competed in 50 m individual events at the 2021 Budapest European championships were the focus of this study. As some swimmers competed in more than one event, a total of 322 performances including 56 males (23.78 ± 3.25 years) and 60 females (24.66 ± 4.12 years) were analyzed. Data were gathered from the finalists (eight finalists x three rounds (i.e., heats, semi-final, and final) x four strokes (i.e., butterfly, backstroke, breaststroke, and freestyle) x two sexes (i.e., male and female)), and semi-finalists (16 semifinalists x two rounds (i.e., heats, semi-final) x four strokes (i.e., butterfly, backstroke, breaststroke, and freestyle) x two sexes (i.e., male and female). In one of the 50 butterfly semi-final there was a last-minute withdrawal, but there were two reserves who did not make the tiebreaker, thus, there were nine semifinalists.

### 2.2 Data Collection

Swimmers’ information and the official race times were retrieved from the official publicly available Budapest 2021 European Championships swimming website (http://len.eu). As this study was a retrospective analysis of publicly available data, without any experimental intervention, informed consent and ethical approval from the local committee was not required.

For each event, the results and changes in performance during the three rounds (i.e., heats, semi-finals, and final) were collected to analyse the process of sports performance. A Web Scraping routine in *Python* ^®^ was implemented to obtain the official data. The information was then checked by two independent researchers. To accomplish the first aim, the following variables were calculated using the final times:

- The intra-athlete CV: which represents the random variation in performance between rounds (Hopkins et al., 1999). Three different intra-athlete CVs were obtained: 1) between heats and semi-finals (H-SF); 2) between semi-finals and finals (SF-F), and; 3) between heats and finals (H-F), including all three rounds, total times and performance variables. The CV was calculated using the following equation:

$$\mathrm{CV}=\;\frac{\mathrm{Standard deviation}\left(e.g.,\mathrm{SF and F}\right)\;}{\mathrm{Mean}\left(e.g.,\mathrm{SF and F}\right)}\;\times \;\mathrm{100}$$ (1)

- Relative change (%∆) in performance variables was calculated between rounds using the following equation:

%Δ = Round 2 performance−Round 1 performance  Round 1 performance × 100 (2)

where, *Round 2 performance* refers to the race time achieved on the second round and *Round 1 performance* refers to the race time achieved on the previous round. The criterion for performance progression, no change, or regression was %∆ being lower, equal, or higher than 0, respectively (Mujika et al., 2019).

**FIGURE 1:**
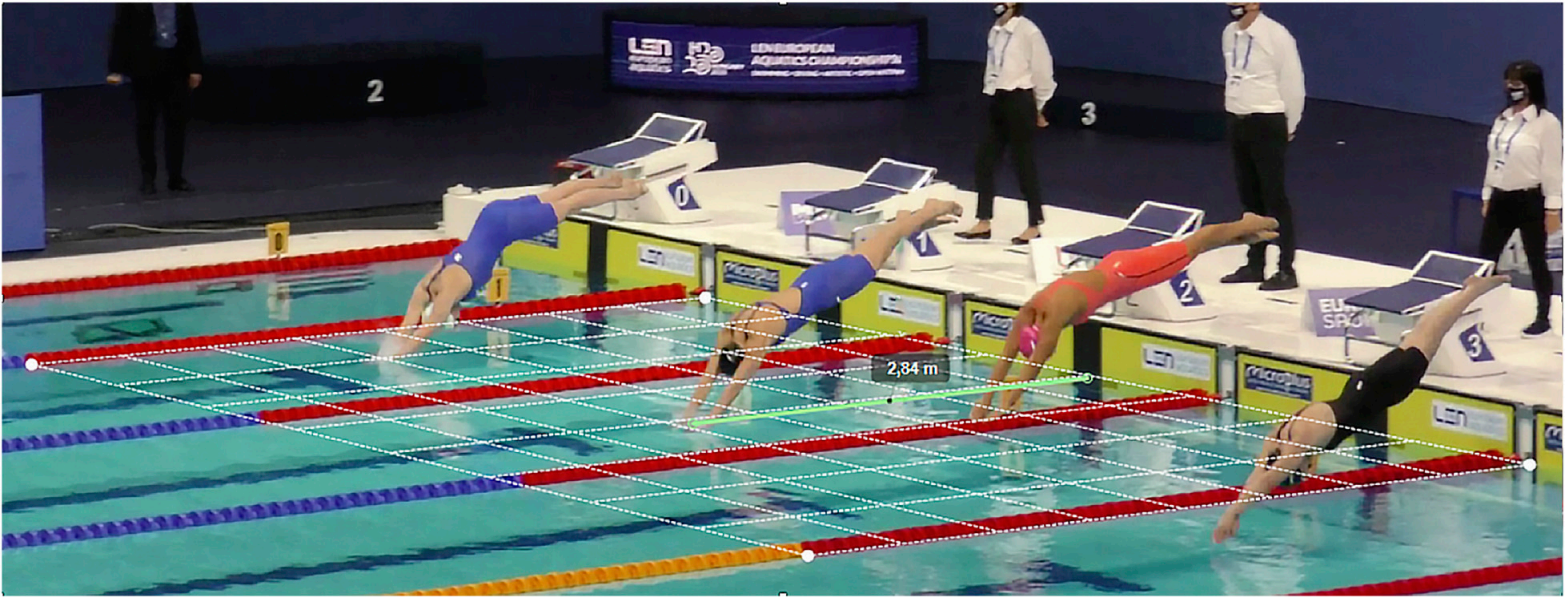
Basic graphical description of the procedure for measuring the swimmer’s entry distance into the water after the start. Similar procedures were used to measure emersion distance.

The performance variables were obtained through indirect photogrammetric methodology, analysing the videos of the swimmer’s performance. This is an indispensable strategy and a major tool for coaches, analysts and researchers to collect qualitative and quantitative data (Smith et al., 2002; O’Donoghue, 2006). All the videos analysed were provided by the championship organisation. A set of 10 pan-tilt-zoom cameras, one for each lane, tracked the swimmer during the race. The video setup included fullHD cameras (1920 × 1,080 pixels resolution, f = 50 Hz Each lane (for each swimmer) had a pan-tilt-zoom camera (Panasonic HC-X1,000 Hybrid O.I.S 4K) tracking the swimmers. Hence, each camera (one per lane) followed along the swimming pool back and forth each swimmer. A calibration zone was defined using the red buoys of the pool lane as a reference (i.e., a distance of 5 m) to correct for the effect of camera position and perspective (Figure 1). A detailed description of the scaling procedures and the calculation of the measurement accuracy can be found in one of the Supplementary Material documents. The starting lights, which were visible from all the cameras, were used to synchronized the official timer with the time-stamp on the race analysis (Morais et al., 2019). The swimmer’s data was obtained after detailed observations by four evaluators through in-house customized software for performance analysis. The Intra-class Correlation Coefficient (ICC) was computed to verify the agreement among evaluators (*n* = 4). This ranged between 0.989 and 0.999, showing high agreement.

### 2.3 Performance Variables

The following variables were measured: Start variables: 1) Reaction time: Defined as the time in seconds (s) from the starting signal until the swimmer moves into the block. Taken from the official results. 2) Flight time: Defined as the time in seconds (s) from when the swimmer leaves the block until the hand touches the water after the start. 3) Entry distance: Defined as the distance in meters (m) between the block wall and the point where the hand touches the water. 4) Underwater time (Und Time): The time in seconds (s) from when the swimmer hand’s touch the water until the swimmer’s head comes out of the water, or if this is not appreciable, when the hands meet at the midpoint of the first stroke. 5) Underwater distance (Und Distance): The distance in meters (m) covered during the underwater phase defined previously. 6) Underwater speed (Und Speed): Obtained by dividing the underwater distance by the time to cover it (m·s^−1^).

Race segments variables: Time 15–50 m (T15 to T50): Defined as the time in seconds (s), from the starting signal, until the swimmer’s head crosses the 1) 15, 2) 25, 2) 35, 4) 45 and 5) 50 m mark (the last one was obtained from the official competition results). 6) Finish time: Defined as the time in seconds (s), from 45 to 50 m. 7) Split25-50 m: Defined as the time in seconds (s), elapsed from 25 to 50 m.

Stroking variables (1,2) Stroke rate (SR): Collected at 15–25 and 35–45 m mark, were obtained using frequency measuring function for each 3 arm strokes and divided by the time elapsed during this action (to obtain the rate in Hertz), and multiplied by 60 (to obtain the rate in cycles/min), 3) final SR: Collected at 45–50 m mark, were obtained using frequency measuring function for each 2 arm strokes and divided by the time elapsed during this action (to obtain the rate in Hertz), and multiplied by 60 (to obtain the rate in cycles/min) (4, 5) average Stroke length (aSL): Collected at 15–25 and 35–45 m mark, were obtained by dividing the mean speed by the mean SR (in Hertz) (to obtain the length in meters/cycle), 6) final SL: Collected at 45–50 m mark, were obtained by dividing the mean speed by the mean SR (in Hertz) (to obtain the length in meters/cycle). The selected variables are noted by the literature on regular basis (Arellano et al., 1994; Mason and Cossor, 2000; Veiga et al., 2014; Morais et al., 2019; Gonjo and Olstad, 2021; Sánchez et al., 2021).

### 2.4 Statistical Analysis

The Shapiro-wilk and Levene test were used to verify the normality and homoscedasticity of the data, respectively. All analyses were conducted differentially by sex (Shapiro et al., 2021). Linear mixed-effects models were applied between rounds (e.g., heats, semi-finals, and final), for all swimmers and performance variables to estimate means (fixed effects) and within-swimmer variations (random effects, modelled as variances) in accordance with Equation 1, as explained in previous studies (Stewart and Hopkins, 2000; Pyne et al., 2004). The fixed main effects were event (50 m freestyle, breaststroke, backstroke and butterfly), performance variables (i.e., the ones presented in Table 1) and rounds (e.g., heats, semi-finals, and final). The performance variables between rounds were compared with repeated-measures ANOVA and the differences between pairs of rounds (e.g., SF to F) were verified with Bonferroni post-hoc test. The effect sizes (_η_^2^) of the obtained variances were calculated and categorized (small = 0.01; medium = 0.06; large = 0.14). Pearson product-moment correlation coefficients (r) between all variables and times performances at 15, 25 and 50 m were obtained and interpreted as follows: 0.1 (low), 0.3 (moderate), 0.5 (large), 0.7 (very high) and 0.9 (nearly perfect) (Hopkins et al., 2009). Simple linear regression analyses were applied to evaluate the associations. All the statistical analyses were conducted in SPSS 24.0 (IBM, Chicago, IL, United States ) with significance level set at *p* < 0.05.

**TABLE 1:** Freestyle performance variables’ results, *p* values, and effect sizes (η^2^) between the different three rounds. Men (M); Women (W) (LEN European Senior Championships 2021).

**Reaction time (s)**			**Heat**	**Semi-final**	**Final**	**Anova**	**η^2^**	**H-SF**	**SF-F**	**H-F**
M	Semifinalist		0.64 ± 0.02	0.63 ± 0.03	-	-	-	0.892	-	-
	Finalist		0.64 ± 0.04	0.62 ± 0.06	0.63 ± 0.03	0.131	0.21	0.041	0.671	0.181
W	Semifinalist		0.65 ± 0.04	0.64 ± 0.04	-	-	-	0.669	-	-
	Finalist		0.66 ± 0.03	0.65 ± 0.02	0.65 ± 0.02	0.670	0.08	0.340	0.557	0.286
**Flight time (s)**			**Heat**	**Semi-final**	**Final**	**Anova**	**η^2^**	**H-SF**	**SF-F**	**H-F**
M	Semifinalist		0.32 ± 0.04	0.33 ± 0.03	-	-	-	0.892	-	-
	Finalist		0.34 ± 0.03	0.35 ± 0.05	0.32 ± 0.05	0.422	0.26	0.999	0.156	0.088
W	Semifinalist		0.29 ± 0.06	0.20 ± 0.34	-	-	-	0.623	-	-
	Finalist		0.28 ± 0.05	0.29 ± 0.04	0.25 ± 0.06	0.003	0.60	0.171	0.012	0.017
**Entry distance (m)**			**Heat**	**Semi-final**	**Final**	**Anova**	**η^2^**	**H-SF**	**SF-F**	**H-F**
M	Semifinalist		3.71 ± 0.18	3.75 ± 0.16	-	-	-	0.524	-	-
	Finalist		3.71 ± 0.19	3.68 ± 0.20	3.68 ± 0.14	0.651	0.13	0.157	0.999	0.480
W	Semifinalist		3.23 ± 0.19	3.26 ± 0.14	-	-	-	0.414	-	-
	Finalist		3.12 ± 0.26	3.21 ± 0.14	3.20 ± 0.24	0.393	0.09	0.073	0.999	0.484
**Underwater Time (s)**			**Heat**	**Semi-final**	**Final**	**Anova**	**η^2^**	**H-SF**	**SF-F**	**H-F**
M	Semifinalist		2.79 ± 0.64	2.61 ± 0.81	-	-	-	0.123	-	-
	Finalist		2.50 ± 0.70	2.52 ± 0.70	2.41 ± 0.66	0.180	0.31	0.611	0.091	0.150
W	Semifinalist		3.42 ± 0.83	3.55 ± 0.73	-	-	-	0.726	-	-
	Finalist		3.47 ± 0.64	3.47 ± 0.73	3.44 ± 0.49	0.542	0.06	0.999	0.866	0.833
**Underwater Distance (m)**			**Heat**	**Semi-final**	**Final**	**Anova**	**η^2^**	**H-SF**	**SF-F**	**H-F**
M	Semifinalist		7.68 ± 1.40	7.42 ± 1.90	-	-	-	0.483	-	-
	Finalist		7.07 ± 1.83	7.37 ± 1.75	7.00 ± 1.70	0.206	0.32	0.182	0.049	0.778
W	Semifinalist		8.60 ± 1.91	8.36 ± 1.66	-	-	-	0.483	-	-
	Finalist		8.69 ± 1.35	8.71 ± 1.80	8.91 ± 0.91	0.607	0.04	0.999	0.778	0.484
**Underwater Speed (m/s)**			**Heat**	**Semi-final**	**Final**	**Anova**	**η^2^**	**H-SF**	**SF-F**	**H-F**
M	Semifinalist		2.81 ± 0.16	2.93 ± 0.26	-	-	-	0.062	-	-
	Finalist		2.84 ± 0.11	2.97 ± 0.30	2.94 ± 0.21	0.208	0.07	0.061	0.340	0.099
W	Semifinalist		2.52 ± 0.13	2.43 ± 0.16	-	-	-	0.059	-	-
	Finalist		2.51 ± 0.13	2.43 ± 0.16	2.60 ± 1.51	0.196	0.58	0.052	0.019	0.152
**Time 15 m (s)**			**Heat**	**Semi-final**	**Final**	**Anova**	**η^2^**	**H-SF**	**SF-F**	**H-F**
M	Semifinalist		5.38 ± 0.14	5.42 ± 0.12	-	-	-	0.309	-	-
	Finalist		5.36 ± 0.06	5.33 ± 0.07	5.34 ± 0.07	0.717	0.07	0.293	0.670	0.670
W	Semifinalist		6.20 ± 0.12	6.16 ± 0.14	-	-	-	0.088	-	-
	Finalist		6.12 ± 0.17	6.05 ± 0.13	6.01 ± 0.18	0.004	0.59	0.027	0.176	0.011
**Time 25 m (s)**			**Heat**	**Semi-final**	**Final**	**Anova**	**η^2^**	**H-SF**	**SF-F**	**H-F**
M	Semifinalist		10.06 ± 0.13	10.05 ± 0.11	-	-	-	0.888	-	-
	Finalist		9.99 ± 0.07	9.87 ± 0.08	9.91 ± 0.14	0.066	0.33	0.027	0.399	0.207
W	Semifinalist		11.48 ± 0.12	11.42 ± 0.10	-	-	-	0.141	-	-
	Finalist		11.32 ± 0.13	11.20 ± 0.11	11.08 ± 0.15	0.001	0.91	0.012	0.012	0.11
**Time 35 m (s)**			**Heat**	**Semi-final**	**Final**	**Anova**	**η^2^**	**H-SF**	**SF-F**	**H-F**
M	Semifinalist		14.80 ± 0.13	14.86 ± 0.10	-	-	-	0.271	-	-
	Finalist		14.68 ± 0.12	14.62 ± 0.10	14.67 ± 0.18	0.648	0.09	0.235	0.528	0.865
W	Semifinalist		16.85 ± 0.09	16.74 ± 0.09	-	-	-	0.017	-	-
	Finalist		16.61 ± 0.16	16.40 ± 0.14	16.34 ± 0.17	0.002	0.89	0.012	0.036	0.011
**Time 45 m (s)**			**Heat**	**Semi-final**	**Final**	**Anova**	**η^2^**	**H-SF**	**SF-F**	**H-F**
M	Semifinalist		19.66 ± 0.10	19.79 ± 0.15	-	-	-	0.068	-	-
	Finalist		19.50 ± 0.14	19.45 ± 0.13	19.50 ± 0.19	0.542	0.09	0.310	0.528	0.944
W	Semifinalist		22.34 ± 0.10	22.25 ± 0.13	-	-	-	0.058	-	-
	Finalist		21.97 ± 0.22	21.74 ± 0.22	21.71 ± 0.26	0.002	0.81	0.012	0.482	0.012
**Time 50 m (s)**			**Heat**	**Semi-final**	**Final**	**Anova**	**η^2^**	**H-SF**	**SF-F**	**H-F**
M	Semifinalist		22.13 ± 0.09	22.14 ± 0.10	-	-	-	0.833	-	-
	Finalist		21.96 ± 0.12	21.78 ± 0.11	21.84 ± 0.18	0.053	0.42	0.017	0.398	0.093
W	Semifinalist		25.00 ± 0.11	24.97 ± 0.14	-	-	-	0.292	-	-
	Finalist		24.57 ± 0.22	24.40 ± 0.22	24.34 ± 0.24	0.002	0.78	0.012	0.159	0.012
**Finish time (s)**			**Heat**	**Semi-final**	**Final**	**Anova**	**η^2^**	**H-SF**	**SF-F**	**H-F**
M	Semifinalist		2.47 ± 0.08	2.34 ± 0.09	-	-	-	0.025	-	-
	Finalist		2.45 ± 0.05	2.33 ± 0.07	2.34 ± 0.06	0.002	0.72	0.012	0.888	0.012
W	Semifinalist		2.65 ± 0.07	2.72 ± 0.09	-	-	-	0.051	-	-
	Finalist		2.60 ± 0.05	2.66 ± 0.07	2.63 ± 0.07	0.197	0.23	0.078	0.141	0.483
**Split 25–50 m (s)**			**Heat**	**Semi-final**	**Final**	**Anova**	**η^2^**	**H-SF**	**SF-F**	**H-F**
M	Semifinalist		12.07 ± 0.10	12.08 ± 0.13	-	-	-	0.779	-	-
	Finalist		11.96 ± 0.08	11.91 ± 0.06	11.92 ± 0.07	0.223	0.18	0.049	0.440	0.725
W	Semifinalist		13.52 ± 0.10	13.54 ± 0.09	-	-	-	0.726	-	-
	Finalist		13.25 ± 0.12	13.19 ± 0.15	13.25 ± 0.13	0.036	0.34	0.018	0.068	0.833
**SR15-25 m (cic/min)**			**Heat**	**Semi-final**	**Final**	**Anova**	**η^2^**	**H-SF**	**SF-F**	**H-F**
M	Semifinalist		61.76 ± 3.58	62.54 ± 3.83	-	-	-	0.779	-	-
	Finalist		61.85 ± 1.09	63.09 ± 1.98	63.04 ± 1.97	0.009	0.60	0.012	0.889	0.025
W	Semifinalist		62.24 ± 3.53	62.05 ± 2.79	-	-	-	0.499	-	-
	Finalist		60.87 ± 3.27	62.12 ± 3.37	62.03 ± 3.64	0.239	0.40	0.028	0.779	0.123
**SR35-45 m (cic/min)**			**Heat**	**Semi-final**	**Final**	**Anova**	**η^2^**	**H-SF**	**SF-F**	**H-F**
M	Semifinalist		58.83 ± 3.31	59.77 ± 3.44	-	-	-	0.069	-	-
	Finalist		59.37 ± 2.18	60.15 ± 1.97	60.88 ± 2.29	0.107	0.29	0.091	0.237	0.123
W	Semifinalist		59.07 ± 3.64	59.32 ± 3.25	-	-	-	0.401	-	-
	Finalist		56.90 ± 2.29	57.64 ± 2.39	58.55 ± 2.84	0.011	0.60	0.036	0.036	0.017
**SR finish (cic/min)**			**Heat**	**Semi-final**	**Final**	**Anova**	**η^2^**	**H-SF**	**SF-F**	**H-F**
M	Semifinalist		57.93 ± 2.55	58.11 ± 3.89	-	-	-	0.917	-	-
	Finalist		57.48 ± 2.96	58.12 ± 2.43	59.77 ± 2.27	0.303	0.27	0.484	0.050	0.123
W	Semifinalist		56.88 ± 3.64	57.59 ± 2.71	-	-	-	0.310	-	-
	Finalist		55.13 ± 2.99	55.68 ± 2.82	55.93 ± 3.18	0.497	0.05	0.484	0.735	0.484
**SL15-25 m (m)**			**Heat**	**Semi-final**	**Final**	**Anova**	**η^2^**	**H-SF**	**SF-F**	**H-F**
M	Semifinalist		2.08 ± 0.11	2.07 ± 0.13	-	-	-	0.779	-	-
	Finalist		2.09 ± 0.06	2.09 ± 0.06	2.08 ± 0.05	0.417	0.20	0.889	0.161	0.161
W	Semifinalist		3.75 ± 0.38	1.82 ± 0.08	-	-	-	0.889	-	-
	Finalist		1.89 ± 0.09	1.87 ± 0.08	1.91 ± 0.09	0.197	0.18	0.208	0.069	0.674
**SL35-45 m (m)**			**Heat**	**Semi-final**	**Final**	**Anova**	**η^2^**	**H-SF**	**SF-F**	**H-F**
M	Semifinalist		2.16 ± 0.12	2.09 ± 0.12	-	-	-	0.093	-	-
	Finalist		2.15 ± 0.08	2.10 ± 0.05	2.07 ± 0.06	0.072	0.52	0.036	0.123	0.036
W	Semifinalist		1.82 ± 0.09	1.90 ± 0.11	-	-	-	0.575	-	-
	Finalist		1.99 ± 0.07	2.00 ± 0.07	1.95 ± 0.08	0.034	0.06	0.401	0.017	0.025
**SL finish (m)**			**Heat**	**Semi-final**	**Final**	**Anova**	**η^2^**	**H-SF**	**SF-F**	**H-F**
M	Semifinalist		2.10 ± 0.16	2.21 ± 0.80	-	-	-	0.025	-	-
	Finalist		2.13 ± 0.11	2.21 ± 0.08	2.14 ± 0.09	0.223	0.24	0.123	0.050	0.779
W	Semifinalist		1.89 ± 0.13	1.92 ± 0.14	-	-	-	0.050	-	-
	Finalist		2.09 ± 0.12	2.03 ± 0.13	2.05 ± 0.14	0.417	0.18	0.093	0.401	0.327

Heat, semi-final, and final (H, SF, and F); stroke rate and length (SR and SL).

## 3 Results

Mean and Standard Deviations (SD) were obtained for all the variables and presented in conjunction with the result of the ANOVA test in Tables 1 to 4, the results for one stroke and both sexes are described in each table.

**TABLE 2:** Backstroke performance variables’ results, *p* values, and effect sizes (η^2^) between the different three rounds. Men (M); Women (W) (LEN European Senior Championships 2021).

**Reaction time (s)**			**Heat**	**Semi-final**	**Final**	**Anova**	**η^2^**	**H-SF**	**SF-F**	**H-F**
M	Semifinalist		0.58 ± 0.03	0.58 ± 0.03	-	-	-	0.550	-	-
	Finalist		0.57 ± 0.05	0.57 ± 0.05	0.56 ± 0.05	0.331	0.19	0.245	0.389	0.121
W	Semifinalist		0.58 ± 0.02	0.57 ± 0.02	-	-	-	0.135	-	-
	Finalist		0.58 ± 0.05	0.57 ± 0.04	0.57 ± 0.04	0.738	0.07	0.480	0.595	0.416
**Flight time (s)**			**Heat**	**Semi-final**	**Final**	**Anova**	**η^2^**	**H-SF**	**SF-F**	**H-F**
M	Semifinalist		0.09 ± 0.05	0.08 ± 0.06	-	-	-	0.210	-	-
	Finalist		0.13 ± 0.05	0.13 ± 0.06	0.11 ± 0.06	0.239	0.28	0.546	0.047	0.287
W	Semifinalist		0.08 ± 0.03	0.10 ± 0.04	-	-	-	0.062	-	-
	Finalist		0.10 ± 0.03	0.10 ± 0.04	0.11 ± 0.06	0.966	0.01	0.999	0.863	0.723
**Entry distance (m)**			**Heat**	**Semi-final**	**Final**	**Anova**	**η^2^**	**H-SF**	**SF-F**	**H-F**
M	Semifinalist		2.60 ± 0.22	2.71 ± 0.18	-	-	-	0.091	-	-
	Finalist		2.88 ± 0.11	2.87 ± 0.10	2.91 ± 0.07	0.311	0.15	0.317	0.216	0.450
W	Semifinalist		2.37 ± 0.11	2.38 ± 0.09	-	-	-	0.705	-	-
	Finalist		2.48 ± 0.13	2.53 ± 0.11	2.48 ± 0.12	0.446	0.01	0.498	0.671	0.865
**Underwater Time (s)**			**Heat**	**Semi-final**	**Final**	**Anova**	**η^2^**	**H-SF**	**SF-F**	**H-F**
M	Semifinalist		4.88 ± 0.41	4.89 ± 0.32	-	-	-	0.779	-	-
	Finalist		4.67 ± 0.22	4.74 ± 0.25	4.56 ± 0.24	0.223	0.23	0.528	0.067	0.263
W	Semifinalist		5.63 ± 0.13	5.59 ± 0.18	-	-	-	0.528	-	-
	Finalist		5.63 ± 0.23	5.55 ± 0.35	5.48 ± 0.36	0.131	0.26	0.263	0.263	0.092
**Underwater Distance (m)**			**Heat**	**Semi-final**	**Final**	**Anova**	**η^2^**	**H-SF**	**SF-F**	**H-F**
M	Semifinalist		11.41 ± 0.88	11.53 ± 1.12	-	-	-	0.779	-	-
	Finalist		10.79 ± 0.60	11.13 ± 0.64	10.71 ± 0.57	0.131	0.35	0.028	0.035	0.622
W	Semifinalist		11.42 ± 0.83	11.38 ± 0.58	-	-	-	0.889	-	-
	Finalist		11.56 ± 0.30	11.58 ± 0.55	11.33 ± 0.62	0.197	0.19	0.833	0.262	0.159
**Underwater Speed (m/s)**			**Heat**	**Semi-final**	**Final**	**Anova**	**η^2^**	**H-SF**	**SF-F**	**H-F**
M	Semifinalist		2.32 ± 0.06	2.34 ± 0.10	-	-	-	0.241	-	-
	Finalist		2.30 ± 0.04	2.34 ± 0.05	2.35 ± 0.06	0.091	0.64	0.128	0.325	0.022
W	Semifinalist		2.04 ± 0.06	2.06 ± 0.07	-	-	-	0.365	-	-
	Finalist		2.05 ± 0.06	2.08 ± 0.08	2.06 ± 0.07	0.452	0.32	0.358	0.681	0.805
**Time 15 m (s)**			**Heat**	**Semi-final**	**Final**	**Anova**	**η^2^**	**H-SF**	**SF-F**	**H-F**
M	Semifinalist		6.09 ± 0.14	6.09 ± 0.20	-	-	-	0.999	-	-
	Finalist		6.09 ± 0.11	5.98 ± 0.13	5.97 ± 0.12	0.030	0.64	0.035	0.933	0.012
W	Semifinalist		7.05 ± 0.19	7.03 ± 0.20	-	-	-	0.672	-	-
	Finalist		6.92 ± 0.20	6.80 ± 0.24	6.88 ± 0.24	0.036	0.33	0.018	0.093	0.441
**Time 25 m (s)**			**Heat**	**Semi-final**	**Final**	**Anova**	**η^2^**	**H-SF**	**SF-F**	**H-F**
M	Semifinalist		11.38 ± 0.15	11.41 ± 0.21	-	-	-	0.307	-	-
	Finalist		11.34 ± 0.13	11.21 ± 0.15	11.16 ± 0.13	0.003	0.80	0.017	0.063	0.012
W	Semifinalist		13.00 ± 0.17	12.99 ± 0.24	-	-	-	0.574	-	-
	Finalist		12.81 ± 0.17	12.66 ± 0.23	12.73 ± 0.22	0.025	0.43	0.018	0.091	0.176
**Time 35 m (s)**			**Heat**	**Semi-final**	**Final**	**Anova**	**η^2^**	**H-SF**	**SF-F**	**H-F**
M	Semifinalist		16.81 ± 0.08	16.87 ± 0.13	-	-	-	0.078	-	-
	Finalist		16.67 ± 0.19	16.51 ± 0.20	16.42 ± 0.17	0.002	0.81	0.025	0.021	0.012
W	Semifinalist		19.04 ± 0.15	19.07 ± 0.30	-	-	-	0.674	-	-
	Finalist		18.79 ± 0.24	18.59 ± 0.23	18.63 ± 0.21	0.021	0.50	0.012	0.483	0.092
**Time 45 m (s)**			**Heat**	**Semi-final**	**Final**	**Anova**	**η^2^**	**H-SF**	**SF-F**	**H-F**
M	Semifinalist		22.41 ± 0.08	22.47 ± 0.13	-	-	-	0.088	-	-
	Finalist		22.13 ± 0.30	21.93 ± 0.29	21.86 ± 0.29	0.001	0.72	0.018	0.092	0.012
W	Semifinalist		25.22 ± 0.12	25.27 ± 0.33	-	-	-	0.933	-	-
	Finalist		24.83 ± 0.29	24.63 ± 0.28	24.72 ± 0.27	0.044	0.50	0.012	0.141	0.123
**Time 50 m (s)**			**Heat**	**Semi-final**	**Final**	**Anova**	**η^2^**	**H-SF**	**SF-F**	**H-F**
M	Semifinalist		25.10 ± 0.11	24.25 ± 1.13	-	-	-	0.499	-	-
	Finalist		24.80 ± 0.36	24.64 ± 0.34	24.59 ± 0.37	0.072	0.55	0.050	0.139	0.017
W	Semifinalist		28.24 ± 0.12	28.34 ± 0.37	-	-	-	0.575	-	-
	Finalist		27.88 ± 0.33	27.69 ± 0.32	27.78 ± 0.26	0.016	0.45	0.012	0.075	0.161
**Finish time (s)**			**Heat**	**Semi-final**	**Final**	**Anova**	**η^2^**	**H-SF**	**SF-F**	**H-F**
M	Semifinalist		2.60 ± 0.07	2.75 ± 0.07	-	-	-	0.035	-	-
	Finalist		2.66 ± 0.07	2.70 ± 0.07	2.72 ± 0.11	0.197	0.19	0.049	0.889	0.326
W	Semifinalist		3.02 ± 0.06	3.07 ± 0.07	-	-	-	0.012	-	-
	Finalist		3.01 ± 0.05	3.05 ± 0.06	3.05 ± 0.09	0.223	0.14	0.079	0.575	0.233
**Split 25–50 m (s)**			**Heat**	**Semi-final**	**Final**	**Anova**	**η^2^**	**H-SF**	**SF-F**	**H-F**
M	Semifinalist		13.71 ± 0.23	13.81 ± 0.24	-	-	-	0.012	-	-
	Finalist		13.45 ± 0.30	13.42 ± 0.28	13.43 ± 0.31	0.798	0.03	0.623	0.726	0.779
W	Semifinalist		15.24 ± 0.17	15.35 ± 0.25	-	-	-	0.080	-	-
	Finalist		15.06 ± 0.25	15.02 ± 0.24	15.04 ± 0.25	0.197	0.14	0.079	0.622	0.441
**SR15-25 m (cic/min)**			**Heat**	**Semi-final**	**Final**	**Anova**	**η^2^**	**H-SF**	**SF-F**	**H-F**
M	Semifinalist		57.06 ± 3.39	57.95 ± 2.89	-	-	-	0.063	-	-
	Finalist		56.60 ± 2.74	57.01 ± 2.61	57.85 ± 3.35	0.005	0.40	0.093	0.093	0.036
W	Semifinalist		56.18 ± 2.67	56.20 ± 2.52	-	-	-	0.401	-	-
	Finalist		53.51 ± 2.21	53.78 ± 2.61	54.41 ± 3.01	0.131	0.39	0.400	0.036	0.069
**SR35-45 m (cic/min)**			**Heat**	**Semi-final**	**Final**	**Anova**	**η^2^**	**H-SF**	**SF-F**	**H-F**
M	Semifinalist		54.10 ± 3.87	54.69 ± 2.91	-	-	-	0.401	-	-
	Finalist		54.01 ± 2.76	54.65 ± 2.31	55.65 ± 3.29	0.008	0.57	0.028	0.036	0.017
W	Semifinalist		54.21 ± 2.36	54.34 ± 3.12	-	-	-	0.484	-	-
	Finalist		52.00 ± 2.99	52.58 ± 3.32	52.69 ± 3.38	0.215	0.21	0.327	0.398	0.043
**SR finish (cic/min)**			**Heat**	**Semi-final**	**Final**	**Anova**	**η^2^**	**H-SF**	**SF-F**	**H-F**
M	Semifinalist		52.24 ± 2.07	53.24 ± 2.28	-	-	-	0.128	-	-
	Finalist		54.56 ± 3.27	53.27 ± 3.01	54.90 ± 3.36	0.485	0.19	0.173	0.176	0.779
W	Semifinalist		53.79 ± 2.54	53.63 ± 3.33	-	-	-	0.889	-	-
	Finalist		51.80 ± 3.92	51.65 ± 3.57	51.79 ± 3.27	0.582	0.01	0.917	0.833	0.753
**SL15-25 m (m)**			**Heat**	**Semi-final**	**Final**	**Anova**	**η^2^**	**H-SF**	**SF-F**	**H-F**
M	Semifinalist		1.99 ± 0.10	1.95 ± 0.09	-	-	-	0.012	-	-
	Finalist		2.02 ± 0.09	2.01 ± 0.08	2.00 ± 0.10	0.607	0.05	0.674	0.779	0.575
W	Semifinalist		1.79 ± 0.07	1.79 ± 0.06	-	-	-	0.575	-	-
	Finalist		1.90 ± 0.08	1.91 ± 0.10	1.88 ± 0.11	0.417	0.19	0.674	0.093	0.263
**SL35-45 m (m)**			**Heat**	**Semi-final**	**Final**	**Anova**	**η^2^**	**H-SF**	**SF-F**	**H-F**
M	Semifinalist		2.05 ± 0.13	2.01 ± 0.08	-	-	-	0.263	-	-
	Finalist		2.09 ± 0.11	2.07 ± 0.08	2.05 ± 0.11	0.197	0.23	0.327	0.208	0.123
W	Semifinalist		1.83 ± 0.08	1.81 ± 0.09	-	-	-	0.161	-	-
	Finalist		1.93 ± 0.11	1.92 ± 0.12	1.93 ± 0.11	0.882	0.04	0.674	0.575	0.999
**SL finish (m)**			**Heat**	**Semi-final**	**Final**	**Anova**	**η^2^**	**H-SF**	**SF-F**	**H-F**
M	Semifinalist		1.92 ± 0.11	1.91 ± 0.14	-	-	-	0.735	-	-
	Finalist		2.06 ± 0.16	2.08 ± 0.10	2.01 ± 0.13	0.325	0.05	0.575	0.050	0.327
W	Semifinalist		1.84 ± 0.09	1.82 ± 0.11	-	-	-	0.575	-	-
	Finalist		1.93 ± 0.13	1.90 ± 0.11	1.90 ± 0.13	0.223	0.07	0.327	0.575	0.263

Heat, semi-final, and final (H, SF, and F); stroke rate and length (SR and SL).

**TABLE 3:** Breaststroke performance variables’ results, *p* values, and effect sizes (η^2^) between the different three rounds. Men (M); Women (W) (LEN European Senior Championships 2021).

**Reaction time (s)**		**Heat**	**Semi-final**	**Final**	**Anova**	**η^2^**	**H-SF**	**SF-F**	**H-F**
M	Semifinalist	0.65 ± 0.02	0.66 ± 0.02	-	-	-	0.202	-	-
	Finalist	0.65 ± 0.03	0.65 ± 0.03	0.65 ± 0.03	0.687	0.07	0.234	0.496	0.916
W	Semifinalist	0.67 ± 0.03	0.67 ± 0.04	-	-	-	0.865	-	-
	Finalist	0.69 ± 0.03	0.67 ± 0.02	0.67 ± 0.03	0.039	0.37	0.016	0.395	0.126
**Flight time (s)**		**Heat**	**Semi-final**	**Final**	**Anova**	**η^2^**	**H-SF**	**SF-F**	**H-F**
M	Semifinalist	0.34 ± 0.01	0.34 ± 0.03	-	-	-	0.306	-	-
	Finalist	0.34 ± 0.05	0.34 ± 0.04	0.33 ± 0.04	0.236	0.19	0.305	0.336	0.121
W	Semifinalist	0.29 ± 0.04	0.28 ± 0.04	-	-	-	0.119	-	-
	Finalist	0.30 ± 0.03	0.31 ± 0.03	0.31 ± 0.05	0.961	0.16	0.914	0.680	0.932
**Entry distance (m)**		**Heat**	**Semi-final**	**Final**	**Anova**	**η^2^**	**H-SF**	**SF-F**	**H-F**
M	Semifinalist	3.87 ± 0.08	3.86 ± 0.14	-	-	-	0.730	-	-
	Finalist	3.81 ± 0.25	3.72 ± 0.30	3.85 ± 0.18	0.595	0.09	0.553	0.309	0.461
W	Semifinalist	3.36 ± 0.43	3.16 ± 0.19	-	-	-	0.088	-	-
	Finalist	3.32 ± 0.12	3.26 ± 0.13	3.28 ± 0.20	0.582	0.05	0.357	0.751	0.671
**Underwater Time (s)**		**Heat**	**Semi-final**	**Final**	**Anova**	**η^2^**	**H-SF**	**SF-F**	**H-F**
M	Semifinalist	4.83 ± 0.67	4.60 ± 0.45	-	-	-	0.123	-	-
	Finalist	4.73 ± 0.51	4.73 ± 0.56	4.53 ± 0.33	0.195	0.23	0.624	0.176	0.106
W	Semifinalist	4.62 ± 0.41	4.49 ± 0.51	-	-	-	0.034		
	Finalist	4.32 ± 0.46	4.32 ± 0.36	4.30 ± 0.35	0.197	0.04	0.889	0.624	0.362
**Underwater Distance (m)**		**Heat**	**Semi-final**	**Final**	**Anova**	**η^2^**	**H-SF**	**SF-F**	**H-F**
M	Semifinalist	10.15 ± 1.41	9.90 ± 0.89	-	-	-	0.482	-	-
	Finalist	10.50 ± 0.70	10.35 ± 1.07	9.71 ± 0.83	0.104	0.37	0.726	0.080	0.035
W	Semifinalist	9.07 ± 0.67	9.07 ± 0.63	-	-	-	0.776	-	-
	Finalist	8.72 ± 0.72	8.80 ± 0.62	8.80 ± 0.46	0.291	0.02	0.726	0.865	0.114
**Underwater Speed (m/s)**		**Heat**	**Semi-final**	**Final**	**Anova**	**η^2^**	**H-SF**	**SF-F**	**H-F**
M	Semifinalist	2.16 ± 0.18	2.17 ± 0.08	-	-	-	0.358	-	-
	Finalist	2.23 ± 0.22	2.19 ± 0.10	2.14 ± 0.11	0.131	0.01	0.526	0.070	0.036
W	Semifinalist	1.99 ± 0.11	2.03 ± 0.07	-	-	-	0.698	-	-
	Finalist	2.01 ± 0.09	2.03 ± 0.08	2.05 ± 0.06	0.291	0.17	0.702	0.751	0.242
**Time 15 m (s)**		**Heat**	**Semi-final**	**Final**	**Anova**	**η^2^**	**H-SF**	**SF-F**	**H-F**
M	Semifinalist	6.29 ± 0.25	6.35 ± 0.19	-	-	-	0.362	-	-
	Finalist	6.23 ± 0.23	6.22 ± 0.24	6.21 ± 0.25	0.303	0.01	0.673	0.498	0.575
W	Semifinalist	7.57 ± 0.22	7.58 ± 0.20	-	-	-	0.866	-	-
	Finalist	7.56 ± 0.22	7.51 ± 0.24	7.47 ± 0.20	0.250	0.18	0.235	0.326	0.161
**Time 25 m (s)**		**Heat**	**Semi-final**	**Final**	**Anova**	**η^2^**	**H-SF**	**SF-F**	**H-F**
M	Semifinalist	12.39 ± 0.13	12.30 ± 0.15	-	-	-	0.125	-	-
	Finalist	12.17 ± 0.22	12.09 ± 0.25	12.11 ± 0.30	0.417	0.11	0.091	0.483	0.400
W	Semifinalist	14.15 ± 0.17	14.05 ± 0.15	-	-	-	0.091	-	-
	Finalist	13.96 ± 0.25	13.90 ± 0.21	13.82 ± 0.17	0.073	0.43	0.183	0.048	0.034
**Time 35 m (s)**		**Heat**	**Semi-final**	**Final**	**Anova**	**η^2^**	**H-SF**	**SF-F**	**H-F**
M	Semifinalist	18.38 ± 0.16	18.28 ± 0.13	-	-	-	0.092	-	-
	Finalist	18.03 ± 0.26	17.92 ± 0.27	17.97 ± 0.35	0.607	0.14	0.106	0.674	0.528
W	Semifinalist	20.78 ± 0.22	20.76 ± 0.17	-	-	-	0.573	-	-
	Finalist	20.41 ± 0.38	20.39 ± 0.36	20.23 ± 0.26	0.024	0.39	0.999	0.036	0.025
**Time 45 m (s)**		**Heat**	**Semi-final**	**Final**	**Anova**	**η^2^**	**H-SF**	**SF-F**	**H-F**
M	Semifinalist	24.45 ± 0.24	24.38 ± 0.20	-	-	-	0.325	-	-
	Finalist	23.90 ± 0.35	23.86 ± 0.33	23.93 ± 0.45	0.542	0.06	0.400	0.499	0.673
W	Semifinalist	27.58 ± 0.35	27.63 ± 0.33	-	-	-	0.017	-	-
	Finalist	27.05 ± 0.44	26.90 ± 0.41	26.88 ± 0.44	0.093	0.40	0.042	0.833	0.042
**Time 50 m (s)**		**Heat**	**Semi-final**	**Final**	**Anova**	**η^2^**	**H-SF**	**SF-F**	**H-F**
M	Semifinalist	27.44 ± 0.22	27.44 ± 0.22	-	-	-	0.933	-	-
	Finalist	26.89 ± 0.35	26.87 ± 0.35	26.95 ± 0.43	0.542	0.09	0.674	0.204	0.400
W	Semifinalist	31.00 ± 0.33	31.07 ± 0.23	-	-	-	0.233	-	-
	Finalist	30.45 ± 0.48	30.35 ± 0.47	30.26 ± 0.43	0.021	0.43	0.208	0.036	0.035
**Finish Time (s)**		**Heat**	**Semi-final**	**Final**	**Anova**	**η^2^**	**H-SF**	**SF-F**	**H-F**
M	Semifinalist	2.99 ± 0.07	3.06 ± 0.14	-	-	-	0.160	-	-
	Finalist	2.98 ± 0.12	3.01 ± 0.08	3.02 ± 0.16	0.417	0.06	0.484	0.889	0.161
W	Semifinalist	3.42 ± 0.08	3.44 ± 0.14	-	-	-	0.674	-	-
	Finalist	3.39 ± 0.15	3.45 ± 0.14	3.38 ± 0.09	0.607	0.18	0.182	0.183	0.726
**Split 25–50 m (s)**		**Heat**	**Semi-final**	**Final**	**Anova**	**η^2^**	**H-SF**	**SF-F**	**H-F**
M	Semifinalist	15.05 ± 0.23	15.14 ± 0.28	-	-	-	0.049	-	-
	Finalist	14.71 ± 0.22	14.77 ± 0.20	14.84 ± 0.28	0.223	0.35	0.068	0.183	0.092
W	Semifinalist	16.85 ± 0.21	17.02 ± 0.16	-	-	-	0.035	-	-
	Finalist	16.49 ± 0.29	16.45 ± 0.33	16.44 ± 0.30	0.748	0.17	0.325	0.624	0.176
**SR15-25 m (cic/min)**		**Heat**	**Semi-final**	**Final**	**Anova**	**η^2^**	**H-SF**	**SF-F**	**H-F**
M	Semifinalist	62.38 ± 3.79	64.18 ± 3.72	-	-	-	0.017	-	-
	Finalist	65.83 ± 4.93	66.19 ± 4.98	67.32 ± 5.24	0.088	0.32	0.499	0.123	0.093
W	Semifinalist	58.81 ± 5.38	59.58 ± 5.64	-	-	-	0.263	-	-
	Finalist	63.76 ± 5.50	62.97 ± 4.69	64.34 ± 5.33	0.081	0.23	0.327	0.018	0.263
**SR35-45 m (cic/min)**		**Heat**	**Semi-final**	**Final**	**Anova**	**η^2^**	**H-SF**	**SF-F**	**H-F**
M	Semifinalist	61.84 ± 3.20	61.85 ± 3.23	-	-	-	0.674	-	-
	Finalist	64.39 ± 6.21	65.01 ± 4.94	66.04 ± 4.74	0.044	0.39	0.624	0.018	0.036
W	Semifinalist	57.04 ± 5.62	57.66 ± 5.43	-	-	-	0.273	-	-
	Finalist	62.95 ± 5.31	62.01 ± 4.61	62.91 ± 4.94	0.197	0.21	0.944	0.036	0.171
**SR finish (cic/min)**		**Heat**	**Semi-final**	**Final**	**Anova**	**η^2^**	**H-SF**	**SF-F**	**H-F**
M	Semifinalist	58.96 ± 2.94	61.16 ± 3.37	-	-	-	0.018	-	-
	Finalist	62.68 ± 5.61	63.72 ± 4.93	64.87 ± 5.61	0.197	0.16	0.326	0.327	0.208
W	Semifinalist	56.06 ± 5.21	58.05 ± 5.80	-	-	-	0.345	-	-
	Finalist	61.75 ± 4.51	61.67 ± 1.95	62.03 ± 4.61	0.250	0.01	0.779	0.161	0.893
**SL15-25 m (m)**		**Heat**	**Semi-final**	**Final**	**Anova**	**η^2^**	**H-SF**	**SF-F**	**H-F**
M	Semifinalist	1.58 ± 0.12	1.57 ± 0.08	-	-	-	0.779	-	-
	Finalist	1.54 ± 0.09	1.55 ± 0.12	1.51 ± 0.12	0.197	0.15	0.674	0.263	0.161
W	Semifinalist	1.56 ± 0.11	1.56 ± 0.12	-	-	-	0.770	-	-
	Finalist	1.48 ± 0.14	1.49 ± 0.11	1.47 ± 0.13	0.417	0.06	0.674	0.327	0.484
**SL35-45 m (m)**		**Heat**	**Semi-final**	**Final**	**Anova**	**η^2^**	**H-SF**	**SF-F**	**H-F**
M	Semifinalist	1.62 ± 0.08	1.62 ± 0.08	-	-	-	0.889	-	-
	Finalist	1.60 ± 0.14	1.59 ± 0.12	1.55 ± 0.11	0.093	0.39	0.484	0.025	0.036
W	Semifinalist	1.59 ± 0.14	1.56 ± 0.16	-	-	-	0.123	-	-
	Finalist	1.51 ± 0.13	1.49 ± 0.09	1.49 ± 0.11	0.882	0.06	0.779	0.779	0.575
**SL finish (m)**		**Heat**	**Semi-final**	**Final**	**Anova**	**η^2^**	**H-SF**	**SF-F**	**H-F**
M	Semifinalist	1.70 ± 0.08	1.60 ± 0.11	-	-	-	0.025	-	-
	Finalist	1.61 ± 0.14	1.57 ± 0.12	1.54 ± 0.14	0.135	0.20	0.263	0.401	0.123
W	Semifinalist	1.57 ± 0.15	1.51 ± 0.19	-	-	-	0.575	-	-
Finalist	1.43 ± 0.13	1.41 ± 0.06	1.43 ± 0.11	0.607	0.07	0.327	0.327	0.779

Heat, semi-final, and final (H, SF, and F); stroke rate and length (SR and SL).

**TABLE 4:** Butterfly performance variables’ results, *p* values, and effect sizes (_η_^2^) between the different three rounds. Men (M); Women (W) (LEN European Senior Championships 2021).

**Reaction time (s)**		**Heat**	**Semi-final**	**Final**	**Anova**	**η^2^**	**H-SF**	**SF-F**	**H-F**
M	Semifinalist	0.65 ± 0.02	0.64 ± 0.02	-	-	-	0.864	-	-
	Finalist	0.62 ± 0.05	0.63 ± 0.06	0.64 ± 0.04	0.772	0.05	0.735	0.917	0.495
W	Semifinalist	0.66 ± 0.02	0.67 ± 0.02	-	-	-	0.233	-	-
	Finalist	0.67 ± 0.04	0.67 ± 0.03	0.66 ± 0.04	0.368	0.10	0.496	0.609	0.167
**Flight time (s)**		**Heat**	**Semi-final**	**Final**	**Anova**	**η^2^**	**H-SF**	**SF-F**	**H-F**
M	Semifinalist	0.38 ± 0.03	0.37 ± 0.04	-	-	-	0.733	-	-
	Finalist	0.36 ± 0.06	0.36 ± 0.07	0.33 ± 0.03	0.576	0.13	0.672	0.395	0.068
W	Semifinalist	0.28 ± 0.05	0.27 ± 0.05	-	-	-	0.258	-	-
	Finalist	0.28 ± 0.05	0.29 ± 0.04	0.28 ± 0.04	0.228	0.11	0.336	0.288	0.779
**Entry distance (m)**		**Heat**	**Semi-final**	**Final**	**Anova**	**η^2^**	**H-SF**	**SF-F**	**H-F**
M	Semifinalist	3.73 ± 0.16	3.70 ± 0.17	-	-	-	0.524	-	-
	Finalist	3.71 ± 0.19	3.68 ± 0.20	3.68 ± 0.14	0.651	0.05	0.157	0.999	0.480
W	Semifinalist	3.14 ± 0.15	3.13 ± 0.16	-	-	-	0.763	-	-
	Finalist	3.10 ± 0.14	3.17 ± 0.08	3.13 ± 0.09	0.692	0.14	0.234	0.414	0.461
**Underwater Time (s)**		**Heat**	**Semi-final**	**Final**	**Anova**	**η^2^**	**H-SF**	**SF-F**	**H-F**
M	Semifinalist	3.30 ± 0.60	3.33 ± 0.50	-	-	-	0.401	-	-
	Finalist	3.31 ± 0.43	3.45 ± 0.64	3.26 ± 0.38	0.875	0.12	0.293	0.674	0.624
W	Semifinalist	4.26 ± 0.74	4.23 ± 0.59	-	-	-	0.779	-	-
	Finalist	4.53 ± 0.35	4.46 ± 0.51	4.44 ± 0.26	0.284	0.08	0.674	0.623	0.128
**Underwater Distance (m)**		**Heat**	**Semi-final**	**Final**	**Anova**	**η^2^**	**H-SF**	**SF-F**	**H-F**
M	Semifinalist	9.23 ± 1.33	9.26 ± 1.19	-	-	-	0.888	-	-
	Finalist	9.10 ± 1.00	9.51 ± 1.23	8.95 ± 0.93	0.035	0.37	0.041	0.028	0.441
W	Semifinalist	9.91 ± 1.61	8.73 ± 3.24	-	-	-	0.260	-	-
	Finalist	10.73 ± 0.73	10.70 ± 0.97	10.72 ± 0.37	0.875	0.01	0.623	0.916	0.779
**Underwater Speed (m/s)**		**Heat**	**Semi-final**	**Final**	**Anova**	**η^2^**	**H-SF**	**SF-F**	**H-F**
M	Semifinalist	2.78 ± 0.19	2.79 ± 0.18	-	-	-	0.541	-	-
	Finalist	2.75 ± 0.18	2.79 ± 0.17	2.75 ± 0.22	0.250	0.26	0.061	0.078	0.741
W	Semifinalist	2.35 ± 0.09	2.24 ± 0.54	-	-	-	0.014	-	-
	Finalist	2.38 ± 0.07	2.41 ± 0.08	2.42 ± 0.09	0.635	0.17	0.513	0.814	0.689
**Time 15 m (s)**		**Heat**	**Semi-final**	**Final**	**Anova**	**η^2^**	**H-SF**	**SF-F**	**H-F**
M	Semifinalist	5.41 ± 0.16	5.43 ± 0.18	-	-	-	0.340	-	-
	Finalist	5.40 ± 0.16	5.35 ± 0.14	5.35 ± 0.17	0.043	0.25	0.105	0.916	0.054
W	Semifinalist	6.36 ± 0.17	6.33 ± 0.21	-	-	-	0.262	-	-
	Finalist	6.10 ± 0.14	6.06 ± 0.09	6.03 ± 0.15	0.343	0.18	0.249	0.396	0.257
**Time 25 m (s)**		**Heat**	**Semi-final**	**Final**	**Anova**	**η^2^**	**H-SF**	**SF-F**	**H-F**
M	Semifinalist	10.49 ± 0.13	10.50 ± 0.14	-	-	-	0.672	-	-
	Finalist	10.43 ± 0.12	10.35 ± 0.12	10.34 ± 0.17	0.026	0.42	0.018	0.888	0.042
W	Semifinalist	12.01 ± 0.18	11.89 ± 0.18	-	-	-	0.011	-	-
	Finalist	11.68 ± 0.21	11.55 ± 0.10	11.53 ± 0.15	0.034	0.41	0.050	0.778	0.017
**Time 35 m (s)**		**Heat**	**Semi-final**	**Final**	**Anova**	**η^2^**	**H-SF**	**SF-F**	**H-F**
M	Semifinalist	15.62 ± 0.09	15.62 ± 0.13	-	-	-	0.999	-	-
	Finalist	15.52 ± 0.11	15.39 ± 0.13	15.38 ± 0.18	0.010	0.48	0.012	0.888	0.017
W	Semifinalist	17.69 ± 0.17	17.58 ± 0.22	-	-	-	0.013	-	-
	Finalist	17.32 ± 0.28	17.17 ± 0.16	17.08 ± 0.19	0.008	0.46	0.067	0.325	0.012
**Time 45 m (s)**		**Heat**	**Semi-final**	**Final**	**Anova**	**η^2^**	**H-SF**	**SF-F**	**H-F**
M	Semifinalist	20.86 ± 0.07	20.83 ± 0.08	-	-	-	0.260	-	-
	Finalist	20.72 ± 0.14	20.56 ± 0.16	20.56 ± 0.19	0.093	0.40	0.058	0.944	0.021
W	Semifinalist	23.48 ± 0.16	23.40 ± 0.26	-	-	-	0.172	-	-
	Finalist	23.06 ± 0.33	22.89 ± 0.18	22.79 ± 0.23	0.036	0.46	0.068	0.123	0.028
**Time 50 m (s)**		**Heat**	**Semi-final**	**Final**	**Anova**	**η^2^**	**H-SF**	**SF-F**	**H-F**
M	Semifinalist	23.50 ± 0.05	23.47 ± 0.07	-	-	-	0.362	-	-
	Finalist	23.36 ± 0.15	23.19 ± 0.16	23.20 ± 0.22	0.030	0.40	0.025	0.999	0.035
W	Semifinalist	26.45 ± 0.17	26.38 ± 0.28	-	-	-	0.123	-	-
	Finalist	25.94 ± 0.31	25.77 ± 0.15	25.66 ± 0.20	0.044	0.43	0.093	0.092	0.050
**Finish time (s)**		**Heat**	**Semi-final**	**Final**	**Anova**	**η^2^**	**H-SF**	**SF-F**	**H-F**
M	Semifinalist	2.63 ± 0.06	2.63 ± 0.09	-	-	-	0.866	-	-
	Finalist	2.64 ± 0.08	2.62 ± 0.07	2.63 ± 0.06	0.587	0.03	0.624	0.752	0.327
W	Semifinalist	2.97 ± 0.08	2.98 ± 0.07	-	-	-	0.406	-	-
	Finalist	2.88 ± 0.09	2.87 ± 0.08	2.87 ± 0.09	0.875	0.01	0.917	0.999	0.673
**Split 25–50 m (s)**		**Heat**	**Semi-final**	**Final**	**Anova**	**η^2^**	**H-SF**	**SF-F**	**H-F**
M	Semifinalist	13.01 ± 0.15	12.97 ± 0.11	-	-	-	0.160	-	-
	Finalist	12.93 ± 0.08	12.84 ± 0.16	12.86 ± 0.13	0.206	0.29	0.092	0.725	0.107
W	Semifinalist	14.43 ± 0.14	14.49 ± 0.16	-	-	-	0.192	-	-
	Finalist	14.13 ± 0.11	13.26 ± 0.11	14.25 ± 0.09	0.034	0.34	0.362	0.058	0.093
**SR15-25 m (cic/min)**		**Heat**	**Semi-final**	**Final**	**Anova**	**η^2^**	**H-SF**	**SF-F**	**H-F**
M	Semifinalist	62.09 ± 3.50	63.19 ± 4.64	-	-	-	0.161	-	-
	Finalist	64.64 ± 2.35	64.91 ± 2.87	65.85 ± 2.69	0.417	0.15	0.327	0.161	0.779
W	Semifinalist	65.48 ± 3.94	66.74 ± 3.70	-	-	-	0.015	-	-
	Finalist	63.42 ± 2.70	64.09 ± 2.66	64.14 ± 2.60	0.012	0.45	0.025	0.999	0.017
**SR35-45 m (cic/min)**		**Heat**	**Semi-final**	**Final**	**Anova**	**η^2^**	**H-SF**	**SF-F**	**H-F**
M	Semifinalist	60.21 ± 3.78	61.09 ± 3.95	-	-	-	0.123	-	-
	Finalist	61.63 ± 2.62	63.35 ± 2.43	63.33 ± 2.22	0.061	0.41	0.035	0.866	0.036
W	Semifinalist	62.15 ± 3.49	62.33 ± 2.85	-	-	-	0.441	-	-
	Finalist	60.16 ± 2.05	61.24 ± 2.34	61.96 ± 1.94	0.002	0.76	0.012	0.123	0.012
**SR finish (cic/min)**		**Heat**	**Semi-final**	**Final**	**Anova**	**η^2^**	**H-SF**	**SF-F**	**H-F**
M	Semifinalist	59.22 ± 2.73	59.63 ± 3.19	-	-	-	0.463	-	-
	Finalist	60.35 ± 1.94	61.80 ± 2.71	60.44 ± 3.01	0.417	0.10	0.327	0.161	0.779
W	Semifinalist	60.20 ± 3.58	60.53 ± 2.07	-	-	-	0.953	-	-
	Finalist	61.09 ± 2.65	60.04 ± 2.90	61.25 ± 2.66	0.140	0.17	0.397	0.092	0.575
**SL15-25 m (m)**		**Heat**	**Semi-final**	**Final**	**Anova**	**η^2^**	**H-SF**	**SF-F**	**H-F**
M	Semifinalist	1.90 ± 0.12	1.88 ± 0.15	-	-	-	0.484	-	-
	Finalist	1.84 ± 0.08	1.85 ± 0.08	1.83 ± 0.06	0.325	0.06	0.889	0.674	0.263
W	Semifinalist	1.62 ± 0.10	1.62 ± 0.10	-	-	-	0.515	-	-
	Finalist	1.69 ± 0.07	1.70 ± 0.05	1.70 ± 0.06	0.882	0.05	0.779	0.889	0.999
**SL35-45 m (m)**		**Heat**	**Semi-final**	**Final**	**Anova**	**η^2^**	**H-SF**	**SF-F**	**H-F**
M	Semifinalist	1.94 ± 0.13	1.92 ± 0.13	-	-	-	0.161	-	-
	Finalist	1.91 ± 0.07	1.88 ± 0.08	1.87 ± 0.05	0.417	0.23	0.123	0.999	0.093
W	Semifinalist	1.70 ± 0.09	1.69 ± 0.09	-	-	-	0.678	-	-
	Finalist	1.77 ± 0.05	1.74 ± 0.07	1.74 ± 0.05	0.072	0.28	0.123	0.889	0.069
**SL finish (m)**		**Heat**	**Semi-final**	**Final**	**Anova**	**η^2^**	**H-SF**	**SF-F**	**H-F**
M	Semifinalist	1.92 ± 0.11	1.91 ± 0.14	-	-	-	0.735	-	-
	Finalist	1.88 ± 0.10	2.08 ± 0.10	1.88 ± 0.11	0.325	0.60	0.575	0.327	0.999
W	Semifinalist	1.68 ± 0.14	1.66 ± 0.07	-	-	-	0.767	-	-
	Finalist	1.70 ± 0.09	1.74 ± 0.09	1.71 ± 0.11	0.930	0.12	0.208	0.069	0.889

Heat, semi-final, and final (H, SF, and F); stroke rate and length (SR and SL).

The values obtained of the linear mixed-effects model analyses, intra-subject CVs and ∆% progression are presented in Tables 5 to 8. A significant progression of performance was observed in most of the events over the rounds (i.e., from heats to semi-finals and then finals). The largest CV and ∆ was noted in butterfly events (CV∼0.7%; ∆ = −0.9%), followed-up by freestyle (CV∼0.6%; ∆∼0.7%), backstroke (CV∼0.5%; ∆ = −0.6%) and breaststroke (CV∼0.5%; ∆ = −0.2%). The CV changed in several key moments related to the start underwater variables. However, it is unclear which variable (distance or time) had a larger partial contribution to underwater speed.

**TABLE 5:** Freestyle intra-athletes’ coefficient of variation (CV) and relative change in performance (%∆). Men (M); Women (W); Heat (H); Semi-final (SF); Final (F) (LEN European Senior Championships 2021).

		H-F (*n* = 8)			H-SF (n = 16)			SF-F (*n* = 8)		
		CV	p	**%** **∆**	CV	p	**%∆**	CV	p	**%∆**
Reaction Time	M	0.09 ± 0.01	0.232	−0.07 ± 0.01	0.08 ± 0.01	0.065	−0.09 ± 0.01	0.15 ± 0.01	0.575	0.06 ± 0.01
	W	0.06 ± 0.01	0.386	−0.03 ± 0.01	0.07 ± 0.01	0.291	−0.03 ± 0.01	0.05 ± 0.01	0.616	0.01 ± 0.01
Flight Time	M	0.10 ± 0.01	0.044*	−0.13 ± 0.01	0.07 ± 0.01	0.871	0.01 ± 0.01	0.12 ± 0.01	0.093	−0.14 ± 0.01
	W	0.10 ± 0.01	0.003*	−0.15 ± 0.01	0.14 ± 0.01	0.451	0.13 ± 0.03	0.13 ± 0.01	0.005	−0.02 ± 0.01
Entry Distance	M	0.25 ± 0.01	0.734	−0.17 ± 0.01	0.16 ± 0.01	0.735	0.02 ± 0.01	0.16 ± 0.01	0.305	−0.14 ± 0.01
	W	0.38 ± 0.01	0.672	0.14 ± 0.01	0.16 ± 0.01	0.067	0.12 ± 0.01	0.23 ± 0.01	0.659	−0.05 ± 0.01
Underwater Time	M	0.40 ± 0.01	0.083	−0.41 ± 0.01	0.70 ± 0.02	0.259	−0.50 ± 0.01	0.41 ± 0.01	0.038*	−0.51 ± 0.01
	W	0.65 ± 0.01	0.847	−0.07 ± 0.01	0.82 ± 0.02	0.505	0.18 ± 0.01	0.68 ± 0.01	0.767	−0.01 ± 0.01
Underwater Distance	M	0.55 ± 0.01	0.570	−0.09 ± 0.01	0.94 ± 0.02	0.926	−0.11 ± 0.01	0.65 ± 0.01	0.025*	−0.81 ± 0.01
	W	0.98 ± 0.01	0.376	0.50 ± 0.01	0.95 ± 0.02	0.602	−0.38 ± 0.01	1.15 ± 0.02	0.688	0.56 ± 0.01
Underwater Velocity	M	3.02 ± 0.02	0.055	2.98 ± 4.62	4.51 ± 0.03	0.060	3.40 ± 7.33	3.59 ± 0.02	0.712	−1.16 ± 6.28
	W	2.30 ± 0.03	0.046*	3.12 ± 4.14	3.94 ± 0.04	0.097	−3.88 ± 8.99	3.72 ± 0.04	0.036*	3.40 ± 7.20
Time 15 m	M	0.25 ± 0.01	0.517	−0.11 ± 0.01	0.23 ± 0.01	0.957	0.01 ± 0.01	0.16 ± 0.01	0.894	0.04 ± 0.01
	W	0.31 ± 0.01	0.001*	−0.44 ± 0.01	0.19 ± 0.01	0.002*	−0.21 ± 0.01	0.20 ± 0.01	0.126	−0.17 ± 0.01
Time 25 m	M	0.42 ± 0.01	0.049*	−0.35 ± 0.01	0.33 ± 0.01	0.034*	−0.29 ± 0.01	0.26 ± 0.01	0.752	0.18 ± 0.01
	W	0.68 ± 0.01	0.001*	−0.98 ± 0.01	0.27 ± 0.01	0.001*	−0.36 ± 0.01	0.35 ± 0.01	0.001*	−0.50 ± 0.01
Time 35 m	M	0.36 ± 0.01	0.551	−0.03 ± 0.01	0.36 ± 0.01	0.864	0.02 ± 0.01	0.40 ± 0.01	0.989	0.21 ± 0.01
	W	0.79 ± 0.01	0.001*	−1.12 ± 0.01	0.44 ± 0.01	0.001*	−0.63 ± 0.01	0.23 ± 0.01	0.010*	−0.27 ± 0.01
Time 45 m	M	0.34 ± 0.01	0.508	0.01 ± 0.01	0.44 ± 0.01	0.326	0.18 ± 0.01	0.40 ± 0.01	0.854	0.24 ± 0.01
	W	0.75 ± 0.01	0.001*	−1.07 ± 0.01	0.49 ± 0.01	0.001*	−0.66 ± 0.01	0.32 ± 0.01	0.241	−0.14 ± 0.01
Time 50 m	M	0.58 ± 0.01	0.009*	−0.53 ± 0.86	0.47 ± 0.01	0.034*	0.19 ± 0.34	0.40 ± 0.01	0.774	0.26 ± 0.69
	W	0.68 ± 0.01	0.001*	−0.96 ± 0.28	0.39 ± 0.01	0.003*	0.21 ± 0.25	0.34 ± 0.01	0.083	−0.25 ± 0.48
Split25-50 m	M	0.29 ± 0.01	0.032*	−0.19 ± 0.01	0.30 ± 0.01	0.418	−0.10 ± 0.01	0.22 ± 0.01	0.974	0.08 ± 0.01
	W	0.14 ± 0.01	0.806	0.01 ± 0.01	0.17 ± 0.01	0.449	−0.07 ± 0.01	0.20 ± 0.01	0.085	0.24 ± 0.01
Finish time	M	0.36 ± 0.01	0.001*	−0.53 ± 0.01	0.42 ± 0.01	0.001*	−0.58 ± 0.01	0.17 ± 0.01	0.904	0.03 ± 0.01
	W	0.21 ± 0.01	0.804	0.09 ± 0.01	0.20 ± 0.01	0.003*	−0.22 ± 0.01	0.13 ± 0.01	0.085	−0.12 ± 0.01
SR15-25 m	M	1.47 ± 0.01	0.006*	1.87 ± 1.52	1.72 ± 0.01	0.042*	1.55 ± 2.98	0.92 ± 0.01	0.925	−0.09 ± 1.58
	W	1.93 ± 0.01	0.091	1.82 ± 2.82	1.21 ± 0.01	0.013*	1.23 ± 1.77	0.84 ± 0.01	0.762	−0.16 ± 1.48
SR35-45 m	M	2.47 ± 0.01	0.065	2.41 ± 3.71	1.47 ± 0.01	0.012*	1.41 ± 2.12	1.78 ± 0.01	0.204	1.14 ± 2.74
	W	2.05 ± 0.01	0.006*	2.70 ± 2.09	0.91 ± 0.01	0.021*	0.85 ± 1.33	1.26 ± 0.01	0.017*	1.46 ± 1.47
SR Final	M	4.53 ± 0.02	0.201	3.70 ± 6.40	2.33 ± 0.01	0.461	0.69 ± 4.00	2.49 ± 0.01	0.038*	2.71 ± 3.37
	W	1.84 ± 0.01	0.508	0.77 ± 2.95	2.08 ± 0.01	0.174	1.11 ± 3.30	1.88 ± 0.02	0.634	−0.27 ± 4.15
SL15-25 m	M	0.88 ± 0.01	0.142	−0.75 ± 1.37	1.50 ± 0.01	0.774	−0.26 ± 3.15	0.56 ± 0.01	0.089	−0.56 ± 0.88
	W	2.11 ± 0.01	0.370	0.62 ± 3.51	1.18 ± 0.01	0.280	−0.55 ± 2.08	1.64 ± 0.01	0.037*	1.71 ± 2.29
SL35-45 m	M	3.15 ± 0.02	0.013*	−4.13 ± 3.86	2.35 ± 0.01	0.002*	−2.98 ± 3.18	1.48 ± 0.01	0.111	−1.37 ± 2.20
	W	1.71 ± 0.01	0.021*	−2.13 ± 2.06	0.84 ± 0.01	0.320	0.38 ± 1.43	1.80 ± 0.01	0.002*	−2.58 ± 1.69
SL Final	M	4.07 ± 0.02	0.571	0.62 ± 6.95	3.94 ± 0.02	0.002*	4.26 ± 4.69	2.53 ± 0.01	0.019*	−3.18 ± 3.27
	W	3.07 ± 0.01	0.513	−1.83 ± 5.08	2.94 ± 0.01	0.002*	−3.38 ± 3.83	3.00 ± 0.02	0.284	1.10 ± 5.37

*Significant differences.

**TABLE 6:** Backstroke intra-athlete’s coefficient of variation (CV) and relative change in performance (%∆). Men (M); Women (W); Heat (H); Semi-final (SF); Final (F) (LEN European Senior Championships 2021).

		H-F (*n* = 8)			H-SF (*n* = 16)			SF-F (*n* = 8)		
		CV	p	**%∆**	CV	p	**%∆**	CV	p	**%∆**
Reaction Time	M	0.05 ± 0.01	0.088	−0.06 ± 0.01	0.04 ± 0.01	0.230	−0.02 ± 0.01	0.04 ± 0.01	0.395	−0.03 ± 0.01
	W	0.04 ± 0.01	0.336	−0.02 ± 0.01	0.04 ± 0.01	0.721	−0.03 ± 0.01	0.17 ± 0.01	0.260	−0.02 ± 0.01
Flight Time	M	0.11 ± 0.01	0.327	−0.15 ± 0.01	0.22 ± 0.01	0.298	−0.05 ± 0.01	0.12 ± 0.01	0.804	−0.12 ± 0.01
	W	0.07 ± 0.01	0.381	0.01 ± 0.01	0.07 ± 0.01	0.936	−0.01 ± 0.01	0.04 ± 0.01	0.243	−0.04 ± 0.01
Entry Distance	M	0.12 ± 0.01	0.214	0.06 ± 0.01	0.13 ± 0.01	0.140	0.10 ± 0.01	0.10 ± 0.01	0.103	0.09 ± 0.01
	W	0.21 ± 0.01	0.262	−0.02 ± 0.01	0.12 ± 0.01	0.548	0.03 ± 0.01	0.13 ± 0.01	0.755	−0.04 ± 0.01
Underwater Time	M	0.74 ± 0.01	0.095	−0.49 ± 0.01	0.49 ± 0.01	0.452	0.15 ± 0.01	0.76 ± 0.01	0.029*	−0.75 ± 0.01
	W	0.52 ± 0.01	0.051	−0.52 ± 0.01	0.43 ± 0.01	0.260	−0.23 ± 0.01	0.37 ± 0.01	0.242	−0.23 ± 0.01
Underwater Distance	M	0.67 ± 0.01	0.421	−0.17 ± 0.01	0.55 ± 0.01	0.069	0.43 ± 0.01	0.72 ± 0.01	0.014*	−0.85 ± 0.01
	W	0.61 ± 0.01	0.196	−0.51 ± 0.01	0.51 ± 0.01	0.911	−0.04 ± 0.01	0.61 ± 0.01	0.219	−0.54 ± 0.01
Underwater Velocity	M	1.47 ± 0.01	0.013*	1.72 ± 1.59	1.85 ± 0.02	0.316	0.94 ± 3.91	1.06 ± 0.01	0.880	0.08 ± 1.94
	W	1.03 ± 0.01	0.297	0.56 ± 2.14	1.20 ± 0.01	0.071	0.87 ± 1.92	1.10 ± 0.01	0.391	−0.98 ± 2.43
Time 15 m	M	0.32 ± 0.01	0.001*	−0.45 ± 0.01	0.21 ± 0.01	0.026	−0.21 ± 0.01	0.15 ± 0.01	0.596	−0.04 ± 0.01
	W	0.28 ± 0.01	0.268	−0.15 ± 0.01	0.29 ± 0.01	0.062	−0.25 ± 0.01	0.21 ± 0.01	0.118	0.23 ± 0.01
Time 25 m	M	0.53 ± 0.01	0.001*	−0.76 ± 0.01	0.29 ± 0.01	0.072	−0.22 ± 0.01	0.19 ± 0.01	0.024*	−0.22 ± 0.01
	W	0.35 ± 0.01	0.071	−0.26 ± 0.01	0.38 ± 0.06	0.050*	−0.32 ± 0.01	0.20 ± 0.04	0.097	0.25 ± 0.01
Time 35 m	M	0.72 ± 0.01	0.001*	−1.03 ± 0.01	0.37 ± 0.01	0.164	−0.22 ± 0.01	0.27 ± 0.01	0.003*	−0.37 ± 0.01
	W	0.48 ± 0.01	0.011*	−0.51 ± 0.01	0.44 ± 0.01	0.155	−0.30 ± 0.01	0.18 ± 0.01	0.396	0.13 ± 0.01
Time 45 m	M	0.75 ± 0.01	0.001*	−1.07 ± 0.01	0.41 ± 0.01	0.120	−0.29 ± 0.01	0.28 ± 0.01	0.049*	−0.27 ± 0.01
	W	0.49 ± 0.01	0.021*	−0.47 ± 0.01	0.53 ± 0.01	0.198	−0.34 ± 0.01	0.27 ± 0.01	0.100	0.29 ± 0.01
Time 50 m	M	0.64 ± 0.01	0.002*	−0.85 ± 0.64	0.43 ± 0.01	0.729	−0.07 ± 0.79	0.32 ± 0.01	0.145	−0.20 ± 0.45
	W	0.51 ± 0.01	0.062	−0.36 ± 0.78	0.48 ± 0.01	0.498	−0.18 ± 0.99	0.33 ± 0.01	0.110	0.34 ± 0.49
Split25-50 m	M	0.34 ± 0.01	0.457	−0.10 ± 0.01	0.30 ± 0.01	0.217	0.15 ± 0.01	0.24 ± 0.02	0.886	0.02 ± 0.01
	W	0.17 ± 0.01	0.369	−0.07 ± 0.01	0.25 ± 0.01	0.314	0.13 ± 0.01	0.13 ± 0.01	0.637	0.06 ± 0.01
Finish time	M	0.23 ± 0.01	0.108	0.21 ± 0.01	0.19 ± 0.01	0.001*	0.21 ± 0.01	0.22 ± 0.01	0.946	0.05 ± 0.01
	W	0.23 ± 0.01	0.199	0.14 ± 0.01	0.14 ± 0.01	0.002*	0.16 ± 0.01	0.17 ± 0.01	0.949	0.01 ± 0.01
SR15-25 m	M	1.74 ± 0.01	0.024*	2.09 ± 2.14	1.02 ± 0.01	0.008*	1.14 ± 1.56	1.22 ± 0.01	0.103	1.36 ± 2.07
	W	1.47 ± 0.01	0.660	1.56 ± 2.07	0.93 ± 0.01	0.536	0.24 ± 1.69	0.89 ± 0.01	0.023*	1.10 ± 1.16
SR35-45 m	M	2.20 ± 0.01	0.003*	2.89 ± 2.01	1.22 ± 0.01	0.048*	1.16 ± 2.17	1.34 ± 0.01	0.004*	1.71 ± 2.11
	W	1.04 ± 0.01	0.022*	1.25 ± 1.31	1.19 ± 0.01	0.219	0.60 ± 2.21	1.00 ± 0.01	0.887	0.16 ± 2.20
SR Final	M	2.62 ± 0.01	0.389	0.52 ± 4.39	2.20 ± 0.02	0.799	−0.35 ± 4.66	3.19 ± 0.02	0.084	2.83 ± 5.01
	W	1.88 ± 0.02	0.767	−0.01 ± 4.24	2.58 ± 0.02	0.808	−0.40 ± 4.81	1.30 ± 0.01	0.967	0.25 ± 3.18
SL15-25 m	M	1.53 ± 0.01	0.509	−0.71 ± 2.54	1.06 ± 0.01	0.015*	−1.10 ± 1.67	1.16 ± 0.01	0.735	−0.57 ± 2.66
	W	1.43 ± 0.01	0.481	−1.02 ± 2.54	0.91 ± 0.01	0.852	0.06 ± 1.66	1.08 ± 0.01	0.051	−1.22 ± 1.43
SL35-45 m	M	1.91 ± 0.01	0.127	−1.76 ± 3.03	1.55 ± 0.01	0.081	−1.26 ± 2.39	1.14 ± 0.01	0.278	−1.07 ± 2.37
	W	0.38 ± 0.01	0.826	0.01 ± 0.71	1.02 ± 0.01	0.127	−0.70 ± 1.72	1.19 ± 0.01	0.436	0.42 ± 2.22
SL Final	M	3.85 ± 0.02	0.118	−2.77 ± 6.52	2.85 ± 0.02	0.168	−1.84 ± 5.19	3.57 ± 0.02	0.059	−3.71 ± 4.96
	W	2.79 ± 0.02	0.535	−1.68 ± 4.83	3.09 ± 0.02	0.393	−1.29 ± 5.39	0.97 ± 0.01	0.563	−0.49 ± 1.80

*Significant differences.

**TABLE 7:** Breaststroke intra-athlete’s coefficient of variation (CV) and relative change in performance (%∆). Men (M); Women (W); Heat (H); Semi-final (SF); Final (F) (LEN European Senior Championships 2021).

		H-F (*n* = 8)			H-SF (*n* = 16)			SF-F (*n* = 8)		
		CV	p	**%∆**	CV	p	**%∆**	CV	p	**%∆**
Reaction Time	M	0.04 ± 0.01	0.959	0.01 ± 0.01	0.04 ± 0.01	0.069	0.03 ± 0.01	0.04 ± 0.01	0.348	−0.02 ± 0.01
	W	0.05 ± 0.01	0.124	−0.05 ± 0.01	0.05 ± 0.01	0.170	−0.03 ± 0.01	0.03 ± 0.01	0.401	0.01 ± 0.01
Flight Time	M	0.03 ± 0.01	0.094	−0.03 ± 0.01	0.04 ± 0.01	0.159	−0.03 ± 0.01	0.02 ± 0.01	0.402	-0.02 ± 0.01
	W	0.05 ± 0.01	0.765	−0.01 ± 0.01	0.03 ± 0.01	0.456	−0.02 ± 0.01	0.03 ± 0.01	0.724	−0.02 ± 0.01
Entry Distance	M	0.13 ± 0.01	0.517	0.08 ± 0.01	0.34 ± 0.01	0.509	−0.13 ± 0.01	0.40 ± 0.01	0.458	0.24 ± 0.01
	W	0.30 ± 0.01	0.635	−0.10 ± 0.01	0.24 ± 0.01	0.058	−0.26 ± 0.01	0.21 ± 0.01	0.399	0.03 ± 0.01
Underwater Time	M	0.68 ± 0.01	0.047*	−0.73 ± 0.01	0.65 ± 0.01	0.189	−0.45 ± 0.01	0.69 ± 0.01	0.121	−0.73 ± 0.01
	W	0.33 ± 0.01	0.256	−0.15 ± 0.01	0.40 ± 0.01	0.108	−0.26 ± 0.01	0.30 ± 0.01	0.516	−0.10 ± 0.01
Underwater Distance	M	1.14 ± 0.01	0.016*	−1.68 ± 0.01	0.83 ± 0.01	0.316	−0.46 ± 0.01	0.85 ± 0.01	0.040*	−1.29 ± 0.01
	W	0.29 ± 0.01	0.570	0.17 ± 0.01	0.57 ± 0.01	0.733	0.06 ± 0.01	0.43 ± 0.01	0.829	0.01 ± 4.35
Underwater Velocity	M	3.90 ± 0.05	0.536	−4.37 ± 9.99	3.59 ± 0.04	0.946	0.06 ± 8.60	3.51 ± 0.03	0.385	−2.36 ± 6.73
	W	2.28 ± 0.01	0.092	1.83 ± 3.27	1.81 ± 0.01	0.007*	2.07 ± 2.85	1.53 ± 0.01	0.468	0.53 ± 2.61
Time 15 m	M	0.42 ± 0.01	0.692	−0.08 ± 0.01	0.24 ± 0.01	0.420	0.09 ± 0.01	0.34 ± 0.01	0.699	−0.02 ± 0.01
	W	0.37 ± 0.01	0.116	−0.31 ± 0.01	0.25 ± 0.01	0.525	−0.08 ± 0.01	0.25 ± 0.01	0.307	−0.12 ± 0.01
Time 25 m	M	0.49 ± 0.01	0.151	−0.24 ± 0.01	0.36 ± 0.01	0.016*	−0.33 ± 0.01	0.30 ± 0.01	0.875	0.07 ± 0.01
	W	0.35 ± 0.01	0.050*	−0.44 ± 0.01	0.27 ± 0.01	0.035*	−0.25 ± 0.01	0.23 ± 0.01	0.018*	−0.25 ± 0.01
Time 35 m	M	0.54 ± 0.03	0.178	−0.23 ± 0.01	0.38 ± 0.03	0.013*	−0.39 ± 0.01	0.41 ± 0.05	0.941	0.19 ± 0.01
	W	0.46 ± 0.01	0.007*	−0.60 ± 0.01	0.30 ± 0.02	0.597	−0.07 ± 0.01	0.41 ± 0.01	0.007*	−0.53 ± 0.01
Time 45 m	M	0.51 ± 0.03	0.860	0.08 ± 0.01	0.38 ± 0.03	0.165	−0.22 ± 0.01	0.44 ± 0.06	0.774	0.26 ± 0.01
	W	0.45 ± 0.01	0.014*	−0.57 ± 0.01	0.29 ± 0.03	0.195	−0.18 ± 0.01	0.27 ± 0.01	0.577	−0.05 ± 0.01
Time 50 m	M	0.54 ± 0.01	0.792	0.22 ± 0.86	0.37 ± 0.01	0.790	−0.04 ± 0.63	0.34 ± 0.01	0.324	0.30 ± 0.65
	W	0.55 ± 0.01	0.007*	−0.61 ± 0.57	0.38 ± 0.01	0.761	−0.05 ± 0.62	0.24 ± 0.01	0.012*	−0.30 ± 0.28
Split25-50 m	M	0.36 ± 0.01	0.064	0.45 ± 0.01	0.26 ± 0.01	0.005*	0.28 ± 0.01	0.32 ± 0.01	0.298	0.23 ± 0.01
	W	0.21 ± 0.01	0.106	−0.17 ± 0.01	0.30 ± 0.01	0.163	0.20 ± 0.01	0.11 ± 0.01	0.381	−0.05 ± 0.01
Finish time	M	0.18 ± 0.01	0.138	0.13 ± 0.01	0.30 ± 0.01	0.121	0.17 ± 0.01	0.25 ± 0.01	0.960	0.02 ± 0.01
	W	0.23 ± 0.01	0.536	−0.05 ± 0.01	0.24 ± 0.01	0.192	0.12 ± 0.01	0.31 ± 0.01	0.178	−0.25 ± 0.01
SR15-25 m	M	2.08 ± 0.01	0.031*	2.16 ± 2.77	1.60 ± 0.01	0.008*	1.58 ± 2.14	1.94 ± 0.01	0.065*	1.63 ± 2.68
	W	2.01 ± 0.01	0.318	0.88 ± 3.41	1.63 ± 0.01	0.985	0.01 ± 3.11	1.48 ± 0.01	0.016*	2.04 ± 2.10
SR35-45 m	M	2.48 ± 0.01	0.020*	2.63 ± 2.90	1.31 ± 0.01	0.382*	0.53 ± 2.18	1.14 ± 0.01	0.001*	1.58 ± 1.01
	W	1.75 ± 0.01	0.106	1.54 ± 2.85	1.48 ± 0.01	0.391	0.61 ± -2.66	1.12 ± 0.01	0.021*	1.39 ± 1.50
SR Final	M	4.89 ± 0.03	0.064	3.12 ± 7.82	2.86 ± 0.01	0.012*	2.59 ± 3.71	2.93 ± 0.02	0.179	1.57 ± 5.01
	W	1.50 ± 0.01	0.517	0.37 ± 3.26	2.72 ± 0.04	0.363	1.52 ± 6.43	2.36 ± 0.02	0.505	0.24 ± 5.38
SL15-25 m	M	1.95 ± 0.01	0.212	−1.53 ± 3.49	1.96 ± 0.01	0.790	0.19 ± 3.31	3.04 ± 0.01	0.143	−2.17 ± 4.19
	W	1.84 ± 0.01	0.681	−0.30 ± 3.44	2.30 ± 0.01	0.456	0.72 ± 3.84	1.98 ± 0.01	0.208	−1.54 ± 3.50
SL35-45 m	M	2.26 ± 0.01	0.017*	−2.82 ± 3.02	1.63 ± 0.01	0.679	−0.27 ± 2.75	1.64 ± 0.01	0.008*	−2.19 ± 1.91
	W	1.54 ± 0.01	0.338	−0.92 ± 3.39	2.09 ± 0.01	0.074	−1.54 ± 3.32	1.45 ± 0.01	0.897	−0.13 ± 2.33
SL Final	M	5.00 ± 0.02	0.030*	−4.93 ± 7.09	3.82 ± 0.02	0.003*	−4.51 ± 5.33	4.79 ± 0.02	0.344	−2.26 ± 7.62
	W	3.48 ± 0.02	0.678	−0.17 ± 5.79	4.17 ± 0.04	0.184	−3.32 ± 9.98	3.87 ± 0.02	0.618	1.57 ± 6.70

*Significant differences.

**TABLE 8:** Butterfly intra-athlete’s coefficient of variation (CV) and relative change in performance (%∆). Men (M); Women (W); Heat (H); Semi-final (SF); Final (F) (LEN European Senior Championships 2021).

		H-F (*n* = 8)			H-SF (*n* = 17)			SF-F (*n* = 8)		
		CV	p	**%∆**	CV	p	**%∆**	CV	p	**%∆**
Reaction Time	M	0.06 ± 0.01	0.276	0.05 ± 0.01	0.06 ± 0.01	0.744	0.01 ± 0.01	0.07 ± 0.01	0.565	0.03 ± 0.01
	W	0.04 ± 0.01	0.196	0.01 ± 0.01	0.04 ± 0.01	0.739	0.01 ± 0.01	0.05 ± 0.01	0.997	−0.02 ± 0.01
Flight Time	M	0.11 ± 0.01	0.327	−0.15 ± 0.01	0.22 ± 0.01	0.298	−0.05 ± 0.01	0.12 ± 0.01	0.804	−0.12 ± 0.01
	W	0.07 ± 0.01	0.381	0.01 ± 0.01	0.07 ± 0.01	0.936	−0.01 ± 0.01	0.04 ± 0.01	0.243	−0.04 ± 0.01
Entry Distance	M	0.11 ± 0.01	0.436	−0.05 ± 0.01	0.13 ± 0.01	0.317	−0.07 ± 0.01	0.14 ± 0.01	0.971	0.01 ± 0.01
	W	0.13 ± 0.01	0.621	0.01 ± 0.01	0.16 ± 0.01	0.386	0.04 ± 0.01	0.09 ± 0.01	0.728	−0.08 ± 0.01
Underwater Time	M	0.71 ± 0.01	0.571	−0.27 ± 0.01	0.31 ± 0.01	0.406	0.11 ± 0.01	0.55 ± 0.01	0.353	−0.31 ± 0.01
	W	0.40 ± 0.01	0.132	−0.34 ± 0.01	0.73 ± 0.01	0.525	−0.19 ± 0.01	0.62 ± 0.01	0.928	−0.05 ± 0.01
Underwater Distance	M	0.63 ± 0.01	0.386	−0.32 ± 0.01	0.44 ± 0.01	0.470	0.18 ± 0.01	0.42 ± 0.01	0.014*	−0.61 ± 0.03
	W	0.58 ± 0.01	0.844	−0.24 ± 0.01	0.73 ± 0.01	0.240	−0.29 ± 0.01	0.65 ± 0.01	0.789	−0.29 ± 0.01
Underwater Velocity	M	2.30 ± 0.03	0.731	−0.14 ± 5.74	1.43 ± 0.01	0.747	0.21 ± 3.33	3.51 ± 0.03	0.529	−1.57 ± 7.23
	W	0.92 ± 0.01	0.129	0.31 ± 1.93	2.02 ± 0.02	0.530	−0.20 ± 5.14	0.53 ± 0.06	0.317	0.01 ± 1.01
Time 150 m	M	0.17 ± 0.01	0.037*	−0.21 ± 0.01	0.18 ± 0.01	0.486	−0.07 ± 0.01	0.16 ± 0.01	0.976	0.01 ± 0.01
	W	0.23 ± 0.01	0.054	−0.26 ± 0.01	0.20 ± 0.01	0.104	−0.13 ± 0.01	0.23 ± 0.01	0.253	−0.12 ± 0.01
Time 25 m	M	0.31 ± 0.05	0.011*	−0.39 ± 0.01	0.19 ± 0.02	0.079	−0.15 ± 0.01	0.22 ± 0.03	0.452	−0.06 ± 0.01
	W	0.42 ± 0.07	0.007*	−0.58 ± 0.01	0.38 ± 0.06	0.001*	−0.48 ± 0.01	0.26 ± 0.04	0.255	−0.10 ± 0.01
Time 35 m	M	0.44 ± 0.01	0.001*	−0.61 ± 0.01	0.32 ± 0.03	0.035	−0.29 ± 0.01	0.36 ± 0.01	0.376	−0.04 ± 0.01
	W	0.64 ± 0.09	0.003*	−0.92 ± 0.01	0.43 ± 0.01	0.001*	−0.51 ± 0.01	0.41 ± 0.06	0.063	−0.35 ± 0.01
Time 45 m	M	0.75 ± 0.09	0.003*	−1.05 ± 0.01	0.48 ± 0.01	0.012*	−0.47 ± 0.01	0.43 ± 0.06	0.059	−0.40 ± 0.01
	W	0.75 ± 0.09	0.003*	−1.05 ± 0.01	0.48 ± 0.01	0.012*	−0.47 ± 0.01	0.43 ± 0.06	0.059	−0.40 ± 0.01
Time 50 m	M	0.64 ± 0.01	0.002*	−0.72 ± 0.82	0.41 ± 0.01	0.012*	−0.44 ± 0.64	0.45 ± 0.01	0.421	0.02 ± 0.78
	W	0.88 ± 0.01	0.004*	−1.09 ± 1.22	0.48 ± 0.01	0.023*	−0.44 ± 0.75	0.41 ± 0.01	0.043*	−0.42 ± 0.01
Split25-50 m	M	0.37 ± 0.02	0.049*	−0.34 ± 0.01	0.33 ± 0.03	0.022*	−0.30 ± 0.01	0.25 ± 0.02	0.928	0.08 ± 0.01
	W	0.51 ± 0.04	0.001*	−0.52 ± 0.01	0.30 ± 0.03	0.823	0.02 ± 0.01	0.28 ± 0.02	0.014*	−0.32 ± 0.01
Finish time	M	0.22 ± 0.05	0.798	−0.06 ± 0.01	0.22 ± 0.05	0.619	−0.06 ± 0.01	0.11 ± 0.03	0.644	0.04 ± 0.01
	W	0.28 ± 0.05	0.216	−0.05 ± 0.01	0.15 ± 0.05	0.721	0.02 ± 0.01	0.21 ± 0.06	0.260	−0.03 ± 0.01
SR15-25 m	M	1.54 ± 0.01	0.021*	1.79 ± 2.09	2.15 ± 0.01	0.264	0.94 ± 3.74	2.18 ± 0.02	0.227	1.36 ± 3.99
	W	0.81 ± 0.01	0.003*	1.13 ± 0.81	1.20 ± 0.01	0.001*	1.49 ± 1.27	0.68 ± 0.01	0.960	0.08 ± 1.31
SR35-45 m	M	2.18 ± 0.01	0.015*	2.67 ± 2.83	2.06 ± 0.01	0.005*	2.03 ± 2.71	1.35 ± 0.01	0.828	−0.04 ± 2.63
	W	2.09 ± 0.01	0.001*	2.90 ± 0.95	1.19 ± 0.01	0.035*	0.99 ± 1.78	1.08 ± 0.01	0.059	1.16 ± 1.48
SR Final	M	3.71 ± 0.01	0.545	−0.09 ± 6.28	2.99 ± 0.02	0.246	1.35 ± 5.14	3.22 ± 0.01	0.338	−2.37 ± 4.66
	W	1.96 ± 0.01	0.711	0.18 ± 3.94	2.19 ± 0.02	0.598	−0.55 ± 4.23	1.79 ± 0.01	0.060	1.94 ± 2.72
SL15-25 m	M	1.64 ± 0.01	0.199	−1.00 ± 2.51	2.14 ± 0.01	0.531	−0.72 ± 4.02	2.55 ± 0.02	0.395	−1.23 ± 4.78
	W	0.89 ± 0.01	0.416	0.36 ± 1.58	1.13 ± 0.01	0.892	0.04 ± 1.99	0.60 ± 0.01	0.894	−0.14 ± 1.33
SL35-45 m	M	1.56 ± 0.01	0.041*	−1.79 ± 2.31	1.59 ± 0.01	0.027*	−1.49 ± 2.48	1.64 ± 0.01	0.768	−0.13 ± 2.95
	W	1.42 ± 0.01	0.049*	−1.46 ± 1.69	1.21 ± 0.01	0.115	−0.87 ± 2.21	1.26 ± 0.01	0.882	−0.07 ± 1.99
SL Final	M	3.99 ± 0.02	0.751	0.17 ± 6.75	2.96 ± 0.02	0.473	−1.19 ± 5.58	3.30 ± 0.01	0.379	1.79 ± 4.90
	W	2.54 ± 0.02	0.824	0.01 ± 5.09	2.71 ± 0.02	0.880	0.01 ± 5.14	1.88 ± 0.01	0.115	−1.86 ± 2.56

*Significant differences.

Correlation analyses between the different variables studied and T15, T25 and T50 on each sex group, stroke and differentiating the rounds are presented in Tables 9 to 12. In most events the correlation between T15 and T25, and between T25 and T50 was very large, however, the correlation between T15 and T50 was moderate or only large for the finalists. So, the improvements in the start and underwater segments of the race abovementioned did not have a strong impact on the final race time (i.e., T50). SR15-25 m and SR35-45 m increased over the competitions (freestyle and butterfly in both sexes, breaststroke and backstroke in men). Meanwhile, the SL was prone to decrease most of the times, trading off with the faster SR.

**TABLE 9:** Pearson correlation coefficients (r) of the 50 m Freestyle’s competition variables (LEN European Senior Championships 2021).

		Males			Females		
Variable	Round	Time 15 m	Time 25 m	Time 50 m	Time 15 m	Time 25 m	Time 50 m
Reaction Time	Heats	0.254	0.370	−0.089	0.018	−0.111	−0.223
	Semi-final	0.278	0.292	0.018	0.164	0.044	−0.064
	Final	0.517	0.617	0.611	−0.107	−0.266	0.079
Flight Time	Heats	−0.163	−0.331	−0.445	0.060	0.030	0.117
	Semi-final	−0.203	−0.206	−0.040	−0.128	−0.191	−0.165
	Final	−0.471	−0.410	−0.327	0.567	0.698	0.499
Entry Distance	Heats	−0.374	−0.678*	−0.572**	0.258	0.228	0.212
	Semi-final	−0.506**	−0.508**	−0.224	0.220	0.235	0.166
	Final	−0.699	−0.368	−0.416	0.156	0.272	0.296
Underwater Time	Heats	−0.280	−0.162	−0.283	−0.276	−0.257	−0.189
	Semi-final	−0.365	−0.064	0.203	−0.275	−0.136	−0.061
	Final	0.152	0.375	0.292	0.138	0.353	0.214
Underwater Distance	Heats	−0.366	−0.212	0.211	−0.344	−0.275	−0.189
	Semi-final	−0.358	−0.067	0.194	−0.435	−0.324	−0.247
	Final	0.045	0.306	0.259	−0.017	0.121	−0.033
Underwater Speed	Heats	−0.056	−0.017	−0.417	−0.083	0.043	0.107
	Semi-final	0.245	0.032	−0.181	−0.357	−0.433	−0.459
	Final	−0.632	−0.579	−0.483	−0.379	−0.694	−0.610
Time 15 m	Heats	-	0.785*	0.388	-	0.852*	0.570**
	Semi-final	-	0.747*	0.400	-	0.871*	0.588**
	Final	-	0.727**	0.700	-	0.846*	0.595
Time 25 m	Heats	-	-	0.646*	-	-	0.834*
	Semi-final	-	-	0.781*	-	-	0.884*
	Final	-	-	0.947*	-	-	0.859*
Split 25-50 m	Heats	-	-	0.623*	-	-	0.885*
	Semi-final	-	-	0.802*	-	-	0.945*
	Final	-	-	0.772**	-	-	0.818**
Finish time	Heats	-	-	0.132	-	-	0.434
	Semi-final	-	-	0.068	-	-	0.084
	Final	-	-	0.095	-	-	−0.088
SR 15-25 m	Heats	-	−0.296	−0.201	-	−0.191	0.035
	Semi-final	-	−0.280	−0.143	-	−0.046	−0.059
	Final	-	−0.682	−0.763**	-	−0.130	−0.448
SR 35-45 m	Heats	-	-	−0.084	-	-	0.079
	Semi-final	-	-	−0.078	-	-	0.093
	Final	-	-	0.502	-	-	−0.813**
SR Final	Heats	-	-	0.031	-	-	−0.024
	Semi-final	-	-	0.246	-	-	0.140
	Final	-	-	−0.328	-	-	−0.908*
SL 15-25 m	Heats	-	0.173	0.069	-	0.118	−0.160
	Semi-final	-	0.069	0.112	-	−0.058	0.137
	Final	-	0.121	0.265	-	0.139	0.406
SL 35-45 m	Heats	-	-	−0.135	-	-	−0.159
	Semi-final	-	-	−0.078	-	-	−0.320
	Final	-	-	0.435	-	-	0.828**
SL Final	Heats	-	-	−0.235	-	-	−0.097
	Semi-final	-	-	−0.006	-	-	−0.269
	Final	-	-	0.219	-	-	0.784**

* *p* < 0.01.

** *p* < 0.05.

**TABLE 10:** Pearson correlation coefficients (r) of the 50 m Backstroke’s competition variables (LEN European Senior Championships 2021).

		Males			Females			
Variable	Round	Time 15 m	Time 25 m	Time 50 m		Time 15 m	Time 25 m	Time 50 m
Reaction Time	Heats	0.027	−0.184	−0.522*	0.489		0.533*	0.157
	Semi-final	0.194	−0.005	−0.236	0.550*		0.515*	0.301
	Final	−0.264	−0.703	−0.697	0.699		0.784	0.443
Flight Time	Heats	0.155	0.327	0.156	−0.141		−0.147	0.110
	Semi-final	−0.093	0.046	0.013	0.021		0.048	0.032
	Final	0.033	0.504	0.887**	−0.93		−0.24	0.653
Entry Distance	Heats	0.247	0.178	−0.441	0.147		0.052	−0.171
	Semi-final	−0.203	−0.246	−0.356	0.206		−0.173	−0.174
	Final	0.158	0.484	0.712*	−0.386		−0.307	0.348
Underwater Time	Heats	−0.259	0.037	0.340	−0.098		−0.105	0.044
	Semi-final	−0.310	−0.076	0.447	0.235		0.133	0.098
	Final	0.027	0.282	0.494	0.290		0.279	0.190
Underwater Distance	Heats	−0.565	−0.246	0.459	−0.597*		0.554	−0.161
	Semi-final	−0.521*	−0.302	0.269	−0.441		−0.482	−0.310
	Final	−0.495	−0.196	0.367	−0.266		−0.245	0.121
Underwater Speed	Heats	−0.749**	−0.670**	0.310	−0.946**		−0.865**	−0.400
	Semi-final	−0.555*	-0.483	−0.150	−0.962**		−0.876**	−0.591*
	Final	−0.938**	−0.850**	−0.220	−0.922**		−0.873**	−0.175
Time 15 m	Heats	-	0.805**	−0.173	-		0.919**	0.443
	Semi-final	-	0.886**	0.206	-		0.946**	0.667**
	Final	-	0.731*	−0.041	-		0.984**	0.352
Time 25 m	Heats	-	-	0.308*	-		-	0.679**
	Semi-final	-	-	0.565*	-		-	0.820**
	Final	-	-	0.603	-		-	0.483
Split 25-50 m	Heats	-	-	0.892**	-		-	0.778**
	Semi-final	-	-	0.852**	-		-	0.828**
	Final	-	-	0.941**	-		-	0.639
Finish time	Heats	-	-	0.671**	-		-	0.478
	Semi-final	-	-	0.564*	-		-	0.554*
	Final	-	-	0.766*	-		-	−0.132
SR 15-25 m	Heats	-	−0.230	−0.090	-		−0.151	0.119
	Semi-final	-	−0.028	0.012	-		−0.288	−0.103
	Final	-	−0.595	−0.321	-		−0.506	−0.510
SR 35-45 m	Heats	-	-	−0.059	-		-	0.247
	Semi-final	-	-	−0.135	-		-	−0.294
	Final	-	-	−0.358	-		-	−0.583
SR Final	Heats	-	-	−0.216	-		-	−0.321
	Semi-final	-	-	−0.270	-		-	0.184
	Final	-	-	−0.382	-		-	−0.812*
SL 15-25 m	Heats	-	0.119	−0.167	-		−0.129	−0.235
	Semi-final	-	−0.170	−0.354	-		0.122	−0.110
	Final	-	0.474	0.025	-		0.072	0.403
SL 35-45 m	Heats	-	-	−0.237	-		-	−0.131
	Semi-final	-	-	−0.305	-		-	0.020
	Final	-	-	0.089	-		-	0.416
SL Final	Heats	-	-	−0.100	-		-	0.193
	Semi-final	-	-	−0.065	-		-	0.145
	Final	-	-	−0.151	-		-	0.667

* *p* < 0.01.

** *p* < 0.05.

**TABLE 11:** Pearson correlation coefficients (r) of the 50 m Breaststroke’s competition variables (LEN European Senior Championships 2021).

		Males			Females		
Variable	Round	Time 15 m	Time 25 m	Time 50 m	Time 15 m	Time 25 m	Time 50 m
Reaction Time	Heats	0.323	0.357	0.058	−0.125	−0.073	−0.034
	Semi-final	0.400	0.511*	0.244	0.377	0.232	0.267
	Final	0.445	0.662	0.592	−0.023	−0.021	−0.071
Flight Time	Heats	−0.193	−0.099	0.082	0.347	−0.042	−0.319
	Semi-final	−0.139	−0.138	0.046	0.047	−0.463	−0.451
	Final	−0.260	−0.380	−0.247	0.030	−0.223	−0.285
Entry Distance	Heats	−0.160	−0.232	−0.040	0.069	−0.074	−0.226
	Semi-final	−0.033	−0.141	−0.258	−0.087	−0.444	−0.418
	Final	−0.058	−0.159	−0.266	−0.620	−0.202	0.097
Underwater Time	Heats	−0.666**	−0.319	0.015	−0.264	0.484	0.303
	Semi-final	−0.220	−0.337	−0.090	0.268	0.499*	0.248
	Final	−0.044	0.004	0.034	0.429	0.449	0.184
Underwater Distance	Heats	−0.782**	−0.430	−0.109	−0.022	0.300	0.286
	Semi-final	−0.475	−0.553	−0.168	0.011	0.354	0.256
	Final	−0.482	−0.465	−0.211	0.341	0.246	0.007
Underwater Speed	Heats	−0.001	−0.036	−0.111	−490	−0.438	−0.129
	Semi-final	−0.542*	−0.420	−0.154	−607*	−0.480	−0.082
	Final	−0.820*	−0.746*	−0.394	−0.562	−0.696	−0.491
Time 15 m	Heats	-	0.731**	0.230	-	0.688**	0.354
	Semi-final	-	0.838**	0.314	-	0.583*	0.337
	Final	-	0.942*	0.577	-	0.440	−0.097
Time 25 m	Heats	-	-	0.758**	-	-	0.755**
	Semi-final	-	-	0.681**	-	-	0.820**
	Final	-	-	0.763*	-	-	0.833*
Split 25-50m	Heats	-	-	0.870**	-	-	0.925**
	Semi-final	-	-	0.838**	-	-	0.943**
	Final	-	-	0.730*	-	-	0.945**
Finish time	Heats	-	-	0.122	-	-	0.287
	Semi-final	-	-	0.416	-	-	0.084
	Final	-	-	0.068	-	-	−0.067
SR 15-25 m	Heats	-	−0.144	−0.404	-	−0.321	−0.429
	Semi-final	-	−0.139	−0.279	-	−0.690**	−0.465
	Final	-	−0.138	−0.108	-	−0.167	−0.230
SR 35-45 m	Heats	-	-	−0.308	-	-	0.416
	Semi-final	-	-	−0.388	-	-	−0.539*
	Final	-	-	0.089	-	-	−0.309
SR Final	Heats	-	-	−0.328	-	-	−0.515*
	Semi-final	-	-	−0.246	-	-	−0.505*
	Final	-	-	−0.115	-	-	−0.240*
SL 15-25 m	Heats	-	0.037	0.163	-	0.209	0.256
	Semi-final	-	0.008	0.049	-	0.635**	0.379
	Final	-	−0.280	−0.069	-	0.001	−0.077
SL 35-45 m	Heats	-	-	0.061	-	-	0.227
	Semi-final	-	-	0.164	-	-	0.308
	Final	-	-	−0.277	-	-	0.091
SL Final	Heats	-	-	0.271	-	-	0.388
	Semi-final	-	-	0.008	-	-	0.394
	Final	-	-	0.096	-	-	0.240

* *p* < 0.01.

** *p* < 0.05.

**TABLE 12:** Pearson correlation coefficients (r) of the 50 m Butterfly’s competition variables (LEN European Senior Championships 2021).

		Males			Females			
Variable	Round	Time 15 m	Time 25 m	Time 50 m	Time 15 m		Time 25 m	Time 50 m
Reaction Time	Heats	0.205	0.123	0.157		0.163	0.144	0.064
	Semi-final	0.113	0.118	0.185		0.247	0.334	0.287
	Final	−0.002	0.097	0.143		−0.170	0.660	0.721*
Flight Time	Heats	−0.045	0.086	0.246		0.060	−0.073	0.023
	Semi-final	−0.046	−0.049	0.083		0.004	−0.086	−0.055
	Final	−0.039	−0.100	−0.147		0.229	0.047	0.025
Entry Distance	Heats	−0.287	−0.364	0.011		0.028	0.036	0.115
	Semi-final	−0.635**	−0.556**	0.062		−0.252	−0.235	−0.178
	Final	−0.333	−0.591	−0.503		0.306	0.205	0.140
Underwater Time	Heats	−0.565	−0.401	0.080		−0.427	−0.324	−0.299
	Semi-final	−0.317	−0.129	0.054		−0.232	−0.224	−0.238
	Final	−0.112	0.264	0.443		0.300	0.411	0.491
Underwater Distance	Heats	−0.750**	−0.574*	−0.016		−0.579*	−0.443	−0.412
	Semi-final	−0.427	−0.181	0.061		−0.383	−0.471	−0.434
	Final	−0.395	0.012	0.267		0.177	0.203	0.496
Underwater Speed	Heats	−0.086	0.118	−0.198		0.384	−0.305	−0.291
	Semi-final	−0.201	−0.088	0.032		−0.270	−0.362	−0.319
	Final	−0.717*	−0.749*	−0.604		−0.308	−0.463	−0.314
Time 15 m	Heats	-	0.906**	0.332		-	0.936**	0.844**
	Semi-final	-	0.895**	0.406		-	0.918**	0.826**
	Final	-	0.831*	0.466		-	0.949**	0.885**
Time 25 m	Heats	-	-	0.519*		-	-	0.924**
	Semi-final	-	-	0.622*		-	-	0.928**
	Final	-	-	0.789*		-	-	0.825*
Split 25-50 m	Heats	-	-	0.496		-	-	0.765**
	Semi-final	-	-	0.621*		-	-	0.897**
	Final	-	-	0.601		-	-	0.673
Finish time	Heats	-	-	0.270		-	-	0.414
	Semi-final	-	-	0.235		-	-	0.551
	Final	-	-	0.540		-	-	−0.110
SR 15-25 m	Heats	-	−0.319	−0.215		-	0.398	0.271
	Semi-final	-	−0.425	−0.241		-	0.355	0.335
	Final	-	−0.242	−0.515		-	−0.065	0.058
SR 35-45 m	Heats	-	-	−0.537*		-	-	0.247
	Semi-final	-	-	−0.419		-	-	0.179
	Final	-	-	−0.486		-	-	−0.173
SR Final	Heats	-	-	−0.557*		-	-	−0.194
	Semi-final	-	-	−0.483		-	-	−0.184
	Final	-	-	−0.462		-	-	−0.339
SL 15-25 m	Heats	-	0.347	0.158		-	−0.563*	−0.452
	Semi-final	-	0.426	0.161		-	−0.409	−0.408
	Final	-	0.042	0.228		-	0.072	0.023
SL 35-45 m	Heats	-	-	0.160		-	-	−0.410
	Semi-final	-	-	0.225		-	-	−0.367
	Final	-	-	0.381		-	-	0.049
SL Final	Heats	-	-	−0.087		-	-	−0.052
	Semi-final	-	-	0.273		-	-	−0.198
	Final	-	-	0.213		-	-	0.263

* *p* < 0.01.

** *p* < 0.05.

The regression analysis for each variable and stroke are presented as Supplementary Material. Additionally, the final time achieved by the medallists in the different rounds (i.e., T50) was plotted against the performances achieved by the finalists, semi-finalists and rest of participants and presented as supplementary material (Supplementary Material).

## 4 Discussion

The first aim of this research was to study the coefficient of variation (CV) and the progression of performance (%∆) in the 50 m event among swimmers who participated in different rounds of the same championship. It was hypothesised that if faster swimmers took the heats slower, a change in performance over the rounds would be detected. The results of the performances achieved during the finals compared to the heats showed that the best swimmers did not excel during the heats, as a significant progression of performance was observed in most of the strokes as the competition progressed. However, when comparing the performances in the final with those in the semi-finals, the progressions of performances in some strokes were poorer or not significant, due to the better performances achieved during the semi-final.

With reference to the 50 m freestyle, there were differences in CV between performances obtained in the finals and semi-finals compared to the heats (Table 5). These CV changes entailed a progressive reduction in the T50 as swimmers progressed between rounds. However, the performance achieved by the men during the final was worse compared to the semi-final (Table 1). Possibly, this failure could be the result of ineffective planning, or the swimmers’ inability to perform at their best under the pressure of international competition (Mujika et al., 2019), but also, it is likely that as the level of the contenders was quite even, many of them tried to perform really well in the semi-final to avoid being left out of the final. In breaststroke, only women obtained differences in T50 between performances obtained in the finals compared to the heats (Table 3). In men, although the CV represented changes in performance (Table 7), it appears that some contenders had performance deteriorations during the final, resulting in a mean ∆ = 0.2%. In any case, it is worth mentioning that, although their CV change was not positive for performance, some managed to reach medal positions, which means that this deterioration came from the difference result after having performed extraordinarily well during the heats. For further information on the performance of the medallists in comparison to the other contenders, it is recommended to consult supplementary material (Supplementary Material).

In the 50 m backstroke, the men showed differences in T50 CV between performances obtained in the finals compared to the heats (Table 6), without differences in women. For the men, these changes in CV meant a progressive reduction in T50 as swimmers progressed between rounds; however, the women’s time performances were better in the semi-final than in the rest of the rounds (Table 3). Therefore, the best male swimmers either did not excel during the heats and/or were able to obtain progressions in performance as the competition progressed. In this sense, it is important to mention that apart from the fact that the level of the finalists was quite similar, the world record in this event was broken in the final, so this influenced the results obtained. Finally, in the men’s 50 butterfly there were differences in the CV T50 for both men and women between the performances obtained in the finals and semi-finals compared to the heats (Table 8). These changes in CV meant a progressive reduction in T50 as the swimmers progressed between rounds, with the exception of the performance achieved by the men during the finals, which was the same as that achieved during the semi-finals (Table 4). Therefore, although the men and women did not excel during the heats, possibly the men were not able to achieve further performance progressions as the competition progressed because performance in the semi-finals was already really of high-level.

On the other hand, this study aimed to specifically analyse which of the key moments of the race or its subfactors are most modified to achieve improvement across the rounds. It was hypothesized that these changes would be a consequence of the improvement in the performance variables of the initial segment. This hypothesis was only partially confirmed as for some races the improvement came in the variables collected at the final stages of the race.

### 4.1 Swimming Start Variables (Reaction Time, Flight Time and Distance of Entry)

In sprint swimming, improving the start could make the difference between winning or not get a medal (García-Hermoso et al., 2017; Arellano et al., 2018; Sánchez et al., 2021). Therefore, several investigations have shown that swimmers should optimise the force-time distribution during the impulse phase (de Jesus et al., 2014; Vantorre et al., 2014; Cuenca-Fernández et al., 2015). Despite swimming start speed was not calculated, a good start is understood as an increase in speed since the swimmer leaves the block and reach the water could be achieved by either a combination of a reduction in execution time and an increase in distance of entry or a combination of both (Vantorre et al., 2014). Therefore, a good start cannot simply be explained by a single parameter (Gonjo and Olstad, 2020).

#### 4.1.1 Freestyle

A change in flight time CV with a corresponding ∆% reduction (Table 5) was a common factor in both men and women progressing between heats and the final (Table 1). It appears that swimmers during the final intentionally tried to get to the water fast rather than trying to increase the hand’s entry distance. According to other authors (Kilani and Zeidan, 2004; Simbaña-Escobar et al., 2018; Morais et al., 2021), the best freestyle swimmers are especially faster in the start sections; however, a shorter flight time obtained a low magnitude on the correlations with any performance variable (i.e., T15, T25 and T50) (Table 9). In addition, during heats and semi-finals, men who achieved a longer entry distance obtained better performance results, while a slight increase was observed in women in semi-finals and finals compared to heats (Table 1). Therefore, it cannot be ruled out that both a reduction in flight time and an increase in entry distance can be modified by the swimmers to influence the speed of the start.

#### 4.1.2 Backstroke

The reaction time did not differ between rounds, although the male finalists showed a significant reduction in flight time together with a non-significant increase in distance compared to previous rounds, which could be translated into an increase in speed (Table 2). On the contrary, in the women, this combination yielded worse results than those obtained in previous rounds. A previous study has shown that men react faster to an auditory stimulus when large muscle groups are involved (Spierer et al., 2010). In this study, the reaction time of men and women was similar, however, this yielded different results. In men, the best performers were those with a slower reaction time, but also those who combined a shorter flight time and a longer entry distance, attaining large to very high correlations (Table 10), which seems to be an indicative of a higher impulse achieved at the start (García-Hermoso et al., 2017). In contrast, the women with a slower reaction time seemed to achieve worse performances at T15 and T25, so for them this did not lead to a higher impulse at the start (Table 10). These differences could be explained by sex, as absolute leg power is higher in men than in women (García-Hermoso et al., 2017; Simbaña-Escobar et al., 2018).

#### 4.1.3 Breaststroke

Changes in start variables were not significant for either men or women (Table 7) and no decreasing or increasing trends were observed between rounds as the competition progressed to the final (Table 3). It is important to mention that the men did not obtain overall performance progressions in the final time (T50). However, apparently the women also did not vary the swim start variables as they progressed between rounds. Therefore, it is possible that the modifications in breaststroke come from other variations occurring in the underwater phase (Olstad et al., 2020; Sánchez et al., 2021).

#### 4.1.4 Butterfly

Both men and women produced no variation in performance in any of the start variables to progress between rounds (Table 8). According to a previous study (Kilani and Zeidan, 2004), the swim start was a differentiating factor between finalists and semi-finalists in 50 m butterfly success. However, the large variations in performance were possibly caused by other variables rather than by the actions taken on the block. By the large magnitude of the correlations, those men who achieved a longer entry distance in the semi-finals were the ones who performed better in T15 and T25 (Table 12).

### 4.2 Underwater Variables (Underwater Time, Distance and Speed)

In previous studies, the underwater phase has been divided into two parts: the glide and the undulatory swim, differentiated by the moment at which the movement of the lower limbs begins (de Jesus et al., 2014; Vantorre et al., 2014). However, a limitation of current methods of competition analysis is that the camera setup is limited to the above-water view only, which means that underwater kinematic information cannot be assessed in detail (Gonjo and Olstad, 2021). In any case, the underwater swim during the start and turn segments must be adjusted to maximise average speed (Veiga et al., 2014), which means that good underwater performances cannot simply be explained by a single parameter (e.g., only underwater distance) (Sánchez et al., 2021).

#### 4.2.1 Freestyle

The male finalists showed CV changes in underwater time and distance that represented a significant ∆% reduction (Table 5). However, the underwater speed of the final did not prove to be superior as a result (Table 1). In this regard, a previous study reported that a long underwater distance is not necessarily related to a fast finish time and suggested that some fast swimmers (as seen during this championship) might prioritise breaking the water quickly to maximise average forward speed (Veiga and Roig, 2016). However, those who achieved higher underwater speeds did not obtain correlations with race times (Table 9), questioning the current paradigm on the best approach to take to the underwater phase of the 50 m freestyle. Only the female finalists showed significant changes in their CV in the final compared to the previous rounds that involved increases in underwater speed (Table 1). As with the men, different profiles were observed with swimmers attempting to reduce distance underwater causing a loss of speed, and others gaining an increase in speed as a result of that reduction. Therefore, it seems that swimmers attempted different manners to increase such speed in order to improve final performance (Table 5).

#### 4.2.2 Backstroke

There was a significant CV in the men between the final and semi-final which showed that the finalists reduced the time and distance of the underwater swim during the final to gain speed in the first few metres of the event, although these improvements were only significant when compared to the heats (Table 6). It has been reported that, in backstroke sprint events, swimmers move faster when performing dolphin kicks than swimming on the surface (Collard, 2007). In some cases (i.e., men in the semi-finals and women in the heats), swimmers with higher underwater distances obtained large correlations with T15 (Table 10); however, swimmers with superior underwater speeds were the best performers at T15 and T25 in most rounds. This is consistent with other research where maximising underwater speed was more important than displacing a long distance underwater (Gonjo and Olstad, 2021). In the women, no significant CVs were obtained for any of the underwater variables (Table 6).

#### 4.2.3 Breaststroke

Significant CV changes were obtained for underwater time and distance in the male finalists, indicating that during the final there was a ∆% reduction compared to the heats (Table 7). However, if this reduction was made with the aim of generating an increase in underwater speed, this was not the case (Table 3), with many swimmers demonstrating very different strategies from each other, as can be seen in the high SD obtained in the ∆% for this variable. A previous study carried out in short pool showed that, in men, a long underwater distance was related to a better final time (Sánchez et al., 2021); in this study, the same relationship was only found in T15, and only during the heats. In the case of the women, no significant changes were generated between rounds in any of the underwater time and distance variables; however, an increase on the underwater speed was detected in the semi-final. Actually, it appears that a short underwater time benefited performance at T25 during the semi-finals, but these relations were only moderate and did not translate to T50 (Table 11). Therefore, although a possible influence was plausible, the changes that occurred in T50 likely came from changes in other variables. A similar result was obtained previously (Olstad et al., 2020; Sánchez et al., 2021) since no correlations were obtained between the variables of emersion time with final time, and no differences were obtained between finalists and non-finalists.

#### 4.2.4 Butterfly

Only in men, there was a reduction in underwater distance during the final (Table 8). Interestingly, those men and women who achieved a greater underwater distance achieved better results in T15 and T25, with very high correlations only during the heats, but only the men who reached a greater underwater speed achieved better results in T15 and T25 only during the final. According to Gonjo and Olstad (2020), average forward speed during the underwater phase is highly correlated with T15. In our study, the finalists obtained the same correlation also for T25 (Table 12), so possibly a reduced underwater phase was adopted during the final with the aim of gaining speed, although it was not effective for all swimmers.

### 4.3 Time Segments (Time to 15, 25, 35 and 45 m; Split Time (From 25 to 50 m); Finish Time (45–50 m).

For the start time at 15 m and the finish segment, there is a lack of knowledge in the sprint events in the long course (Gonjo and Olstad, 2021; Morais et al., 2021), even more so if what is studied is how these variables change over the different rounds.

#### 4.3.1 Freestyle

In men, no significant CV was obtained in T15 as the time performances were similar between rounds (Table 1). According to other studies (Trinidad et al., 2020; Morais et al., 2021), in the comparison between faster and slower swimmers in 50 m freestyle, the largest differences are observed in T15. However, while T15 was the main predictor of T25 performance for both men and women, with very high to nearly perfect correlations, this variable did not affect T50 in the case of men (Table 9), possibly due to the different profiles found in the underwater phase, and the fact that some of the swimmers were able to progress even in the face of disadvantageous starts (or vice versa, fade after advantageous starts). The women showed changes in CV in T15, which led to improvements in performance in the semi-finals and final compared to the heats (Table 1).

With the exception of T25, the men did not obtain significant CV changes for the 35 and 45 m mark, as performances during the semi-finals were better than achieved in the finals. For the same variables, the women obtained changes in CV corresponding to ∆% reductions in swim time as the race progressed, especially between the semi-final and final compared to the heats (Table 5). In this case, it appears that improvements in T15 not only influenced final performance, but also that those with excellent performances early in the race were difficult to beat by other contenders in the middle of the 50 m-lap (Simbaña-Escobar et al., 2018). Therefore, in the case of the women, it was much more relevant a good development in the early stages of the race (15 and 25 m) to improve the final time obtained in the previous rounds.

For the Split25-50 m and finish time, there were CV changes and ∆% reductions in the men in the final and semi-finals compared to the heats (Table 5), so it is possible that regardless of the improvements obtained in the first metres of the event, some swimmers had the ability to avoid a sharp decrease in speed at the end (Simbaña-Escobar et al., 2018; Morais et al., 2021). In the case of the women, these variables did not improve as the competition progressed.

#### 4.3.2 Backstroke

The men increased the speed of swimming between rounds since a significant CV change was obtained in all time variables in the comparison of the time of the final and heats and in most of the comparisons between the final and the semi-finals (Table 6). In the case of females, it appeared to be performance improvements during the semi-finals; however, the expected improvements were not obtained during the final (Table 3). The variable T15 obtained a very high correlation T25 performance in most cases but did not predict T50. In the case of T25 this variable appears not to be valid to predict T50 performance during the Final.

On the other hand, both men and women obtained CV changes in the finish time, with better performance during the semi-final than during the heats, so, in terms of swimming strategy, increasing the pace in the first split of the race (15 or 25 m) seemed to be a determining factor to reduce the final time, especially in men, as improvements here translated to final performance and neither the pace of the 25–50 split, nor the finish Time, had an influence on the worsening of these results. That said, lower Split25-50 and finish times obviously benefited better T50 performances (Table 10).

#### 4.3.3 Breaststroke

For both men and women, there were no changes in CV at T15 in the different rounds (Table 7), so the changes made in the previous underwater phase had no relevant effect on performance. Similarly, T15 was shown to predict at 25, but not T50. In other study (Sánchez et al., 2021), male and female 50 m breaststroke finalists had better T15 m values during finals compared to heats (*p* < 0.05), and these values were related to better final performance (r > 0.6); however, the participants in that study were national level swimmers and the relationships might be different among higher level contenders (i.e., international championship finalists and semi-finalists) (Hellard et al., 2008).

For T25, T35, and T45, in the men, CV changes were only significant in the semi-finals, as time performances appeared to be better than those achieved during the final (Table 7), confirming the fact that the winner and/or medallists may not always be the fastest of the tournament (Supplementary Material). On the contrary, women showed CV changes and ∆% reductions to progress between rounds, especially significant between the final and the heats (Table 3). Thus, performance changes in the women occurred mainly during the clean swim splits (T25, T35, and T45).

There were no variations or reductions in performance in the variables Split25-50 m and finish time, meaning that possibly the swimmers acquired high speed in the first stage of the race and found it very difficult to continue progressing in performance as the race proceeds.

#### 4.3.4 Butterfly

Despite no improvement in the underwater phase, T15 m improved in men and almost in women (*p* = 0.054) to progress between rounds. In fact, the CV of T25, T35 and T45 changed in men and women in the finals, and especially in women in most of the semi-finalists (Table 8). These changes showed reductions in ∆% between rounds. It seems that starting the race at high speed to reduce the time to 15 m was more determinant for the women than the men to achieve better performance in T25 and T50. In this regard, Kilani and Zeidan (2004), reported that the first split of the race, including the swim start, was more determinant than the second to achieve a great result. In any case, both men and women who progressed between rounds to the final showed changes in Split25-50 CV, with significant reductions ∆% especially during the semi-finals (Table 4), indicating that they were able to improve performance both at the beginning and at the end of the race.

### 4.4 Stroke Patterns (Stroke Rate and Stroke Length)

Changes in stroke patterns have been interpreted as a strategy used by swimmers to cope with performance changes within a race (Seifert et al., 2005; Hellard et al., 2008). Stroke rate is related to neuromuscular power and energetic capacities (Wakayoshi et al., 1995), whereas stroke length depends more on technical skill resulting from the increased propulsive force generated by the arms and legs (Seifert et al., 2005). The literature, in middle-distance swimming, has reported that high-level swimmers have a higher stroke rate and length than low-level swimmers (Hellard et al., 2008). However, evidence in sprint swimming showed that swimming speed, stroke rate and stroke length are not linearly related (Craig and Pendergast, 1979; Wakayoshi et al., 1995).

#### 4.4.1 Freestyle

Although changes in CV were not always statistically significant, an overall increase in SR15-25 and SR35-45 appeared to be determinant for those men and women who progressed between rounds (Table 5). A high SR helps to maintain a high swim speed between stroke cycles and to overcome drag (Barbosa et al., 2010; Ribeiro et al., 2017; Simbaña-Escobar et al., 2018). Within the race, the values of this variable decreased progressively from 15–25 to 35–45 m, possibly as a consequence of fatigue, as reported previously (Cuenca-Fernández et al., 2021a; Morais et al., 2021). In the case of the male finalists, a significant CV change (higher SR) was observed in the last metres (Table 1), which would be consistent with the CV and ∆% results obtained for Split25-50 m and finish time. For the women, CV changes showed increases in SR in the second half of the event (i.e., from 35 to 45 m), to move into the semi-finals and final (Table 5). In terms of SL, CV changes accounted for ∆% reductions for both men and women between rounds. This was in agreement with Maglischo (2003), who stated that “when swimmers want to go faster, they increase their SR, although their SL decreases”. While the swimmers during the final showed higher SL values from 15 to 25 m compared to the previous rounds (Table 1), in most cases, the values at 35–45 m were higher than at 15–25 m, presumably as a consequence of the decrease of SR. According to some studies (Kilani and Zeidan, 2004; Arellano et al., 2018; Simbaña-Escobar et al., 2018; Morais et al., 2021), SL is one of the main factors responsible for the difference in swim speed in 50 m freestyle. In this sense, a higher SL could reflect a greater ability to transfer the propulsive thrust to the water (Cuenca-Fernández et al., 2020; Ruiz-Navarro et al., 2020). However, men did not obtain any significant correlation, and those female finalists who showed a high SL at the end of the race obtained very high positive correlations with T50, attaining worse performances (Table 9). Therefore, it cannot be ruled out that swimmers who were able to increase their SR while maintaining or decreasing in a non-meaningful way SL, gained advantages in progressing between rounds in the sprint freestyle.

#### 4.4.2 Backstroke

The CV differences showed that increases in SR between rounds were common in men (Table 6), but these changes were not a consistent pattern in all women. As observed for the other strokes, higher SR was accompanied by reductions in SL (Table 2). In any case, higher or lower SR and SL were not a determining factor for those who performed better, and the SR final was only noticeable for the female finalists at the end of the race, possibly because most of them did not significantly increase SR15-25 and SR35-45 as they progressed between rounds. It has been reported that backstroke often leads to lower SR values due to the longer duration of the propulsion and recovery phases (Gonjo et al., 2020). Compared to other strokes, less propulsive drag force is applied by the hands during the push phase due to the wrist moving backwards with respect to the swimming direction. Thus, this would imply that the contribution of the other body parts to propulsion, such as the lower limbs, is much greater and could therefore be much less detectable if progressing between rounds.

#### 4.4.3 Breaststroke

The men maintained similar SR values throughout the race, however, the CV showed the increase in ∆% SR between rounds (Table 7). Similarly, the women obtained significant CV reflecting that they were able to increase SR especially during the final, but only in them, relationships were observed with improved performance. Previous studies have denoted that high SR (above 60 cycles/min) and lower glide is necessary for success in breaststroke sprint swimming (Kilani and Zeidan, 2004; Strzala et al., 2013); however, as swimmers increased SR as they progressed between heats, it resulted in a reduction of the glide phase and thus SL, especially in men (Table 3). Therefore, within the swimmers who were able to progress between rounds, the SR increase could be a relevant factor as showed in the women; however, when the increase in SR induces a severe reduction in SL, a worsening of performance may occur as demonstrated in the men.

#### 4.4.4 Butterfly

Both men and women obtained CV differences with clear trends towards increased SR during the final and semi-finals compared to the heats (Table 4). Sprint butterfly swimmers have been reported to achieve high speed with very high SR, often exceeding 60 cycles per minute, as demonstrated in previous European swimming championships (Strzała et al., 2017). Furthermore, in the study of Seifert et al. (2008), more skilled butterfly swimmers had higher SR and SL than less skilled swimmers. In our study, however, only SR showed certain relationships in men with T50 during the heats, while SL did not seem to predict final performance in any case, with low to moderate correlations (Table 12). Similar to what was obtained for other strokes, the increase in SR was possibly the main cause of the decreases in SL in the second part of the race (SL35-45), as in both men and women CV changes with ∆% performance reductions were obtained in the finals and semi-finals compared to the heats.

## 5 Conclusion

During the different rounds of the 50 m competitions, intra-individual performances varied in a significant range of 0.5–0.7%. With the exception of the men’s breaststroke, there were significant improvements in T50 as the competition progressed, meaning that the best swimmers did not excel during the heats to perform at their best during the final. For all strokes, apart from slight improvements in the actions performed in the block, it was a common tendency to reduce the underwater phase and increase SR with the aim of increasing speed. However, this result was not always obtained or was not adequately transferred to the final performance.

It is important to bear in mind that elite sports are often composed of “outliers” performances coming from athletes with different backgrounds and, therefore, trends will always be somewhat influenced by this. In addition, high achievements are also influenced by post-training factors that increase with years of practice and the level of expertise to know how to move from heats, to semi-finals and finals. Clearly, top swimmers who are able to gather those qualities, will improve their performance in major international competitions and their chances of winning a medal.

## Data Availability

The original contributions presented in the study are included in the article/Supplementary Material, further inquiries can be directed to the corresponding author.
