# Supplementary material for: Are the 50 m Race Segments Changed From Heats to Finals at the 2021 European Swimming Championships?
Source: Front Physiol. 2022 Jul 13;13:797367. doi: 10.3389/fphys.2022.797367 (PMC9326221; doi:10.3389/fphys.2022.797367)
Supplement: Supplementary file 1 [file DataSheet2.pdf]

SUPPLEMENTARY MATERIAL (I)

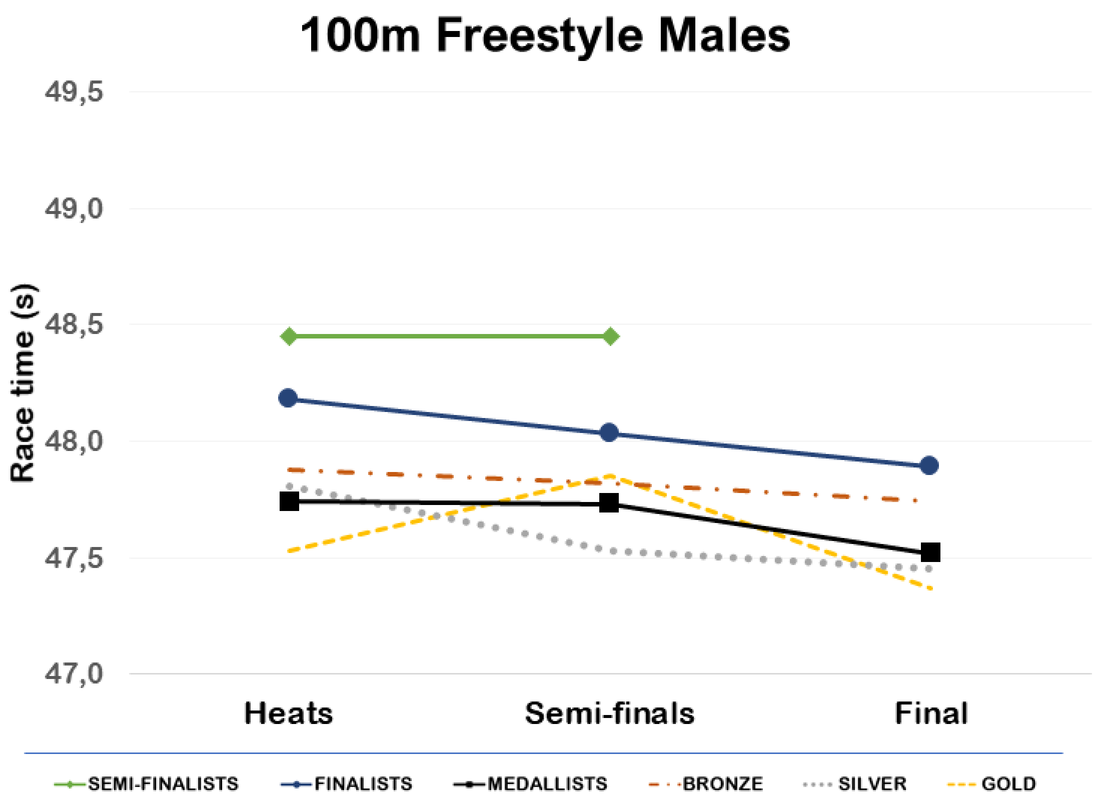

Figure 1. Evolution of the average values of the athletes analyzed in the heats, semi-finals, finals and the individual values of the medalists in the event 100m freestyle male (LEN European Senior Championships 2021).

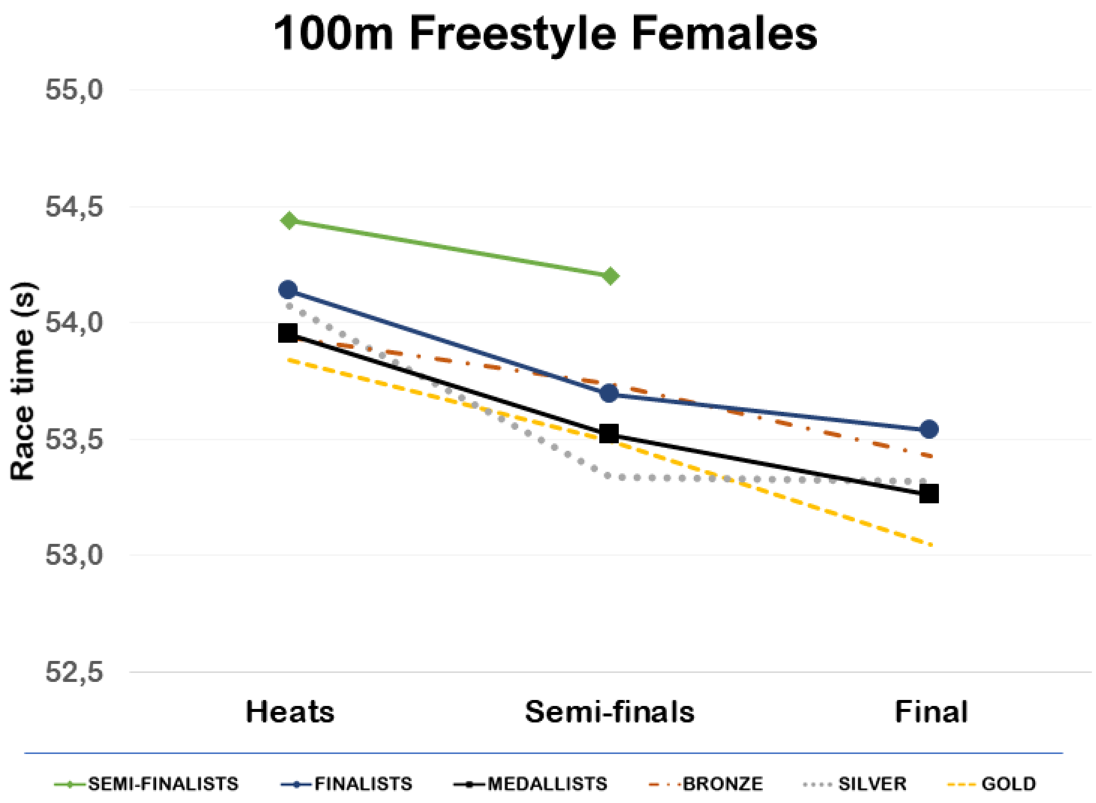

Figure 2. Evolution of the average values of the athletes analyzed in the heats, semi-finals, finals and the individual values of the medalists in the event 100m freestyle female (LEN European Senior Championships 2021).

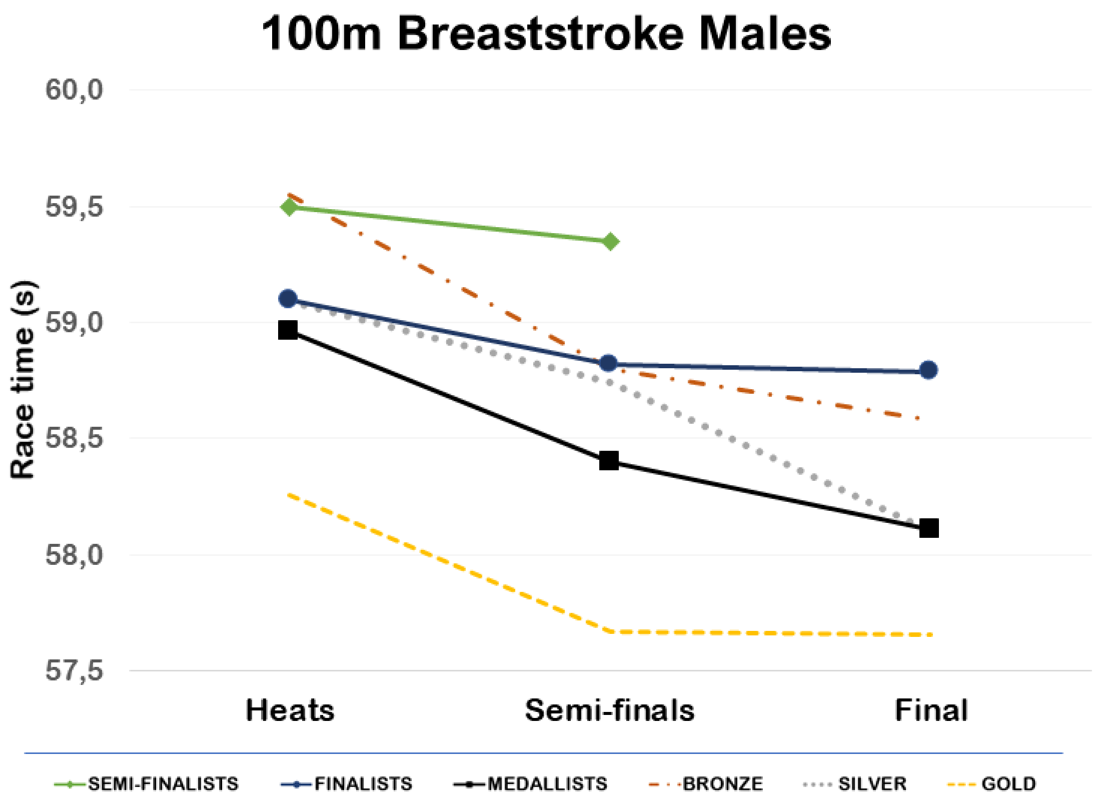

Figure 3. Evolution of the average values of the athletes analyzed in the heats, semi-finals, finals and the individual values of the medalists in the event 100m breaststroke male (LEN European Senior Championships 2021).

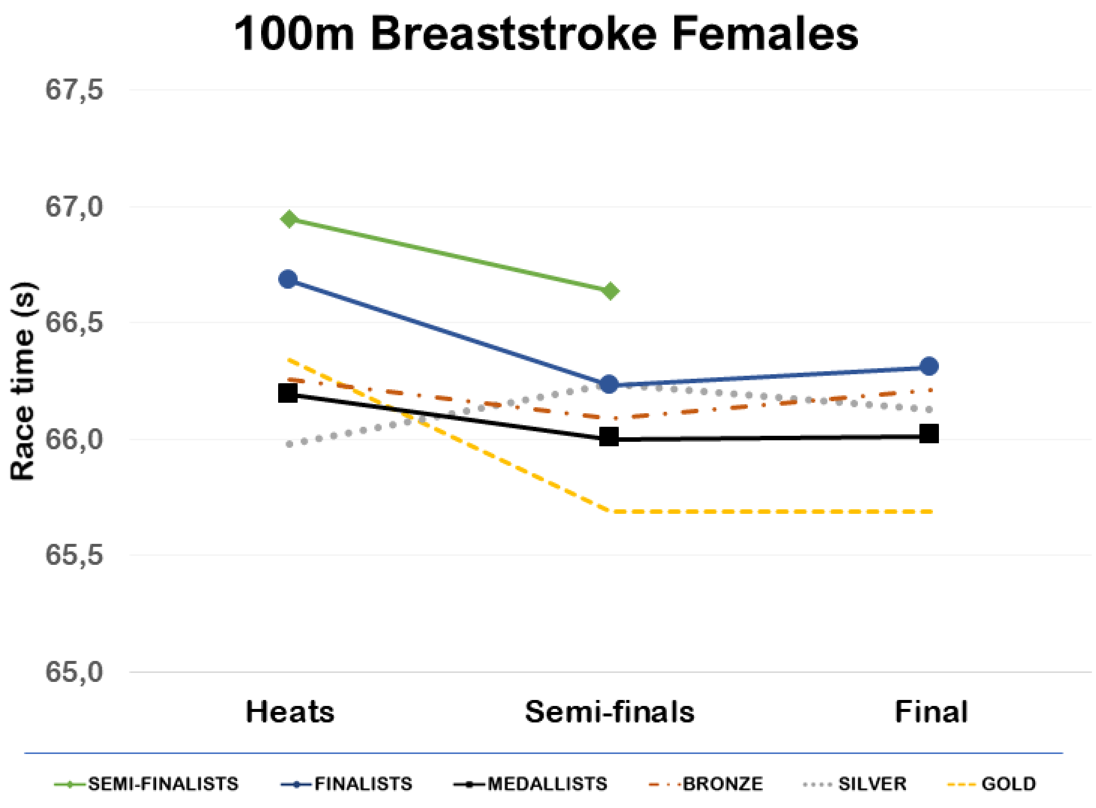

Figure 4. Evolution of the average values of the athletes analyzed in the heats, semi-finals, finals and the individual values of the medalists in the event 100m breaststroke female (LEN European Senior Championships 2021).

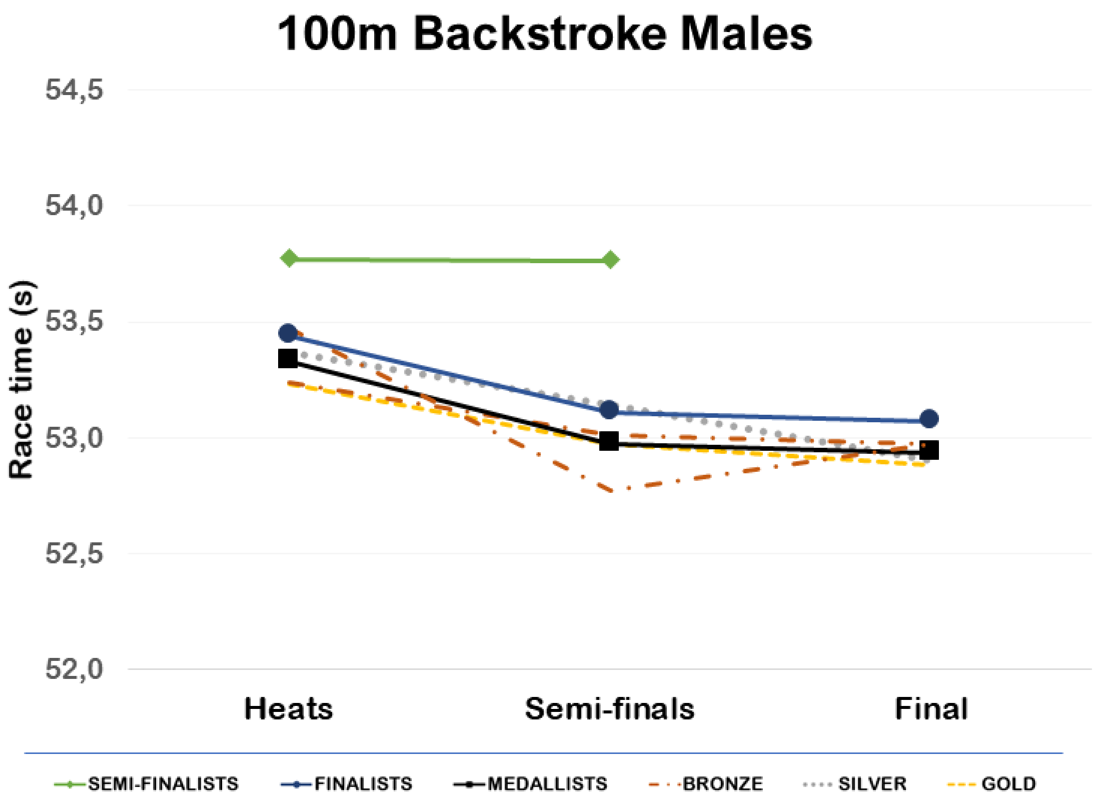

Figure 5. Evolution of the average values of the athletes analyzed in the heats, semi-finals, finals and the individual values of the medalists in the event 100m backstroke male (LEN European Senior Championships 2021).

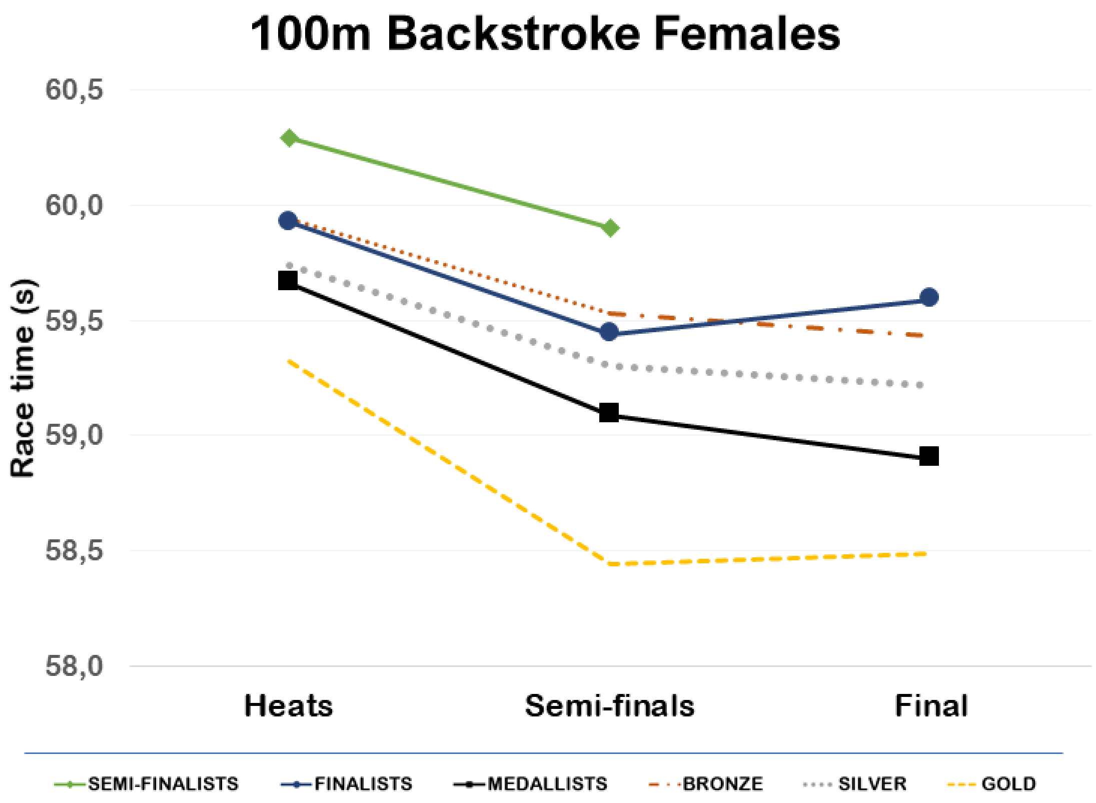

Figure 6. Evolution of the average values of the athletes analyzed in the heats, semi-finals, finals and the individual values of the medalists in the event 100m backstroke female (LEN European Senior Championships 2021).

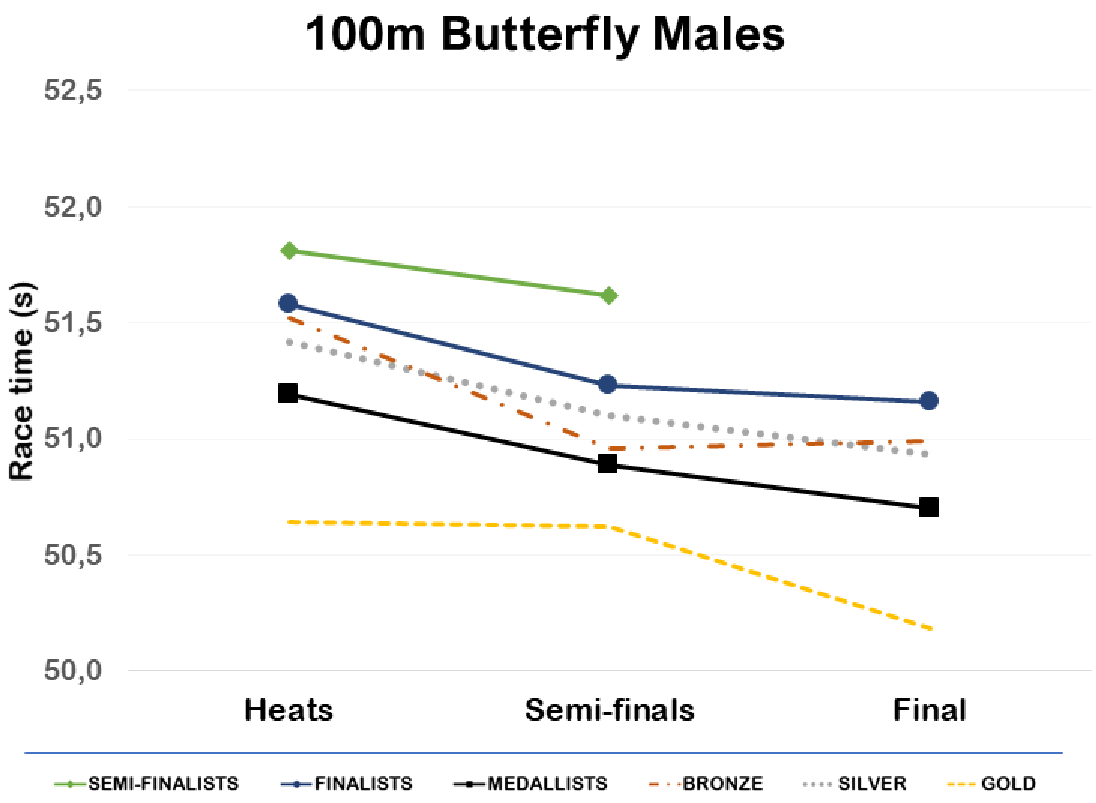

Figure 7. Evolution of the average values of the athletes analyzed in the heats, semi-finals, finals and the individual values of the medalists in the event 100m butterfly male (LEN European Senior Championships 2021).

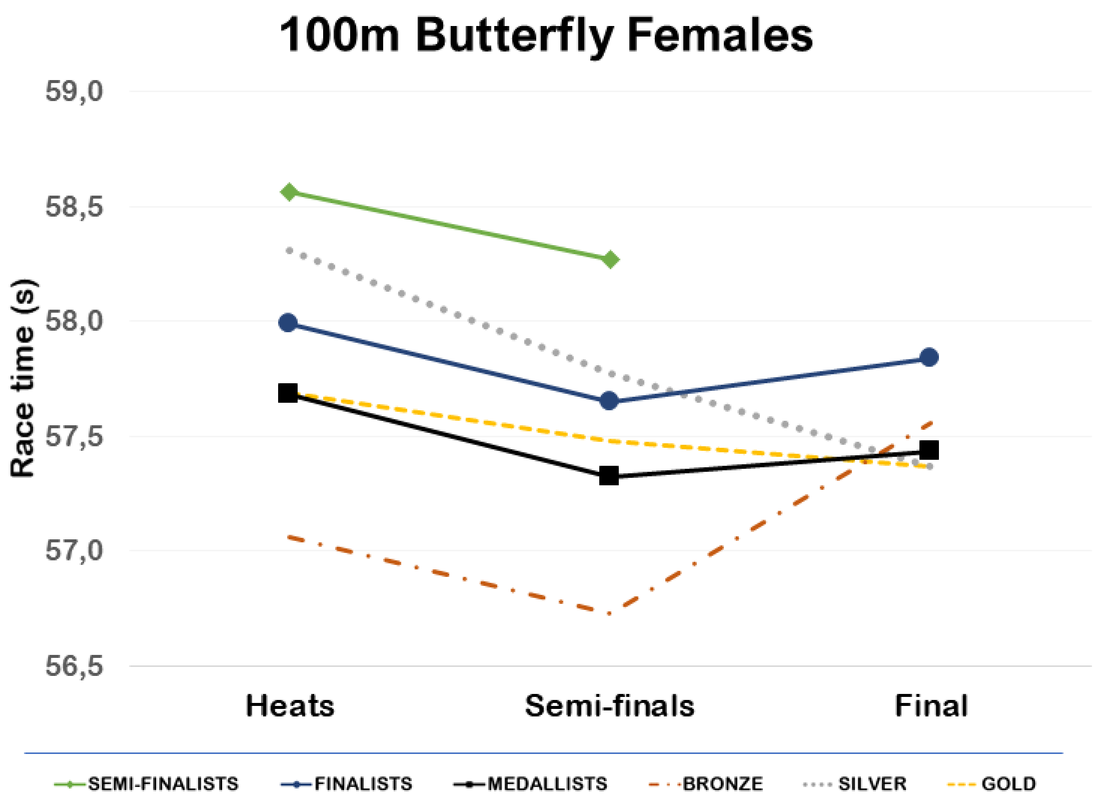

Figure 8. Evolution of the average values of the athletes analyzed in the heats, semi-finals, finals and the individual values of the medalists in the event 100m butterfly female (LEN European Senior Championships 2021).

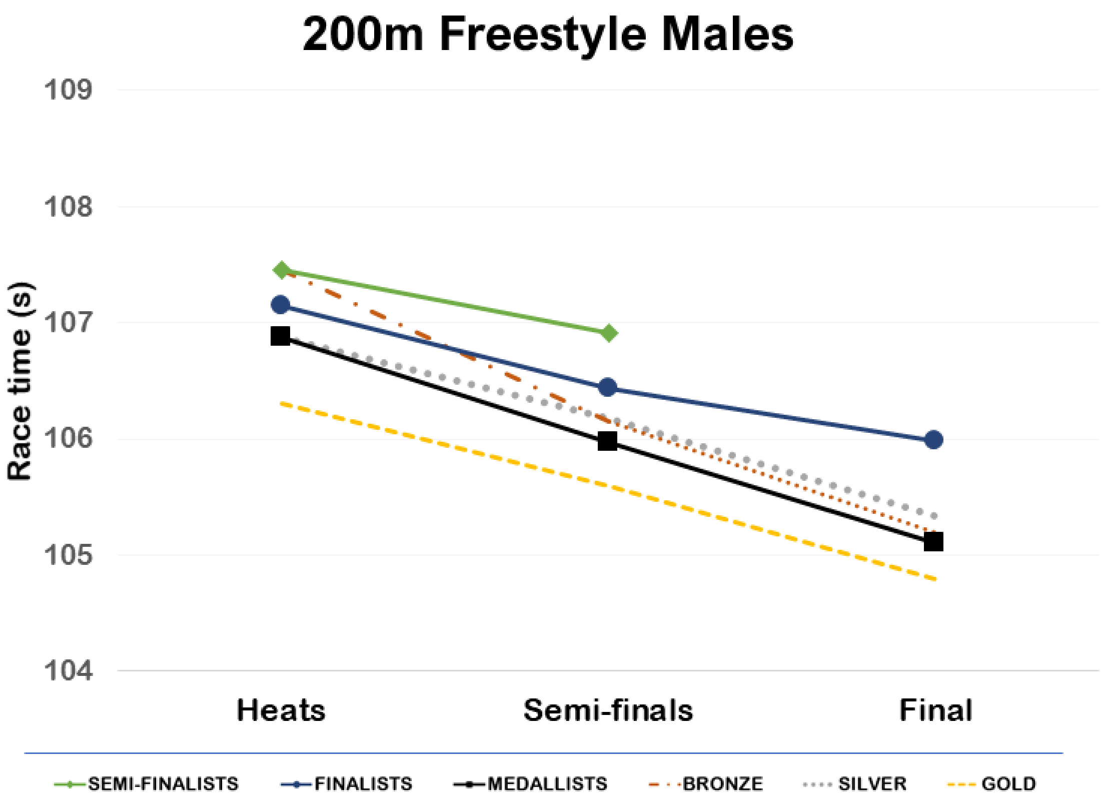

Figure 9. Evolution of the average values of the athletes analyzed in the heats, semi-finals, finals and the individual values of the medalists in the event 200m freestyle male (LEN European Senior Championships 2021).

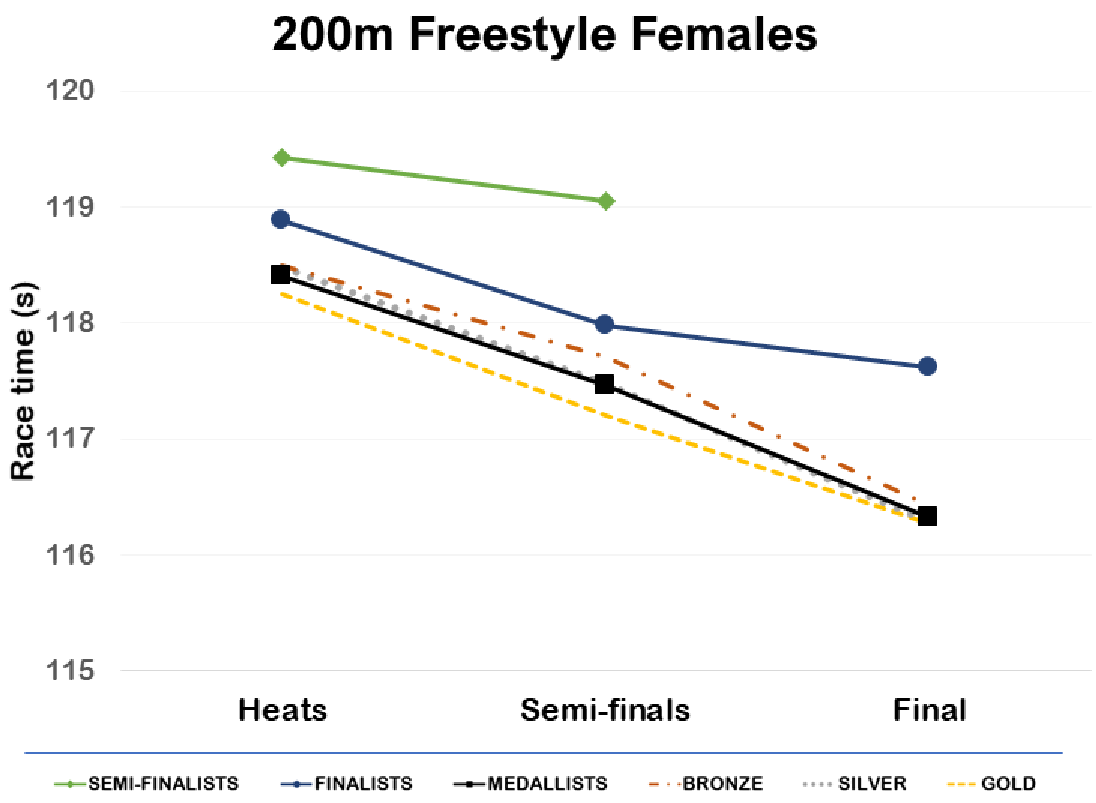

Figure 10. Evolution of the average values of the athletes analyzed in the heats, semi-finals, finals and the individual values of the medalists in the event 200m freestyle female (LEN European Senior Championships 2021).

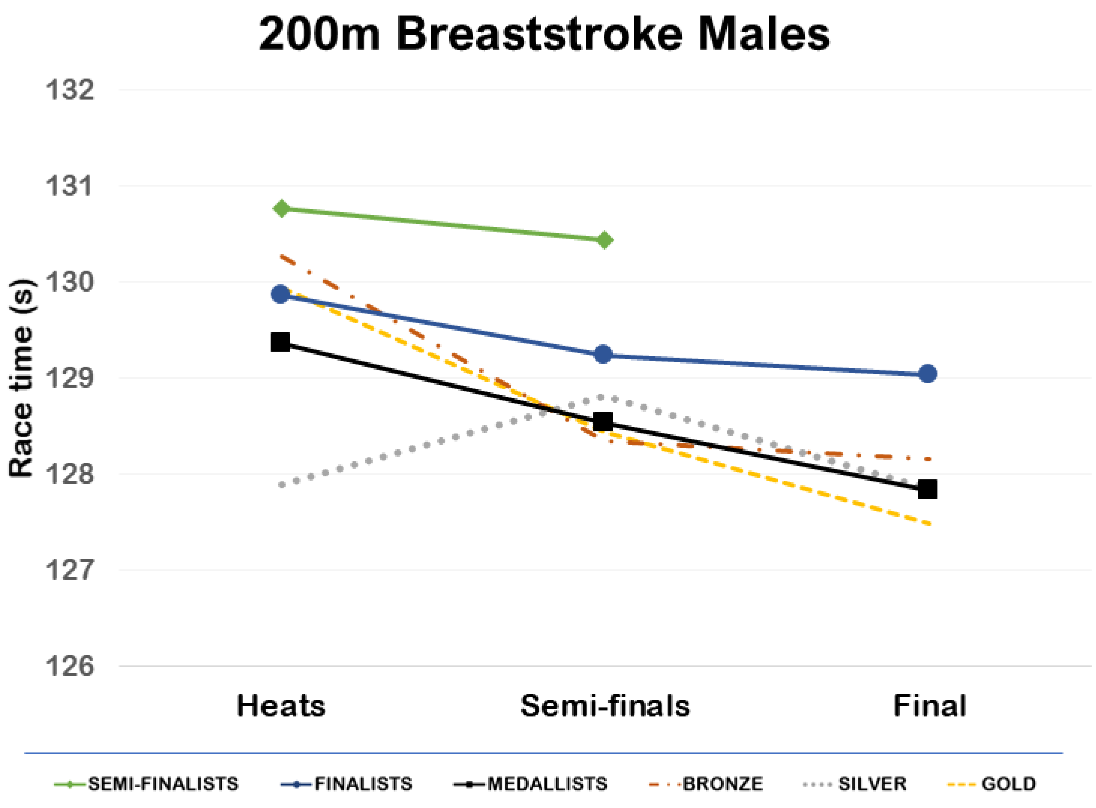

Figure 11. Evolution of the average values of the athletes analyzed in the heats, semi-finals, finals and the individual values of the medalists in the event 200m breaststroke male (LEN European Senior Championships 2021).

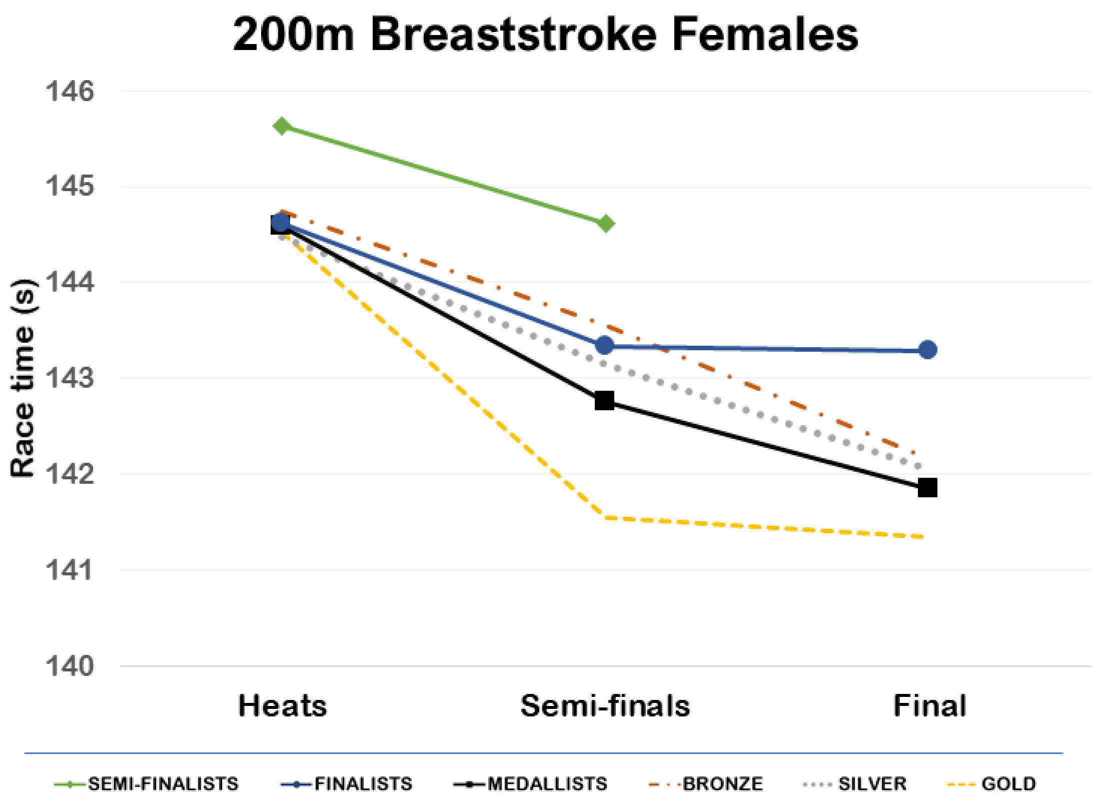

Figure 12. Evolution of the average values of the athletes analyzed in the heats, semi-finals, finals and the individual values of the medalists in the event 200m breaststroke female (LEN European Senior Championships 2021).

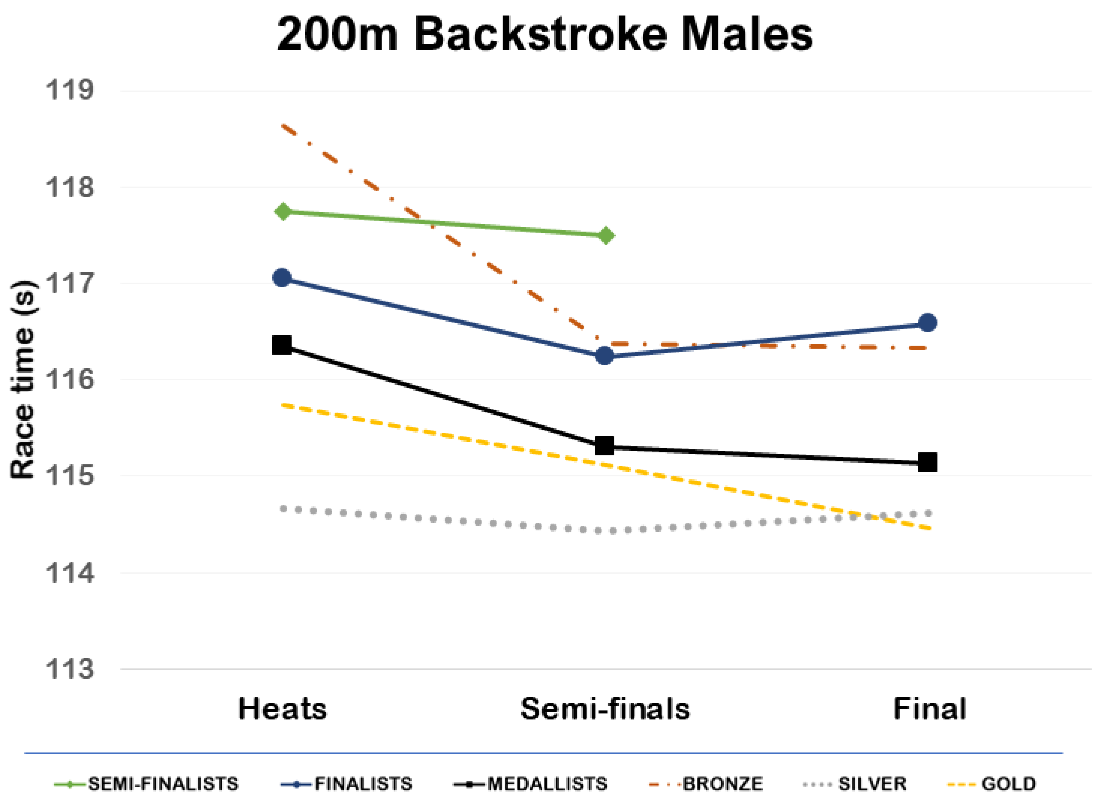

Figure 13. Evolution of the average values of the athletes analyzed in the heats, semi-finals, finals and the individual values of the medalists in the event 200m backstroke male (LEN European Senior Championships 2021).

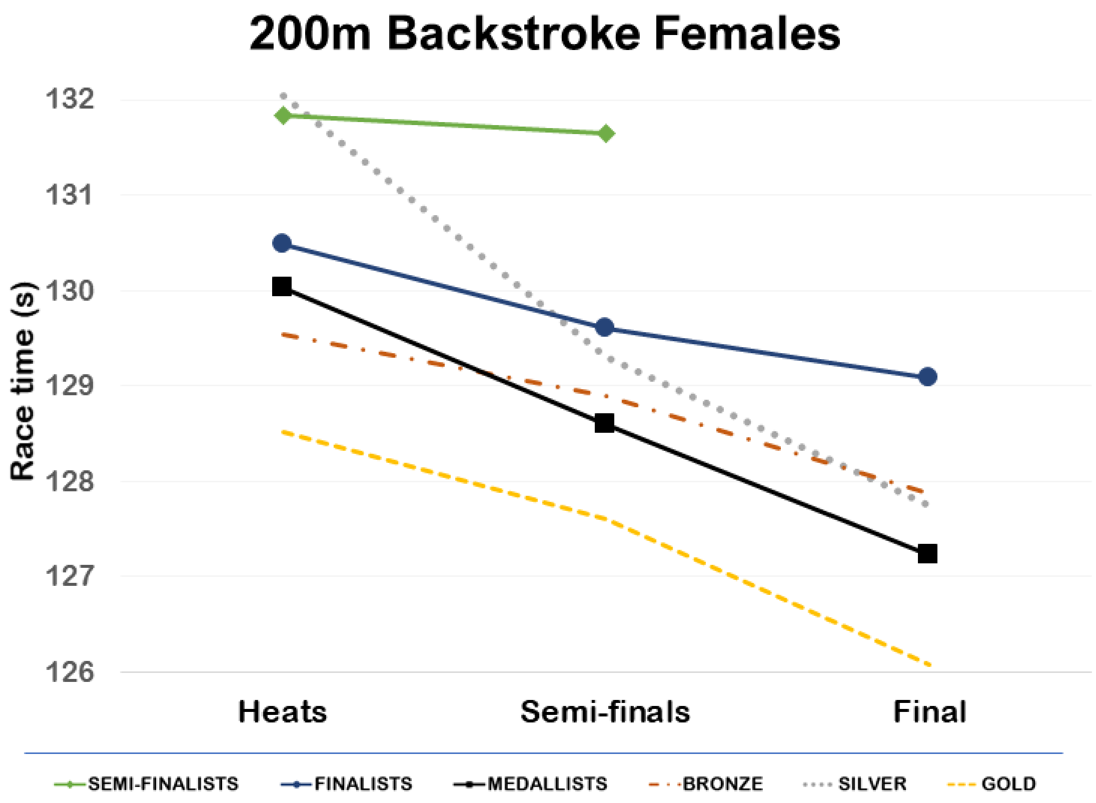

Figure 14. Evolution of the average values of the athletes analyzed in the heats, semi-finals, finals and the individual values of the medalists in the event 200m backstroke female (LEN European Senior Championships 2021).

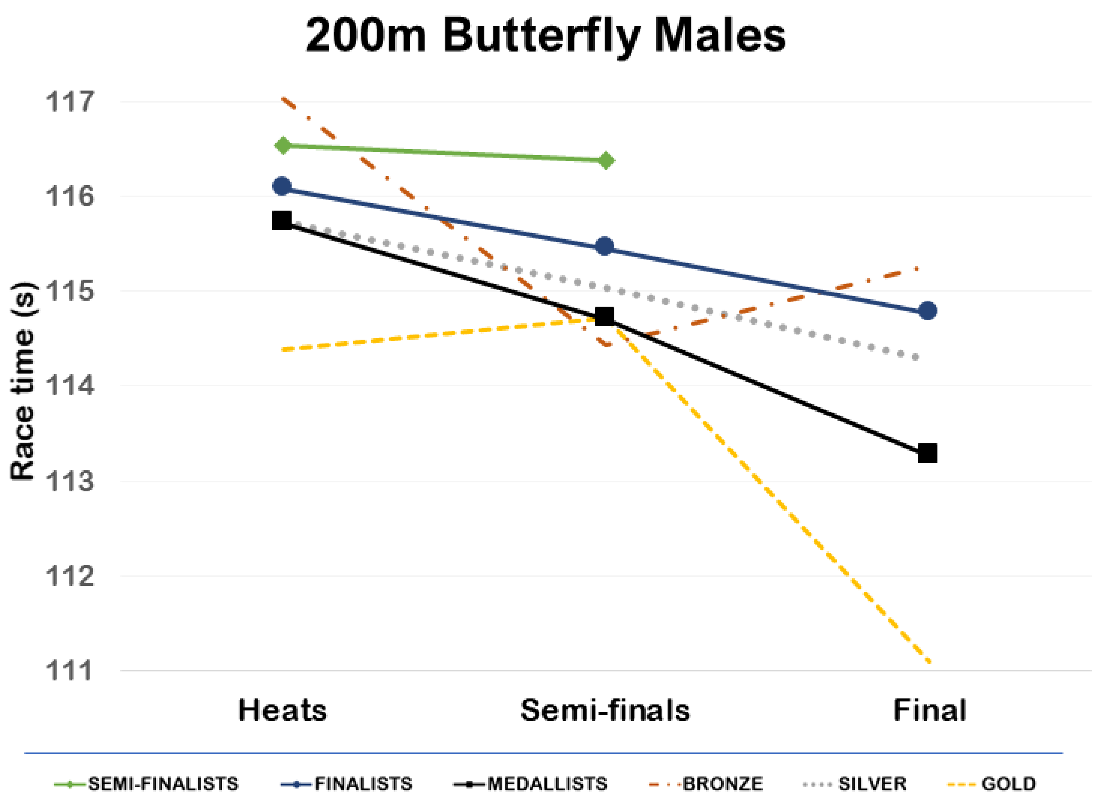

Figure 15. Evolution of the average values of the athletes analyzed in the heats, semi-finals, finals and the individual values of the medalists in the event 200m butterfly male (LEN European Senior Championships 2021).

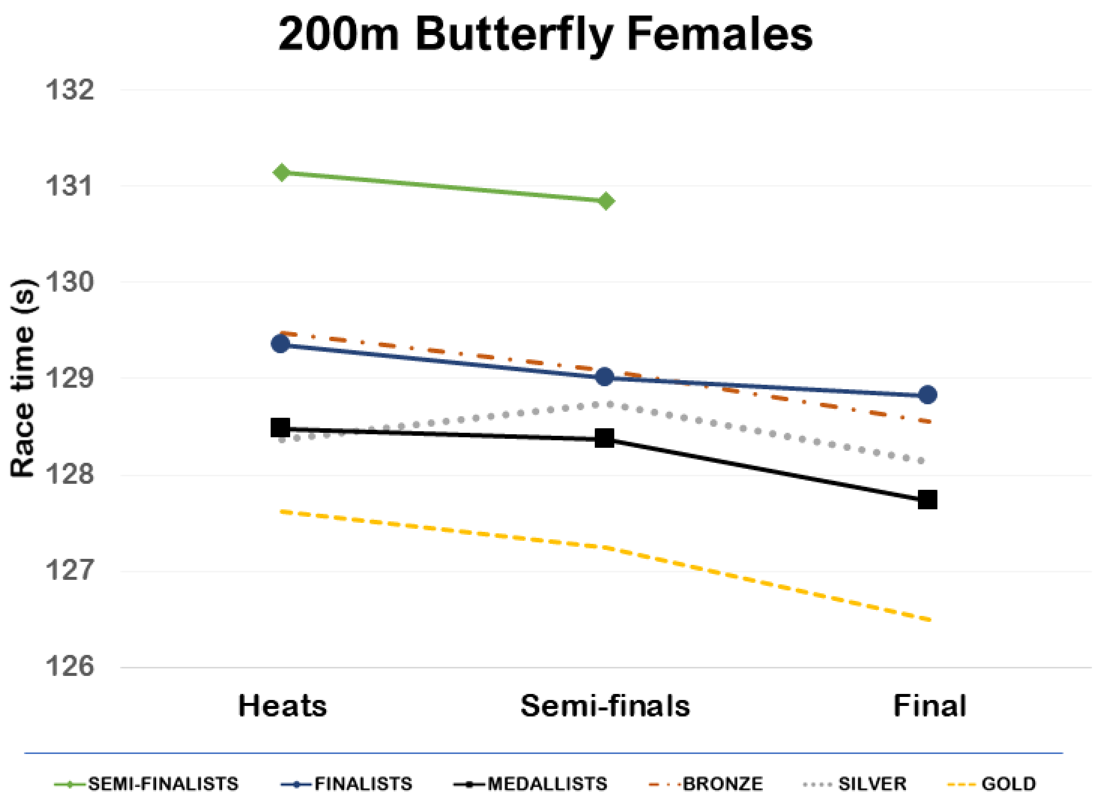

Figure 16. Evolution of the average values of the athletes analyzed in the heats, semi-finals, finals and the individual values of the medalists in the event 200m butterfly female (LEN European Senior Championships 2021).
