# Supplementary material for: Are the 50 m Race Segments Changed From Heats to Finals at the 2021 European Swimming Championships?
Source: Front Physiol. 2022 Jul 13;13:797367. doi: 10.3389/fphys.2022.797367 (PMC9326221; doi:10.3389/fphys.2022.797367)
Supplement: Supplementary file 2 [file Presentation1.PDF]

Description of the collecting data procedures and accuracy applied in the study: Are the 50m race segments changed from heats to finals at the 2021 European Swimming Championships?

Raúl Arellano[1†*], Jesús J. Ruíz-Navarro[†1], Tiago M. Barbosa[‡2,3], Gracia López-Contreras[‡1], Esther Morales-Ortiz[‡1], Ana Gay[1], Oscar López-Belmonte[1], Angela González-Ponce[1], Francisco Cuenca-Fernández[†1]
[1] Aquatics Lab, Department of Physical Education and Sport, Faculty of Sport Sciences, University of Granada, Granada, Spain.
[2] Department of Sport Sciences, Instituto Politécnico de Bragança, Bragança, Portugal
[3] Research Centre in Sports, Health and Human Development, Vila Real, Portugal

***Correspondence:**
Raúl Arellano
r.arellano@ugr.es

How to calibrate and measure in the areas of the pool where water entry or emersion will occur?

In this study, as in many others, accuracy is established by the ratio of the actual distance of the reference on the study plane to the pixels that exist at that distance. The resolution of the video recording system, HD, **fullHD (1920x1080 pixels)** or 4K, means that in the same distance or reference space there may be more or less pixels of resolution. We have not applied procedures that allow the measurement of sub-pixels, which would have made the study extraordinarily complicated.

The measurements of the study area are known thanks to the calibration carried out by means of lane buoys. As described in Figure 1, fifty buoys are 5m and therefore 10 buoys are one meter. This measurement should be done before each championship because the lane manufacturing companies may use buoys of different sizes, the standard being 10cm or 12.5cm per buoy.
The changes of colors allow to differentiate the zones that mark the regulation of competition in swimming 5 and 15m, and additionally the 25m. In our case, given that no swimmer is able to reach further than 5m at the start, we take this reference for the measurement of this hand's contact distance and 15m as the maximum distance of emersion, limited by the regulations.

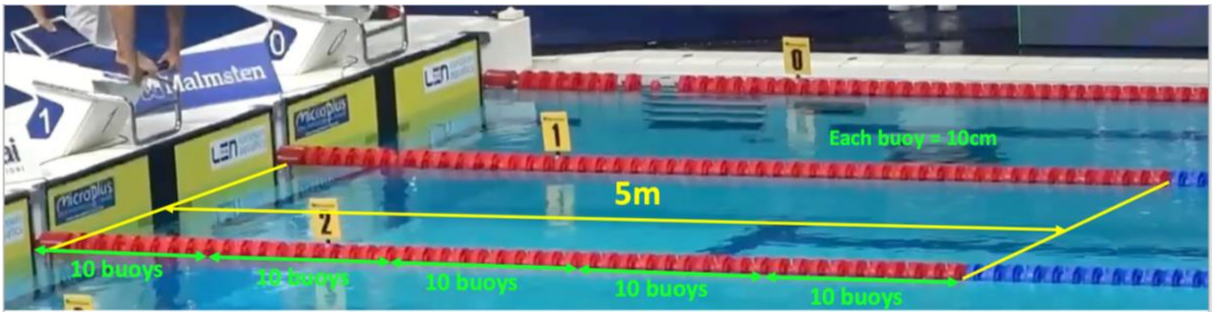

Figure 1: Measurement of the buoys used during the competition, to establish them as a scale while following the swimmer during the event by means of the rotation of the camera.

As can be seen in the following image taken from one of the videos used for our data acquisition, we defined a calibration zone trying to correct the effect of the camera position and its perspective. This allows us to measure the distance as accurately as possible. In the example we use an already calibrated line, but we can also use the calibration in "pixels", set for the reference distance of 5m (where the red buoys of the pool lane end).

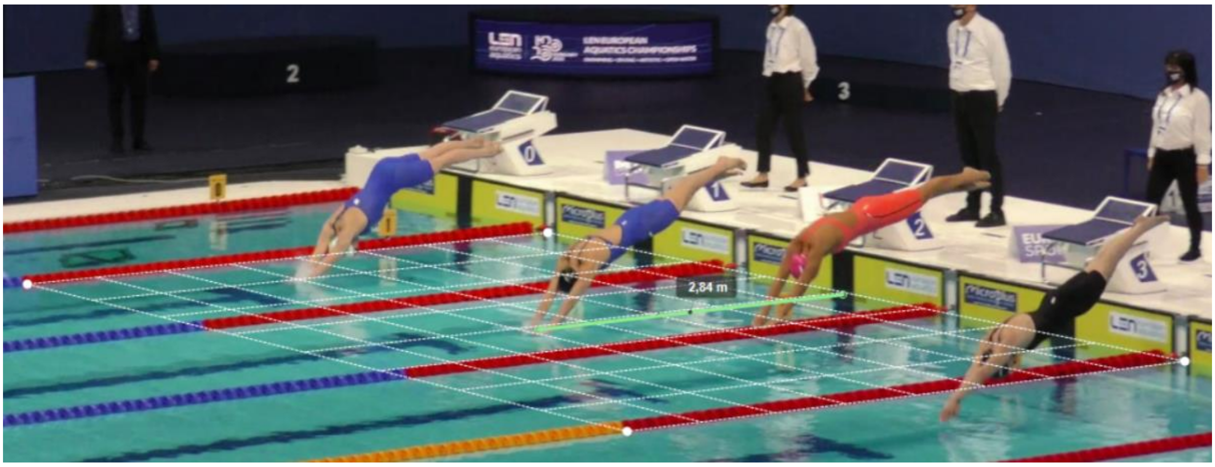

Figure 2: Example of measurement of hand contact distance in the water in a calibrated zone during the exit from the starting block.

In general, basic techniques are used to calibrate a 2D plane, with arbitrary measurements within the plane visible in the video. The following steps have been followed (as described in the instructions for the Kinovea 0.9.5 software used):

a) Have a rectangle of known dimensions visible in the video;
b) Add a perspective grid object and move its corners to match the rectangle;
c) Right click a corner and select the Calibrate menu;
d) Enter the real-world width and height of the rectangle.

The following figure 3 shows an example of how to enter the above information in cited software, in order to define the calibration of the known plane to measure the emersion distance.

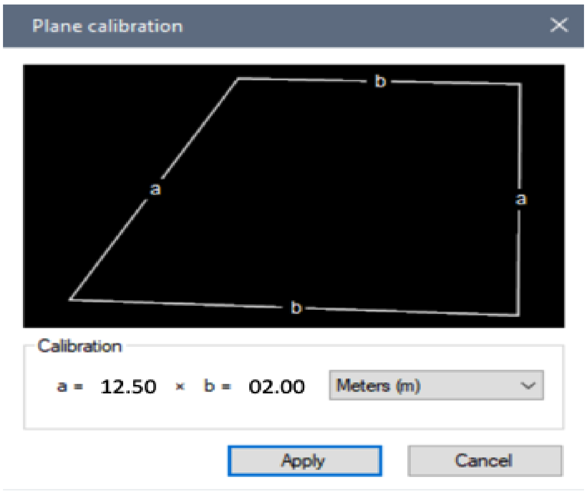

Figure 3: Plane calibration window of the Kinovea 0.9.5 software with the specific data of the example of figure 4.

In this case we have selected an area comprising 2m before the 15m boundary and three lanes of 2.5m distance each (total = 7.5m), see figure 4. The yellow buoys different from the rest of the lanes correspond to the central lanes number 4 and 5. In this case we intend to measure the emersion distance of the swimmers in lanes 3 and 5. The results of the measurements in real coordinates can be seen in Figure 5.

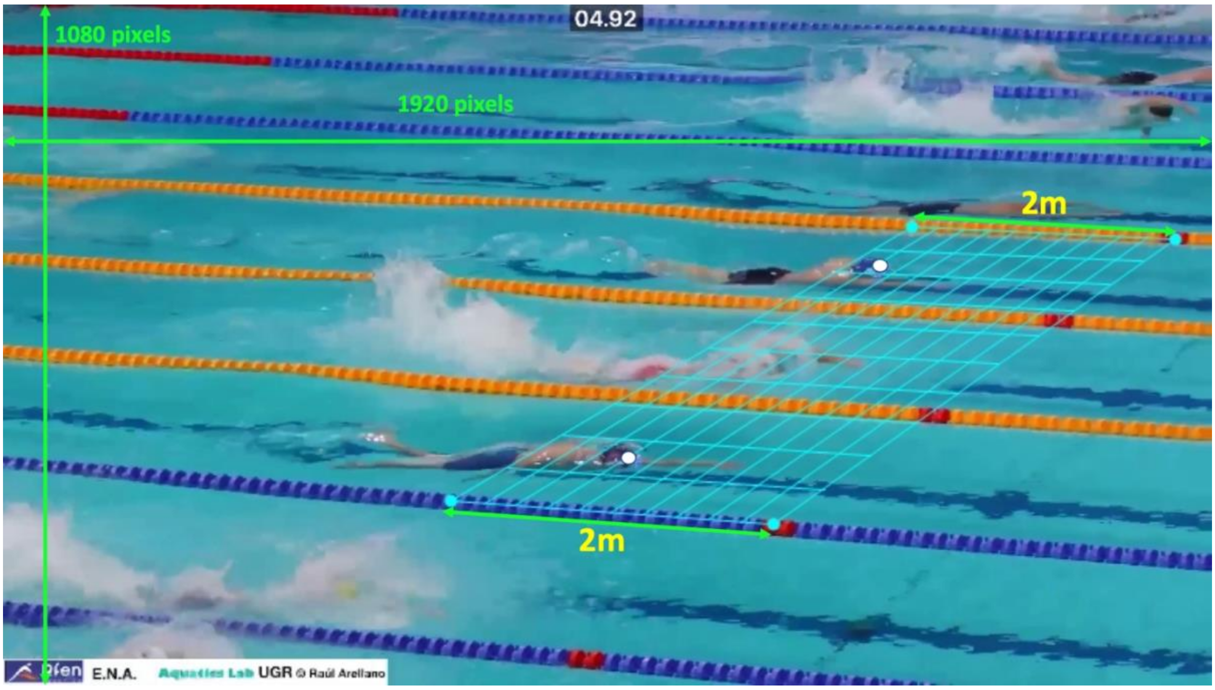

Figure 4: Definition of the emersion distance measurement zone in one of the videos used from the European swimming championships. Frame dimensions have been included: resolution equal to 1920 by 1080 pixels [fullHD].

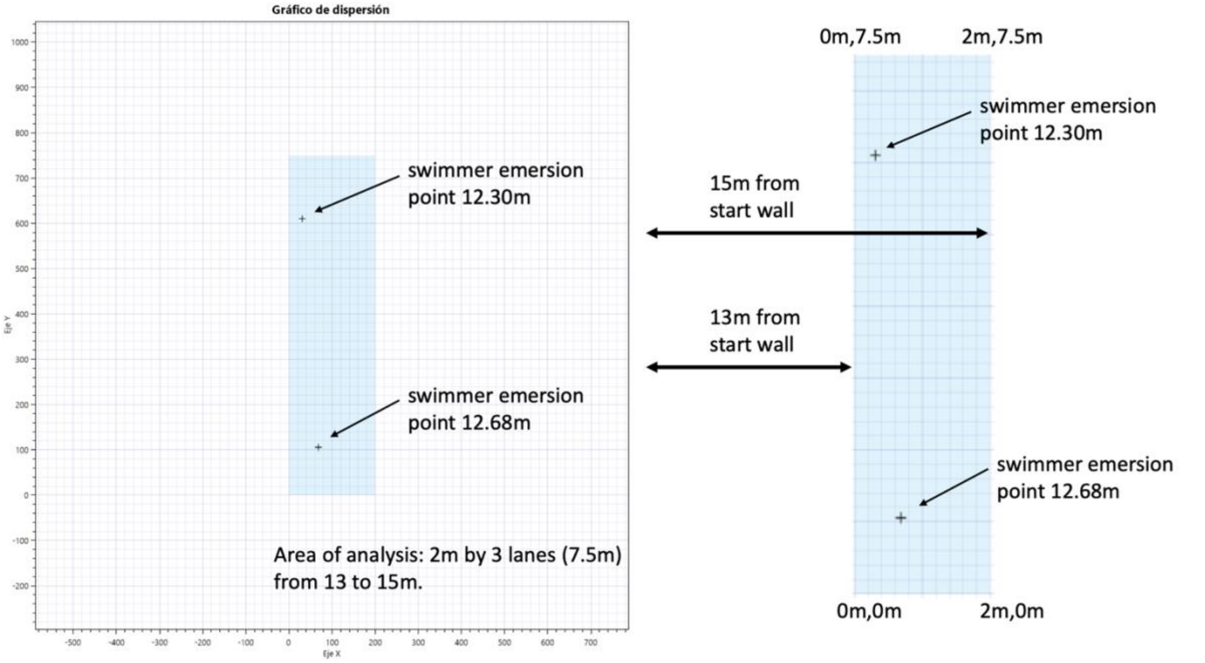

Figure 5: Conversion of coordinates subject to perspective due to the pan-tilt-zoom of the camera to real coordinates. The emersion position of each swimmer within the

analysis zone and the necessary correction to know the total distance from the wall are included.
The method used to follow the swimmers along the pool makes the procedure of calibrating the measurement zones of the emersion distances in particular more complex. The camera moves following the swimmer at a speed as tight as possible, with possible zoom adjustments, which modify the scale dynamically. All this slows down the process of measuring these variables. The above figures attempt to correct this problem as accurately and validly as possible.

What causes variability in accuracy and how we can measure it?

1. Distance between camera and swimmer: The cameras are placed as centered as possible and perpendicular to the longitudinal axis of the pool, but as each camera is focused on a different swimmer, the swimmer in the nearest lane is at a different distance than the swimmer in the farthest lane, necessitating an adjustment in the scale. Longer underwater distances are closer to the central area of the pool.
2. Camera rotation (or panning): this necessitates the establishment of a fixed reference system independent of the rotational movement of the camera on the tripod. The ends of the pool are going to be farther away than the central area so it is necessary in the case of distance measurements the definition of a reference plane in each event and for each swimmer or group of swimmers that may be in plane. As the distances can be very different for each swimmer the establishment of the reference plane occurs after the starts or each turn (not used in this study). The reference system used is the lanes and their buoys, as well as the front walls of the pool, since it is assumed that they do not move during the competition.
3. Possible zoom variations: the cameramen, when following the swimmer, may need to adjust the recording area by use of the camera zoom. This introduces the possibility of additional adjustment of the calibration space.

Figures 6 and 7 below serve as an example of how in two different areas of the pool, near the front wall of the start and in the emersion zone (about 15m), the pixel measurements of lanes of known dimensions, thanks to the fixed reference of the buoys, have different values due to a greater distance to the camera and different values of the zoom used.

Doing some simple calculations on the information given in the images you can see how due to the perspective in figure 6, the 5m length of the red buoys measures 766, 774 and 777 pixels respectively. This would give us an ***accuracy value of 1.55, 1.548 and 1.532 pixels per centimeter.*** This, in a general way, would be approximately the accuracy in the area of the exit to measure the contact distance in the water (it should be remembered that this can change from camera to camera and from lane to lane, although with very similar values.

In Figure 7, as the swimmer is closer to the central area where the cameras are located, the accuracy increases slightly as shown in the following data: for a distance measured with the buoys of 2m we have in each measurement 469 and 484 pixels respectively. This gives us a value of ***2.345 and 2.42 pixels per centimeter***, which is more than reasonable for measuring the emersion distance.

It should be remembered here that, depending on the emersion distance of each swimmer, the lane of participation and the camera that records it, it will often be necessary to define an individual reference system for each zone of the pool.

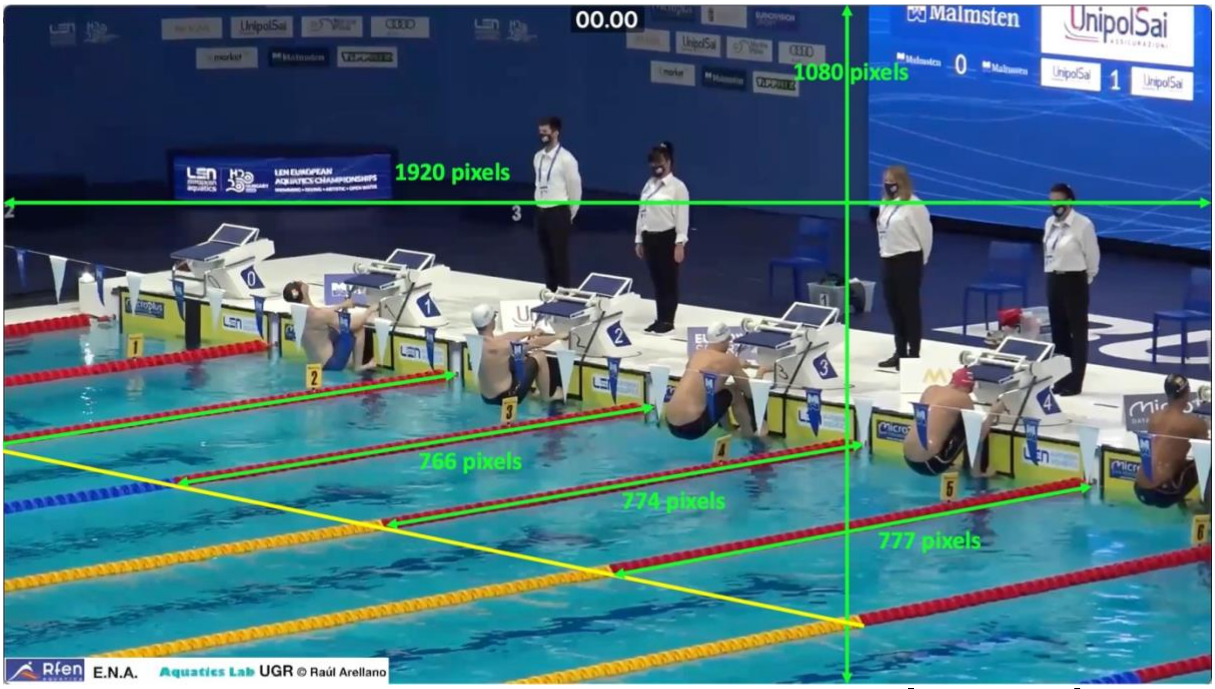

Figure 6: Example frame with the pixel dimensions of "Full HD" [1920x1080] and how the distance used as a reference (the red color of the 5m lane) changes its length in pixels depending on the distance from the camera position.

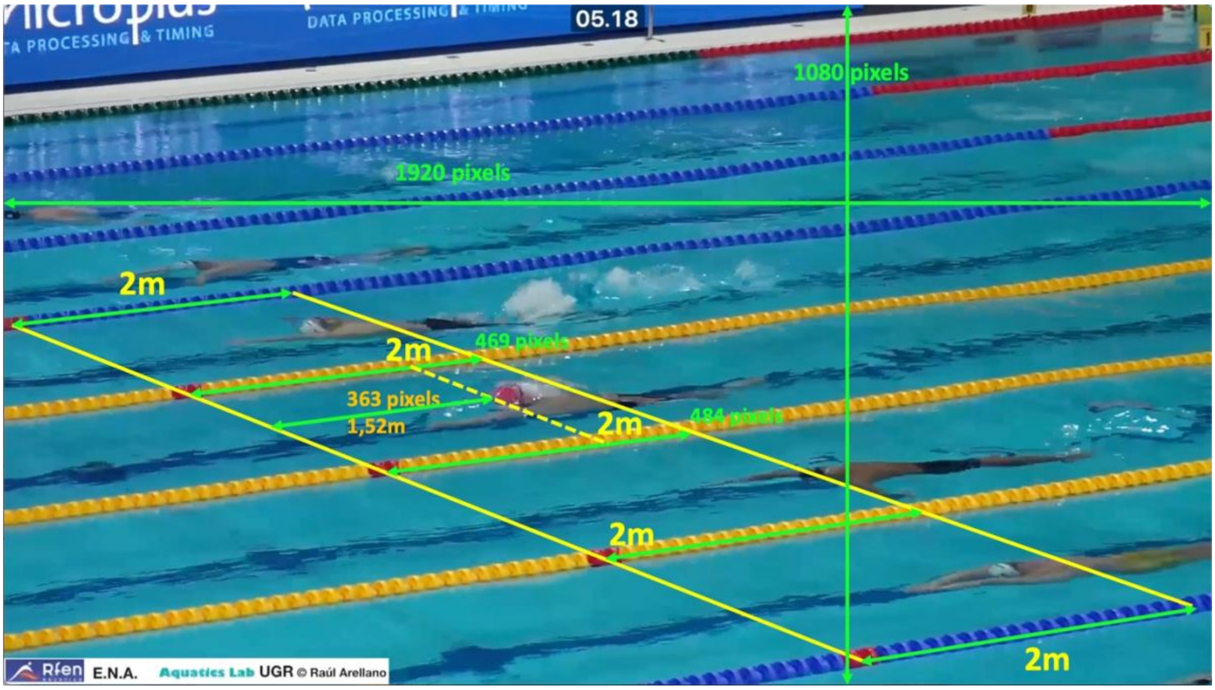

Figure 7: Example of emersion distance measurement in an area where it is not possible to observe reference buoys (e.g. from 5m to 15m) based on the standard buoy size of 10cm. This example shows the emersion distance measurement on the swimmer in lane 4, in a reference zone of 2m. The swimmer emerges 1.52m from the 15m distance, for a total of 13.48m of total emersion distance on his emersion.

About the accuracy of stroke frequency and related variables.

The video frame rate indicated (50Hz) is the one commonly used in this type of studies at present. In the three areas of the pool, where the cycle frequency was measured [from 15m to 25m, from 35 to 45m and from 45 to 50m] the time taken to perform 3 cycles or 2 cycles (in the last 5m) was measured.

In the case of the 3-cycle time measurement, if a swimmer takes 2.80s, his frequency is 1.071Hz, or 64.29 cic/min, the unit used in practice by coaches and swimmers. In this case, measuring one frame more or less means obtaining a value of 2.82 or 2.78s, which would change the frequency to 63.83 cic/min or 64.75 cic/min, i.e. plus or minus 0.46 cic/min. In this range of values is about 0.72% error, something perfectly acceptable in this type of study, where in practice coaches and swimmers only use integers.

To measure the stroke length, we use the base equation V = SL * SF. From there we clear and therefore SL = V (m/s) / SF (Hz). Following with the previous example and considering an average speed of 1.82 m/s, SL would be equal to 1.699m. If we introduce a frame of variation in the frequency, the SL could vary between 1.711 and 1.68, or in other words plus or minus 0.011m or 0.64%. Also, a more than acceptable value for this type of study.

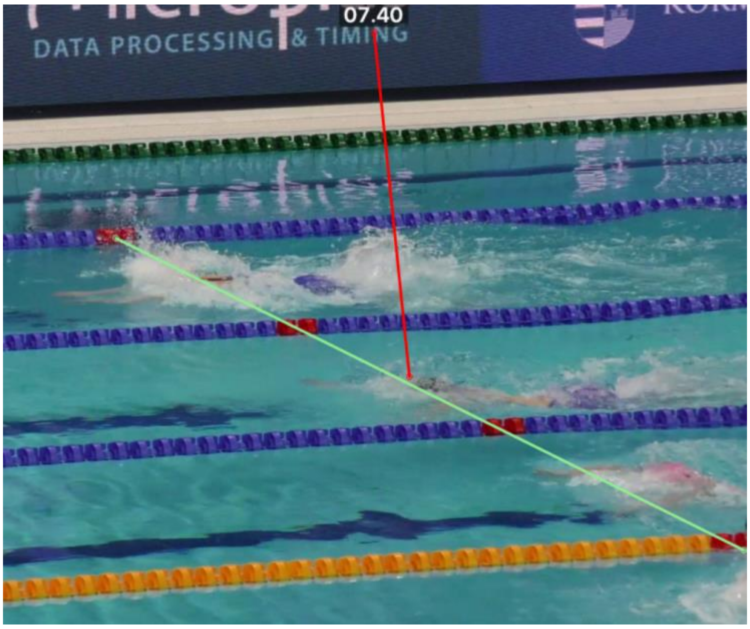

Figure 8: How the 15m time is measured in breaststroke.

The effect on calculated velocity from measuring when the head crossed a specific distance reference (see figure 8), for example, from 15m to 25m, it can be calculated in the same way. First, it should be confirmed that the buoys have not be displaced (the organization provided to us pictures with these calibrations procedures). Later, the time measured between 15m to 25m [6.04s] can be affected with a frame error in one or both locations. For example, a male breaststroker can obtain a velocity of 1.655 m/s (10m/6.04s) between these references. If a frame is added the velocity change to 1.650m/s (10m/6.06s), if two frames are added the velocity changes to 1.644m/s

(10m/6.08s). Both situations introduce a value of percentage of variation of 0.03% and 0.06 % respectively.

The results shown in the previous examples give you an idea of the low level of error that characterizes all recent publications on this type of measures in competition. This is the big difference over laboratory measurements where everything can be much more precise, but in many cases less relevant, if you take into account that in this case, we have measured all the best 50m swimmers of a continent like Europe. And above all, very few months after the end of the competition.
