# Supplementary material for: Are the 50 m Race Segments Changed From Heats to Finals at the 2021 European Swimming Championships?
Source: Front Physiol. 2022 Jul 13;13:797367. doi: 10.3389/fphys.2022.797367 (PMC9326221; doi:10.3389/fphys.2022.797367)

LEN European Aquatics Championships [BUDAPEST, Hungria] 17/05/2021 - 23/05/2021

50 Breaststroke Female

						LANE	Pos.	RT	T_entr	T_emer	t15	t25	t35	t45	T50	2nd 25	F01	F02	F03	LC1	LC2	LC3	Flight_T	T_Underw_01	D_Underw	Speed Underw
F	Breaststroke	Braza	50	Final	Final	4	1	0,71	1,04	5,32	7,44	13,50	19,62	26,00	29,35	15,85	71,43	69,23	65,22	1,39	1,39	1,24	0,33	4,28	9,00	2,10
F	Breaststroke	Braza	50	Final	Final	6	2	0,63	0,94	4,62	7,16	13,66	20,18	26,74	30,19	16,53	65,22	62,94	63,16	1,42	1,46	1,24	0,31	3,68	8,00	2,17
F	Breaststroke	Braza	50	Final	Final	5	3	0,68	1,06	5,52	7,72	13,92	20,32	26,70	30,22	16,30	66,18	66,67	67,42	1,46	1,41	1,14	0,38	4,46	9,00	2,02
F	Breaststroke	Braza	50	Final	Final	3	4	0,67	0,90	5,14	7,74	13,92	20,32	26,94	30,23	16,31	60,81	62,07	62,50	1,60	1,48	1,31	0,23	4,24	8,60	2,03
F	Breaststroke	Braza	50	Final	Final	7	5	0,70	1,06	5,70	7,38	13,76	20,20	26,82	30,31	16,55	55,56	53,25	51,72	1,69	1,73	1,50	0,36	4,64	9,30	2,00
F	Breaststroke	Braza	50	Final	Final	2	6	0,63	0,94	5,22	7,64	13,92	20,34	27,08	30,35	16,43	59,60	59,21	61,22	1,60	1,54	1,35	0,31	4,28	8,70	2,03
F	Breaststroke	Braza	50	Final	Final	1	7	0,69	0,94	4,94	7,28	13,88	20,50	27,38	30,72	16,84	66,18	64,29	63,16	1,37	1,38	1,28	0,25	4,00	8,40	2,10
F	Breaststroke	Braza	50	Final	Final	8	8	0,71	1,02	5,84	7,44	14,06	20,38	27,42	30,77	16,71	69,77	65,69	61,86	1,30	1,37	1,30	0,31	4,82	9,40	1,95
						MEANS		0,68	0,99	5,29	7,48	13,83	20,23	26,89	30,27	16,44	64,34	62,92	62,03	1,48	1,47	1,29	0,31	4,30	8,80	2,05

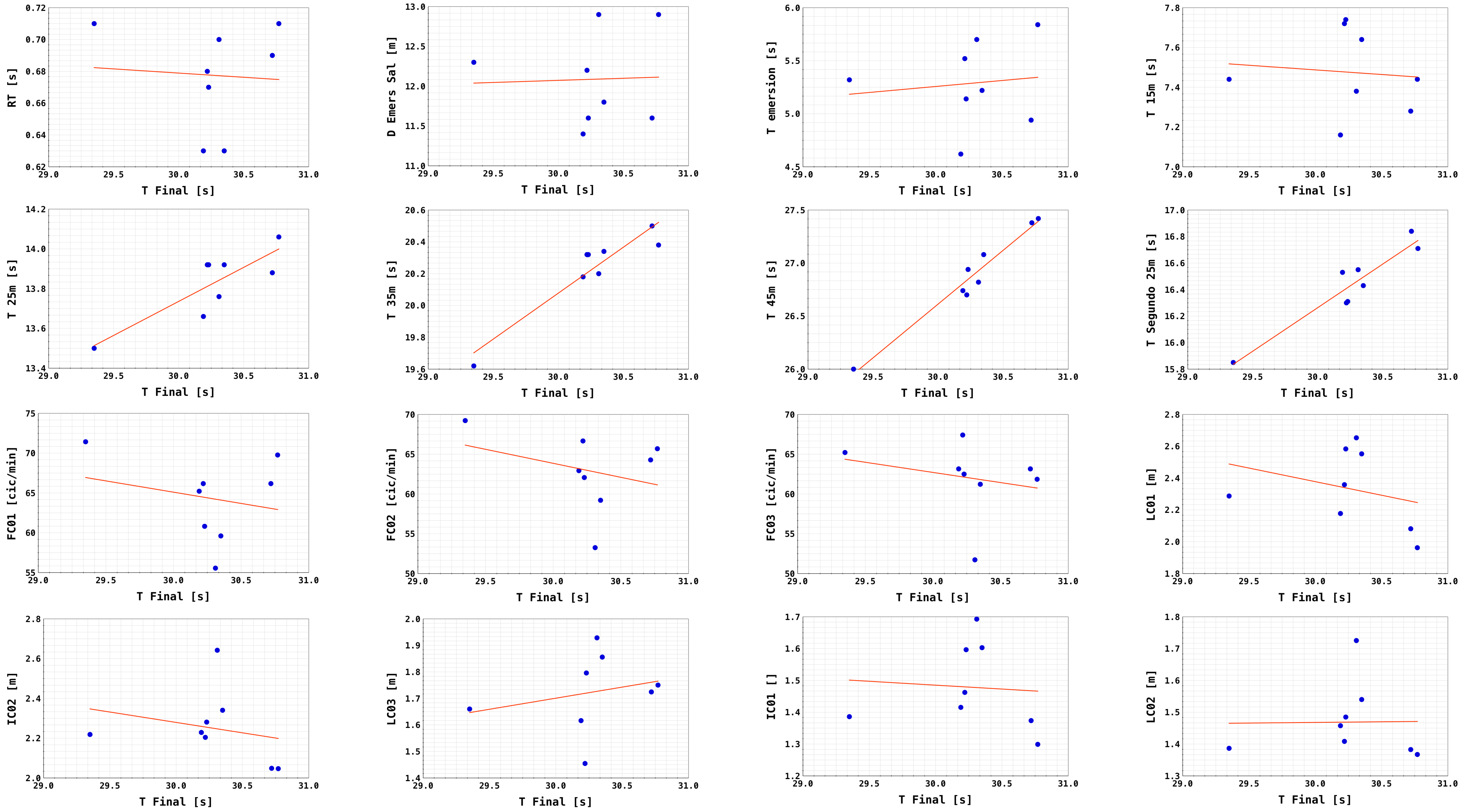

LEN European Aquatics Championships [BUDAPEST, Hungria] 17/05/2021 - 23/05/2021

50 Breaststroke Female

						LANE	Pos.	RT	T_entr	T_emer	t15	t25	t35	t45	T50	2nd 25	F01	F02	F03	LC1	LC2	LC3	Flight_T	T_Underw_01	D_Underw	Speed Underw
F	Breaststroke	Braza	50	Heat 6 of 6	Heat	4	1	0,69	1,00	5,36	7,24	13,50	19,56	26,16	29,50	16,00	71,43	69,77	67,42	1,34	1,36	1,20	0,31	4,36	8,90	2,04
F	Breaststroke	Braza	50	Heat 4 of 6	Heat	5	1	0,65	0,96	5,38	7,58	13,86	20,36	26,98	30,15	16,29	63,38	60,00	60,00	1,51	1,52	1,42	0,31	4,42	8,60	1,95
F	Breaststroke	Braza	50	Heat 6 of 6	Heat	5	2	0,64	0,94	4,40	7,50	13,88	20,48	26,82	30,40	16,52	63,38	60,81	63,16	1,48	1,52	1,19	0,30	3,46	7,40	2,14
F	Breaststroke	Braza	50	Heat 5 of 6	Heat	5	1	0,74	1,04	5,78	7,42	13,88	20,28	26,98	30,46	16,58	53,89	52,63	53,57	1,72	1,74	1,45	0,30	4,74	9,80	2,07
F	Breaststroke	Braza	50	Heat 5 of 6	Heat	4	2	0,71	1,10	5,74	7,80	14,14	20,68	27,16	30,55	16,41	63,83	65,22	66,67	1,48	1,41	1,19	0,39	4,64	8,90	1,92
F	Breaststroke	Braza	50	Heat 6 of 6	Heat	6	3	0,67	0,94	5,40	7,96	14,16	20,52	27,38	30,58	16,42	58,82	57,69	58,25	1,65	1,57	1,45	0,27	4,46	8,50	1,91
F	Breaststroke	Braza	50	Heat 5 of 6	Heat	3	3	0,72	1,00	5,46	7,36	14,02	20,62	27,20	30,63	16,61	56,60	54,88	52,63	1,59	1,66	1,50	0,28	4,46	9,00	2,02
F	Breaststroke	Braza	50	Heat 5 of 6	Heat	2	4	0,65	0,96	5,66	7,32	13,84	20,56	27,22	30,64	16,80	58,06	52,94	54,05	1,58	1,69	1,46	0,31	4,70	9,30	1,98
F	Breaststroke	Braza	50	Heat 6 of 6	Heat	3	4	0,71	0,98	5,58	7,44	14,08	20,62	27,34	30,81	16,73	57,32	56,96	59,41	1,58	1,59	1,31	0,27	4,60	9,00	1,96
F	Breaststroke	Braza	50	Heat 6 of 6	Heat	2	5	0,67	1,06	5,58	7,96	14,24	20,74	27,44	30,83	16,59	68,70	67,16	63,83	1,39	1,35	1,25	0,39	4,52	8,80	1,95
F	Breaststroke	Braza	50	Heat 5 of 6	Heat	8	5	0,73	1,00	4,88	7,44	13,94	20,60	27,46	30,95	17,01	67,16	65,22	63,16	1,37	1,36	1,22	0,27	3,88	8,30	2,14
F	Breaststroke	Braza	50	Heat 4 of 6	Heat	3	3	0,70	1,02	5,88	7,60	14,34	20,84	27,52	31,05	16,71	68,18	64,29	61,86	1,31	1,42	1,24	0,32	4,86	9,40	1,93
F	Breaststroke	Braza	50	Heat 5 of 6	Heat	7	6	0,67	0,96	4,84	7,44	14,06	20,58	27,52	31,09	17,03	63,38	60,00	60,00	1,43	1,49	1,26	0,29	3,88	7,90	2,04
F	Breaststroke	Braza	50	Heat 2 of 6	Heat	3	1	0,67	0,97	6,28	7,76	14,34	21,08	28,00	31,19	16,85	56,96	60,00	57,14	1,60	1,46	1,48	0,30	5,31	8,90	1,68
F	Breaststroke	Braza	50	Heat 6 of 6	Heat	8	6	0,73	1,00	5,54	7,72	14,28	20,98	28,10	31,42	17,14	59,21	56,25	54,55	1,54	1,54	1,49	0,27	4,54	9,40	2,07
F	Breaststroke	Braza	50	Heat 6 of 6	Heat	0	7	0,61	0,84	5,86	7,58	14,34	21,08	27,98	31,46	17,12	50,28	48,13	46,88	1,77	1,83	1,66	0,23	5,02	10,30	2,05
						MEANS		0,69	0,99	5,48	7,57	14,06	20,60	27,33	30,73	16,68	61,29	59,50	58,91	1,52	1,53	1,36	0,30	4,49	8,90	1,99

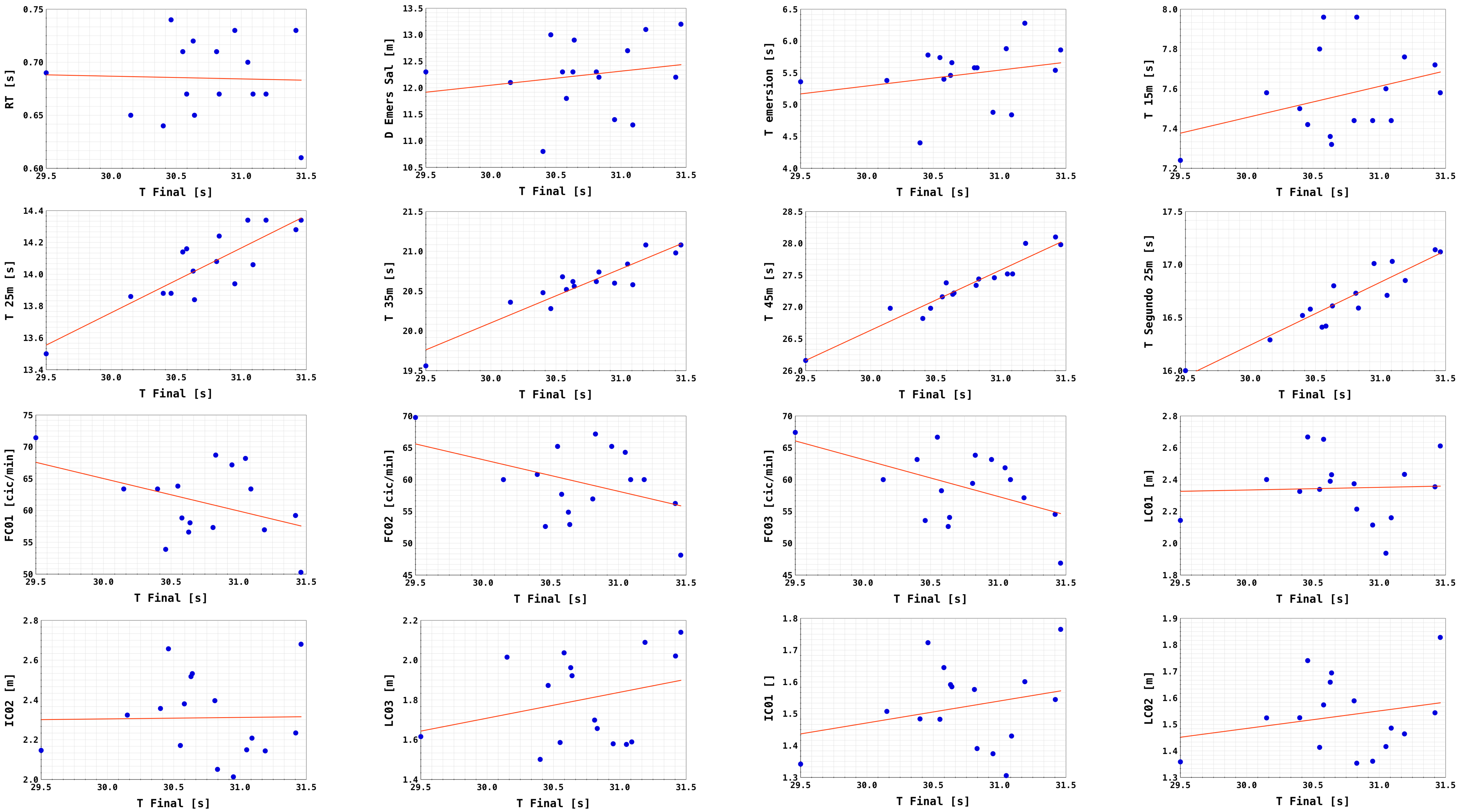

LEN European Aquatics Championships [BUDAPEST, Hungria] 17/05/2021 - 23/05/2021

50 Breaststroke Female

						LANE	Pos.	RT	T_entr	T_emer	t15	t25	t35	t45	T50	2nd 25	F01	F02	F03	LC1	LC2	LC3	Flight_T	T_Underw_01	D_Underw	Speed Underw
F	Breaststroke	Braza	50	Semifinal 2	SF	4	1	0,68	1,00	5,38	7,24	13,46	19,56	25,98	29,30	15,84	69,77	67,67	63,16	1,38	1,42	1,29	0,32	4,38	9,20	2,10
F	Breaststroke	Braza	50	Semifinal 2	SF	3	2	0,69	1,06	5,54	7,86	13,98	20,46	26,92	30,25	16,27	62,07	64,29	64,52	1,58	1,44	1,26	0,37	4,48	8,70	1,94
F	Breaststroke	Braza	50	Semifinal 1	SF	3	1	0,66	0,92	4,98	7,70	14,06	20,52	26,98	30,35	16,29	60,40	60,00	60,61	1,56	1,55	1,32	0,26	4,06	7,90	1,95
F	Breaststroke	Braza	50	Semifinal 2	SF	5	3	0,63	0,94	4,68	7,24	13,80	20,20	26,70	30,39	16,59	63,83	63,38	61,86	1,43	1,47	1,18	0,31	3,74	8,00	2,14
F	Breaststroke	Braza	50	Semifinal 1	SF	4	2	0,65	0,96	5,42	7,80	14,08	20,64	27,10	30,44	16,36	58,82	58,82	60,61	1,62	1,57	1,33	0,31	4,46	8,90	2,00
F	Breaststroke	Braza	50	Semifinal 1	SF	5	3	0,69	1,04	5,46	7,34	13,90	20,60	27,06	30,53	16,63	55,56	52,94	58,25	1,65	1,72	1,34	0,35	4,42	9,10	2,06
F	Breaststroke	Braza	50	Semifinal 2	SF	7	4	0,69	0,96	5,08	7,40	13,82	20,54	27,16	30,76	16,94	66,18	63,83	63,16	1,41	1,41	1,19	0,27	4,12	8,80	2,14
F	Breaststroke	Braza	50	Semifinal 1	SF	7	4	0,69	1,00	5,96	7,52	14,12	20,62	27,30	30,84	16,72	67,16	65,22	61,22	1,35	1,40	1,25	0,31	4,96	9,80	1,98
F	Breaststroke	Braza	50	Semifinal 1	SF	6	5	0,64	0,92	5,60	7,34	13,94	20,64	27,30	30,88	16,94	57,69	54,22	54,05	1,58	1,66	1,40	0,28	4,68	9,60	2,05
F	Breaststroke	Braza	50	Semifinal 2	SF	6	5	0,69	0,94	5,12	7,58	14,02	20,78	27,26	30,90	16,88	59,60	57,32	67,42	1,56	1,58	1,10	0,25	4,18	8,60	2,06
F	Breaststroke	Braza	50	Semifinal 2	SF	2	6	0,68	0,96	5,44	7,44	14,04	20,54	27,42	30,91	16,87	60,00	60,00	57,69	1,52	1,49	1,34	0,28	4,48	9,10	2,03
F	Breaststroke	Braza	50	Semifinal 1	SF	2	6	0,66	1,04	5,30	7,80	13,82	20,70	27,46	30,97	17,15	69,77	67,16	82,19	1,43	1,31	0,94	0,38	4,26	8,40	1,97
F	Breaststroke	Braza	50	Semifinal 1	SF	1	7	0,67	0,98	6,08	7,70	14,20	20,92	27,86	31,04	16,84	57,69	58,25	57,14	1,60	1,51	1,49	0,31	5,10	9,60	1,88
F	Breaststroke	Braza	50	Semifinal 2	SF	1	7	0,65	0,94	4,84	7,32	13,94	20,56	27,58	31,04	17,10	63,38	60,00	59,41	1,43	1,47	1,31	0,29	3,90	8,20	2,10
F	Breaststroke	Braza	50	Semifinal 1	SF	8	8	0,66	0,88	5,90	7,62	14,28	20,92	28,06	31,37	17,09	49,72	48,13	49,18	1,81	1,81	1,66	0,22	5,02	10,00	1,99
F	Breaststroke	Braza	50	Semifinal 2	SF	8	8	0,78	1,02	5,32	7,86	14,20	21,02	28,12	31,51	17,31	58,82	56,25	55,05	1,61	1,53	1,45	0,24	4,30	9,10	2,12
						MEANS		0,68	0,97	5,38	7,55	13,98	20,58	27,27	30,72	16,74	61,28	59,84	60,97	1,53	1,52	1,30	0,30	4,41	8,94	2,03

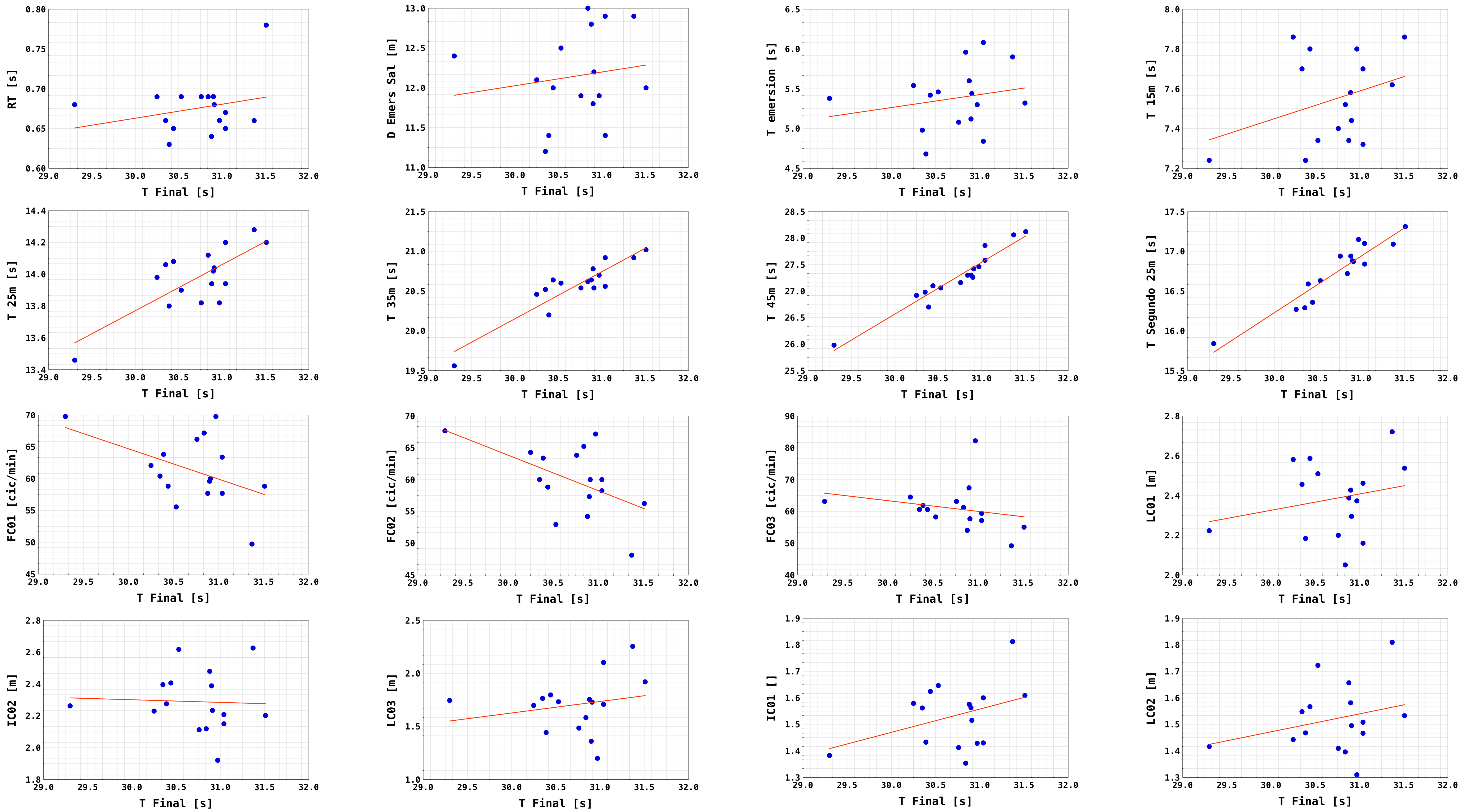

LEN European Aquatics Championships [BUDAPEST, Hungria] 17/05/2021 - 23/05/2021

50 Backstroke Female

						LANE	Pos.	RT	T_entr	T_emer	t15	t25	t35	t45	T50	2nd 25	F01	F02	F03	LC1	LC2	LC3	Flight_T	T_Underw_01	D_Underw	Speed Underw
F	Backstroke	Espalda	50	Final	Final	5	1	0,49	0,58	5,68	6,56	12,40	18,22	24,22	27,36	14,96	58,63	58,44	68,97	1,75	1,74	1,25	0,09	5,10	11,00	2,16
F	Backstroke	Espalda	50	Final	Final	4	2	0,59	0,65	6,10	7,10	12,88	18,60	24,50	27,46	14,58	56,25	53,25	54,55	1,85	1,94	1,67	0,06	5,45	10,80	1,98
F	Backstroke	Espalda	50	Final	Final	6	3	0,58	0,70	5,96	6,66	12,57	18,52	24,58	27,74	15,17	54,88	54,88	54,55	1,85	1,82	1,57	0,12	5,26	11,40	2,17
F	Backstroke	Espalda	50	Final	Final	2	?	0,56	0,67	6,78	6,66	12,55	18,54	24,86	27,81	15,26	55,38	53,10	53,57	1,84	1,84	1,71	0,11	6,11	12,70	2,08
F	Backstroke	Espalda	50	Final	Final	1	4	0,57	0,64	6,38	7,01	12,84	18,79	24,86	27,85	15,01	48,13	46,51	48,00	2,14	2,15	1,88	0,07	5,74	11,60	2,02
F	Backstroke	Espalda	50	Final	Final	3	5	0,65	0,71	6,44	7,22	13,06	18,92	24,90	27,90	14,84	53,25	52,02	50,42	1,93	1,95	1,79	0,06	5,73	11,30	1,97
F	Backstroke	Espalda	50	Final	Final	8	6	0,59	0,74	5,78	6,78	12,71	18,74	24,86	28,01	15,30	53,89	50,85	47,62	1,88	1,94	1,80	0,15	5,04	10,75	2,13
F	Backstroke	Espalda	50	Final	Final	7	7	0,56	0,80	6,24	7,06	12,90	18,78	25,04	28,16	15,26	54,88	52,48	49,59	1,87	1,88	1,75	0,24	5,44	11,10	2,04
MEANS								0,57	0,69	6,17	6,88	12,74	18,64	24,73	27,79	15,05	54,41	52,69	53,41	1,89	1,91	1,68	0,11	5,48	11,33	2,07

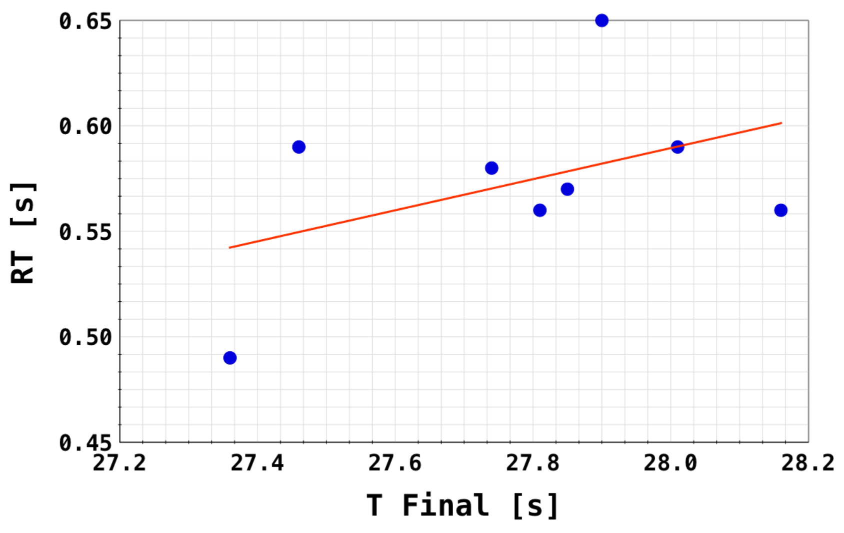

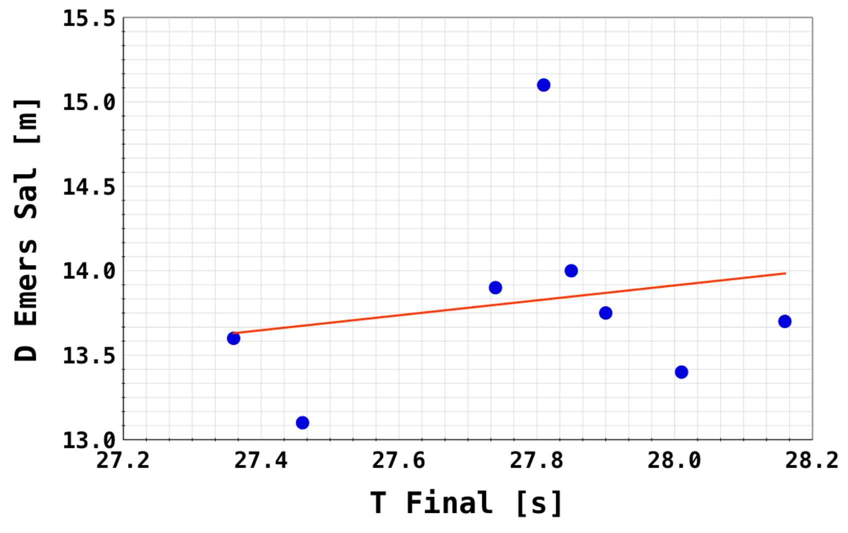

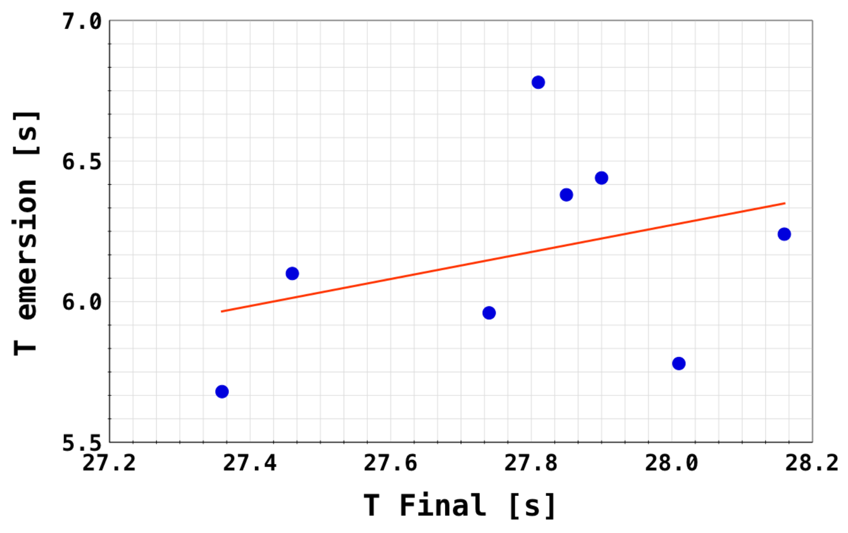

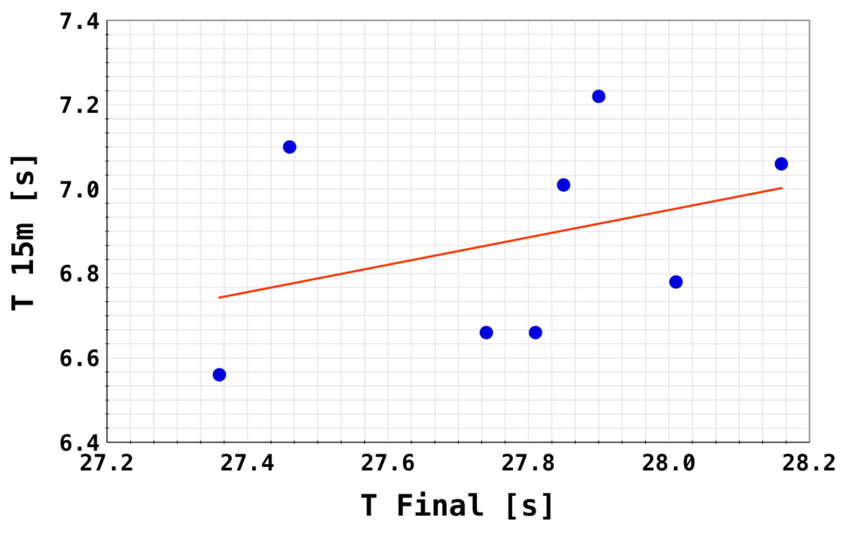

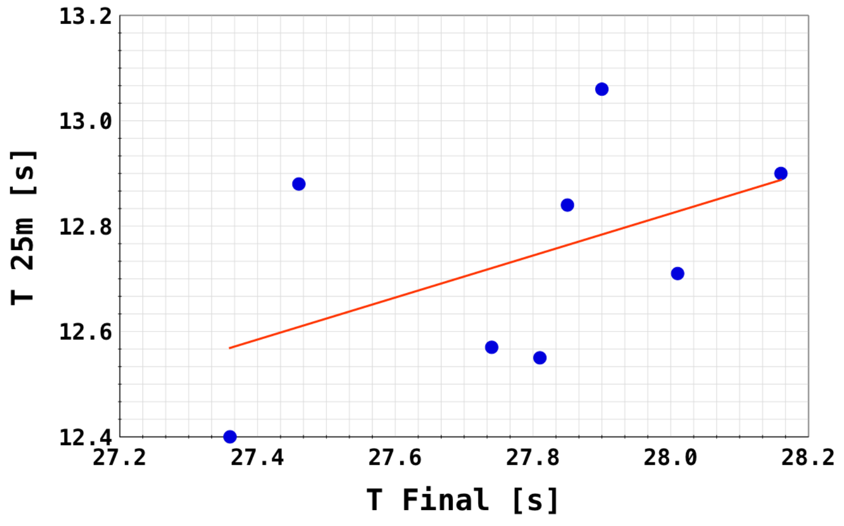

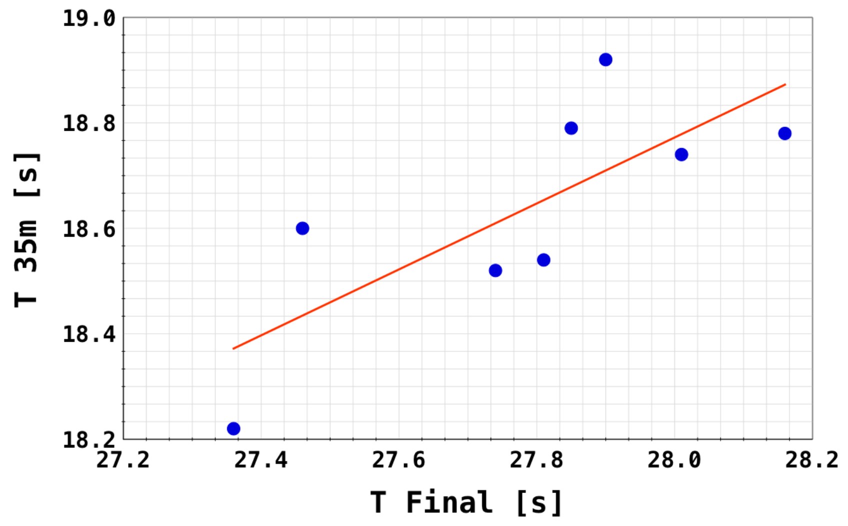

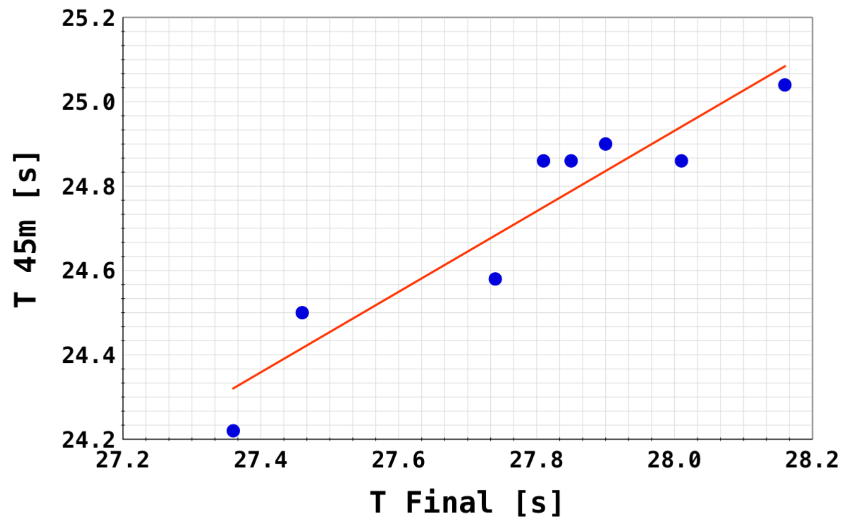

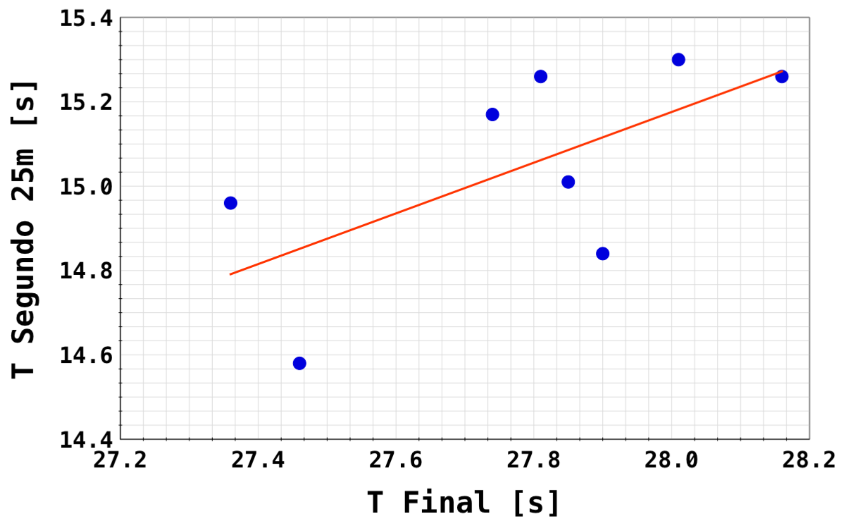

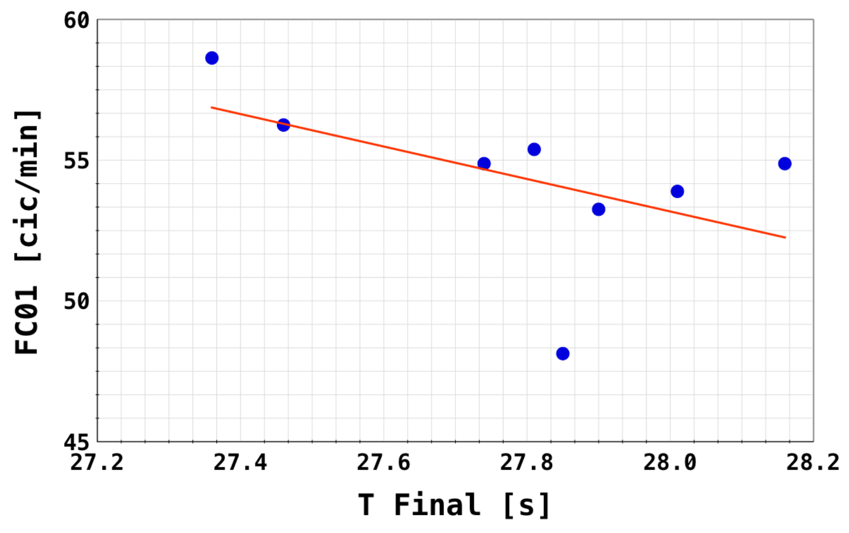

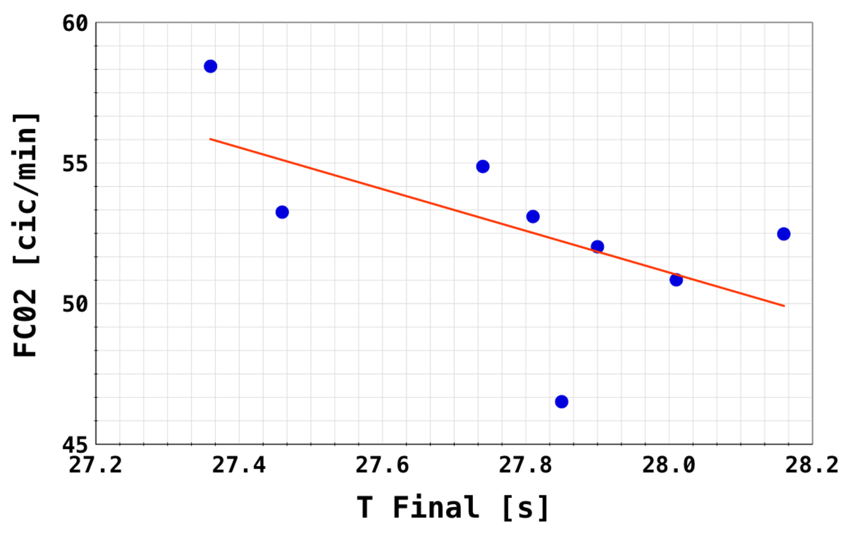

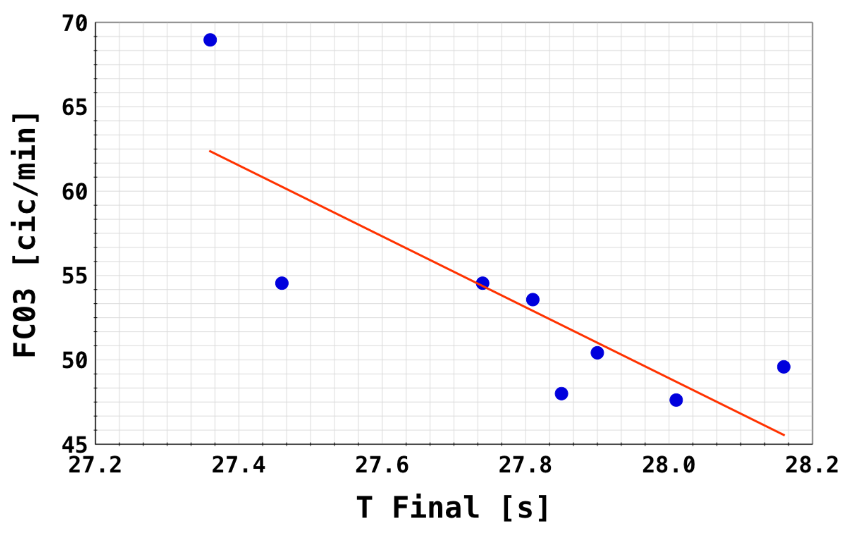

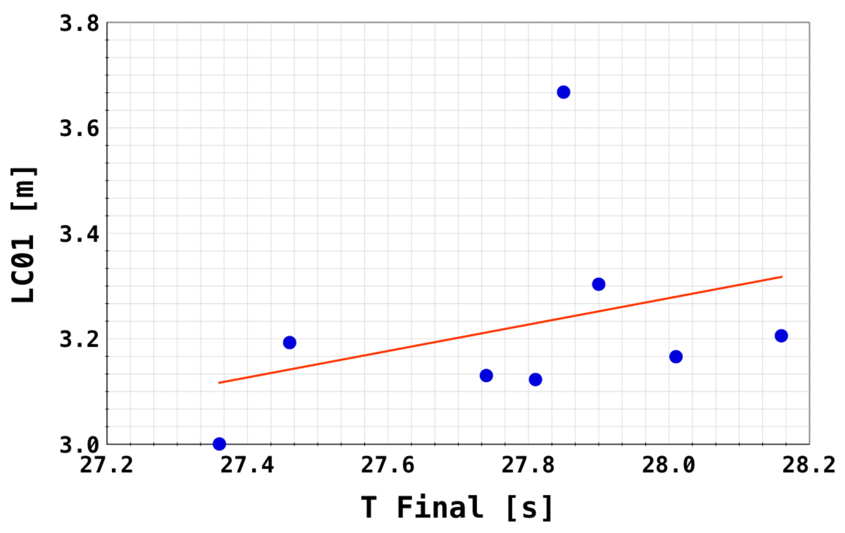

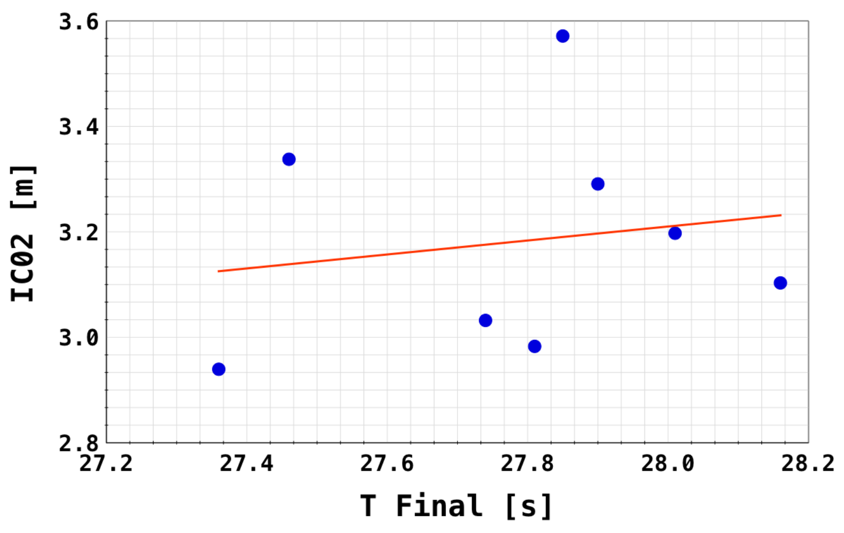

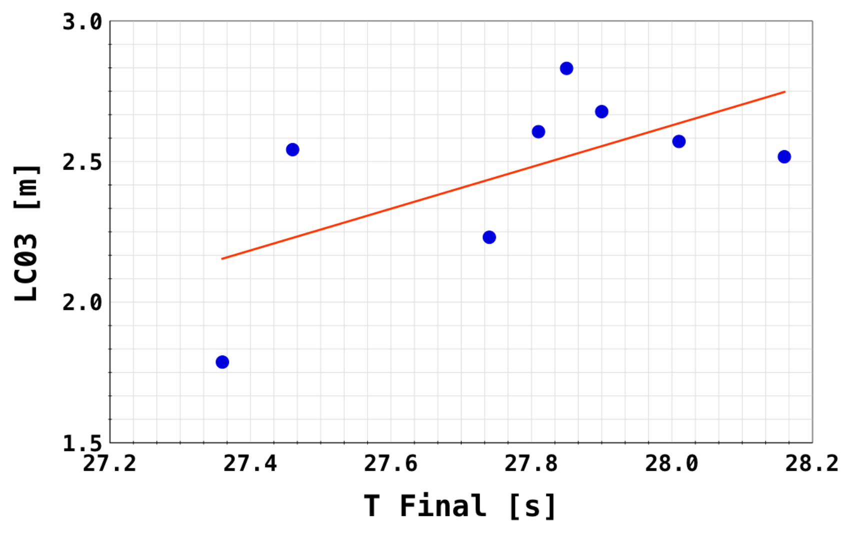

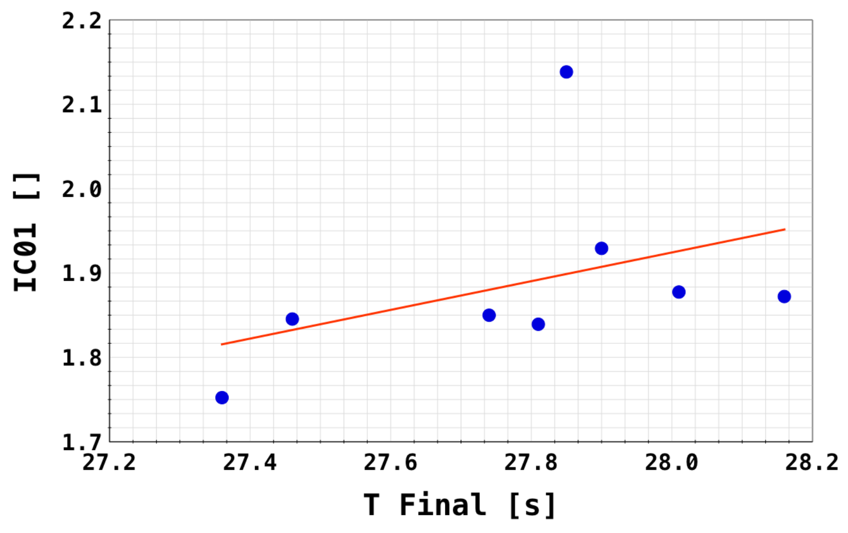

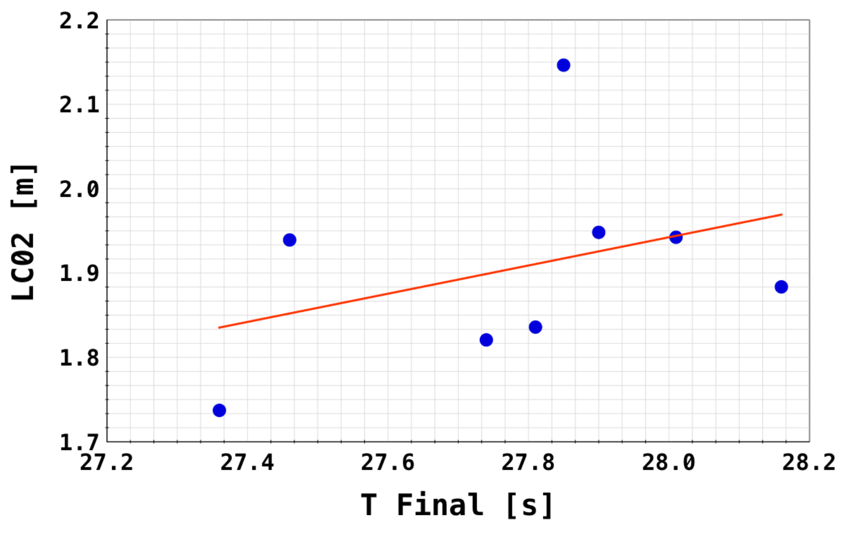

LEN European Aquatics Championships [BUDAPEST, Hungria] 17/05/2021 - 23/05/2021

50 Backstroke Female

						LANE	Pos.	RT	T_entr	T_emer	t15	t25	t35	t45	T50	2nd 25	F01	F02	F03	LC1	LC2	LC3	Flight_T	T_Underw_01	D_Underw	Speed Underw
F	Backstroke	Espalda	50	Heat 6 of 6	Heat	6	1	0,56	0,64	6,24	6,88	12,66	18,40	24,34	27,29	14,63	53,25	52,94	54,55	1,95	1,94	1,68	0,08	5,60	11,40	2,04
F	Backstroke	Espalda	50	Heat 6 of 6	Heat	4	2	0,49	0,60	5,98	6,68	12,58	18,54	24,66	27,60	15,02	56,96	56,96	56,07	1,79	1,74	1,64	0,11	5,38	11,60	2,16
F	Backstroke	Espalda	50	Heat 4 of 6	Heat	4	1	0,61	0,72	6,12	6,62	12,66	18,66	24,80	27,84	15,18	54,22	54,38	54,05	1,83	1,82	1,64	0,11	5,40	11,60	2,15
F	Backstroke	Espalda	50	Heat 5 of 6	Heat	4	1	0,70	0,74	6,72	7,25	13,06	18,90	24,82	27,86	14,80	53,41	52,02	54,55	1,93	1,96	1,63	0,04	5,98	11,70	1,96
F	Backstroke	Espalda	50	Heat 6 of 6	Heat	3	3	0,56	0,72	6,48	7,00	12,82	18,92	25,01	27,97	15,15	53,89	50,85	50,85	1,91	1,94	1,79	0,16	5,76	11,70	2,03
F	Backstroke	Espalda	50	Heat 6 of 6	Heat	5	4	0,58	0,72	6,52	6,90	12,82	18,86	24,98	28,02	15,20	54,22	52,02	52,17	1,87	1,90	1,70	0,14	5,80	11,90	2,05
F	Backstroke	Espalda	50	Heat 4 of 6	Heat	3	2	0,59	0,66	6,68	6,68	12,70	18,72	25,04	28,05	15,35	58,06	55,90	58,25	1,72	1,74	1,54	0,07	6,02	12,80	2,13
F	Backstroke	Espalda	50	Heat 6 of 6	Heat	7	5	0,60	0,68	5,96	7,22	13,10	19,04	25,04	28,05	14,95	54,88	52,94	52,63	1,86	1,90	1,70	0,08	5,28	10,60	2,01
F	Backstroke	Espalda	50	Heat 5 of 6	Heat	2	2	0,57	0,66	6,46	7,06	12,98	18,92	25,04	28,11	15,13	48,91	46,88	44,78	2,07	2,12	1,96	0,09	5,80	11,75	2,03
F	Backstroke	Espalda	50	Heat 5 of 6	Heat	6	3	0,60	0,64	5,92	7,22	13,12	19,08	25,24	28,27	15,15	58,44	56,78	55,56	1,74	1,74	1,60	0,04	5,28	10,60	2,01
F	Backstroke	Espalda	50	Heat 5 of 6	Heat	5	4	0,57	0,68	6,28	7,02	12,90	18,98	25,22	28,28	15,38	59,60	55,21	53,10	1,71	1,76	1,66	0,11	5,60	11,50	2,05
F	Backstroke	Espalda	50	Heat 4 of 6	Heat	5	3	0,55	0,62	6,36	6,88	12,90	19,06	25,24	28,30	15,40	56,60	55,56	53,57	1,76	1,75	1,65	0,07	5,74	11,80	2,06
F	Backstroke	Espalda	50	Heat 6 of 6	Heat	2	6	0,62	0,76	6,08	7,26	13,26	19,28	25,42	28,31	15,05	54,55	54,22	52,63	1,83	1,82	1,78	0,14	5,32	10,40	1,95
F	Backstroke	Espalda	50	Heat 4 of 6	Heat	2	4	0,62	0,68	6,68	7,04	13,06	19,12	25,26	28,33	15,27	51,43	49,18	49,59	1,94	2,00	1,77	0,06	6,00	12,10	2,02
F	Backstroke	Espalda	50	Heat 4 of 6	Heat	7	5	0,55	0,68	6,52	7,12	13,02	19,08	25,32	28,39	15,37	55,90	53,89	55,05	1,82	1,81	1,60	0,13	5,84	11,60	1,99
F	Backstroke	Espalda	50	Heat 5 of 6	Heat	3	5	0,58	0,72	6,08	7,00	12,96	19,16	25,34	28,40	15,44	53,25	50,00	47,43	1,89	1,94	1,86	0,14	5,36	10,90	2,03
				MEANS				0,58	0,68	6,32	6,99	12,91	18,92	25,05	28,07	15,15	54,85	53,11	52,80	1,85	1,87	1,70	0,10	5,64	11,50	2,04

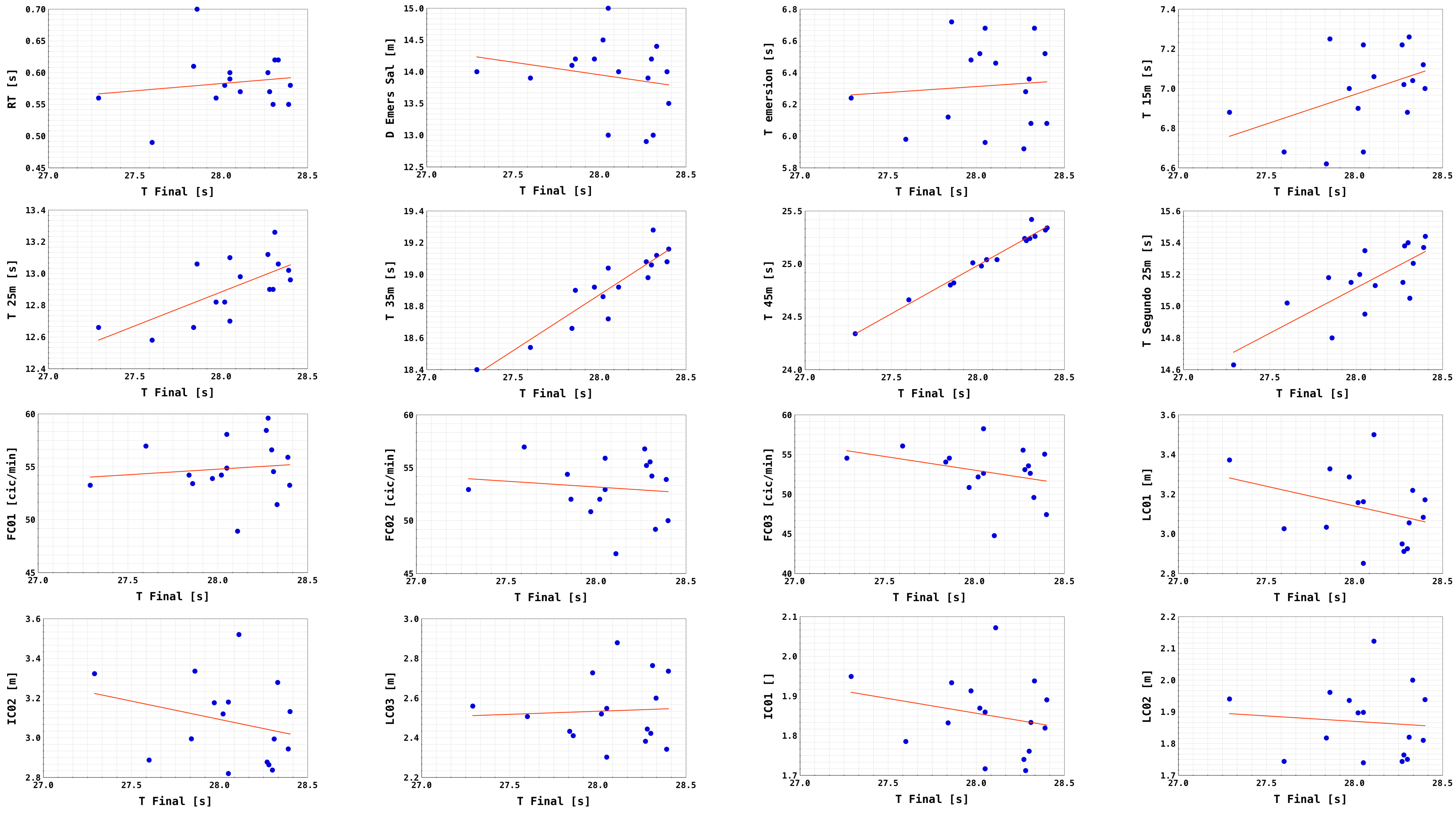

LEN European Aquatics Championships [BUDAPEST, Hungria] 17/05/2021 - 23/05/2021

50 Backstroke Female

						LANE	Pos.	RT	T_entr	T_emer	t15	t25	t35	t45	T50	2nd 25	F01	F02	F03	LC1	LC2	LC3	Flight_T	T_Underw_01	D_Underw	Speed Underw
F	Backstroke	Espalda	50	Semifinal 2	SF	4	1	0,55	0,63	6,30	6,82	12,60	18,38	24,22	27,19	14,59	54,55	53,25	54,55	1,90	1,94	1,67	0,08	5,67	11,90	2,10
F	Backstroke	Espalda	50	Semifinal 1	SF	4	1	0,49	0,56	5,82	6,38	12,22	18,16	24,18	27,22	15,00	57,69	58,44	57,69	1,78	1,72	1,54	0,07	5,26	11,70	2,22
F	Backstroke	Espalda	50	Semifinal 1	SF	5	2	0,65	0,72	6,52	7,17	12,96	18,76	24,74	27,69	14,73	52,63	51,14	50,42	1,97	1,99	1,82	0,07	5,80	11,45	1,97
F	Backstroke	Espalda	50	Semifinal 2	SF	5	2	0,60	0,72	5,98	6,62	12,56	18,54	24,64	27,69	15,13	54,71	54,38	54,55	1,85	1,83	1,62	0,12	5,26	11,35	2,16
F	Backstroke	Espalda	50	Semifinal 1	SF	3	3	0,58	0,70	6,56	6,72	12,56	18,60	24,68	27,81	15,25	54,71	51,72	49,59	1,88	1,91	1,74	0,12	5,86	12,35	2,11
F	Backstroke	Espalda	50	Semifinal 2	SF	3	3	0,57	0,76	6,54	6,98	12,82	18,77	24,86	27,93	15,11	53,73	51,87	50,85	1,91	1,92	1,73	0,19	5,78	11,80	2,04
F	Backstroke	Espalda	50	Semifinal 2	SF	2	4	0,58	0,66	6,54	7,02	12,87	18,86	24,90	27,98	15,11	48,39	46,63	47,62	2,12	2,14	1,84	0,08	5,88	11,70	1,99
F	Backstroke	Espalda	50	Semifinal 1	SF	8	4	0,58	0,72	5,64	6,76	12,70	18,72	24,88	28,01	15,31	53,89	53,25	48,00	1,87	1,85	1,80	0,14	4,92	10,45	2,12
F	Backstroke	Espalda	50	Semifinal 2	SF	6	5	0,57	0,66	6,62	6,62	12,52	18,64	24,94	28,01	15,49	58,44	56,96	55,05	1,74	1,70	1,60	0,09	5,96	12,60	2,11
F	Backstroke	Espalda	50	Semifinal 2	SF	1	6	0,60	0,76	6,12	7,12	13,12	19,12	25,12	28,04	14,92	55,56	55,21	53,10	1,80	1,81	1,74	0,16	5,36	10,70	2,00
F	Backstroke	Espalda	50	Semifinal 1	SF	6	5	0,56	0,66	6,24	6,98	12,92	18,92	25,04	28,06	15,14	55,38	53,89	57,69	1,82	1,84	1,55	0,10	5,58	11,50	2,06
F	Backstroke	Espalda	50	Semifinal 2	SF	7	7	0,56	0,68	6,26	6,94	12,86	18,88	25,14	28,24	15,38	56,96	54,88	52,17	1,78	1,78	1,67	0,12	5,58	11,45	2,05
F	Backstroke	Espalda	50	Semifinal 1	SF	2	6	0,61	0,66	6,22	7,22	13,02	19,04	25,18	28,30	15,28	59,21	56,96	57,69	1,75	1,73	1,50	0,05	5,56	11,00	1,98
F	Backstroke	Espalda	50	Semifinal 2	SF	8	8	0,53	0,68	6,08	7,16	13,06	19,16	25,38	28,46	15,40	56,60	53,25	50,85	1,80	1,83	1,72	0,15	5,40	10,90	2,02
F	Backstroke	Espalda	50	Semifinal 1	SF	7	7	0,57	0,62	6,24	6,98	13,04	19,16	25,38	28,52	15,48	56,60	56,25	54,55	1,75	1,73	1,58	0,05	5,62	11,60	2,06
F	Backstroke	Espalda	50	Semifinal 1	SF	1	8	0,61	0,70	6,40	7,28	13,40	19,70	26,02	29,16	15,76	50,85	47,37	48,00	1,93	2,01	1,79	0,09	5,70	11,30	1,98
				MEANS				0,58	0,68	6,26	6,92	12,83	18,84	24,96	28,02	15,19	54,99	53,47	52,65	1,85	1,86	1,68	0,11	5,57	11,48	2,06

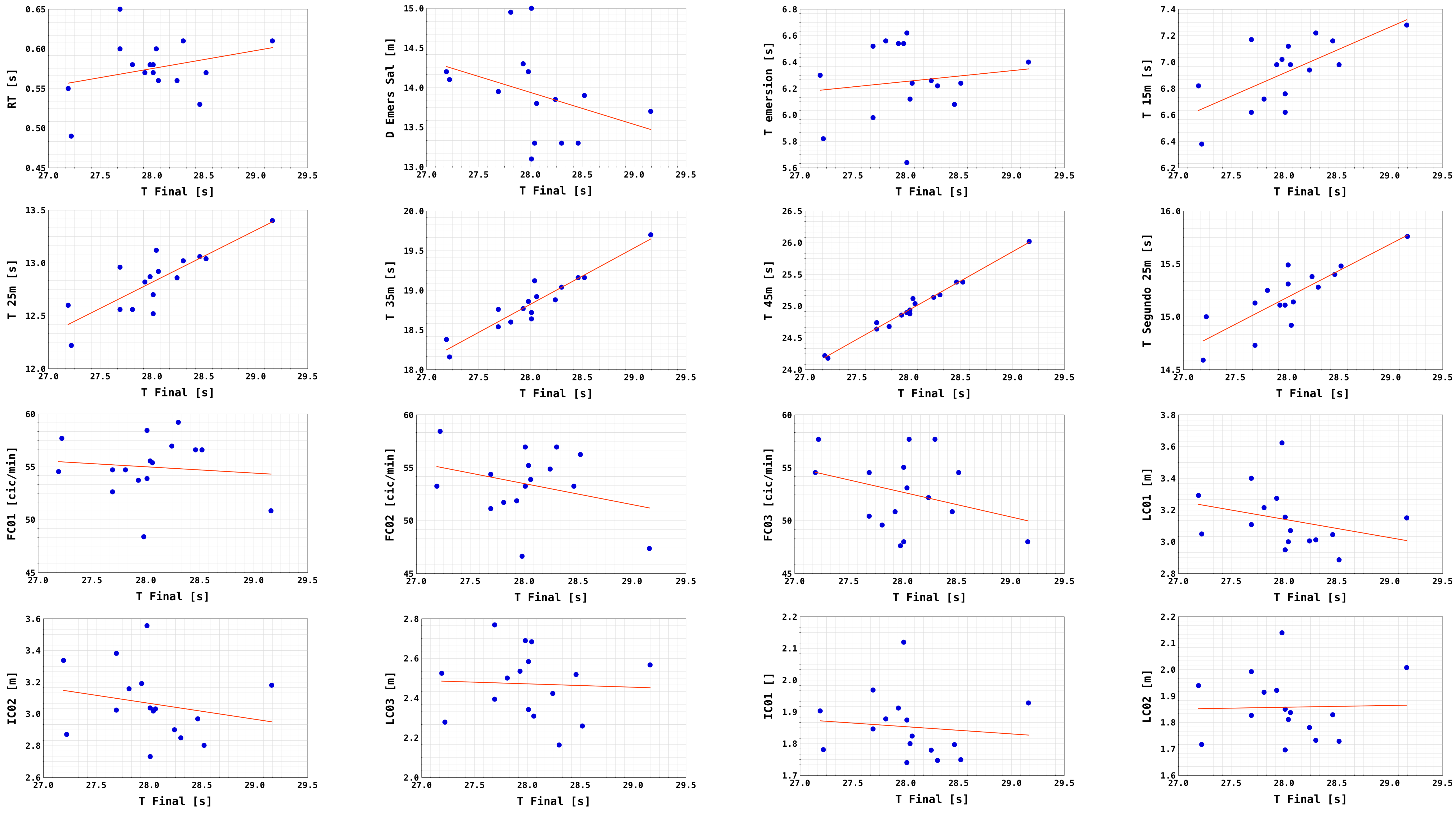

LEN European Aquatics Championships [BUDAPEST, Hungria] 17/05/2021 - 23/05/2021

50 Freestyle Female

						LANE	Pos.	RT	T_entr	T_emer	t15	t25	t35	t45	T50	2nd 25	F01	F02	F03	LC1	LC2	LC3	Flight_T	T_Underw_01	D_Underw	Speed Underw
F	Freestyle	Libre	50	Final	Final	5	1	0,64	0,84	4,38	5,64	10,82	16,06	21,30	23,97	13,15	60,40	60,40	58,25	1,92	1,90	1,74	0,20	3,54	9,50	2,68
F	Freestyle	Libre	50	Final	Final	4	2	0,64	0,97	4,58	6,20	11,12	16,30	21,64	24,17	13,05	63,38	60,81	57,14	1,92	1,88	1,87	0,33	3,61	9,40	2,60
F	Freestyle	Libre	50	Final	Final	3	2	0,67	0,88	3,37	5,95	10,95	16,23	21,55	24,17	13,22	67,16	61,22	57,97	1,79	1,85	1,78	0,21	2,49	7,20	2,89
F	Freestyle	Libre	50	Final	Final	6	4	0,65	0,89	4,89	6,07	11,15	16,31	21,57	24,29	13,14	67,67	60,00	57,69	1,75	1,92	1,72	0,24	4,00	9,80	2,45
F	Freestyle	Libre	50	Final	Final	2	5	0,70	0,84	3,92	6,00	11,00	16,32	21,64	24,32	13,32	59,21	58,44	58,25	2,03	1,93	1,73	0,14	3,08	8,30	2,69
F	Freestyle	Libre	50	Final	Final	1	6	0,67	0,95	4,76	6,11	11,16	16,48	21,93	24,51	13,35	60,00	56,60	52,86	1,98	1,97	1,98	0,28	3,81	9,70	2,55
F	Freestyle	Libre	50	Final	Final	7	7	0,68	1,00	4,80	6,00	11,18	16,38	21,92	24,65	13,47	58,06	58,06	51,28	1,99	1,92	1,93	0,32	3,80	9,20	2,42
F	Freestyle	Libre	50	Final	Final	8	8	0,62	0,92	4,16	6,18	11,32	16,64	22,14	24,67	13,35	60,40	52,63	51,28	1,93	2,11	2,08	0,30	3,24	8,20	2,53
MEANS								0,66	0,91	4,36	6,02	11,09	16,34	21,71	24,34	13,26	62,04	58,52	55,59	1,91	1,93	1,85	0,25	3,45	8,91	2,60

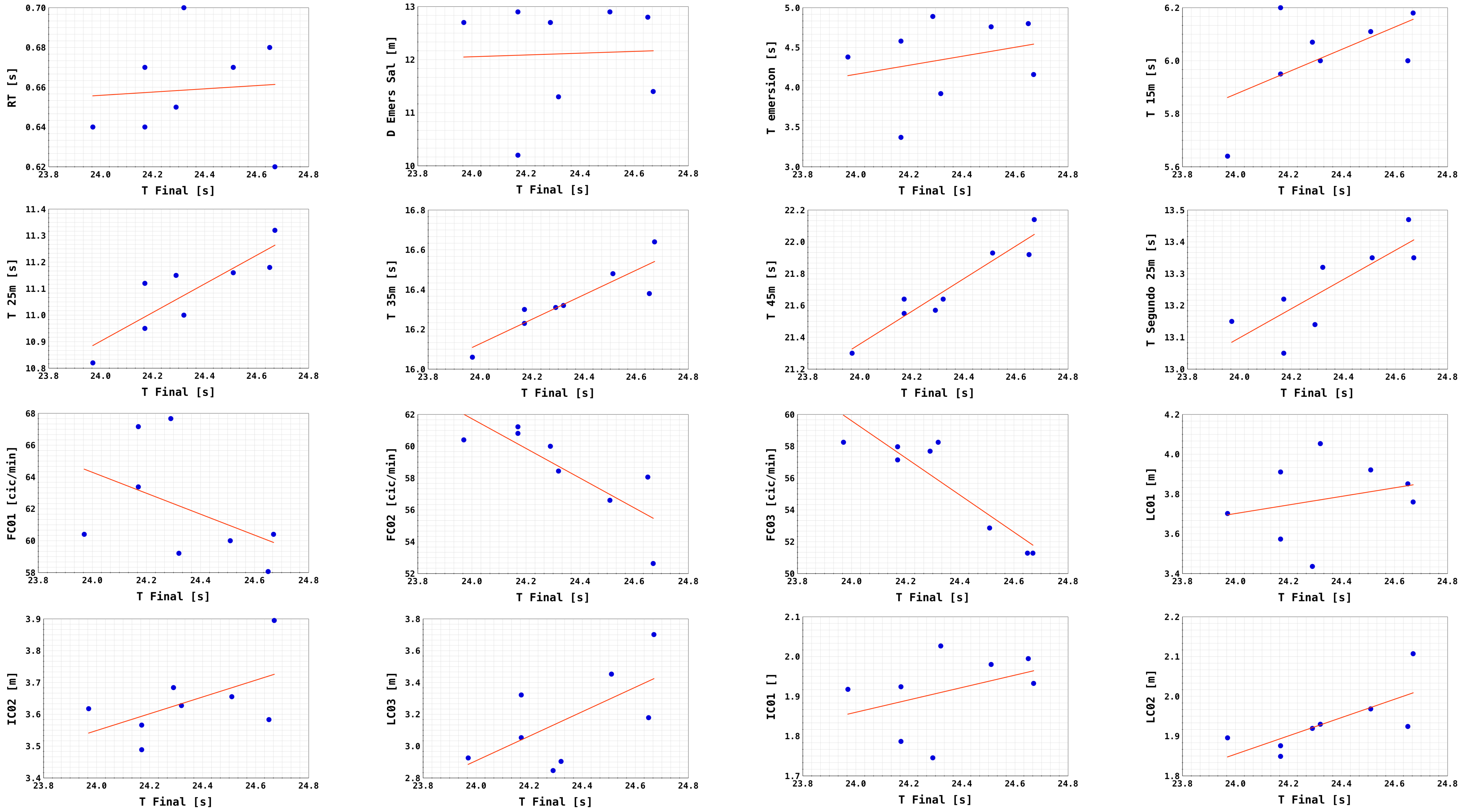

LEN European Aquatics Championships [BUDAPEST, Hungria] 17/05/2021 - 23/05/2021

50 Freestyle Female

						LANE	Pos.	RT	T_entr	T_emer	t15	t25	t35	t45	T50	2nd 25	F01	F02	F03	LC1	LC2	LC3	Flight_T	T_Underw_01	D_Underw	Speed Underw
F	Freestyle	Libre	50	Heat 6 of 7	Heat	4	1	0,67	0,90	4,58	5,74	11,06	16,32	21,62	24,24	13,18	61,64	59,21	57,14	1,83	1,92	1,80	0,23	3,68	9,87	2,68
F	Freestyle	Libre	50	Heat 7 of 7	Heat	4	1	0,67	1,02	4,32	6,24	11,32	16,58	21,82	24,42	13,10	61,64	56,60	56,60	1,92	2,02	1,83	0,35	3,30	8,17	2,48
F	Freestyle	Libre	50	Heat 5 of 7	Heat	4	1	0,63	0,94	5,62	6,26	11,34	16,56	21,90	24,47	13,13	65,22	58,44	56,60	1,81	1,94	1,86	0,31	4,68	10,56	2,26
F	Freestyle	Libre	50	Heat 6 of 7	Heat	5	2	0,72	0,92	4,26	6,12	11,30	16,58	21,92	24,51	13,21	59,60	56,96	55,56	1,94	1,98	1,88	0,20	3,34	8,91	2,67
F	Freestyle	Libre	50	Heat 6 of 7	Heat	3	2	0,69	0,92	3,36	6,10	11,28	16,62	21,96	24,51	13,23	64,29	60,00	58,82	1,80	1,87	1,80	0,23	2,44	6,30	2,58
F	Freestyle	Libre	50	Heat 7 of 7	Heat	5	2	0,67	1,02	4,68	6,12	11,38	16,66	22,06	24,77	13,39	54,55	55,90	53,10	2,09	2,01	1,88	0,35	3,66	8,86	2,42
F	Freestyle	Libre	50	Heat 5 of 7	Heat	5	2	0,68	0,98	4,64	6,12	11,36	16,66	22,10	24,78	13,42	59,21	55,21	54,05	1,93	2,02	1,86	0,30	3,66	9,35	2,55
F	Freestyle	Libre	50	Heat 6 of 7	Heat	2	4	0,61	0,94	5,48	5,96	11,30	16,70	22,18	24,86	13,56	66,18	60,40	57,14	1,70	1,83	1,76	0,33	4,54	10,80	2,38
F	Freestyle	Libre	50	Heat 7 of 7	Heat	3	3	0,59	0,96	5,04	6,08	11,36	16,80	22,24	24,89	13,53	61,22	61,22	58,82	1,86	1,80	1,73	0,37	4,08	9,90	2,43
F	Freestyle	Libre	50	Heat 7 of 7	Heat	6	4	0,67	1,04	4,58	6,22	11,50	16,82	22,30	24,92	13,42	60,40	58,44	54,55	1,88	1,90	1,89	0,37	3,54	8,40	2,37
F	Freestyle	Libre	50	Heat 5 of 7	Heat	1	4	0,60	0,90	3,90	6,28	11,56	16,92	22,38	24,93	13,37	60,81	52,94	49,18	1,87	2,09	2,15	0,30	3,00	7,51	2,50
F	Freestyle	Libre	50	Heat 4 of 7	Heat	1	1	0,74	0,98	3,72	6,24	11,46	16,82	22,34	24,99	13,53	60,40	55,90	52,17	1,90	1,97	1,95	0,24	2,74	7,00	2,55
F	Freestyle	Libre	50	Heat 6 of 7	Heat	6	5	0,64	0,98	4,58	6,24	11,54	16,94	22,44	25,01	13,47	61,64	60,00	59,41	1,84	1,83	1,77	0,34	3,60	8,80	2,44
F	Freestyle	Libre	50	Heat 7 of 7	Heat	1	5	0,68	0,88	4,32	6,34	11,70	17,00	22,48	25,07	13,37	56,96	52,02	51,72	1,97	2,14	2,02	0,20	3,44	9,20	2,67
F	Freestyle	Libre	50	Heat 7 of 7	Heat	2	6	0,63	0,90	2,70	6,26	11,52	16,90	22,36	25,09	13,57	62,94	60,81	61,22	1,81	1,82	1,62	0,27	1,80	4,80	2,67
F	Freestyle	Libre	50	Heat 5 of 7	Heat	6	5	0,65	0,92	4,60	6,26	11,50	16,86	22,44	25,22	13,72	68,18	63,83	60,00	1,68	1,72	1,62	0,27	3,68	9,90	2,69
MEANS								0,66	0,95	4,40	6,16	11,41	16,73	22,16	24,79	13,39	61,56	57,99	56,01	1,86	1,93	1,84	0,29	3,45	8,65	2,52

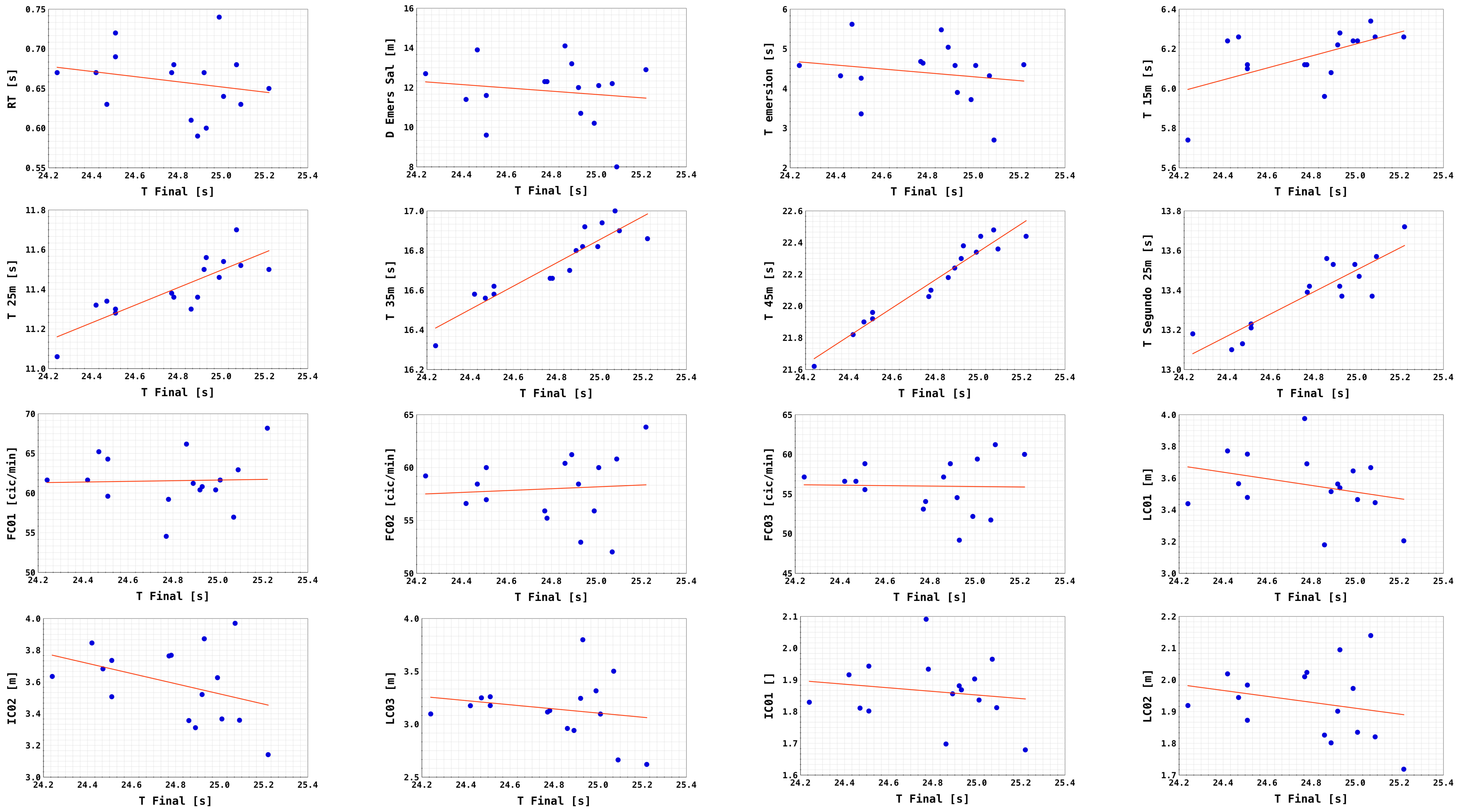

LEN European Aquatics Championships [BUDAPEST, Hungria] 17/05/2021 - 23/05/2021

50 Freestyle Female

						LANE	Pos.	RT	T_entr	T_emer	t15	t25	t35	t45	T50	2nd 25	F01	F02	F03	LC1	LC2	LC3	Flight_T	T_Underw_01	D_Underw	Speed Underw
F	Freestyle	Libre	50	Semifinal 1	SF	4	1	0,62	0,98	4,58	6,08	11,18	16,26	21,52	24,06	12,88	63,83	58,82	57,69	1,84	1,97	1,84	0,36	3,60	9,80	2,72
F	Freestyle	Libre	50	Semifinal 2	SF	4	1	0,65	0,88	4,86	5,76	10,98	16,18	21,46	24,14	13,16	61,64	60,00	58,82	1,86	1,91	1,71	0,23	3,98	10,30	2,59
F	Freestyle	Libre	50	Semifinal 2	SF	3	2	0,65	0,90	3,00	6,06	11,14	16,30	21,62	24,34	13,20	66,67	60,81	58,82	1,77	1,88	1,69	0,25	2,10	4,90	2,33
F	Freestyle	Libre	50	Semifinal 2	SF	5	3	0,63	0,96	5,56	6,18	11,30	16,46	21,72	24,40	13,10	66,67	58,06	54,05	1,76	1,98	1,86	0,33	4,60	10,60	2,30
F	Freestyle	Libre	50	Semifinal 1	SF	5	2	0,68	0,92	4,00	6,04	11,20	16,42	21,76	24,41	13,21	60,40	57,32	56,60	1,93	1,98	1,80	0,24	3,08	8,20	2,66
F	Freestyle	Libre	50	Semifinal 1	SF	3	3	0,70	1,02	4,72	6,02	11,22	16,44	21,76	24,54	13,32	56,96	56,96	55,56	2,03	2,00	1,75	0,32	3,70	9,10	2,46
F	Freestyle	Libre	50	Semifinal 2	SF	6	4	0,68	0,98	4,56	6,12	11,28	16,56	22,00	24,66	13,38	60,40	55,90	52,63	1,93	2,00	1,93	0,30	3,58	8,90	2,49
F	Freestyle	Libre	50	Semifinal 2	SF	7	5	0,64	0,98	4,14	6,18	11,36	16,62	22,12	24,69	13,33	60,40	53,25	51,28	1,92	2,09	2,05	0,34	3,16	7,90	2,50
F	Freestyle	Libre	50	Semifinal 1	SF	2	4	0,68	1,02	4,70	6,14	11,36	16,66	22,12	24,76	13,40	60,81	60,00	54,55	1,89	1,86	1,87	0,34	3,68	8,80	2,39
F	Freestyle	Libre	50	Semifinal 1	SF	6	5	0,62	0,96	5,14	5,90	11,26	16,58	22,02	24,81	13,55	65,69	61,22	56,07	1,70	1,82	1,73	0,34	4,18	10,30	2,46
F	Freestyle	Libre	50	Semifinal 2	SF	1	6	0,62	0,98	4,54	6,16	11,38	16,78	22,18	24,90	13,52	62,94	59,60	125,00	1,83	1,86	0,79	0,36	3,56	8,70	2,44
F	Freestyle	Libre	50	Semifinal 1	SF	1	6	0,70	0,94	4,00	6,28	11,50	16,84	22,40	24,96	13,46	58,06	52,63	55,05	1,98	2,09	1,92	0,24	3,06	7,30	2,39
F	Freestyle	Libre	50	Semifinal 1	SF	7	7	0,69	0,92	3,98	6,20	11,46	16,76	22,32	24,97	13,51	60,40	56,60	101,69	1,89	1,95	1,00	0,23	3,06	7,80	2,55
F	Freestyle	Libre	50	Semifinal 2	SF	2	7	0,58	0,94	4,92	6,02	11,36	16,70	22,30	25,08	13,72	62,50	61,22	60,61	1,80	1,79	1,60	0,36	3,98	9,60	2,41
F	Freestyle	Libre	50	Semifinal 1	SF	8	8	0,63	0,96	5,00	6,32	11,52	16,86	22,28	25,14	13,62	66,67	62,94	59,41	1,73	1,77	1,59	0,33	4,04	9,40	2,33
F	Freestyle	Libre	50	Semifinal 2	SF	8	8	0,65	0,94	2,90	6,30	11,56	16,84	22,38	25,14	13,58	62,94	60,40	58,82	1,81	1,84	1,66	0,29	1,96	5,00	2,55
				MEANS				0,65	0,96	4,41	6,11	11,32	16,58	22,00	24,69	13,37	62,31	58,49	63,54	1,85	1,93	1,67	0,30	3,46	8,54	2,47

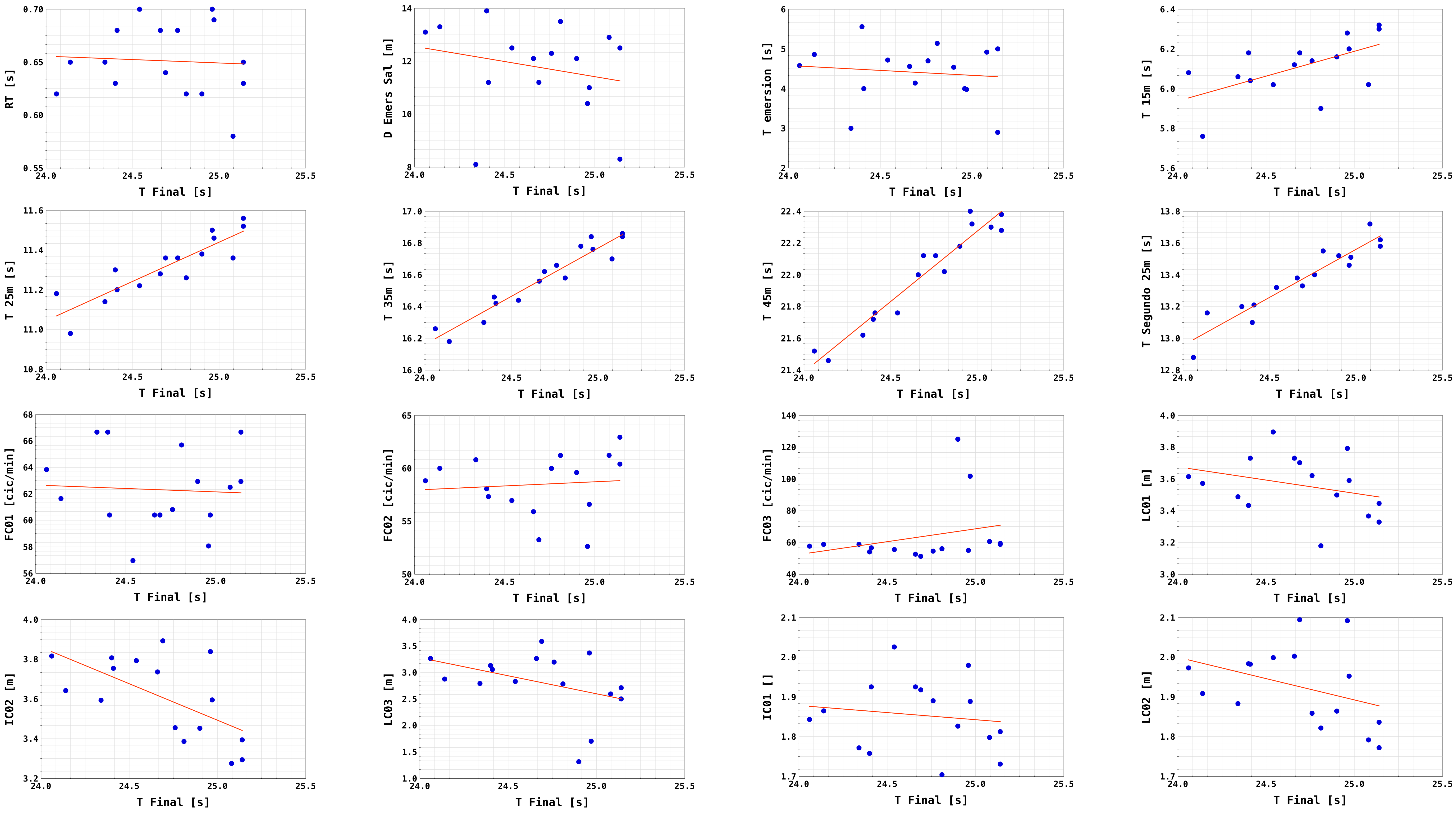

LEN European Aquatics Championships [BUDAPEST, Hungria] 17/05/2021 - 23/05/2021

50 Butterfly Female

						LANE	Pos.	RT	T_entr	T_emer	t15	t25	t35	t45	T50	2nd 25	F01	F02	F03	LC1	LC2	LC3	Flight_T	T_Underw_01	D_Underw	Speed Underw
F	Butterfly	Mariposa	50	Final	Final	3	1	0,64	0,84	5,04	5,74	11,30	16,86	22,46	25,30	14,00	63,38	64,29	63,16	1,70	1,67	1,51	0,20	4,20	10,60	2,52
F	Butterfly	Mariposa	50	Final	Final	4	2	0,58	0,94	5,14	5,86	11,28	16,74	22,42	25,46	14,18	65,22	62,07	62,50	1,70	1,74	1,42	0,36	4,20	10,40	2,48
F	Butterfly	Mariposa	50	Final	Final	5	3	0,68	1,00	5,54	6,08	11,58	17,04	22,72	25,59	14,01	62,94	60,40	63,83	1,73	1,78	1,47	0,32	4,54	10,70	2,36
F	Butterfly	Mariposa	50	Final	Final	1	4	0,63	0,94	5,24	6,14	11,66	17,18	22,94	25,65	13,99	65,69	60,40	57,14	1,65	1,76	1,74	0,31	4,30	10,20	2,37
F	Butterfly	Mariposa	50	Final	Final	2	5	0,70	0,98	5,78	6,08	11,60	17,20	22,92	25,76	14,16	63,38	61,64	61,22	1,71	1,72	1,55	0,28	4,80	11,20	2,33
F	Butterfly	Mariposa	50	Final	Final	8	6	0,72	1,00	5,46	6,14	11,66	17,28	22,96	25,84	14,18	59,60	59,60	57,14	1,82	1,78	1,64	0,28	4,46	10,60	2,38
F	Butterfly	Mariposa	50	Final	Final	7	7	0,67	0,92	5,76	6,06	11,56	17,20	22,98	25,85	14,29	64,29	62,07	61,86	1,70	1,69	1,52	0,25	4,84	11,30	2,33
F	Butterfly	Mariposa	50	Final	Final	6	8	0,71	0,98	5,16	6,20	11,62	17,18	22,96	25,87	14,25	68,70	65,22	63,16	1,61	1,62	1,47	0,27	4,18	10,80	2,58
MEANS								0,67	0,95	5,39	6,04	11,53	17,08	22,80	25,67	14,13	64,15	61,96	61,25	1,70	1,72	1,54	0,28	4,44	10,73	2,42

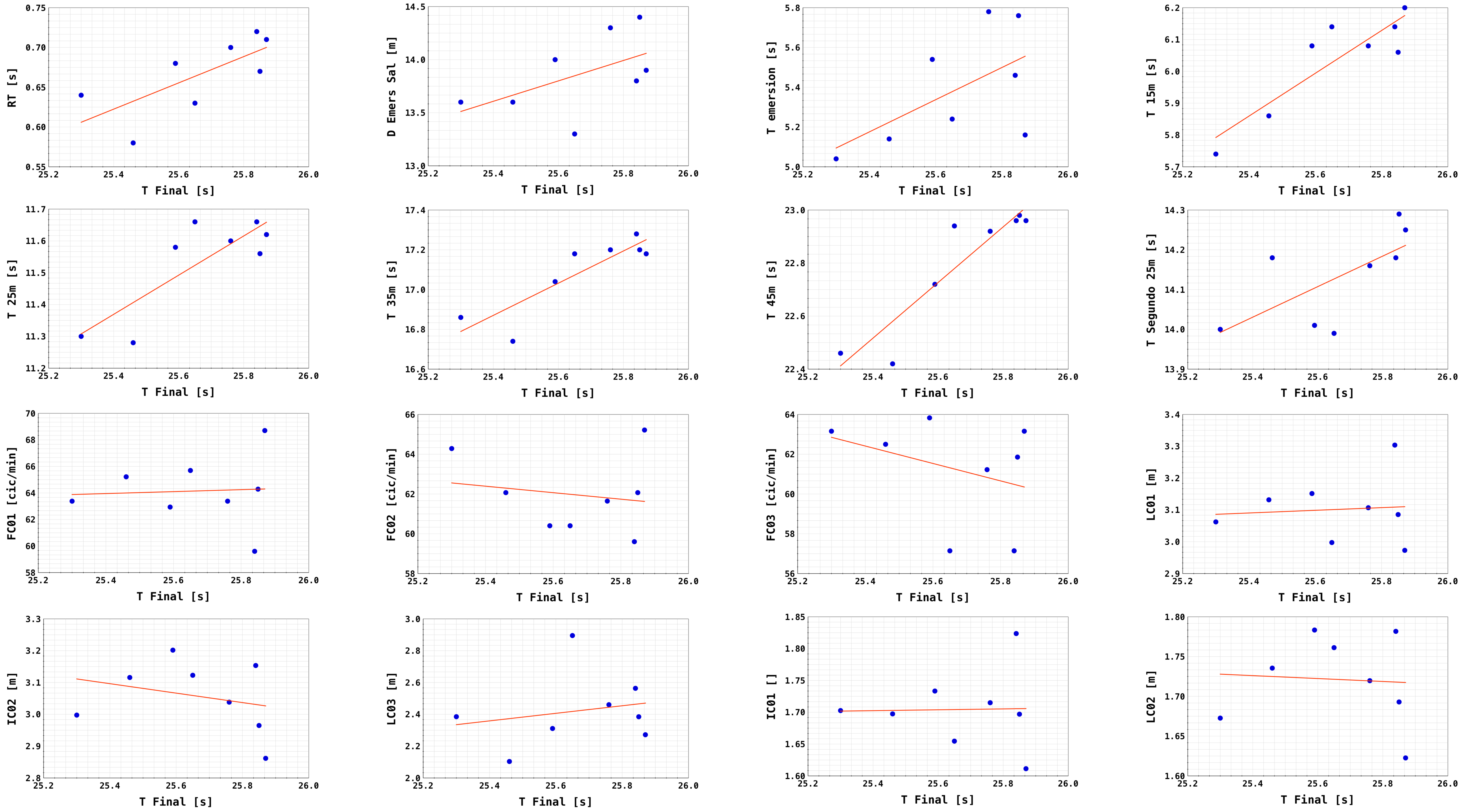

LEN European Aquatics Championships [BUDAPEST, Hungria] 17/05/2021 - 23/05/2021

50 Butterfly Female

						LANE	Pos.	RT	T_entr	T_emer	t15	t25	t35	t45	T50	2nd 25	F01	F02	F03	LC1	LC2	LC3	Flight_T	T_Underw_01	D_Underw	Speed Underw
F	Butterfly	Mariposa	50	Heat 6 of 6	Heat	4	1	0,59	0,96	5,24	5,84	11,26	16,78	22,42	25,30	14,04	64,29	60,00	62,50	1,72	1,79	1,50	0,37	4,28	10,60	2,48
F	Butterfly	Mariposa	50	Heat 5 of 6	Heat	4	1	0,67	0,90	4,88	6,02	11,68	17,36	23,08	25,88	14,20	61,64	61,64	61,86	1,72	1,71	1,56	0,23	3,98	10,00	2,51
F	Butterfly	Mariposa	50	Heat 4 of 6	Heat	5	1	0,67	0,92	5,92	6,08	11,70	17,28	23,10	25,91	14,21	63,38	60,81	63,83	1,68	1,73	1,51	0,25	5,00	11,60	2,32
F	Butterfly	Mariposa	50	Heat 6 of 6	Heat	4	2	0,69	1,00	5,58	6,16	11,64	17,28	23,02	25,96	14,32	62,50	58,06	63,83	1,75	1,82	1,44	0,31	4,58	10,90	2,38
F	Butterfly	Mariposa	50	Heat 5 of 6	Heat	5	2	0,71	0,96	5,38	6,18	11,68	17,24	22,94	25,96	14,28	68,18	63,83	61,22	1,60	1,67	1,46	0,25	4,42	10,40	2,35
F	Butterfly	Mariposa	50	Heat 5 of 6	Heat	1	3	0,70	1,02	5,72	6,10	11,70	17,40	23,22	25,99	14,29	59,21	57,32	56,60	1,81	1,82	1,72	0,32	4,70	11,10	2,36
F	Butterfly	Mariposa	50	Heat 4 of 6	Heat	3	3	0,72	0,94	5,92	6,10	11,74	17,40	23,10	26,11	14,37	62,50	60,40	57,69	1,70	1,75	1,55	0,22	4,98	11,90	2,39
F	Butterfly	Mariposa	50	Heat 5 of 6	Heat	3	4	0,71	1,00	5,28	6,20	11,84	17,52	23,28	26,20	14,36	65,22	64,29	63,16	1,63	1,63	1,46	0,29	4,28	10,00	2,34
F	Butterfly	Mariposa	50	Heat 6 of 6	Heat	6	2	0,66	0,90	5,84	6,18	11,80	17,48	23,28	26,28	14,48	68,70	64,75	63,16	1,55	1,61	1,43	0,24	4,94	11,20	2,27
F	Butterfly	Mariposa	50	Heat 4 of 6	Heat	2	4	0,68	0,92	5,80	6,54	12,08	17,70	23,38	26,31	14,23	66,18	61,22	60,61	1,64	1,73	1,52	0,24	4,88	10,70	2,19
F	Butterfly	Mariposa	50	Heat 6 of 6	Heat	5	3	0,68	0,94	5,32	6,32	11,92	17,62	23,52	26,35	14,43	58,82	54,88	52,63	1,82	1,89	1,81	0,26	4,38	10,10	2,31
F	Butterfly	Mariposa	50	Heat 6 of 6	Heat	3	4	0,61	0,90	5,62	6,16	11,82	17,54	23,34	26,41	14,59	64,75	61,22	57,69	1,64	1,70	1,52	0,29	4,72	10,90	2,31
F	Butterfly	Mariposa	50	Heat 6 of 6	Heat	2	5	0,65	0,94	5,24	6,34	12,06	17,82	23,64	26,46	14,40	65,69	59,21	61,22	1,60	1,75	1,56	0,29	4,30	9,80	2,28
F	Butterfly	Mariposa	50	Heat 5 of 6	Heat	2	6	0,68	0,92	5,00	6,58	12,38	17,96	23,66	26,59	14,21	71,43	66,67	62,50	1,45	1,60	1,47	0,24	4,08	9,70	2,38
F	Butterfly	Mariposa	50	Heat 3 of 6	Heat	5	1	0,66	1,00	5,50	6,38	12,10	17,92	23,66	26,63	14,53	63,38	61,64	61,22	1,66	1,68	1,48	0,34	4,50	10,10	2,24
F	Butterfly	Mariposa	50	Heat 3 of 6	Heat	2	2	0,64	0,94	5,12	6,32	12,06	17,82	23,68	26,65	14,59	61,64	90,00	57,69	1,70	1,15	1,58	0,30	4,18	10,70	2,56
F	Butterfly	Mariposa	50	Heat 6 of 6	Heat	7	7	0,66	1,06	3,50	6,60	12,14	17,72	23,52	26,65	14,51	69,23	64,75	63,16	1,56	1,63	1,37	0,40	2,44	5,80	2,38
MEANS								0,67	0,95	5,34	6,24	11,86	17,52	23,28	26,21	14,36	64,51	62,98	60,62	1,66	1,69	1,53	0,28	4,39	10,32	2,36

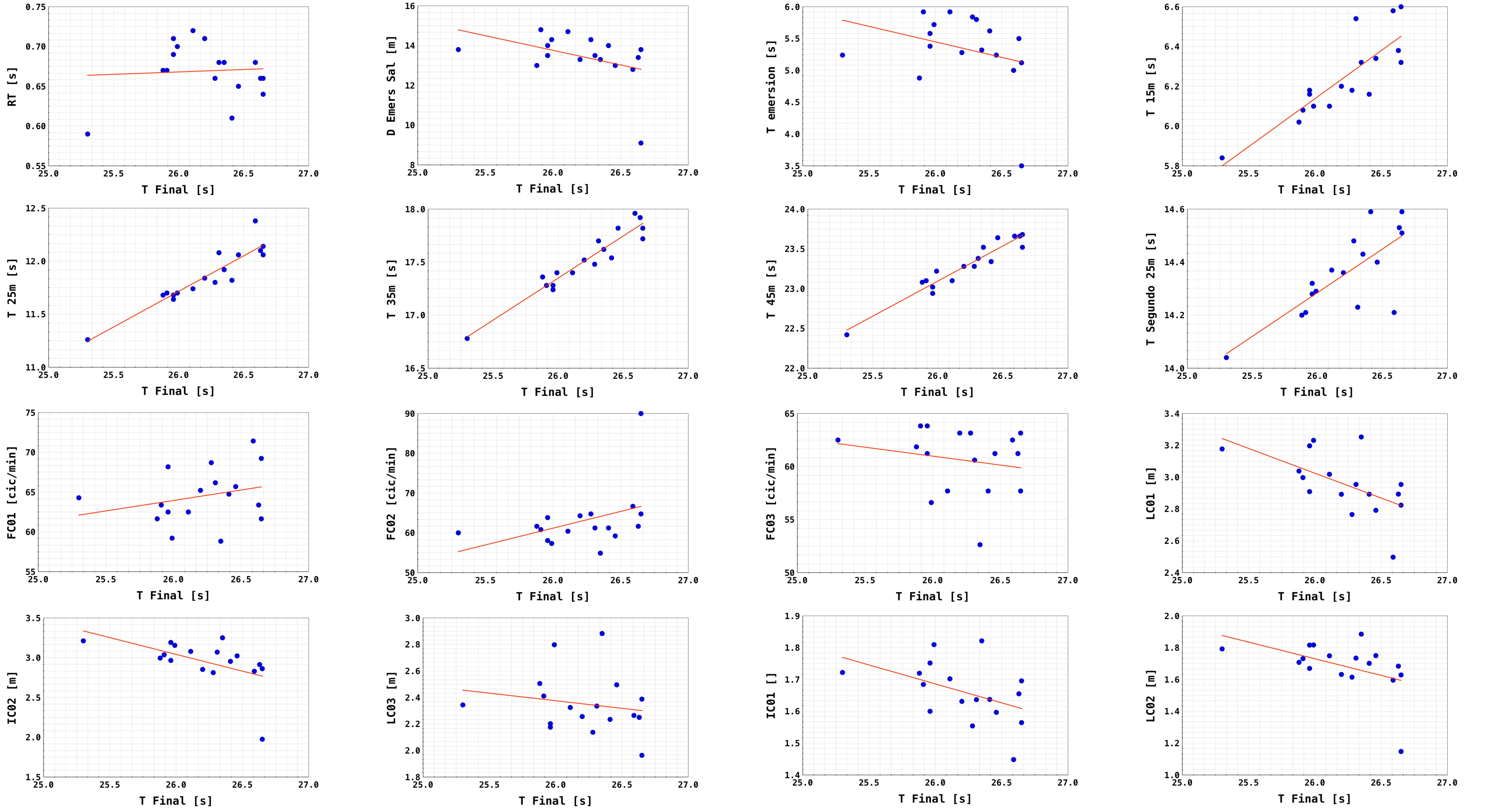

LEN European Aquatics Championships [BUDAPEST, Hungria] 17/05/2021 - 23/05/2021

50 Butterfly Female

						LANE	Pos.	RT	T_entr	T_emer	t15	t25	t35	t45	T50	2nd 25	F01	F02	F03	LC1	LC2	LC3	Flight_T	T_Underw_01	D_Underw	Speed Underw
F	Butterfly	Mariposa	50	Semifinal 2	SF	4	1	0,61	0,98	5,00	5,96	11,40	16,96	22,58	25,53	14,13	65,22	62,50	63,83	1,69	1,72	1,43	0,37	4,02	10,10	2,51
F	Butterfly	Mariposa	50	Semifinal 2	SF	3	2	0,67	1,00	5,90	6,10	11,56	17,06	22,74	25,64	14,08	63,38	58,82	60,61	1,73	1,82	1,54	0,33	4,90	11,60	2,37
F	Butterfly	Mariposa	50	Semifinal 1	SF	4	1	0,67	0,92	4,82	5,94	11,58	17,26	22,96	25,71	14,13	62,07	62,07	62,50	1,71	1,70	1,57	0,25	3,90	9,80	2,51
F	Butterfly	Mariposa	50	Semifinal 1	SF	5	2	0,67	0,94	4,80	6,02	11,48	17,10	22,86	25,75	14,27	68,70	65,69	62,50	1,60	1,61	1,49	0,27	3,86	9,50	2,46
F	Butterfly	Mariposa	50	Semifinal 2	SF	6	3	0,71	1,00	6,16	6,08	11,50	17,06	22,80	25,81	14,31	64,29	61,22	57,14	1,72	1,73	1,57	0,29	5,16	12,10	2,34
F	Butterfly	Mariposa	50	Semifinal 2	SF	5	3	0,67	0,92	5,94	6,08	11,54	17,18	23,00	25,81	14,27	62,94	61,64	60,61	1,75	1,70	1,59	0,25	5,02	11,70	2,33
F	Butterfly	Mariposa	50	Semifinal 2	SF	1	5	0,65	0,94	5,26	6,24	11,72	17,46	23,10	25,93	14,21	66,18	59,60	56,60	1,65	1,77	1,69	0,29	4,32	10,20	2,36
F	Butterfly	Mariposa	50	Semifinal 1	SF	3	3	0,71	1,00	5,54	6,10	11,68	17,30	23,14	26,00	14,32	60,00	58,44	56,60	1,79	1,79	1,67	0,29	4,54	10,60	2,33
F	Butterfly	Mariposa	50	Semifinal 2	SF	7	6	0,66	0,94	5,52	6,38	11,78	17,56	23,22	26,07	14,29	60,40	56,60	58,82	1,84	1,85	1,61	0,28	4,58	10,40	2,27
F	Butterfly	Mariposa	50	Semifinal 1	SF	7	4	0,63	0,94	5,28	6,00	11,64	17,26	23,12	26,14	14,50	67,16	62,50	60,00	1,58	1,67	1,49	0,31	4,34	10,30	2,37
F	Butterfly	Mariposa	50	Semifinal 1	SF	6	5	0,71	0,96	4,78	6,10	11,70	17,36	23,14	26,15	14,45	65,69	63,38	64,52	1,63	1,66	1,39	0,25	3,82	9,20	2,41
F	Butterfly	Mariposa	50	Semifinal 2	SF	2	7	0,65	0,86	5,62	6,10	11,72	17,38	23,14	26,23	14,51	69,77	65,22	61,22	1,53	1,61	1,43	0,21	4,76	11,10	2,33
F	Butterfly	Mariposa	50	Semifinal 1	SF	2	6	0,68	0,88	5,24	6,46	11,94	17,54	23,36	26,27	14,33	68,18	62,07	59,41	1,61	1,69	1,56	0,20	4,36	9,80	2,25
F	Butterfly	Mariposa	50	Semifinal 1	SF	8	7	0,70	1,04	5,76	6,38	12,02	17,68	23,46	26,49	14,47	65,69	61,64	61,22	1,62	1,70	1,46	0,34	4,72	10,80	2,29
F	Butterfly	Mariposa	50	Semifinal 1	SF	1	8	0,70	0,96	5,04	6,54	12,18	17,90	23,64	26,58	14,40	72,58	64,29	60,61	1,47	1,63	1,52	0,26	4,08	10,10	2,48
F	Butterfly	Mariposa	50	Semifinal 2	SF	8	8	0,65	1,00	3,86	6,58	12,02	17,68	23,68	26,69	14,67	68,70	65,69	61,86	1,61	1,57	1,45	0,35	2,86	6,80	2,38
F	Butterfly	Mariposa	50	Semifinal 2	SF	9	9	0,69	0,98	5,56	6,46	12,06	17,86	23,84	26,88	14,82	62,50	59,60	57,14	1,71	1,71	1,55	0,29	4,58	10,20	2,23
						MEANS		0,67	0,96	5,30	6,21	11,74	17,39	23,16	26,10	14,36	65,50	61,82	60,31	1,66	1,70	1,53	0,28	4,34	10,25	2,37

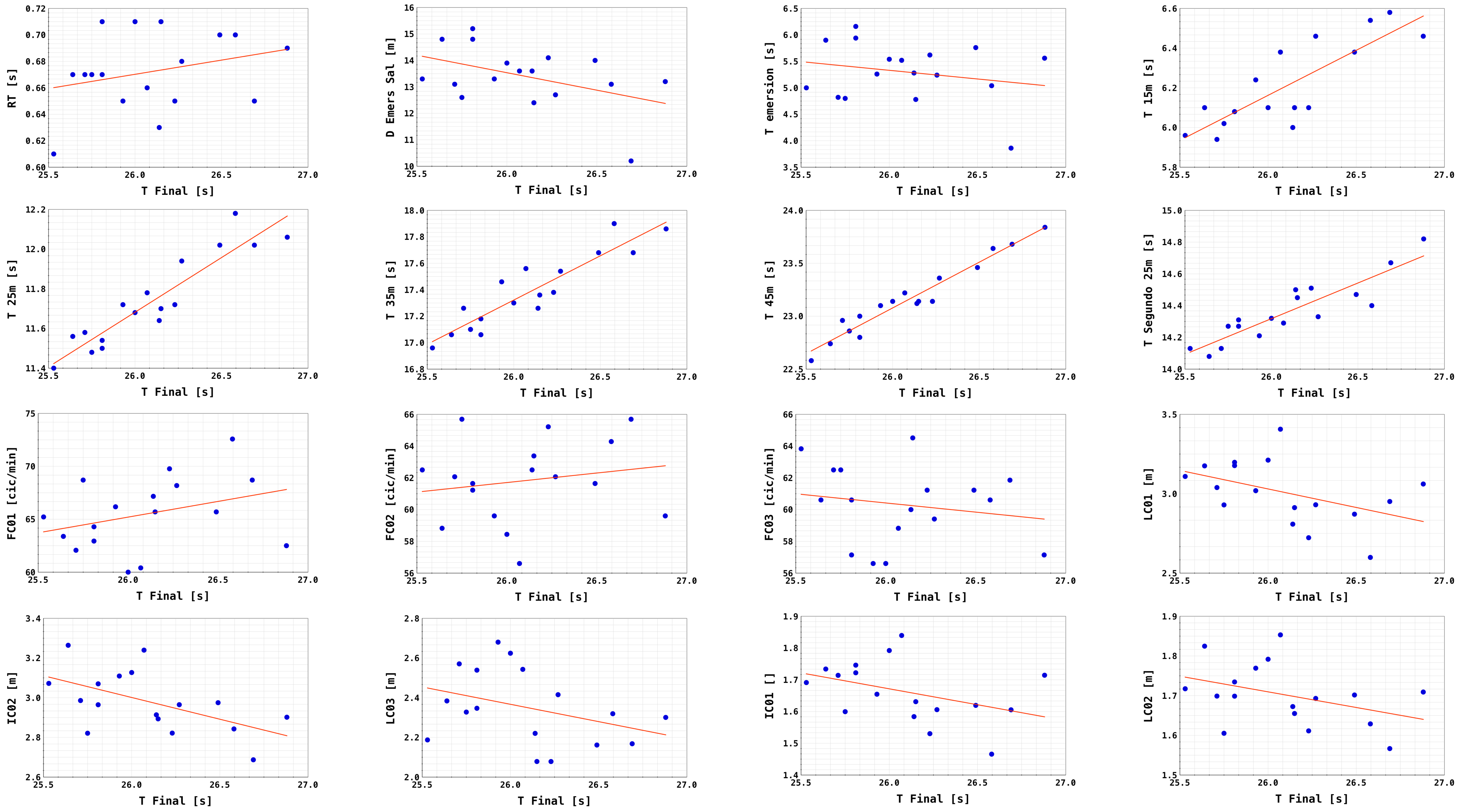

LEN European Aquatics Championships [BUDAPEST, Hungria] 17/05/2021 - 23/05/2021

50 Breaststroke Male

						LANE	Pos.	RT	T_entr	T_emer	t15	t25	t35	t45	T50	2nd 25	F01	F02	F03	LC1	LC2	LC3	Flight_T	T_Underw_01	D_Underw	Speed Underw
M	Breaststroke	Braza	50	Final	Final	4	1	0,62	0,98	5,62	6,18	11,92	17,64	23,44	26,21	14,29	68,18	64,75	67,42	1,53	1,61	1,45	0,36	4,64	10,00	2,16
M	Breaststroke	Braza	50	Final	Final	5	2	0,63	0,94	5,58	6,00	11,78	17,56	23,42	26,55	14,77	62,50	62,50	61,86	1,66	1,65	1,39	0,31	4,64	10,30	2,22
M	Breaststroke	Braza	50	Final	Final	3	3	0,63	1,02	5,42	5,88	11,76	17,60	23,50	26,68	14,92	75,00	72,58	69,77	1,36	1,41	1,22	0,39	4,40	9,50	2,16
M	Breaststroke	Braza	50	Final	Final	6	4	0,71	0,98	5,48	6,30	12,36	18,20	24,12	27,10	14,74	73,77	70,31	68,97	1,34	1,45	1,31	0,27	4,50	9,60	2,13
M	Breaststroke	Braza	50	Final	Final	7	5	0,61	1,02	5,78	6,42	12,28	18,00	23,88	27,13	14,85	65,22	63,83	63,83	1,57	1,62	1,30	0,41	4,76	10,00	2,10
M	Breaststroke	Braza	50	Final	Final	1	5	0,67	0,96	4,74	6,32	12,24	18,16	24,14	27,13	14,89	63,38	63,38	61,22	1,60	1,59	1,47	0,29	3,78	8,20	2,17
M	Breaststroke	Braza	50	Final	Final	8	7	0,65	1,00	5,78	6,00	11,98	18,04	24,28	27,30	15,32	60,81	59,60	54,55	1,65	1,64	1,64	0,35	4,78	11,00	2,30
M	Breaststroke	Braza	50	Final	Final	2	8	0,69	1,00	5,76	6,64	12,60	18,58	24,68	27,54	14,94	69,77	71,43	71,43	1,44	1,39	1,32	0,31	4,76	9,10	1,91
						MEANS		0,65	0,99	5,52	6,22	12,12	17,97	23,93	26,96	14,84	67,33	66,05	64,88	1,52	1,54	1,39	0,34	4,53	9,71	2,14

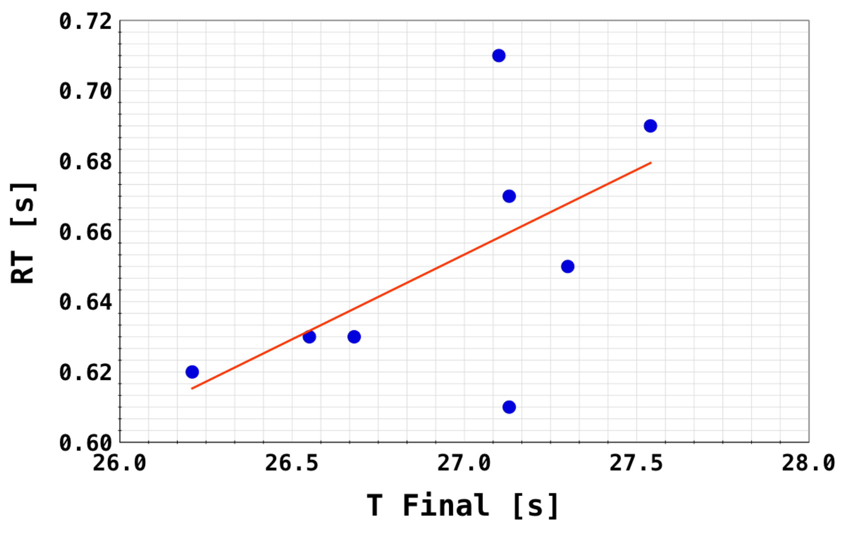

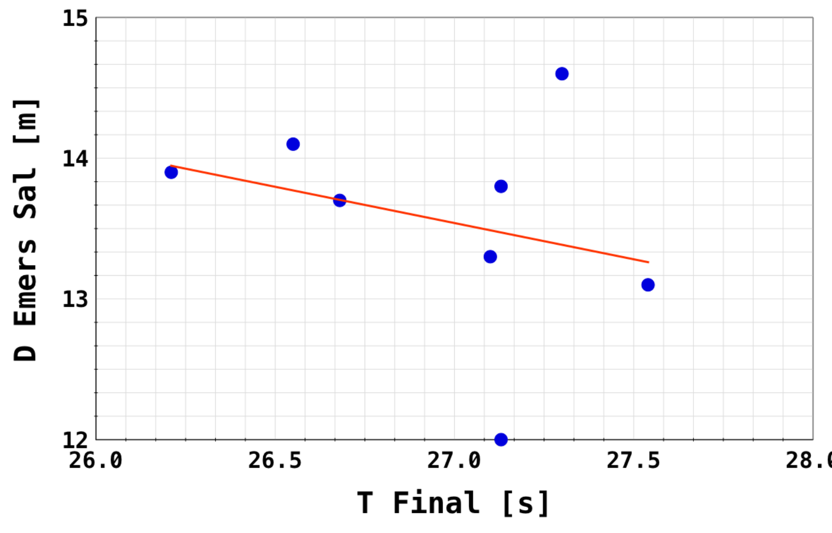

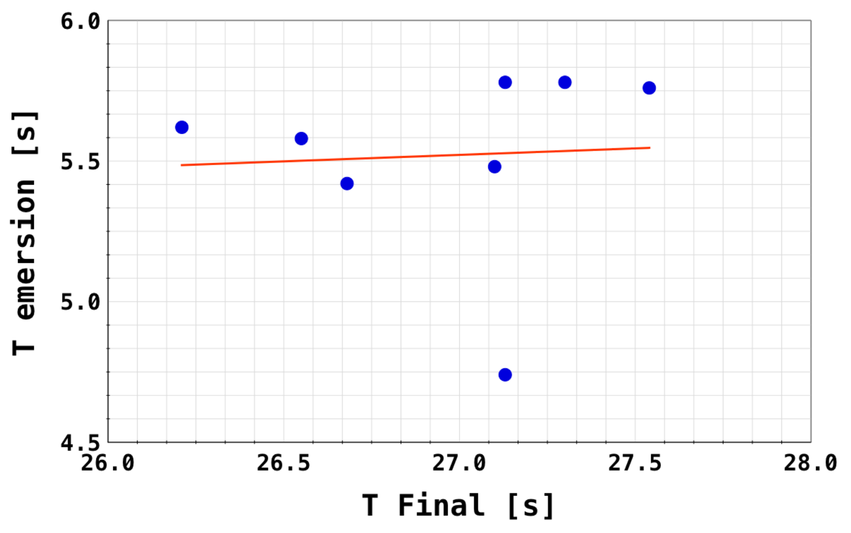

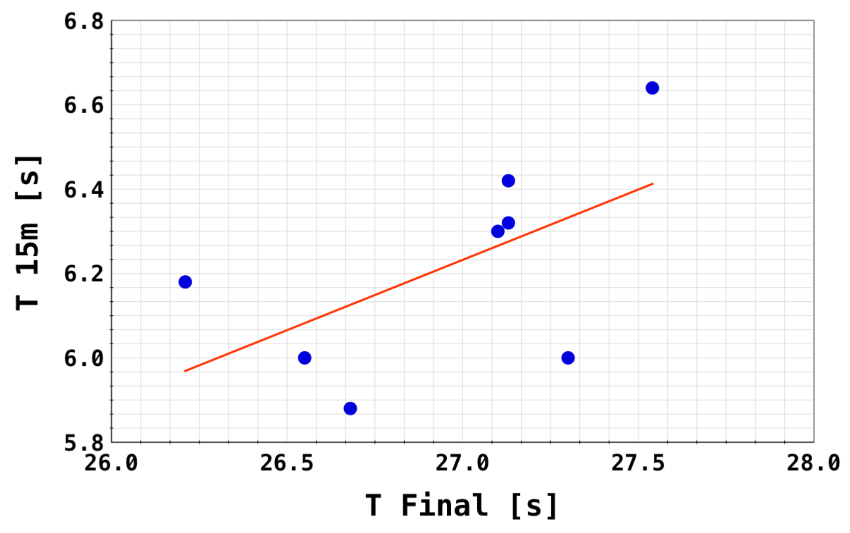

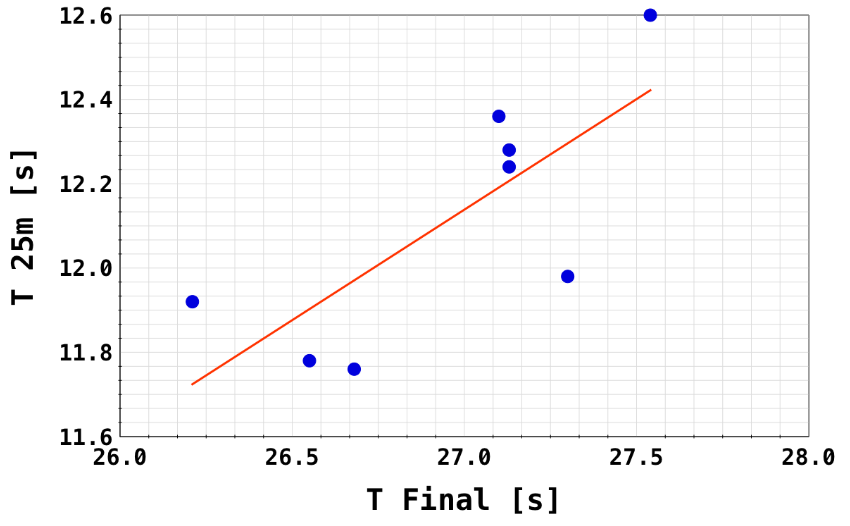

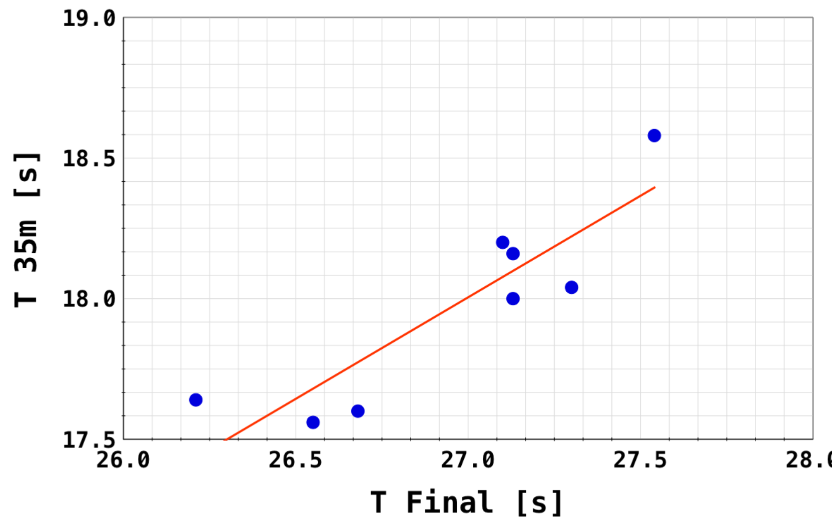

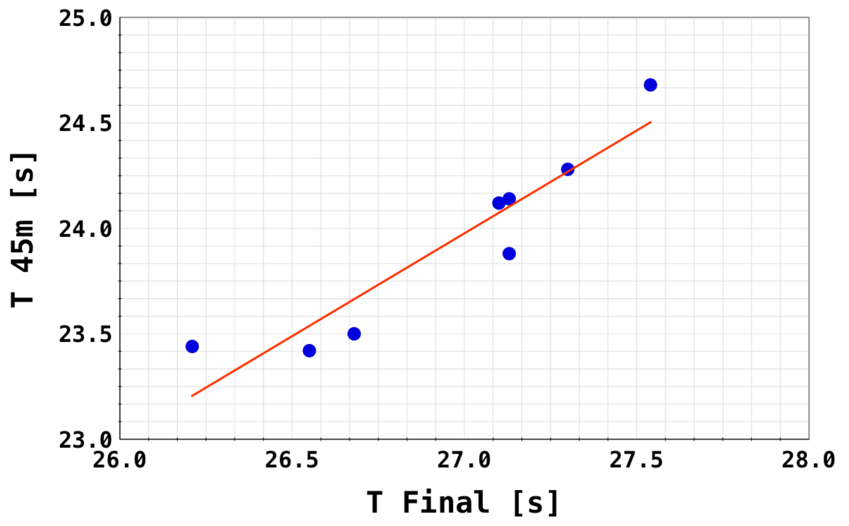

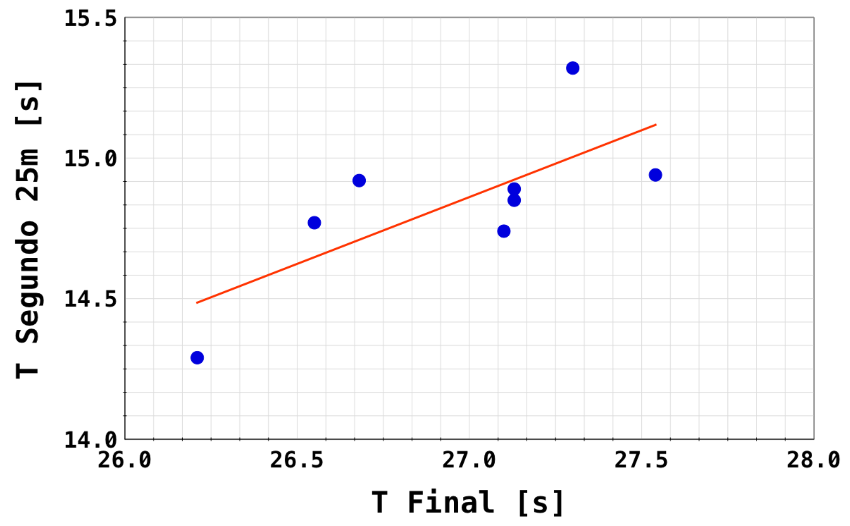

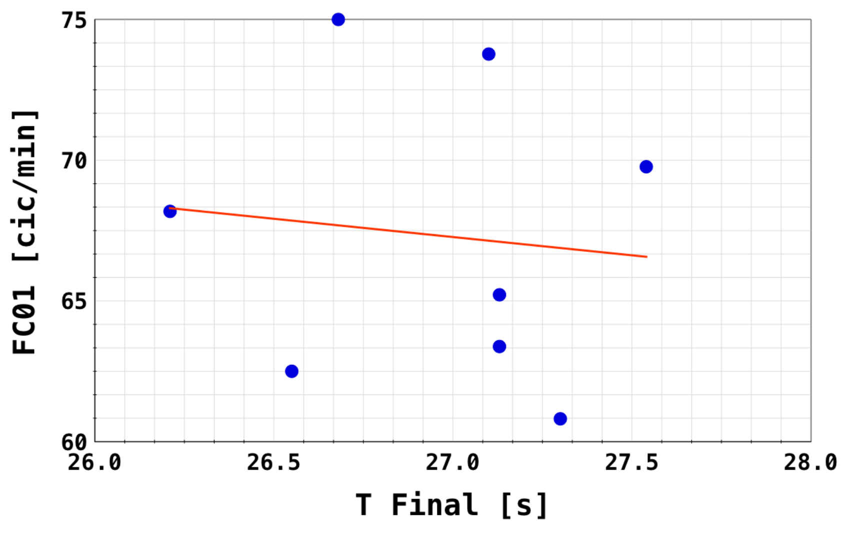

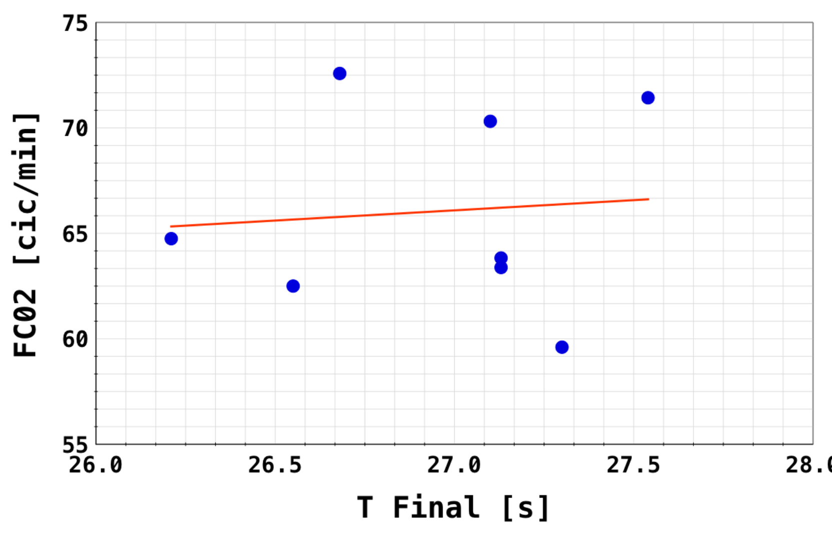

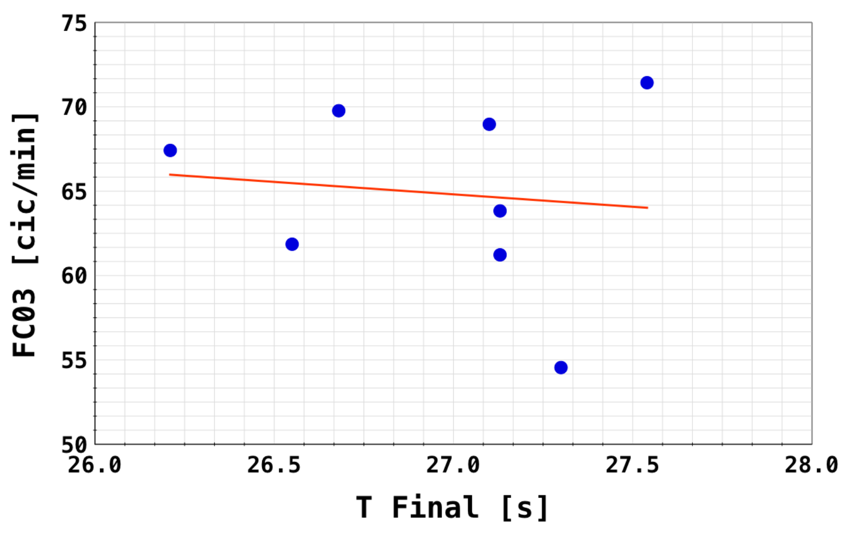

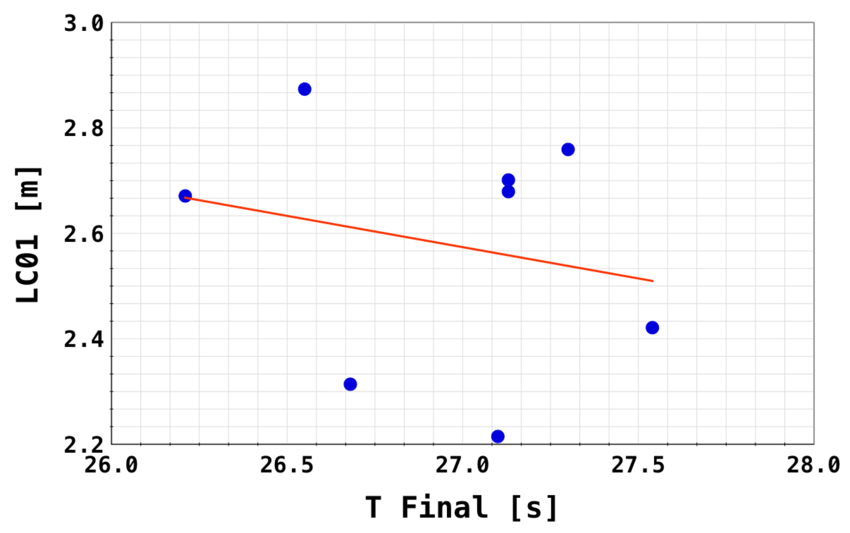

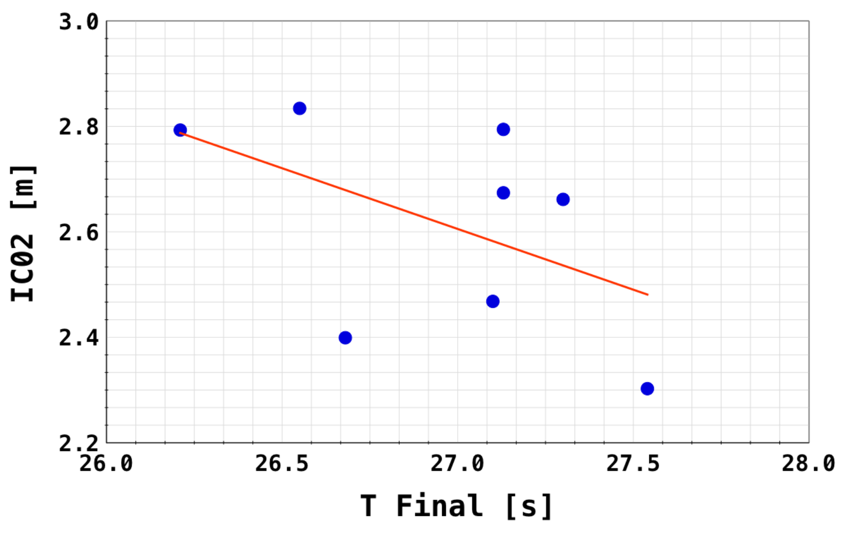

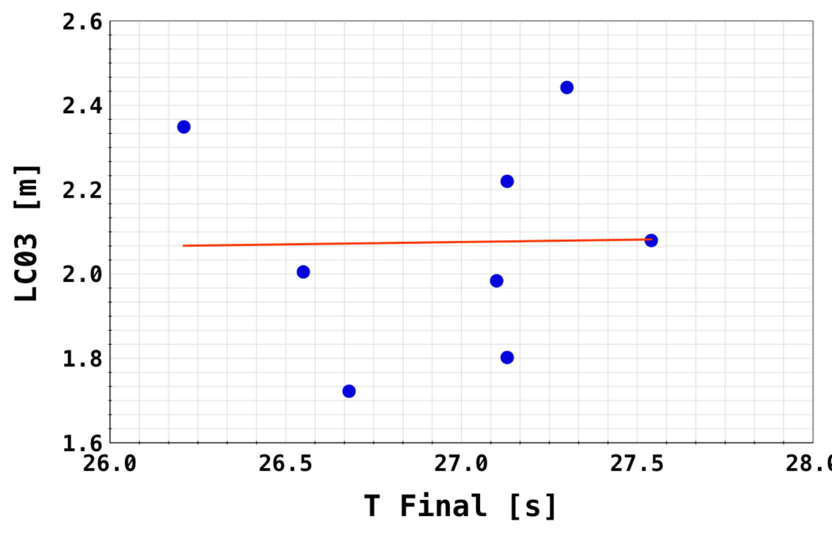

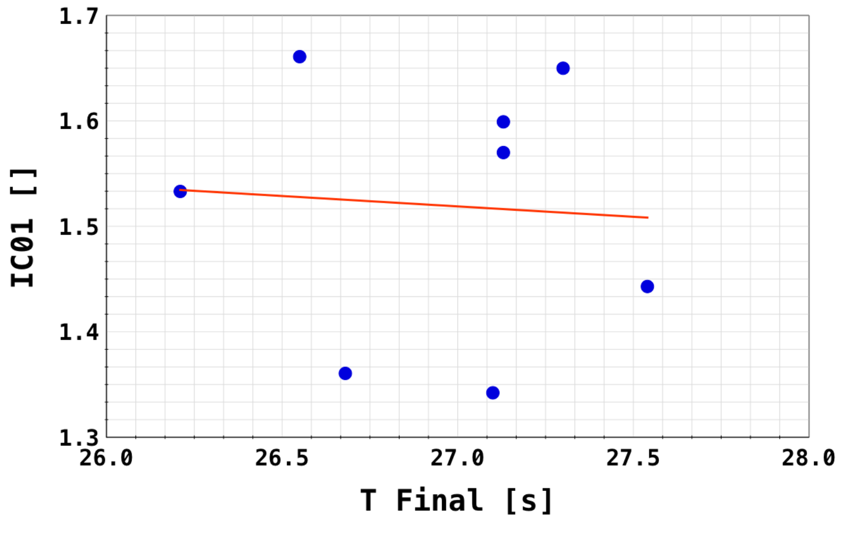

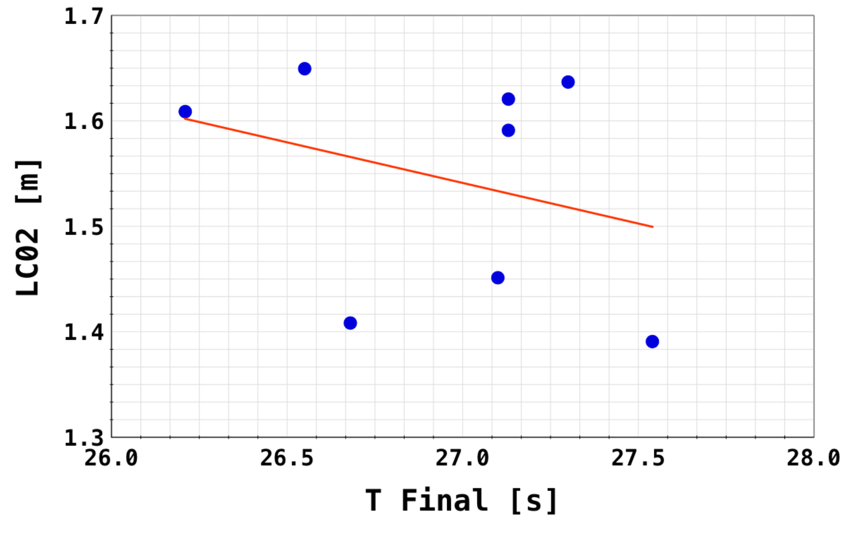

LEN European Aquatics Championships [BUDAPEST, Hungria] 17/05/2021 - 23/05/2021

50 Breaststroke Male

						LANE	Pos.	RT	T_entr	T_emer	t15	t25	t35	t45	T50	2nd 25	F01	F02	F03	LC1	LC2	LC3	Flight_T	T_Underw_01	D_Underw	Speed Underw
M	Breaststroke	Braza	50	Heat 6 of 6	Heat	4	1	0,63	0,98	5,96	6,26	12,04	17,76	23,60	26,34	14,30	66,18	60,81	58,25	1,57	1,71	1,69	0,35	4,98	10,40	2,09
M	Breaststroke	Braza	50	Heat 5 of 6	Heat	4	1	0,64	1,04	6,00	6,10	11,94	17,70	23,46	26,54	14,60	70,31	73,77	68,18	1,46	1,41	1,29	0,40	4,96	10,50	2,12
M	Breaststroke	Braza	50	Heat 4 of 6	Heat	4	1	0,63	0,96	5,60	6,06	12,02	17,84	23,62	26,72	14,70	59,60	60,81	56,60	1,69	1,70	1,54	0,33	4,64	10,30	2,22
M	Breaststroke	Braza	50	Heat 5 of 6	Heat	3	2	0,69	0,96	5,30	6,40	12,22	18,06	23,92	26,82	14,60	73,77	71,43	70,59	1,40	1,44	1,32	0,27	4,34	9,50	2,19
M	Breaststroke	Braza	50	Heat 6 of 6	Heat	5	2	0,60	0,96	6,24	5,76	11,90	17,92	24,06	26,93	15,03	59,60	56,25	61,22	1,64	1,75	1,54	0,36	5,28	12,00	2,27
M	Breaststroke	Braza	50	Heat 4 of 6	Heat	3	2	0,70	1,04	5,64	6,28	12,26	18,14	23,98	27,00	14,74	62,50	62,07	60,61	1,61	1,65	1,48	0,34	4,60	10,20	2,22
M	Breaststroke	Braza	50	Heat 3 of 6	Heat	1	1	0,70	0,98	4,66	6,44	12,48	18,38	24,30	27,18	14,70	65,22	61,64	58,82	1,52	1,65	1,59	0,28	3,68	10,17	2,76
M	Breaststroke	Braza	50	Heat 5 of 6	Heat	6	3	0,69	1,02	6,12	6,24	12,28	18,26	24,36	27,26	14,98	68,18	69,23	68,97	1,46	1,43	1,35	0,33	5,10	10,70	2,10
M	Breaststroke	Braza	50	Heat 4 of 6	Heat	5	3	0,65	1,00	5,78	6,46	12,38	18,18	24,22	27,27	14,89	64,29	62,94	60,00	1,58	1,61	1,48	0,35	4,78	9,90	2,07
M	Breaststroke	Braza	50	Heat 5 of 6	Heat	5	4	0,62	1,06	5,98	6,56	12,50	18,34	24,16	27,36	14,86	63,83	61,22	58,82	1,58	1,68	1,43	0,44	4,92	10,50	2,13
M	Breaststroke	Braza	50	Heat 5 of 6	Heat	2	5	0,67	1,00	5,74	6,40	12,50	18,48	24,54	27,43	14,93	65,69	64,29	58,25	1,50	1,55	1,60	0,33	4,74	8,80	1,86
M	Breaststroke	Braza	50	Heat 4 of 6	Heat	6	5	0,66	1,04	7,44	5,76	12,26	18,26	24,44	27,44	15,18	68,18	66,67	61,22	1,35	1,48	1,47	0,38	6,40	13,30	2,08
M	Breaststroke	Braza	50	Heat 6 of 6	Heat	6	3	0,65	0,98	5,20	6,62	12,62	18,52	24,54	27,51	14,89	61,64	60,00	58,82	1,62	1,68	1,55	0,33	4,22	8,80	2,09
M	Breaststroke	Braza	50	Heat 6 of 6	Heat	2	4	0,66	1,02	6,00	6,42	12,52	18,54	24,68	27,58	15,06	59,21	60,40	59,41	1,66	1,63	1,57	0,36	4,98	10,50	2,11
M	Breaststroke	Braza	50	Heat 3 of 6	Heat	7	2	0,64	0,98	5,30	6,20	12,28	18,40	24,52	27,64	15,36	55,90	55,90	52,17	1,77	1,75	1,66	0,34	4,32	9,60	2,22
M	Breaststroke	Braza	50	Heat 4 of 6	Heat	1	6	0,62	0,98	5,68	6,18	12,34	18,54	24,70	27,71	15,37	62,50	62,50	61,22	1,56	1,55	1,47	0,36	4,70	10,10	2,15
MEANS								0,65	1,00	5,79	6,26	12,28	18,21	24,19	27,17	14,89	64,16	63,12	60,82	1,56	1,61	1,50	0,35	4,79	10,33	2,17

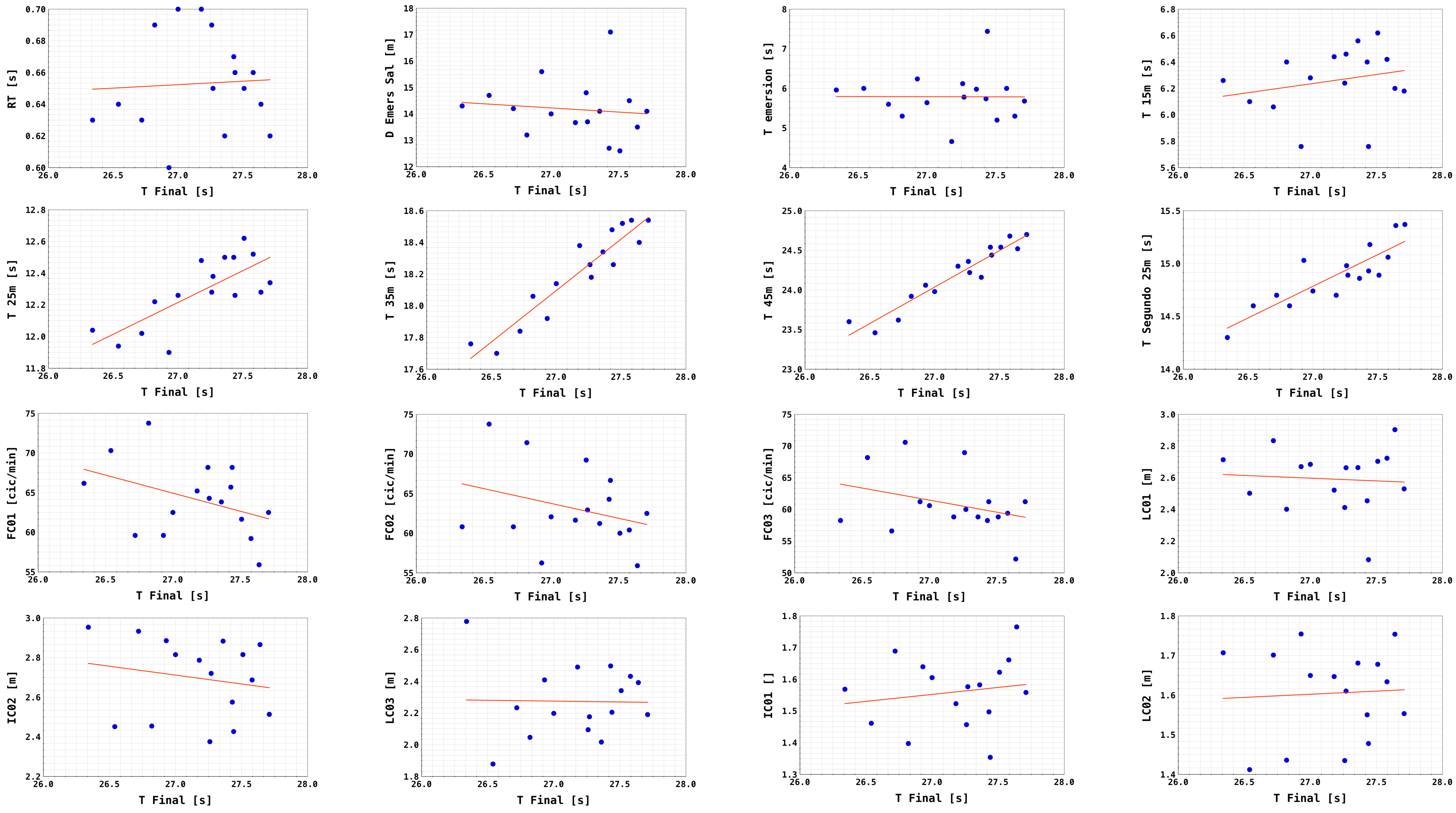

frontiers

LEN European Aquatics Championships [BUDAPEST, Hungria] 17/05/2021 - 23/05/2021

50 Breaststroke Male

						LANE	Pos.	RT	T_entr	T_emer	t15	t25	t35	t45	T50	2nd 25	F01	F02	F03	LC1	LC2	LC3	Flight_T	T_Underw_01	D_Underw	Speed Underw
M	Breaststroke	Braza	50	Semifinal 2	SF	4	1	0,62	0,96	5,78	6,30	11,94	17,80	23,56	26,38	14,44	66,67	62,50	61,86	1,60	1,65	1,55	0,34	4,82	10,00	2,07
M	Breaststroke	Braza	50	Semifinal 2	SF	5	2	0,64	0,96	5,78	6,00	11,76	17,42	23,42	26,47	14,71	60,00	62,07	58,25	1,74	1,66	1,52	0,32	4,82	11,30	2,34
M	Breaststroke	Braza	50	Semifinal 1	SF	4	1	0,63	1,02	5,70	5,96	11,80	17,64	23,40	26,49	14,69	73,17	71,43	68,97	1,40	1,45	1,27	0,39	4,68	10,30	2,20
M	Breaststroke	Braza	50	Semifinal 1	SF	5	2	0,69	0,98	5,18	6,42	12,32	18,10	24,10	27,09	14,77	72,58	69,23	65,93	1,40	1,47	1,37	0,29	4,20	9,60	2,29
M	Breaststroke	Braza	50	Semifinal 1	SF	6	3	0,69	1,00	6,00	6,22	12,12	18,06	24,08	27,11	14,99	68,18	71,43	72,29	1,49	1,40	1,23	0,31	5,00	11,00	2,20
M	Breaststroke	Braza	50	Semifinal 1	SF	2	4	0,65	1,08	6,76	6,60	12,38	18,22	24,14	27,13	14,75	62,07	62,94	61,86	1,67	1,62	1,46	0,43	5,68	11,40	2,01
M	Breaststroke	Braza	50	Semifinal 2	SF	6	3	0,71	1,00	4,78	6,36	12,40	18,14	24,10	27,14	14,74	65,69	62,07	61,86	1,51	1,65	1,44	0,29	3,78	8,20	2,17
M	Breaststroke	Braza	50	Semifinal 2	SF	3	4	0,63	0,98	5,90	5,90	12,04	17,98	24,08	27,16	15,12	61,22	58,44	58,82	1,60	1,71	1,49	0,35	4,92	11,00	2,24
M	Breaststroke	Braza	50	Semifinal 1	SF	3	5	0,70	1,04	5,40	6,24	12,26	18,12	24,00	27,20	14,94	63,83	61,64	62,50	1,56	1,66	1,35	0,34	4,36	9,40	2,16
M	Breaststroke	Braza	50	Semifinal 1	SF	1	6	0,64	0,98	5,66	6,26	12,20	18,24	24,30	27,32	15,12	60,40	60,81	60,00	1,67	1,63	1,49	0,34	4,68	10,20	2,18
M	Breaststroke	Braza	50	Semifinal 2	SF	7	5	0,70	1,00	5,22	6,54	12,34	18,20	24,36	27,33	14,99	65,22	63,38	61,22	1,59	1,58	1,48	0,30	4,22	9,20	2,18
M	Breaststroke	Braza	50	Semifinal 2	SF	2	6	0,68	1,04	5,80	6,60	12,54	18,36	24,44	27,34	14,80	66,18	63,38	63,83	1,53	1,59	1,46	0,36	4,76	9,80	2,06
M	Breaststroke	Braza	50	Semifinal 2	SF	1	7	0,65	0,94	5,06	6,60	12,46	18,44	24,48	27,36	14,90	64,29	61,64	58,82	1,59	1,62	1,59	0,29	4,12	8,90	2,16
M	Breaststroke	Braza	50	Semifinal 1	SF	7	7	0,67	1,02	6,54	6,12	12,06	18,10	24,24	27,44	15,38	70,87	66,67	65,22	1,43	1,48	1,29	0,35	5,52	11,70	2,12
M	Breaststroke	Braza	50	Semifinal 2	SF	8	8	0,63	1,00	5,34	6,28	12,32	18,38	24,58	27,79	15,47	58,44	55,21	54,55	1,70	1,77	1,54	0,37	4,34	9,50	2,19
M	Breaststroke	Braza	50	Semifinal 1	SF	8	8	0,65	1,02	5,84	6,20	12,24	18,42	24,64	27,80	15,56	64,29	62,07	63,16	1,55	1,56	1,35	0,37	4,82	10,50	2,18
MEANS								0,66	1,00	5,67	6,29	12,20	18,10	24,12	27,16	14,96	65,19	63,43	62,45	1,56	1,59	1,43	0,34	4,67	10,13	2,17

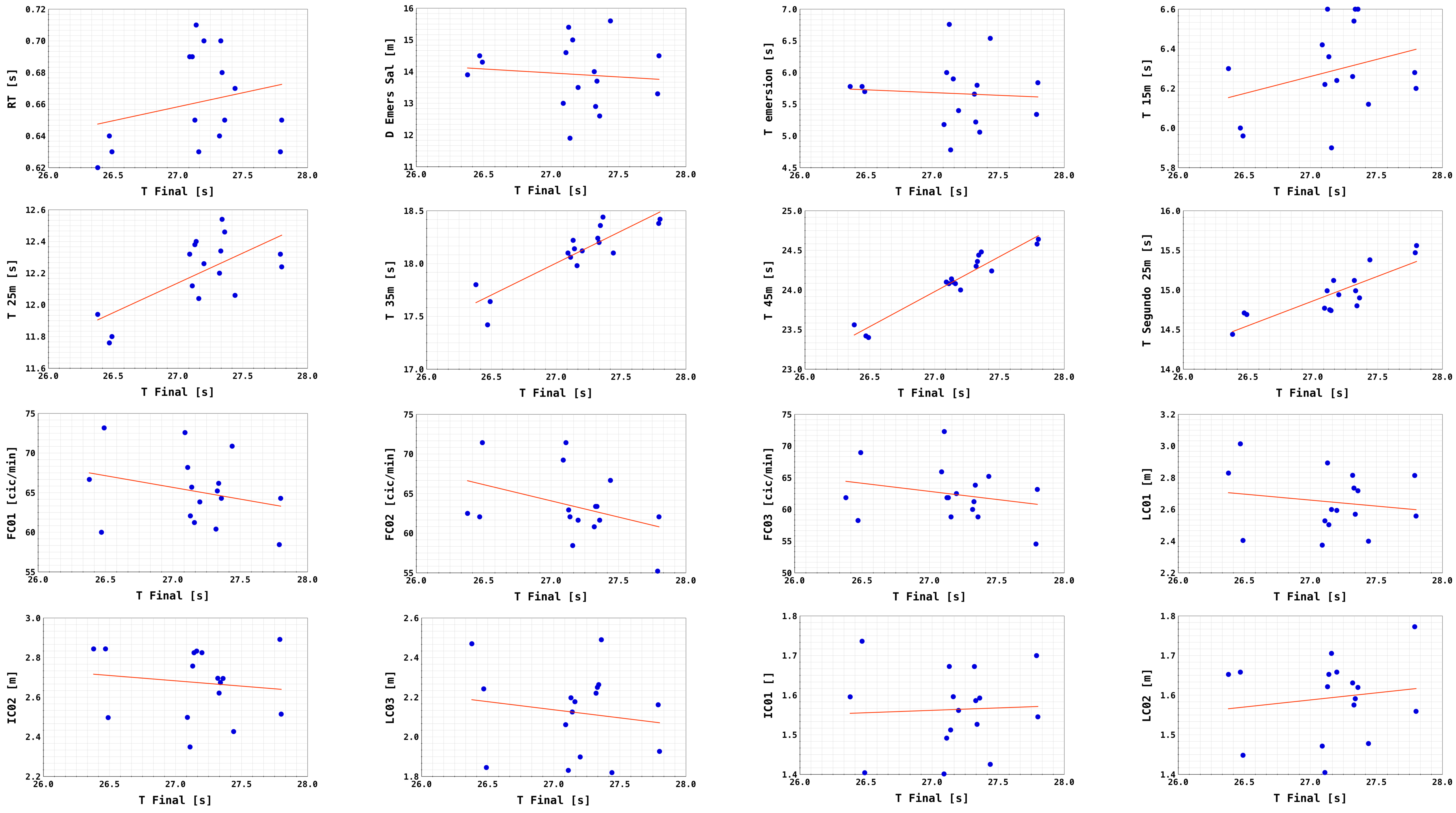

LEN European Aquatics Championships [BUDAPEST, Hungria] 17/05/2021 - 23/05/2021

50 Backstroke Male

						LANE	Pos.	RT	T_entr	T_emer	t15	t25	t35	t45	T50	2nd 25	F01	F02	F03	LC1	LC2	LC3	Flight_T	T_Underw_01	D_Underw	Speed Underw
M	Backstroke	Espalda	50	Final	Final	4	1	0,69	0,71	5,19	5,95	10,95	16,05	21,21	23,80	12,85	58,82	58,06	56,34	2,04	2,01	1,85	0,02	4,48	10,80	2,41
M	Backstroke	Espalda	50	Final	Final	3	2	0,57	0,64	5,02	5,88	11,04	16,28	21,72	24,42	13,38	65,22	61,43	61,86	1,78	1,83	1,62	0,07	4,38	10,50	2,40
M	Backstroke	Espalda	50	Final	Final	6	3	0,53	0,64	4,94	6,12	11,22	16,42	21,86	24,47	13,25	56,96	54,22	55,30	2,07	2,08	1,87	0,11	4,30	9,80	2,28
M	Backstroke	Espalda	50	Final	Final	5	4	0,57	0,63	4,93	5,95	11,20	16,51	21,96	24,59	13,39	55,56	52,33	50,85	2,06	2,13	2,02	0,06	4,30	10,20	2,37
M	Backstroke	Espalda	50	Final	Final	2	5	0,55	0,68	5,56	6,20	11,38	16,54	21,98	24,72	13,34	54,22	52,33	52,17	2,14	2,16	1,89	0,13	4,88	10,80	2,21
M	Backstroke	Espalda	50	Final	Final	1	6	0,53	0,68	5,56	5,86	11,14	16,54	22,16	24,89	13,75	56,25	53,57	55,56	2,02	2,03	1,78	0,15	4,88	11,60	2,38
M	Backstroke	Espalda	50	Final	Final	7	7	0,47	0,67	5,21	5,99	11,25	16,55	22,01	24,92	13,67	58,82	58,44	54,30	1,94	1,91	1,71	0,20	4,54	10,70	2,36
M	Backstroke	Espalda	50	Final	Final	8	7	0,62	0,80	5,54	5,88	11,10	16,48	22,05	24,92	13,82	56,96	54,88	52,86	2,02	2,00	1,78	0,18	4,74	11,35	2,39
MEANS								0,57	0,68	5,24	5,98	11,16	16,42	21,87	24,59	13,43	57,85	55,66	54,90	2,01	2,02	1,81	0,12	4,56	10,72	2,35

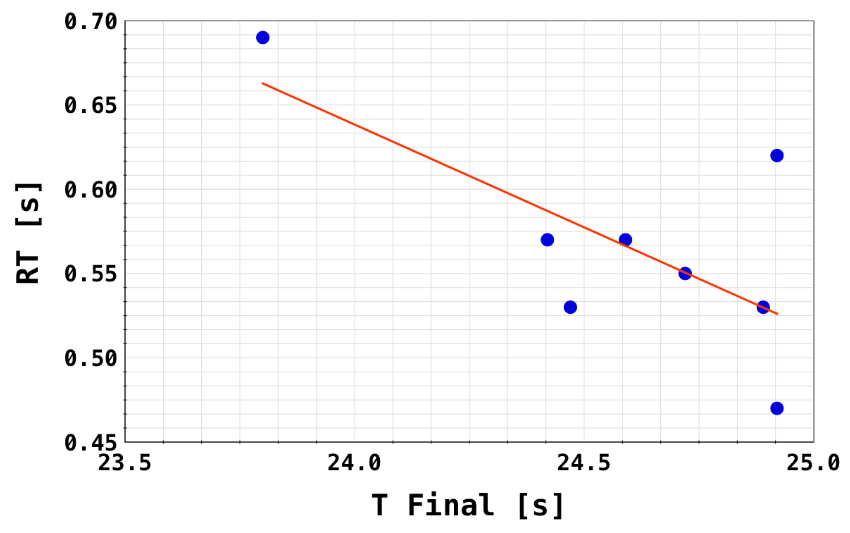

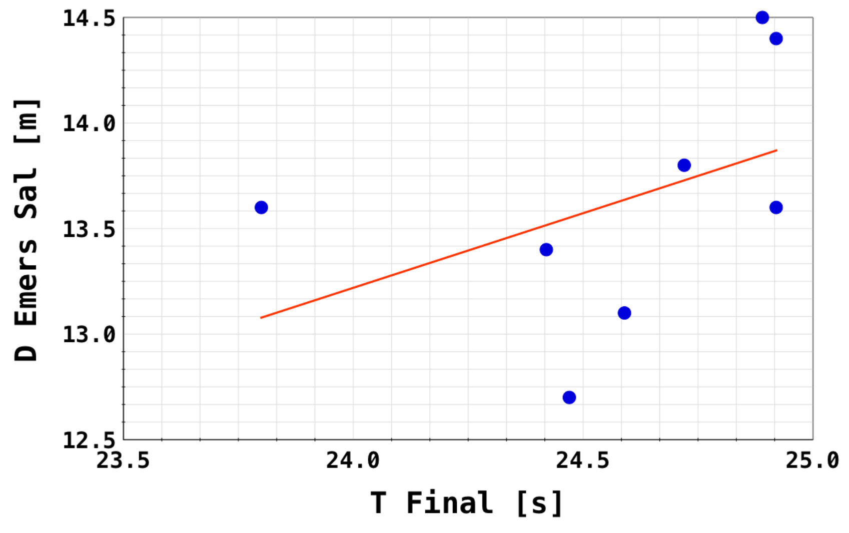

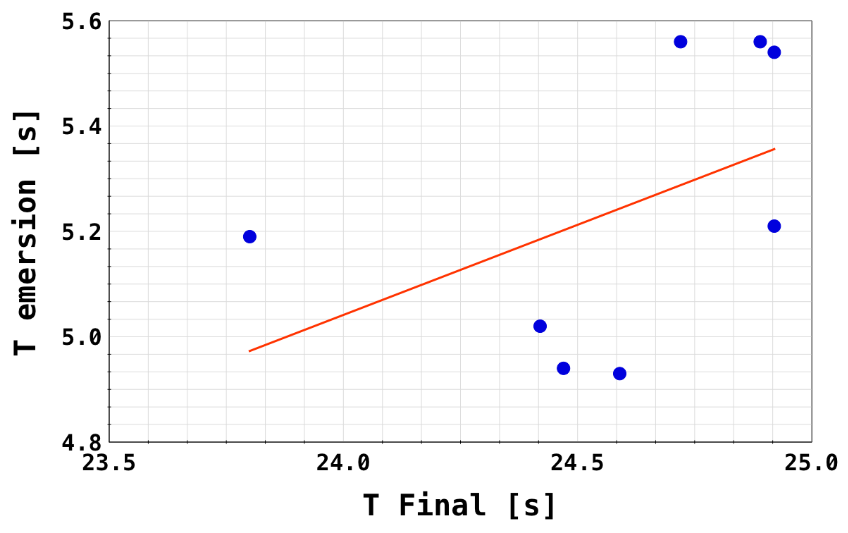

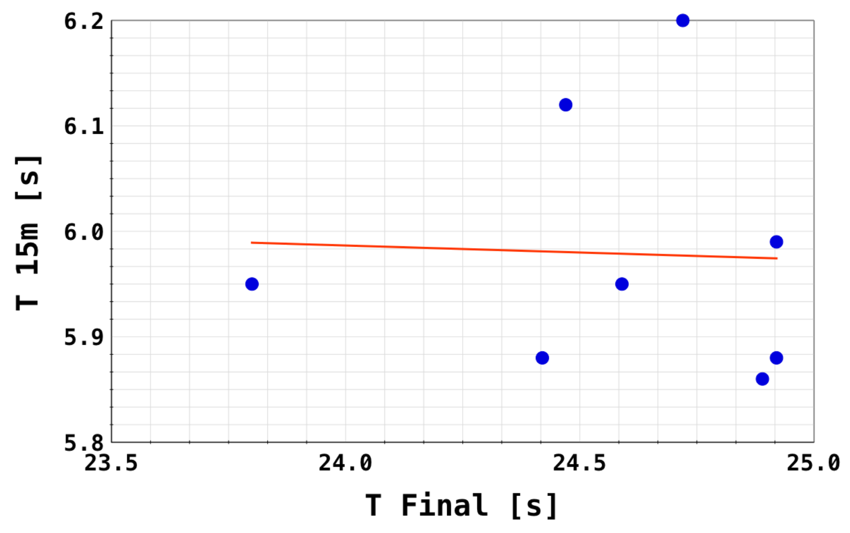

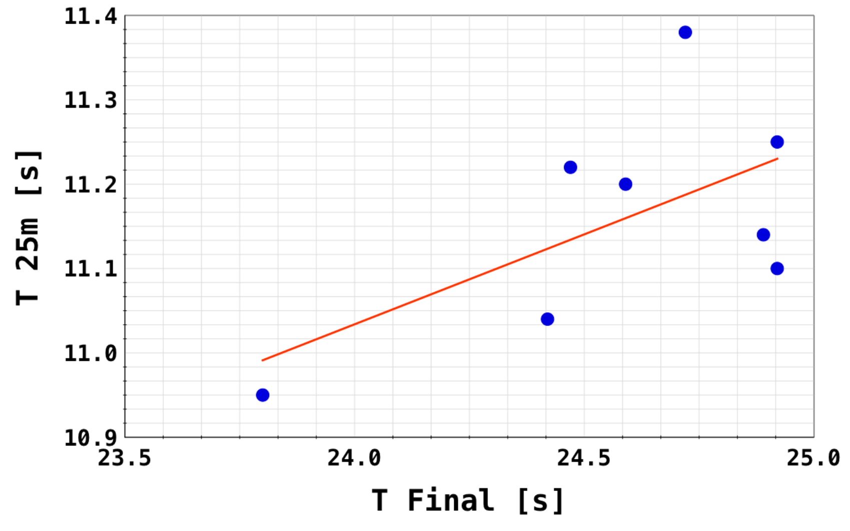

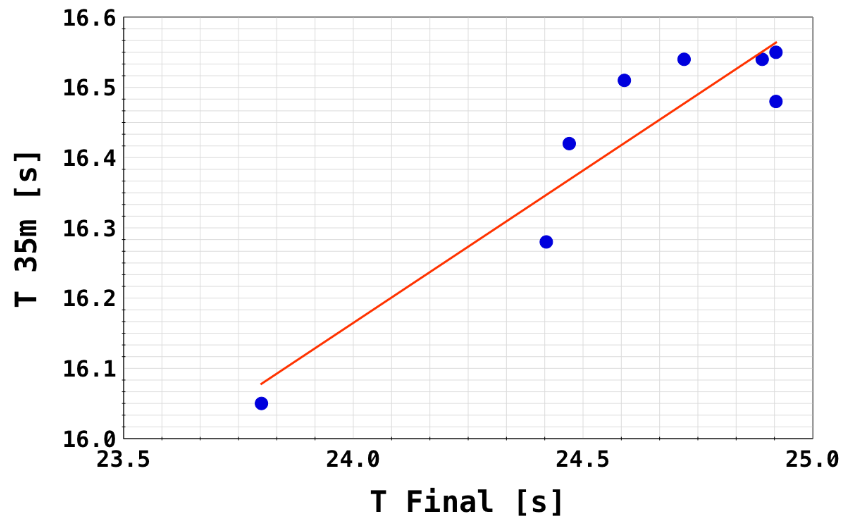

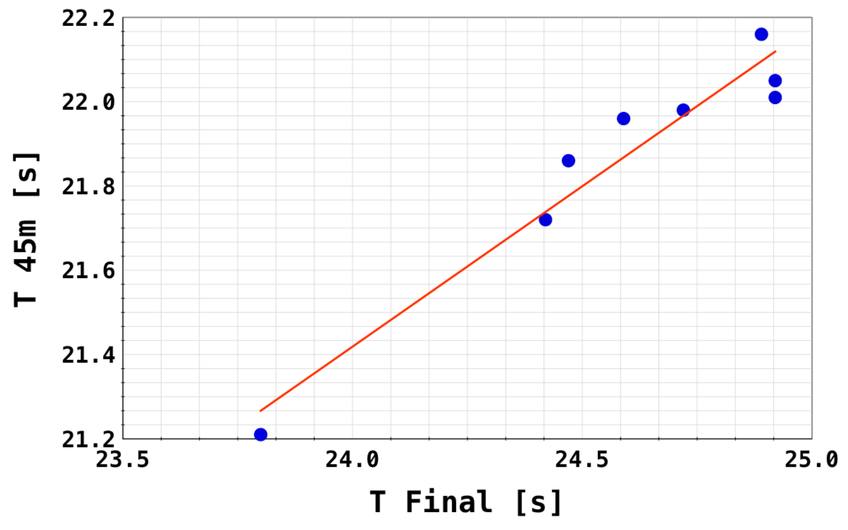

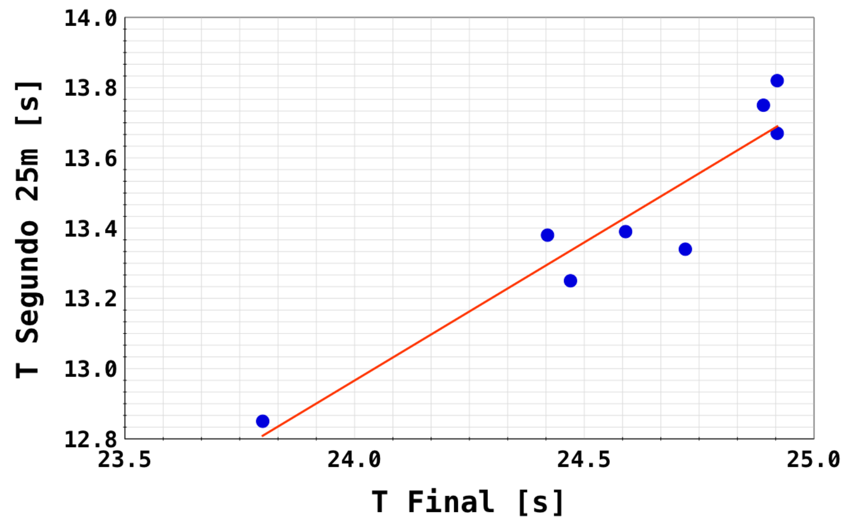

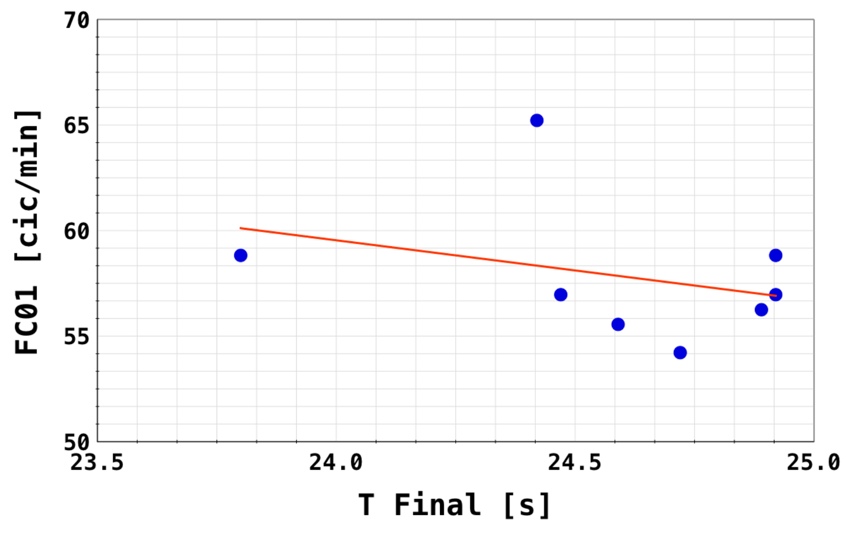

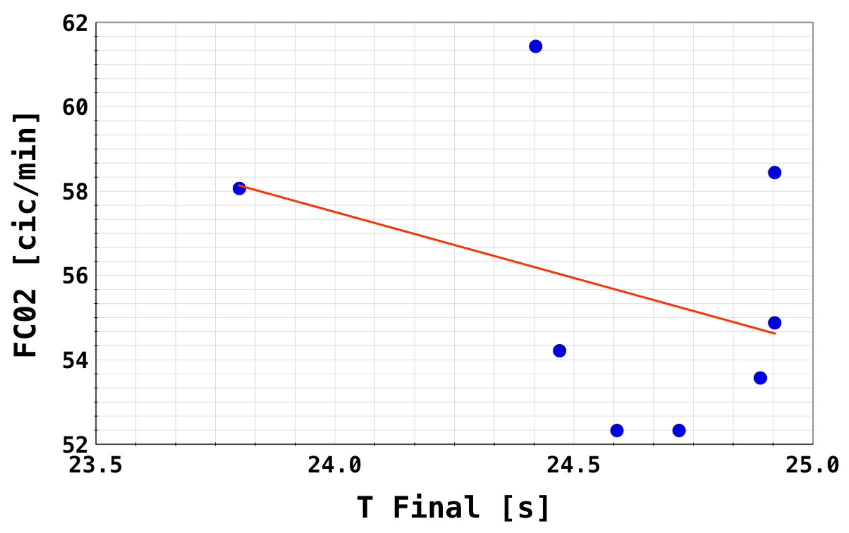

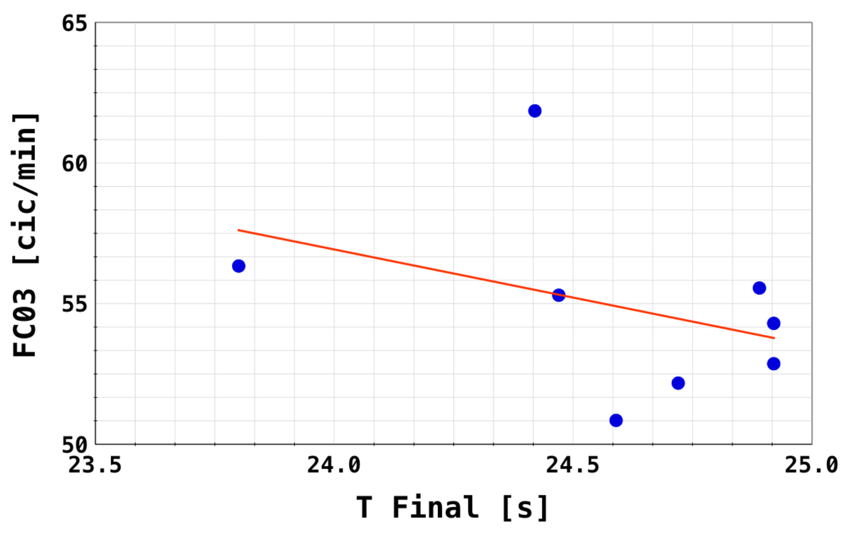

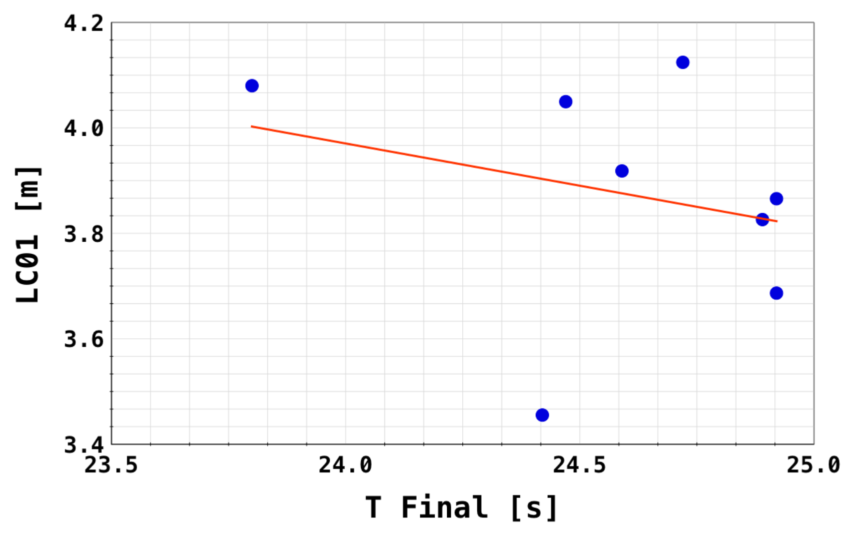

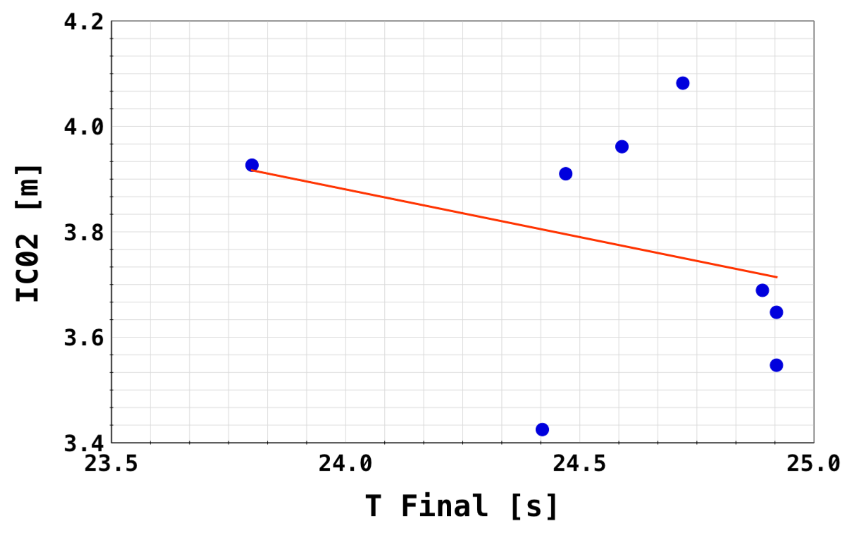

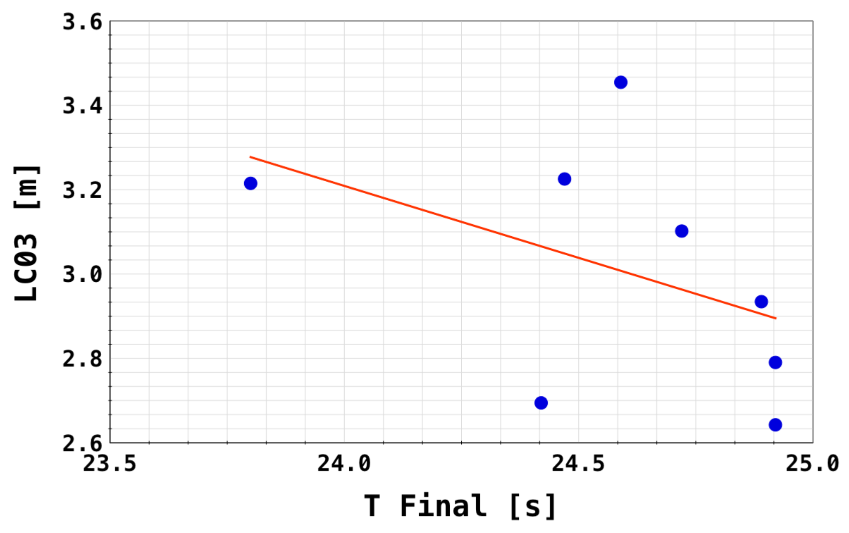

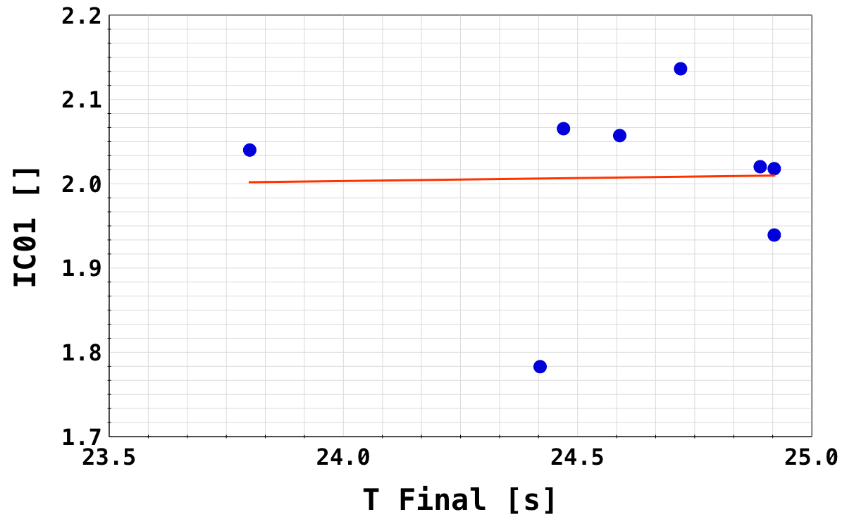

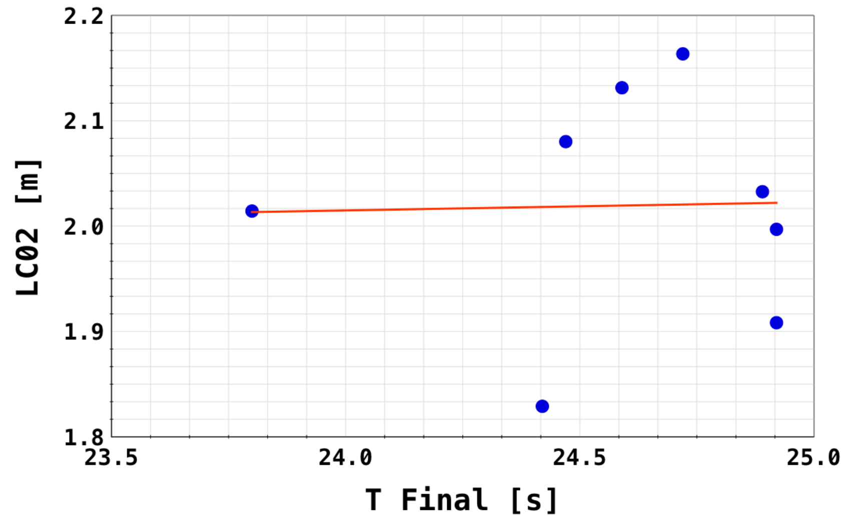

LEN European Aquatics Championships [BUDAPEST, Hungria] 17/05/2021 - 23/05/2021

50 Backstroke Male

						LANE	Pos.	RT	T_entr	T_emer	t15	t25	t35	t45	T50	2nd 25	F01	F02	F03	LC1	LC2	LC3	Flight_T	T_Underw_01	D_Underw	Speed Underw
M	Backstroke	Espalda	50	Heat 6 of 6	Heat	4	1	0,69	0,72	5,22	6,02	11,12	16,36	21,62	24,23	13,11	59,21	56,96	58,25	1,99	2,01	1,78	0,03	4,50	10,50	2,33
M	Backstroke	Espalda	50	Heat 5 of 6	Heat	4	1	0,58	0,70	5,30	6,00	11,22	16,46	21,86	24,51	13,29	61,22	57,32	57,69	1,88	1,97	1,77	0,12	4,60	10,80	2,35
M	Backstroke	Espalda	50	Heat 4 of 6	Heat	7	1	0,58	0,70	5,30	6,20	11,30	16,54	22,00	24,62	13,32	55,56	52,63	53,57	2,12	2,13	1,92	0,12	4,60	10,40	2,26
M	Backstroke	Espalda	50	Heat 4 of 6	Heat	4	2	0,57	0,78	5,20	6,26	11,52	16,76	22,08	24,67	13,15	52,63	50,85	48,58	2,17	2,23	2,15	0,21	4,42	9,90	2,24
M	Backstroke	Espalda	50	Heat 6 of 6	Heat	5	2	0,58	0,66	5,30	6,14	11,42	16,72	22,14	24,78	13,36	54,22	50,56	51,72	2,10	2,21	1,98	0,08	4,64	10,80	2,33
M	Backstroke	Espalda	50	Heat 5 of 6	Heat	3	2	0,62	0,62	6,12	6,14	11,46	16,82	22,33	24,96	13,50	54,22	50,56	50,42	2,08	2,18	2,04	0,00	5,50	12,30	2,24
M	Backstroke	Espalda	50	Heat 1 of 6	Heat	5	1	0,59	0,72	6,04	6,04	11,32	16,74	22,34	24,98	13,66	54,88	51,43	48,78	2,07	2,12	2,10	0,13	5,32	12,30	2,31
M	Backstroke	Espalda	50	Heat 6 of 6	Heat	1	3	0,57	0,68	4,98	6,32	11,54	16,88	22,40	25,05	13,51	63,83	61,64	55,05	1,80	1,79	1,85	0,11	4,30	10,00	2,33
M	Backstroke	Espalda	50	Heat 5 of 6	Heat	5	3	0,54	0,66	5,16	6,08	11,37	16,78	22,42	25,08	13,71	58,44	55,21	54,55	1,94	1,97	1,86	0,12	4,50	10,70	2,38
M	Backstroke	Espalda	50	Heat 6 of 6	Heat	3	4	0,58	0,76	5,56	6,12	11,46	16,84	22,32	25,09	13,63	55,90	53,89	53,10	2,01	2,05	1,84	0,18	4,80	11,05	2,30
M	Backstroke	Espalda	50	Heat 4 of 6	Heat	3	3	0,61	0,78	5,94	5,96	11,36	16,80	22,40	25,11	13,75	55,73	52,94	54,55	1,99	2,05	1,83	0,17	5,16	11,95	2,32
M	Backstroke	Espalda	50	Heat 5 of 6	Heat	1	4	0,65	0,72	5,70	6,24	11,58	16,96	22,54	25,14	13,56	54,88	52,33	51,28	2,05	2,09	2,03	0,07	4,98	11,15	2,24
M	Backstroke	Espalda	50	Heat 6 of 6	Heat	0	5	0,50	0,68	5,42	6,16	11,50	16,88	22,48	25,21	13,71	57,69	56,96	55,05	1,95	1,92	1,80	0,18	4,74	10,80	2,28
M	Backstroke	Espalda	50	Heat 6 of 6	Heat	6	6	0,56	0,66	5,24	5,86	11,17	16,68	22,44	25,23	14,06	59,80	57,32	52,63	1,89	1,86	1,84	0,10	4,58	11,30	2,47
M	Backstroke	Espalda	50	Heat 6 of 6	Heat	9	7	0,58	0,62	5,72	5,94	11,20	16,80	22,54	25,28	14,08	54,55	50,42	52,17	2,09	2,10	1,89	0,04	5,10	12,50	2,45
M	Backstroke	Espalda	50	Heat 6 of 6	Heat	7	7	0,52	0,66	5,40	5,98	11,34	16,88	22,48	25,28	13,94	56,60	53,89	57,14	1,98	2,00	1,69	0,14	4,74	11,20	2,36
						MEANS		0,58	0,70	5,48	6,09	11,37	16,74	22,27	24,95	13,58	56,83	54,06	53,41	2,01	2,04	1,90	0,11	4,78	11,10	2,32

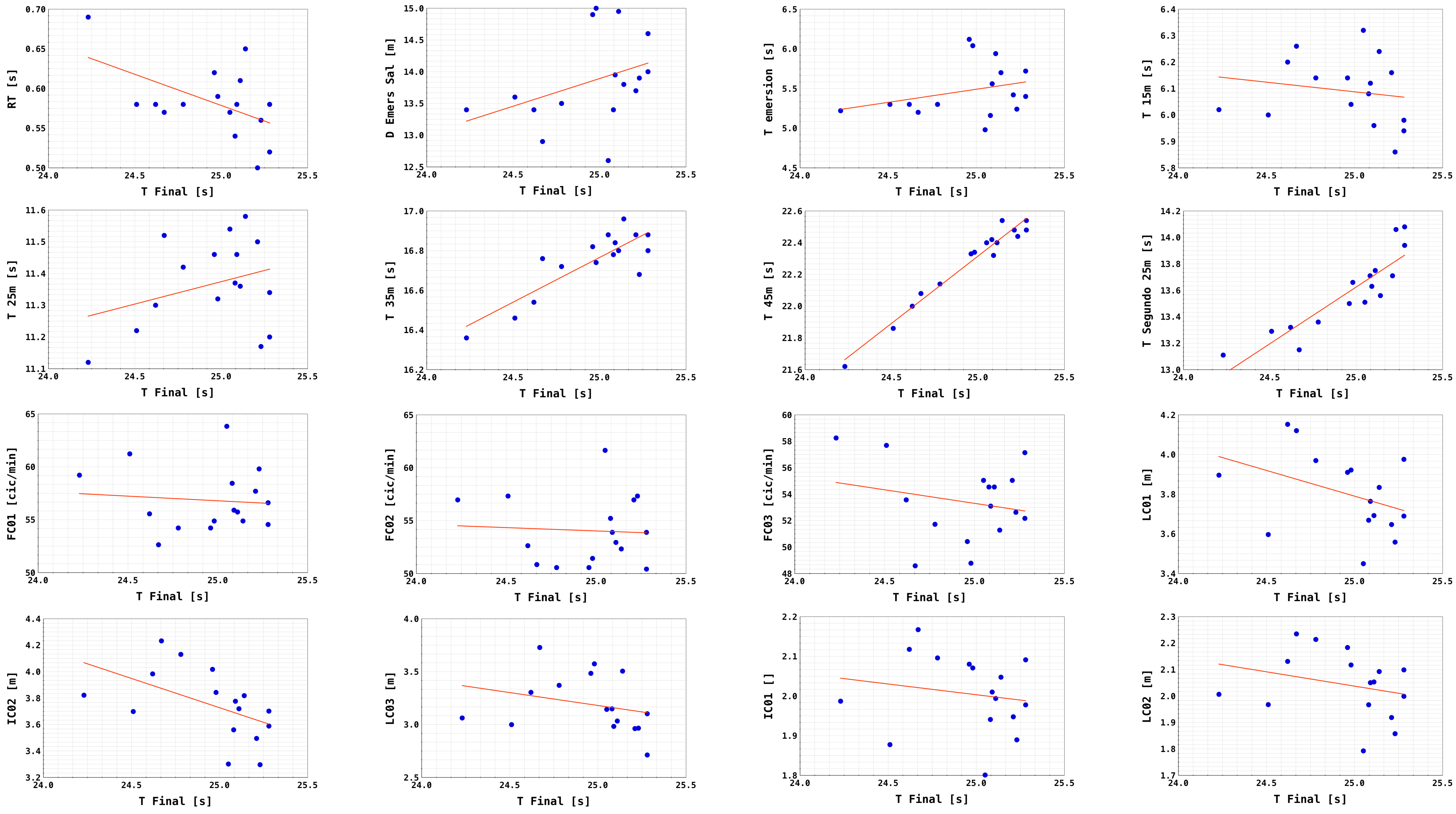

LEN European Aquatics Championships
[BUDAPEST, Hungria] 17/05/2021 - 23/05/2021

50 Backstroke Male

						LANE	Pos.	RT	T_entr	T_emer	t15	t25	t35	t45	T50	2nd 25	F01	F02	F03	LC1	LC2	LC3	Flight_T	T_Underw_01	D_Underw	Speed Underw
M	Backstroke	Espalda	50	Semifinal 2	SF	4	1	0,66	0,70	5,08	5,88	10,90	16,04	21,30	23,93	13,03	59,60	56,96	58,25	2,01	2,03	1,76	0,04	4,38	10,60	2,42
M	Backstroke	Espalda	50	Semifinal 2	SF	3	2	0,60	0,68	5,24	5,96	11,20	16,52	21,83	24,49	13,29	55,56	52,17	50,85	2,06	2,16	2,00	0,08	4,56	11,00	2,41
M	Backstroke	Espalda	50	Semifinal 1	SF	4	1	0,58	0,68	5,56	6,02	11,15	16,42	21,86	24,57	13,42	61,64	57,51	55,56	1,90	1,95	1,79	0,10	4,88	11,40	2,34
M	Backstroke	Espalda	50	Semifinal 2	SF	5	3	0,55	0,68	5,30	6,22	11,36	16,60	21,94	24,60	13,24	55,21	53,57	53,57	2,11	2,12	1,89	0,13	4,62	10,40	2,25
M	Backstroke	Espalda	50	Semifinal 1	SF	5	2	0,56	0,76	5,38	6,14	11,40	16,60	22,00	24,68	13,28	53,57	52,02	50,00	2,13	2,18	2,01	0,20	4,62	10,60	2,29
M	Backstroke	Espalda	50	Semifinal 2	SF	1	4	0,48	0,68	5,40	5,98	11,32	16,62	22,12	24,81	13,49	58,06	57,51	55,81	1,94	1,93	1,80	0,20	4,72	11,00	2,33
M	Backstroke	Espalda	50	Semifinal 2	SF	7	5	0,61	0,82	5,82	5,88	11,20	16,64	22,24	25,02	13,82	56,60	53,57	51,72	1,99	2,03	1,88	0,21	5,00	11,90	2,38
M	Backstroke	Espalda	50	Semifinal 2	SF	8	5	0,53	0,66	5,82	5,82	11,18	16,66	22,18	25,02	13,84	55,90	53,89	50,42	2,00	2,02	1,89	0,13	5,16	12,20	2,36
M	Backstroke	Espalda	50	Semifinal 2	SF	6	7	0,59	0,68	5,78	6,02	11,34	16,86	22,28	25,03	13,69	54,88	52,94	49,59	2,06	2,07	1,98	0,09	5,10	13,50	2,65
M	Backstroke	Espalda	50	Semifinal 1	SF	3	3	0,61	0,58	6,04	6,14	11,44	16,88	22,42	25,11	13,67	56,96	54,55	54,05	1,99	2,00	1,86	-0,03	5,46	12,25	2,24
M	Backstroke	Espalda	50	Semifinal 2	SF	2	8	0,55	0,70	5,36	6,08	11,44	16,88	22,42	25,19	13,75	58,44	55,56	52,63	1,92	1,97	1,85	0,15	4,66	10,90	2,34
M	Backstroke	Espalda	50	Semifinal 1	SF	1	4	0,54	0,64	5,64	5,78	11,06	16,58	22,38	25,23	14,17	60,40	56,25	53,10	1,88	1,88	1,78	0,10	5,00	12,00	2,40
M	Backstroke	Espalda	50	Semifinal 1	SF	6	5	0,58	0,66	5,16	6,40	11,70	17,00	22,46	25,25	13,55	63,60	60,61	57,69	1,78	1,84	1,68	0,08	4,50	10,10	2,24
M	Backstroke	Espalda	50	Semifinal 1	SF	7	6	0,63	0,68	5,48	6,30	11,64	17,00	22,62	25,26	13,62	55,56	53,25	52,63	2,02	2,05	1,94	0,05	4,80	10,90	2,27
M	Backstroke	Espalda	50	Semifinal 1	SF	2	7	0,61	0,80	5,38	6,13	11,48	16,90	22,50	25,36	13,88	57,88	53,57	54,05	1,94	2,03	1,75	0,19	4,58	10,50	2,29
M	Backstroke	Espalda	50	Semifinal 1	SF	8	8	0,55	0,58	5,66	5,90	11,20	16,86	22,72	25,40	14,20	55,90	50,85	52,17	2,03	2,05	1,93	0,03	5,08	12,10	2,38
MEANS								0,58	0,69	5,51	6,04	11,31	16,69	22,20	24,93	13,62	57,49	54,67	53,26	1,98	2,02	1,86	0,11	4,82	11,33	2,35

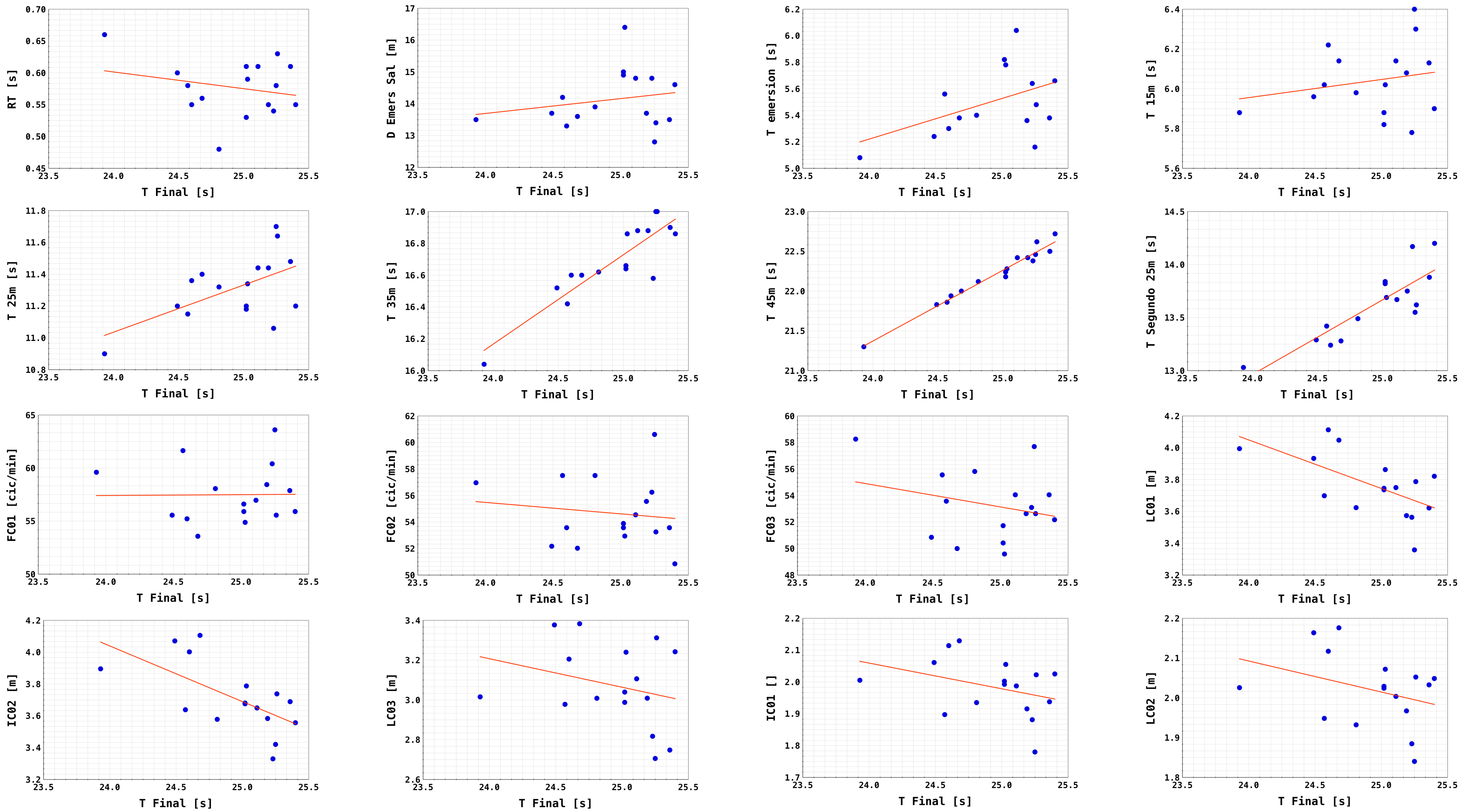

LEN European Aquatics Championships
[BUDAPEST, Hungria] 17/05/2021 - 23/05/2021

50 Freestyle Male

						LANE	Pos.	RT	T_entr	T_emer	t15	t25	t35	t45	T50	2nd 25	F01	F02	F03	LC1	LC2	LC3	Flight_T	T_Underw_01	D_Underw	Speed Underw
M	Freestyle	Libre	50	Final	Final	3	1	0,64	0,92	2,04	5,24	9,76	14,54	19,30	21,61	11,85	63,38	58,82	57,69	2,09	2,14	2,03	0,28	1,12	3,80	3,39
M	Freestyle	Libre	50	Final	Final	2	2	0,58	0,96	4,28	5,30	9,86	14,56	19,40	21,69	11,83	65,22	63,83	61,22	2,02	1,97	1,93	0,38	3,32	9,10	2,74
M	Freestyle	Libre	50	Final	Final	4	3	0,65	0,94	3,12	5,38	9,80	14,62	19,46	21,73	11,93	64,29	61,22	61,86	2,11	2,03	1,92	0,29	2,18	6,60	3,03
M	Freestyle	Libre	50	Final	Final	6	4	0,62	0,98	3,44	5,34	9,88	14,58	19,42	21,80	11,92	65,69	63,83	63,16	2,01	1,97	1,80	0,36	2,46	7,00	2,85
M	Freestyle	Libre	50	Final	Final	5	5	0,61	1,01	3,86	5,24	9,84	14,52	19,36	21,81	11,97	62,94	62,50	60,61	2,07	2,02	1,82	0,40	2,85	8,70	3,05
M	Freestyle	Libre	50	Final	Final	1	6	0,61	0,96	2,94	5,42	9,94	14,64	19,52	21,92	11,98	60,81	59,21	56,60	2,18	2,12	1,99	0,35	1,98	5,60	2,83
M	Freestyle	Libre	50	Final	Final	8	7	0,68	0,94	3,78	5,44	10,12	15,00	19,70	22,02	11,90	61,64	59,60	58,25	2,08	2,10	2,00	0,26	2,84	7,80	2,75
M	Freestyle	Libre	50	Final	Final	7	8	0,68	0,96	3,50	5,40	10,14	14,94	19,98	22,19	12,05	60,40	58,06	58,82	2,10	2,10	2,08	0,28	2,54	7,40	2,91
						MEANS		0,63	0,96	3,37	5,35	9,92	14,68	19,52	21,85	11,93	63,05	60,89	59,78	2,08	2,06	1,94	0,33	2,41	7,00	2,94

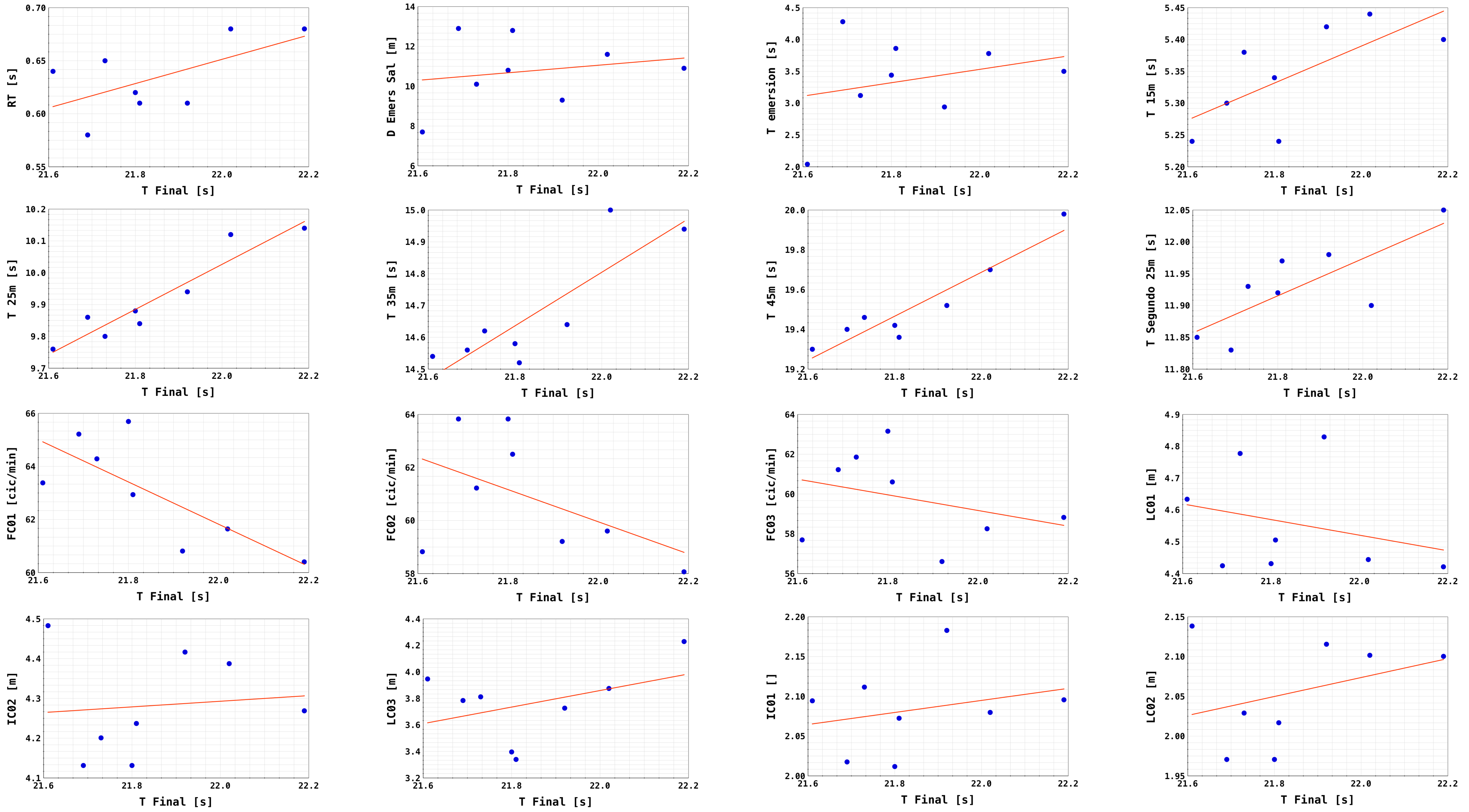

LEN European Aquatics Championships [BUDAPEST, Hungria] 17/05/2021 - 23/05/2021

50 Freestyle Male

						LANE	Pos.	RT	T_entr	T_emer	t15	t25	t35	t45	T50	2nd 25	F01	F02	F03	LC1	LC2	LC3	Flight_T	T_Underw_01	D_Underw	Speed Underw
M	Freestyle	Libre	50	Heat 7 of 8	Heat	5	1	0,64	1,00	3,82	5,28	9,90	14,48	19,32	21,76	11,86	64,29	62,94	60,61	2,02	2,02	1,83	0,36	2,82	7,80	2,77
M	Freestyle	Libre	50	Heat 6 of 8	Heat	4	1	0,67	1,06	4,06	5,34	9,96	14,60	19,32	21,86	11,90	61,64	56,96	53,10	2,11	2,25	2,00	0,39	3,00	8,70	2,90
M	Freestyle	Libre	50	Heat 8 of 8	Heat	4	1	0,57	0,98	4,34	5,30	9,88	14,56	19,42	21,93	12,05	64,75	60,81	57,69	2,02	2,07	1,86	0,41	3,36	9,00	2,68
M	Freestyle	Libre	50	Heat 8 of 8	Heat	2	2	0,69	0,98	3,36	5,34	9,98	14,74	19,60	21,97	11,99	60,00	59,21	53,57	2,16	2,11	2,13	0,29	2,38	7,00	2,94
M	Freestyle	Libre	50	Heat 7 of 8	Heat	4	2	0,68	1,02	3,32	5,44	10,04	14,70	19,54	21,98	11,94	62,07	59,60	56,60	2,16	2,12	1,95	0,34	2,30	6,40	2,78
M	Freestyle	Libre	50	Heat 6 of 8	Heat	6	2	0,69	1,02	2,14	5,46	10,08	14,76	19,56	21,99	11,91	61,22	58,44	58,25	2,12	2,17	1,91	0,33	1,12	3,40	3,04
M	Freestyle	Libre	50	Heat 7 of 8	Heat	7	3	0,66	0,98	3,52	5,18	9,92	14,62	19,54	22,00	12,08	60,40	55,90	53,10	2,10	2,23	2,07	0,32	2,54	7,50	2,95
M	Freestyle	Libre	50	Heat 8 of 8	Heat	6	3	0,62	0,96	3,02	5,44	10,04	14,72	19,54	22,01	11,97	58,82	56,25	51,28	2,22	2,25	2,13	0,34	2,06	6,00	2,91
M	Freestyle	Libre	50	Heat 7 of 8	Heat	2	4	0,65	1,06	2,76	5,54	10,16	14,84	19,72	22,05	11,89	59,21	55,56	57,69	2,19	2,26	2,01	0,41	1,70	5,20	3,06
M	Freestyle	Libre	50	Heat 8 of 8	Heat	5	4	0,61	0,96	3,16	5,28	10,00	14,76	19,54	22,08	12,08	69,23	65,22	65,93	1,84	1,93	1,61	0,35	2,20	6,70	3,05
M	Freestyle	Libre	50	Heat 6 of 8	Heat	2	4	0,61	0,98	4,38	5,34	9,96	14,78	19,66	22,13	12,17	62,94	59,21	57,14	2,06	2,09	1,91	0,37	3,40	9,10	2,68
M	Freestyle	Libre	50	Heat 7 of 8	Heat	9	5	0,62	0,96	4,20	5,28	9,90	14,62	19,54	22,15	12,25	58,82	56,60	51,28	2,21	2,20	2,02	0,34	3,24	8,40	2,59
M	Freestyle	Libre	50	Heat 6 of 8	Heat	8	5	0,67	1,00	3,50	5,52	10,14	14,84	19,70	22,19	12,05	64,29	62,07	61,22	2,02	2,02	1,77	0,33	2,50	6,70	2,68
M	Freestyle	Libre	50	Heat 8 of 8	Heat	3	5	0,63	0,96	3,96	5,34	10,06	14,88	19,74	22,19	12,13	62,07	60,81	61,22	2,05	2,04	1,80	0,33	3,00	8,30	2,77
M	Freestyle	Libre	50	Heat 7 of 8	Heat	6	6	0,67	1,00	4,34	5,38	10,24	14,94	19,80	22,24	12,00	59,21	58,44	59,41	2,09	2,15	1,86	0,33	3,34	9,00	2,69
M	Freestyle	Libre	50	Heat 6 of 8	Heat	0	6	0,63	0,90	4,32	5,58	10,20	15,00	19,78	22,27	12,07	60,00	57,69	57,69	2,16	2,17	1,88	0,27	3,42	8,90	2,60
						MEANS		0,64	0,99	3,64	5,38	10,03	14,74	19,58	22,05	12,02	61,81	59,11	57,24	2,09	2,13	1,92	0,34	2,65	7,38	2,82

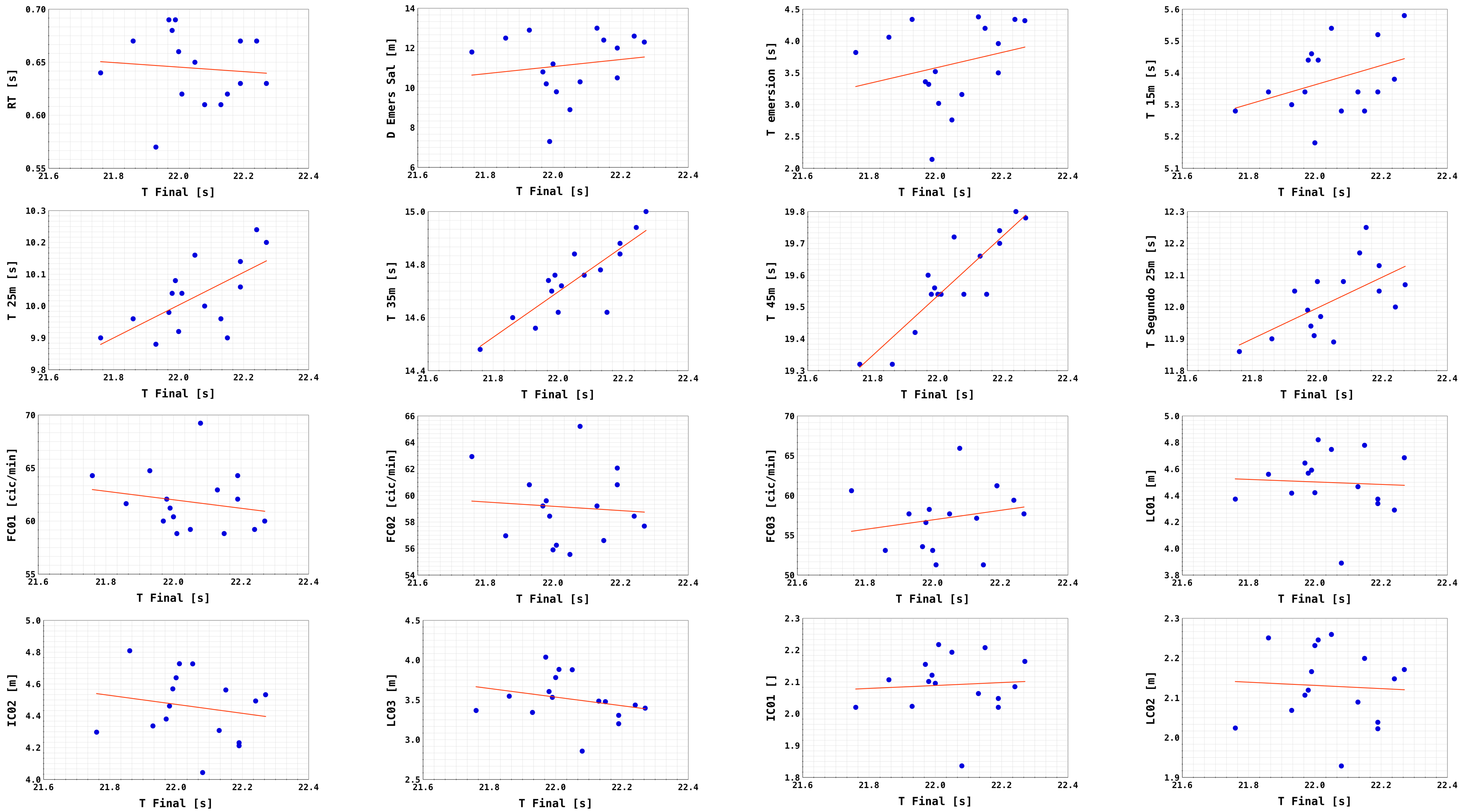

LEN European Aquatics Championships [BUDAPEST, Hungria] 17/05/2021 - 23/05/2021

50 Freestyle Male

						LANE	Pos.	RT	T_entr	T_emer	t15	t25	t35	t45	T50	2nd 25	F01	F02	F03	LC1	LC2	LC3	Flight_T	T_Underw_01	D_Underw	Speed Underw
M	Freestyle	Libre	50	Semifinal 2	SF	3	1	0,67	0,98	3,26	5,36	9,80	14,48	19,36	21,60	11,80	63,83	61,22	60,00	2,12	2,05	2,01	0,31	2,28	6,30	2,76
M	Freestyle	Libre	50	Semifinal 1	SF	4	1	0,63	1,02	4,14	5,18	9,72	14,48	19,20	21,67	11,95	63,83	59,60	55,56	2,07	2,12	1,97	0,39	3,12	8,80	2,82
M	Freestyle	Libre	50	Semifinal 1	SF	3	2	0,68	0,98	2,10	5,32	9,88	14,68	19,42	21,72	11,84	62,07	56,96	55,05	2,12	2,21	2,13	0,30	1,12	4,10	3,66
M	Freestyle	Libre	50	Semifinal 2	SF	4	2	0,64	0,98	3,76	5,40	9,90	14,66	19,52	21,80	11,90	65,22	62,94	62,50	2,04	1,98	1,89	0,34	2,78	7,60	2,73
M	Freestyle	Libre	50	Semifinal 2	SF	5	3	0,48	0,96	4,26	5,30	9,88	14,58	19,42	21,84	11,96	65,22	61,64	58,82	2,01	2,04	1,90	0,48	3,30	9,90	3,00
M	Freestyle	Libre	50	Semifinal 1	SF	5	3	0,63	0,97	3,56	5,30	9,92	14,74	19,52	21,86	11,94	61,64	59,60	56,60	2,11	2,10	2,04	0,34	2,59	7,60	2,93
M	Freestyle	Libre	50	Semifinal 1	SF	6	3	0,63	0,96	3,14	5,44	9,94	14,70	19,56	21,86	11,92	60,00	58,06	58,25	2,22	2,15	2,02	0,33	2,18	6,60	3,03
M	Freestyle	Libre	50	Semifinal 1	SF	1	5	0,60	0,93	3,94	5,38	9,98	14,70	19,62	21,95	11,97	62,94	61,22	58,25	2,07	2,03	1,99	0,33	3,01	8,10	2,69
M	Freestyle	Libre	50	Semifinal 1	SF	2	6	0,61	0,93	2,66	5,34	9,94	14,76	19,58	21,97	12,03	69,23	66,18	65,93	1,88	1,88	1,71	0,32	1,73	4,70	2,72
M	Freestyle	Libre	50	Semifinal 2	SF	2	4	0,69	1,06	2,72	5,68	10,24	14,98	19,84	22,07	11,83	59,21	57,69	54,55	2,22	2,17	2,22	0,37	1,66	5,40	3,25
M	Freestyle	Libre	50	Semifinal 1	SF	8	7	0,63	0,90	3,86	5,48	10,10	14,86	19,72	22,10	12,00	63,38	58,06	56,60	2,05	2,15	2,00	0,27	2,96	8,00	2,70
M	Freestyle	Libre	50	Semifinal 1	SF	7	7	0,63	0,96	4,54	5,34	10,04	14,80	19,78	22,10	12,06	56,60	55,21	54,55	2,26	2,23	2,13	0,33	3,58	9,70	2,71
M	Freestyle	Libre	50	Semifinal 2	SF	7	5	0,59	0,96	4,70	5,32	9,92	14,70	19,60	22,14	12,22	61,64	59,60	57,14	2,12	2,08	1,86	0,37	3,74	9,80	2,62
M	Freestyle	Libre	50	Semifinal 2	SF	6	6	0,67	1,03	3,20	5,34	10,02	14,96	19,92	22,21	12,19	61,22	57,69	55,56	2,09	2,10	2,12	0,36	2,17	6,60	3,04
M	Freestyle	Libre	50	Semifinal 2	SF	1	7	0,59	0,95	3,02	5,50	10,00	14,86	19,98	22,24	12,24	63,83	62,50	60,61	2,09	1,92	1,97	0,36	2,07	6,60	3,19
M	Freestyle	Libre	50	Semifinal 2	SF	8	8	0,67	1,00	4,02	5,38	10,18	15,00	19,94	22,32	12,14	65,22	61,22	60,00	1,92	2,01	1,89	0,33	3,02	8,60	2,85
						MEANS		0,63	0,97	3,55	5,38	9,97	14,75	19,62	21,97	12,00	62,82	59,96	58,12	2,09	2,08	1,99	0,35	2,58	7,40	2,92

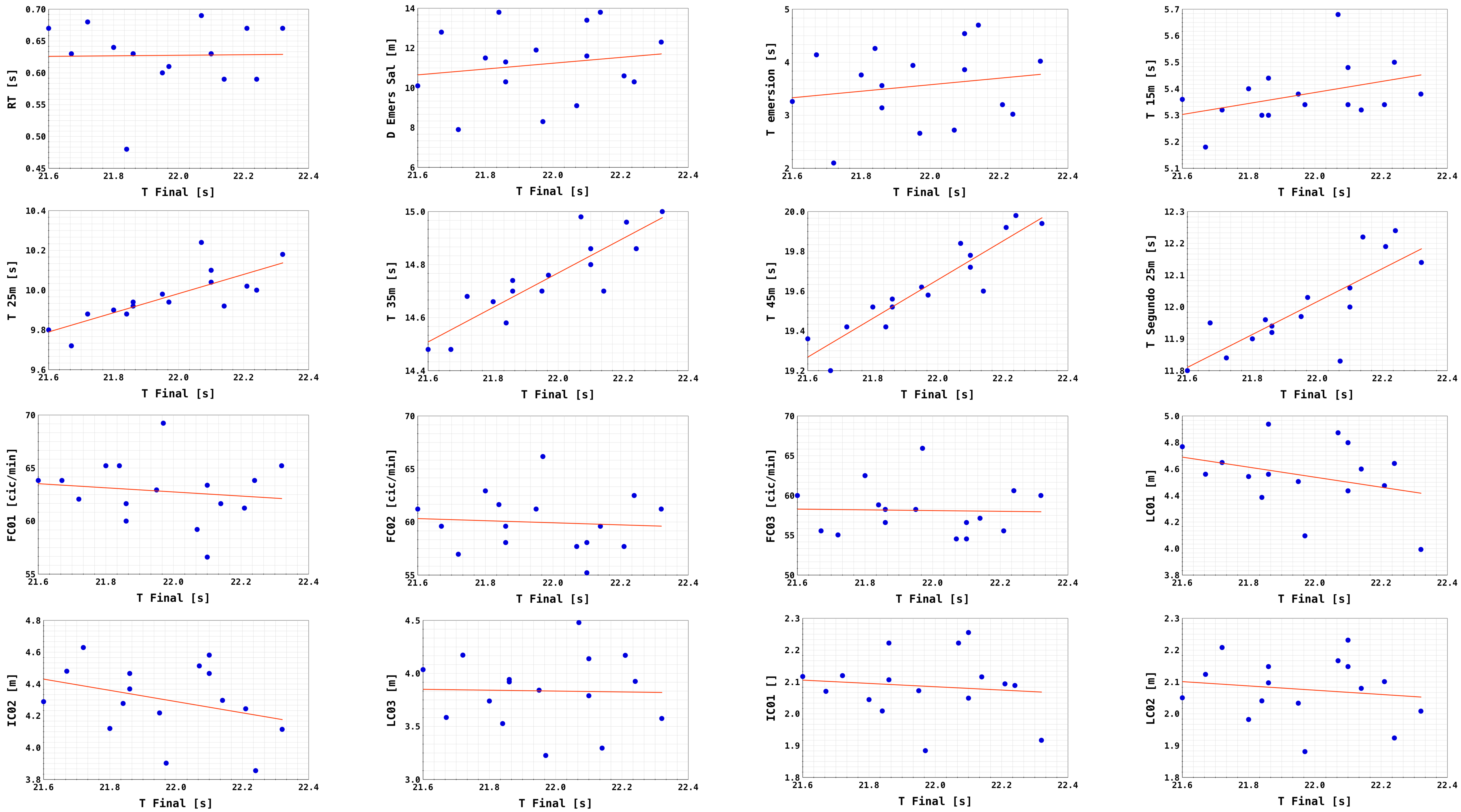

LEN European Aquatics Championships [BUDAPEST, Hungria] 17/05/2021 - 23/05/2021

50 Butterfly Male

						LANE	Pos.	RT	T_entr	T_emer	t15	t25	t35	t45	T50	2nd 25	F01	F02	F03	LC1	LC2	LC3	Flight_T	T_Underw_01	D_Underw	Speed Underw
M	Butterfly	Mariposa	50	Final	Final	6	1	0,61	0,96	4,48	5,08	10,10	15,20	20,38	23,00	12,90	62,50	61,22	61,22	1,91	1,91	1,68	0,35	3,52	9,90	2,81
M	Butterfly	Mariposa	50	Final	Final	4	2	0,60	0,98	3,44	5,50	10,30	15,26	20,38	23,01	12,71	66,18	64,75	61,22	1,89	1,84	1,68	0,38	2,46	6,80	2,76
M	Butterfly	Mariposa	50	Final	Final	5	3	0,66	0,96	4,44	5,52	10,44	15,42	20,56	23,08	12,64	68,70	65,22	59,41	1,78	1,82	1,80	0,30	3,48	9,30	2,67
M	Butterfly	Mariposa	50	Final	Final	8	4	0,73	1,02	4,22	5,26	10,20	15,26	20,44	23,09	12,89	67,67	62,50	59,41	1,79	1,87	1,72	0,29	3,20	9,10	2,84
M	Butterfly	Mariposa	50	Final	Final	7	5	0,63	0,98	4,24	5,22	10,16	15,22	20,46	23,14	12,98	68,70	67,16	63,16	1,77	1,73	1,60	0,35	3,26	9,10	2,79
M	Butterfly	Mariposa	50	Final	Final	3	6	0,63	0,98	4,08	5,30	10,40	15,42	20,58	23,18	12,78	67,16	63,38	63,83	1,75	1,86	1,63	0,35	3,10	8,80	2,84
M	Butterfly	Mariposa	50	Final	Final	1	7	0,62	0,98	4,74	5,52	10,60	15,68	20,90	23,54	12,94	63,83	61,64	54,05	1,85	1,89	1,89	0,36	3,76	9,60	2,55
M	Butterfly	Mariposa	50	Final	Final	2	8	0,68	0,98	4,28	5,46	10,52	15,64	20,82	23,56	13,04	62,07	60,81	61,22	1,91	1,92	1,61	0,30	3,30	9,00	2,73
MEANS								0,65	0,98	4,24	5,36	10,34	15,39	20,57	23,20	12,86	65,85	63,34	60,44	1,83	1,85	1,70	0,34	3,26	8,95	2,75

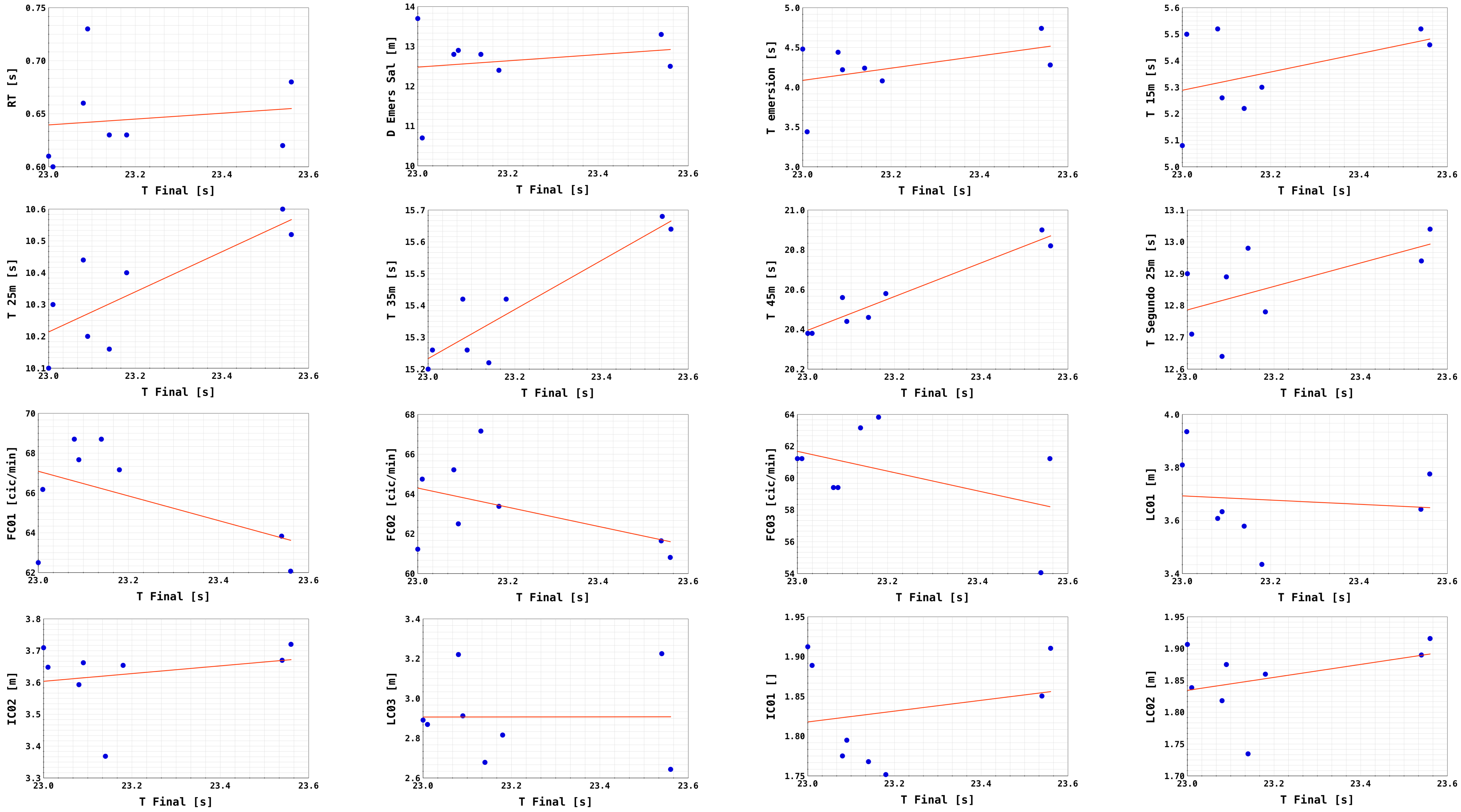

LEN European Aquatics Championships [BUDAPEST, Hungria] 17/05/2021 - 23/05/2021

50 Butterfly Male

						LANE	Pos.	RT	T_entr	T_emer	t15	t25	t35	t45	T50	2nd 25	F01	F02	F03	LC1	LC2	LC3	Flight_T	T_Underw_01	D_Underw	Speed Underw
M	Butterfly	Mariposa	50	Heat 6 of 7	Heat	4	1	0,62	0,96	4,50	5,12	10,18	15,32	20,44	23,03	12,85	63,38	61,64	58,82	1,87	1,90	1,77	0,34	3,54	9,80	2,77
M	Butterfly	Mariposa	50	Heat 6 of 7	Heat	5	2	0,61	0,96	4,62	5,48	10,52	15,60	20,72	23,32	12,80	65,69	62,07	60,00	1,81	1,90	1,73	0,35	3,66	9,70	2,65
M	Butterfly	Mariposa	50	Heat 5 of 7	Heat	4	1	0,64	1,00	3,92	5,34	10,42	15,48	20,78	23,39	12,97	65,22	61,64	60,00	1,81	1,88	1,72	0,36	2,92	8,30	2,84
M	Butterfly	Mariposa	50	Heat 7 of 7	Heat	3	1	0,69	0,98	4,68	5,46	10,48	15,68	20,86	23,39	12,91	60,81	56,96	63,83	1,97	2,03	1,67	0,29	3,70	9,90	2,68
M	Butterfly	Mariposa	50	Heat 7 of 7	Heat	5	1	0,50	1,00	3,98	5,26	10,36	15,46	20,60	23,39	13,03	68,70	66,67	60,00	1,71	1,76	1,61	0,50	2,98	9,40	3,15
M	Butterfly	Mariposa	50	Heat 5 of 7	Heat	6	2	0,63	0,98	3,90	5,44	10,48	15,58	20,82	23,41	12,93	62,94	60,40	60,61	1,89	1,92	1,72	0,35	2,92	9,10	3,12
M	Butterfly	Mariposa	50	Heat 7 of 7	Heat	4	3	0,63	1,04	3,60	5,64	10,48	15,54	20,76	23,41	12,93	63,38	60,81	57,69	1,96	1,92	1,77	0,41	2,56	7,00	2,73
M	Butterfly	Mariposa	50	Heat 6 of 7	Heat	6	3	0,68	1,02	4,70	5,38	10,40	15,48	20,74	23,44	13,04	66,18	61,22	62,50	1,81	1,90	1,60	0,34	3,68	9,70	2,64
M	Butterfly	Mariposa	50	Heat 5 of 7	Heat	1	3	0,64	0,98	3,92	5,36	10,40	15,56	20,74	23,45	13,05	66,18	65,69	62,50	1,80	1,77	1,59	0,34	2,94	9,30	3,16
M	Butterfly	Mariposa	50	Heat 5 of 7	Heat	5	4	0,65	1,08	3,20	5,80	10,80	15,80	20,89	23,47	12,67	60,00	57,69	57,14	2,00	2,06	1,83	0,43	2,12	6,10	2,88
M	Butterfly	Mariposa	50	Heat 7 of 7	Heat	2	4	0,60	0,96	4,94	5,36	10,50	15,68	20,94	23,52	13,02	63,38	61,64	57,69	1,84	1,86	1,81	0,36	3,98	10,50	2,64
M	Butterfly	Mariposa	50	Heat 6 of 7	Heat	7	4	0,65	1,00	4,56	5,42	10,48	15,68	20,92	23,53	13,05	54,55	52,63	54,55	2,17	2,18	1,90	0,35	3,56	9,70	2,72
M	Butterfly	Mariposa	50	Heat 7 of 7	Heat	1	5	0,68	1,12	4,70	5,28	10,36	15,50	20,92	23,54	13,18	62,50	61,64	61,22	1,89	1,84	1,68	0,44	3,58	9,60	2,68
M	Butterfly	Mariposa	50	Heat 5 of 7	Heat	7	6	0,62	1,06	4,64	5,38	10,50	15,60	20,90	23,55	13,05	64,29	61,64	61,86	1,82	1,87	1,65	0,44	3,58	9,60	2,68
M	Butterfly	Mariposa	50	Heat 6 of 7	Heat	3	5	0,64	1,04	4,78	5,26	10,40	15,60	20,80	23,55	13,15	62,94	60,40	58,25	1,85	1,91	1,69	0,40	3,74	10,00	2,67
M	Butterfly	Mariposa	50	Heat 5 of 7	Heat	3	7	0,62	0,98	4,48	5,56	10,60	15,66	20,86	23,57	12,97	63,83	62,07	60,00	1,87	1,88	1,66	0,36	3,50	9,00	2,57
					MEANS			0,63	1,01	4,32	5,41	10,46	15,58	20,79	23,44	12,98	63,37	60,93	59,79	1,88	1,91	1,71	0,38	3,31	9,17	2,79

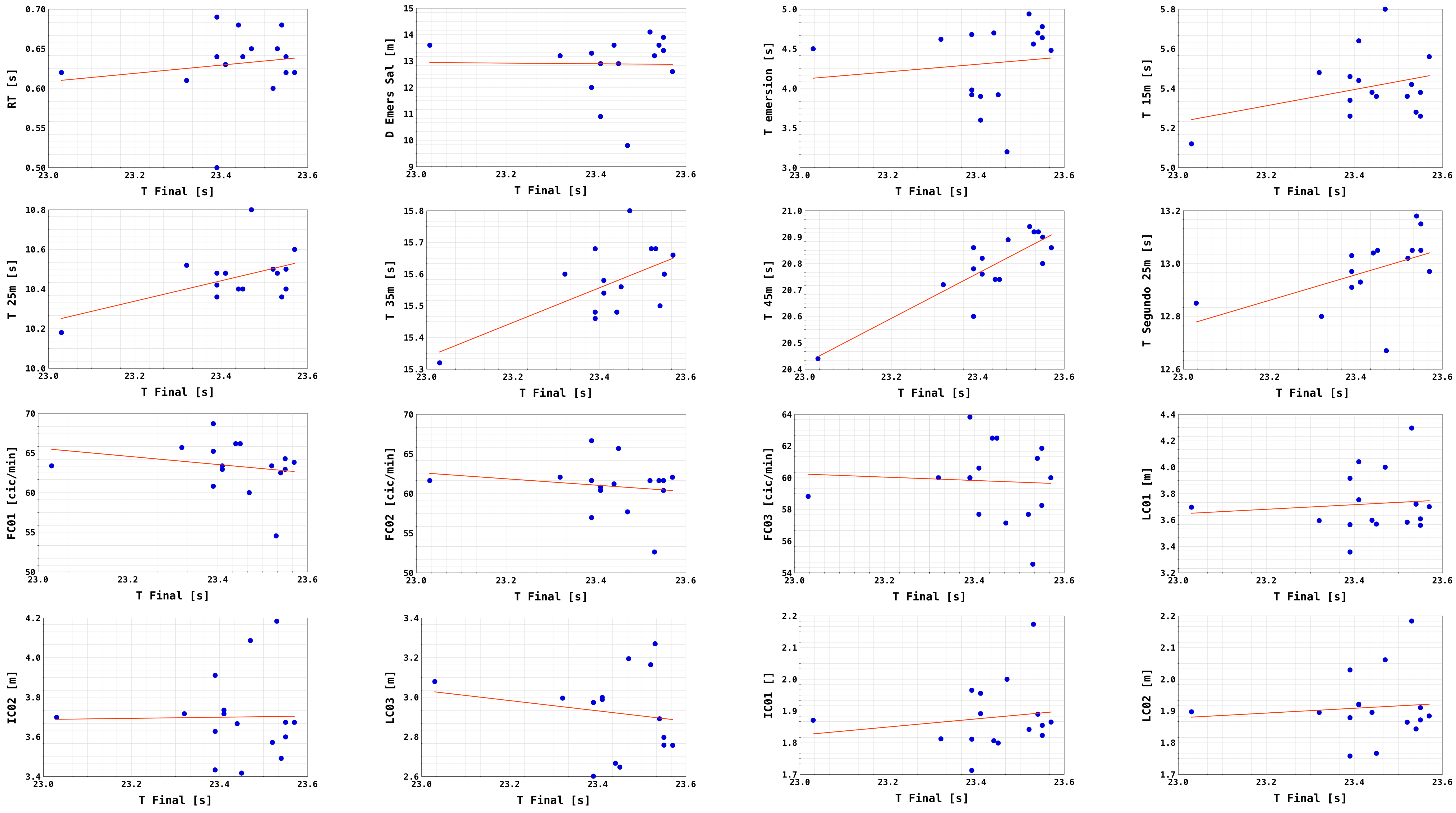

LEN European Aquatics Championships
[BUDAPEST, Hungria] 17/05/2021 - 23/05/2021

50 Butterfly Male

						LANE	Pos.	RT	T_entr	T_emer	t15	t25	t35	t45	T50	2nd 25	F01	F02	F03	LC1	LC2	LC3	Flight_T	T_Underw_01	D_Underw	Speed Underw
M	Butterfly	Mariposa	50	Semifinal 2	SF	6	1	0,61	1,00	3,44	5,40	10,28	15,18	20,26	22,97	12,69	66,67	62,94	63,83	1,84	1,91	1,56	0,39	2,44	6,90	2,83
M	Butterfly	Mariposa	50	Semifinal 1	SF	4	1	0,68	0,98	4,80	5,44	10,46	15,52	20,54	23,02	12,56	67,16	66,18	63,83	1,78	1,80	1,71	0,30	3,82	10,10	2,64
M	Butterfly	Mariposa	50	Semifinal 2	SF	5	2	0,49	1,00	4,10	5,30	10,32	15,40	20,48	23,10	12,78	62,07	63,38	60,61	1,93	1,86	1,70	0,51	3,10	8,80	2,84
M	Butterfly	Mariposa	50	Semifinal 2	SF	4	2	0,66	1,00	4,52	5,08	10,12	15,22	20,48	23,10	12,98	64,29	63,38	64,52	1,85	1,83	1,60	0,34	3,52	9,90	2,81
M	Butterfly	Mariposa	50	Semifinal 2	SF	3	4	0,68	0,94	4,50	5,54	10,48	15,54	20,64	23,31	12,83	62,50	61,22	58,82	1,94	1,93	1,72	0,26	3,56	9,70	2,72
M	Butterfly	Mariposa	50	Semifinal 1	SF	5	2	0,66	1,00	4,04	5,24	10,28	15,40	20,66	23,34	13,06	70,31	67,16	64,52	1,69	1,72	1,56	0,34	3,04	9,50	3,13
M	Butterfly	Mariposa	50	Semifinal 1	SF	8	3	0,62	1,00	4,52	5,48	10,50	15,44	20,78	23,35	12,85	63,38	62,94	57,69	1,89	1,85	1,82	0,38	3,52	10,10	2,87
M	Butterfly	Mariposa	50	Semifinal 1	SF	6	4	0,66	1,04	5,68	5,36	10,38	15,46	20,70	23,36	12,98	62,94	59,60	60,61	1,90	1,95	1,67	0,38	4,64	9,70	2,09
M	Butterfly	Mariposa	50	Semifinal 1	SF	3	5	0,66	1,00	3,96	5,48	10,52	15,70	20,76	23,39	12,87	63,38	61,64	60,61	1,88	1,90	1,69	0,34	2,96	9,30	3,14
M	Butterfly	Mariposa	50	Semifinal 1	SF	1	5	0,63	0,96	4,78	5,26	10,38	15,46	20,78	23,39	13,01	66,18	62,07	60,61	1,77	1,86	1,71	0,33	3,82	10,20	2,67
M	Butterfly	Mariposa	50	Semifinal 2	SF	1	5	0,65	1,08	4,52	5,18	10,28	15,44	20,84	23,41	13,13	68,18	63,83	60,00	1,73	1,78	1,75	0,43	3,44	9,20	2,67
M	Butterfly	Mariposa	50	Semifinal 1	SF	7	7	0,65	1,00	4,68	5,42	10,46	15,58	20,82	23,43	12,97	54,88	54,55	54,55	2,17	2,12	1,90	0,35	3,68	10,00	2,72
M	Butterfly	Mariposa	50	Semifinal 2	SF	7	6	0,61	0,98	4,98	5,40	10,60	15,76	20,96	23,47	12,87	60,40	61,22	61,22	1,91	1,89	1,76	0,37	4,00	10,20	2,55
M	Butterfly	Mariposa	50	Semifinal 2	SF	2	7	0,66	1,02	4,02	5,40	10,48	15,60	20,76	23,51	13,03	68,18	66,18	63,83	1,73	1,76	1,54	0,36	3,00	9,30	3,10
M	Butterfly	Mariposa	50	Semifinal 1	SF	2	8	0,66	1,06	3,36	5,78	10,76	15,84	20,98	23,57	12,81	59,60	55,90	55,05	2,02	2,10	1,89	0,40	2,30	6,50	2,83
M	Butterfly	Mariposa	50	Semifinal 2	SF	8	8	0,60	1,04	4,52	5,54	10,52	15,62	20,78	23,59	13,07	64,75	63,38	61,22	1,86	1,85	1,57	0,44	3,48	9,40	2,70
						MEANS		0,64	1,01	4,40	5,39	10,43	15,51	20,70	23,33	12,91	64,05	62,22	60,72	1,87	1,88	1,70	0,37	3,39	9,30	2,77

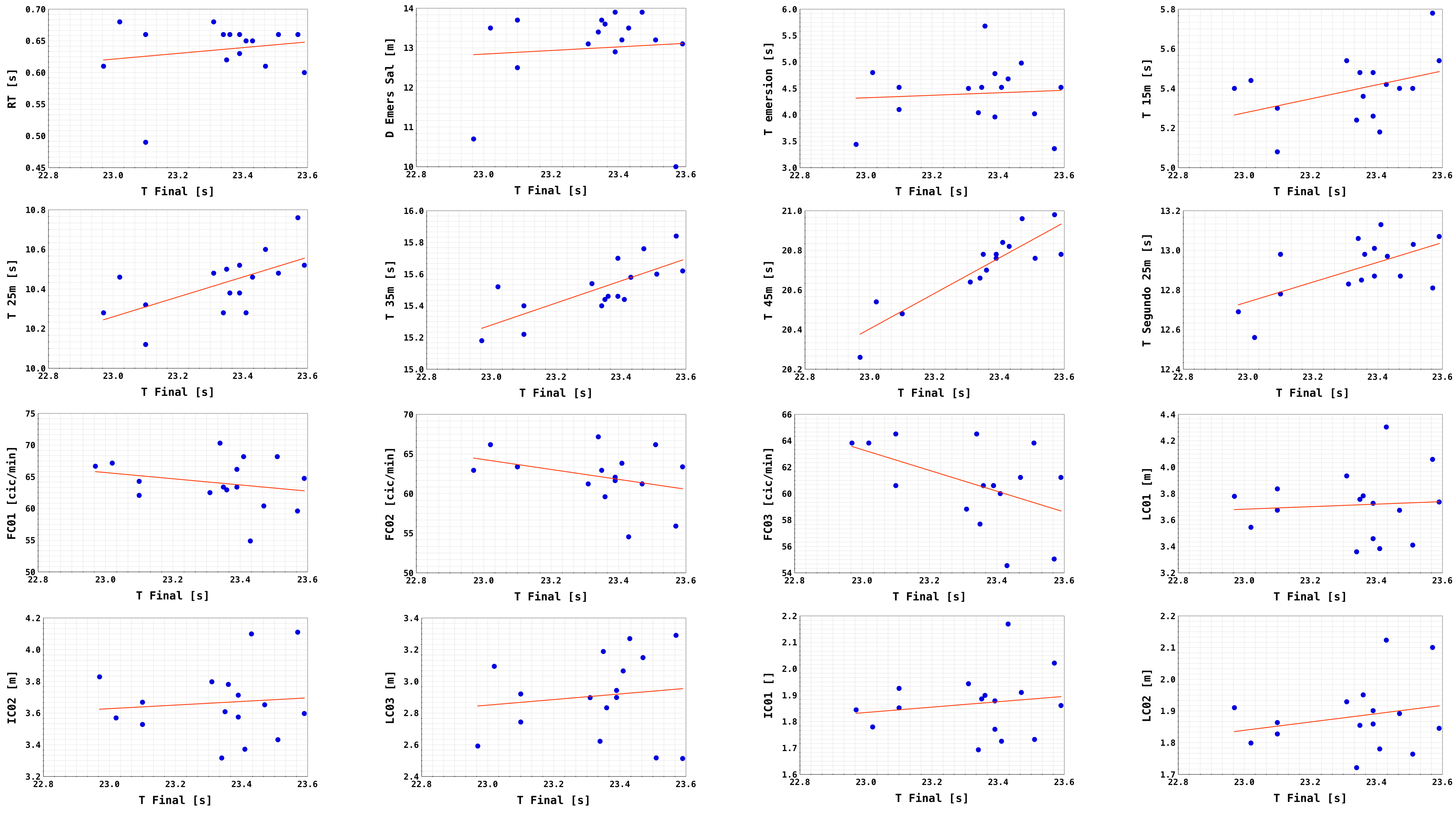

Supplement: Supplementary file 3 [file DataSheet1.PDF]
